# Supplementary material for: Quality of life, healthcare use and costs in ‘at-risk’ children after early antibiotic treatment versus placebo for influenza-like illness: within-trial descriptive economic analyses of the ARCHIE randomised controlled trial
Source: BMJ Open. 2022 Apr 15;12(4):e049373. doi: 10.1136/bmjopen-2021-049373 (PMC9014043; doi:10.1136/bmjopen-2021-049373)
Supplement: Supplementary data [file bmjopen-2021-049373supp001.pdf]

Supplemental material for:

Quality of life, healthcare use and costs in 'at risk' children after early antibiotic treatment vs. placebo for influenza-like illness: Within-trial descriptive economic analyses of the ARCHIE randomised controlled trial

Authors

Ines Rombach (ines.rombach@ndorms.ox.ac.uk)[1],

Kay Wang (kay.wang@phc.ox.ac.uk)[2],

Sharon Tonner (sharon.tonner@phc.ox.ac.uk)[2],

Jenna Grabey (jenna.grabey@phc.ox.ac.uk)[2],

Anthony Harnden (anthony.harnden@phc.ox.ac.uk)[2],

Jane Wolstenholme (jane.wolstenholme@ndph.ox.ac.uk)[1],

ARCHIE Investigators

Affilitations

[1]Health Economics Research Centre, Nuffield Department of Population Health, Oxford University, Oxford, UK

[2]Nuffield Department of Primary Care Health Sciences, University of Oxford, Oxford, UK

Corresponding author:
Ines Rombach
Health Economics Research Centre
Nuffield Department of Population Health
Old Road Campus
University of Oxford
Oxford
OX3 7LF
United Kingdom
ines.rombach@ndorms.ox.ac.uk
https://orcid.org/0000-0003-3464-3867

Published in BMJ Open

Table of Contents

Supplemental File 1: Unit costs 3
Supplemental Table S1: Unit costs 3
Supplemental File 2: Availability of data reported on behalf of the children, and by the children 6
Supplemental Table S2a: Available EQ-5D-Y data for participants aged less than 2 years 6
Supplemental Table S2b: Available EQ-5D-Y data for participants aged 2+ years 8
Supplemental Table S2c: Missing data pattern over time for the EQ-5D-Y proxy, completed on behalf of the child 9
Supplemental Table S2d: Proxy-reported EQ-5D-Y scores by data availability at the subsequent follow-up 9
Supplemental Table S2e: Missing data pattern for the EQ-5D-Y domains (proxy data, completed on behalf of the child) 10
Supplemental Table S2f: Available EQ-5D-Y items 11
Supplemental Table S2g: Available EQ-5D-Y items for children aged less than 2 years 12
Supplemental Table S2h: Available EQ-5D-Y items for children aged 2+ years (proxy) 13
Supplemental File 3: Reponses to the EQ-5D-Y items 14
Supplemental Table S3a: Responses to EQ-5D-Y domains (proxy, completed on behalf of child) 15
Supplemental Table S3b: Responses to EQ-5D-Y domains (proxy, completed on behalf of child for children aged less than 2 years) 16
Supplemental Table S3c: Responses to EQ-5D-Y domains (proxy, completed on behalf of child for children aged 2+ years) 17
Supplemental Table S3d: Responses to EQ-5D-Y domains (completed by child) 18
Supplemental Table S3e: Responses to EQ-5D-Y domains (completed by child for children aged less than 2 years) 19
Supplemental Table S3f: Responses to EQ-5D-Y domains (completed by child for children aged 2+ years) 20
Supplemental Table 3g: Responses to CARIFS domains (only including questionnaires with sufficient data to calculate the overall CARIFS score) 21
Supplemental Table 3h: Responses to CARIFS domains for children less than 2 years old (only including questionnaires with sufficient data to calculate the overall CARIFS score) 24
Supplemental Table 3i: Responses to CARIFS domains for children aged 2+ years (only including questionnaires with sufficient data to calculate the overall CARIFS score) 26
Supplemental File 4: Additional summaries of research use 29
Supplemental Table 4a: Details of all reported hospital admissions 29
Supplemental Table 4b: Overview of healthcare use for re-consultations due to clinical deterioration and hospitalisations related to the illness episode for which the child was randomised to ARCHIE 30
Supplemental File 5: Number of days children were unable to attend school or nursery, and subsequent changes to childcare requirements 31
Supplemental Table 5: Daily activity and child care 31

Supplemental File 1: Unit costs

Supplemental Table S1: Unit costs

Cost item	Cost	Data Source
Re-consultations primary care (GP)	£31	Personal Social Services Research Unit (PSSRU), online unit cost database of health and social care professionals 2017/2018: GP including direct care, cost per surgery lasting 9.22 minutes.
Re-consultation in paediatric ambulatory assessment units	£201	Weighted average of consultant-led and non-consultant led non-admitted face-to-face follow-up attendance for paediatric respiratory medicine; National Schedule of reference costs 2017/2018, CL and NCL tab.
Chest x-ray	£231	Outpatient procedures, paediatric same-day diagnostic imaging; National Schedule of reference costs 2017/2018, OPROC tab. (Note: the same cost was applied to chest x-rays at re-consultation and during hospital admission, as it was assumed that x-rays for the ARCHIE participants would have to be performed on the same day)
Night in hospital	£652	Weighted average of cost per day for non-elective paediatric hospital stays; National Schedule of reference costs 2017/2018, NEL, NEL_XS and NES tab.
Emergency department	£160	Weighted average of all emergency department attendances excluding dental care and patient dead on arrival, National Schedule of reference costs 2017/2018, AE tab.
**Day case**	£610	
**Investigations performed**		
Blood test	£1	Cost of clinical biochemistry, National Schedule of reference costs 2017/2018, DAPS tab.
Nasal aspirate/ npa	£8	Cost of microbiological diagnostic test, National Schedule of reference costs 2017/2018, DAPS tab.
Throat swab	£8	Cost of microbiological diagnostic test, National Schedule of reference costs 2017/2018, DAPS tab.
Urine test	£2.80	Dipstick £0.20 + Laboratory culture £2.60, Fenwick et al[10]

**Antibiotics and other drugs prescribed**		Costed in line with British National Formulary for Children – accessed 22Aug2019.<br>The minimum Drug Tariff is used for all costs.
**Antibiotics**		
Amoxicillin	£1.26	Assume all amoxicillin given as oral suspension.<br>Amoxicillin (as Amoxicillin trihydrate) 50 mg per 1 ml – 100 ml cost £1.26
Azithromycin	£4.06	Assume all AZITHROMYCIN given as oral suspension.<br>Azithromycin 40 mg per 1 ml – 15 ml cost £4.06
Ceftriaxone	£9.58	By IV. One vial of Ceftriaxone (as Ceftriaxone sodium) 1g. 1 vial £9.58
Chloramphenicol	£1.55	Assume used as eye drops. Cost by vial.<br>Chloramphenicol 5 mg per 1 ml. 10 ml cost £1.55
Clarithromycin	£3.36	Assume oral suspension used, as tablet doses too high.<br>• Clarithromycin 25 mg per 1 ml. 70 ml cost £3.36
**Study intervention: Co-amoxiclav 400/57**	£4.13 children below 7<br>£5.79 children 7+	Amoxicillin (as Amoxicillin trihydrate) 80 mg per 1 ml, Clavulanic acid (as Potassium clavulanate) 11.4 mg per 1 ml<br>35 ml: £4.13, 70 ml: £5.79<br>Note: in the trial, co-amoxiclav 400/57 was provided by Brown & Burk UK Ltd. This was the only company who was able to procure a placebo suitable for use in this study. Recommendations from the study will not be restricted to this manufacturer. Therefore, the costing will also include other manufacturers, whose products are available in 35ml and 70 ml.
Co-amoxiclav	£1.65	Assume oral suspension.<br>Amoxicillin (as Amoxicillin trihydrate) 25 mg per 1 ml, Clavulanic acid (as Potassium clavulanate) 6.25 mg per 1 ml. (lower dose than study drug)<br>➔100ml cost £1.65
Dexamethasone	£21.15	Dexamethasone (as Dexamethasone sodium phosphate) 400 microgram per 1 ml. 150 ml cost £42.30.
Erythromycin ethyl succinate	£5.21	Assume oral suspension.<br>Erythromycin (as Erythromycin ethyl succinate) 25 mg per 1 ml. 100ml cost £5.21
Phenoxymethylpenicillin	£4.28	Assume oral solution.<br>Phenoxymethylpenicillin (as Phenoxymethylpenicillin potassium) 25 mg per 1 ml. 100 ml costs £4.28
**Other drugs**		

Atrovent	£5.56	Ipratropium bromide 20 microgram per 1 dose. Inhaler with 200 doses costs £5.56
Beclomethasone /unidentified steroids	£3.70	Beclometasone dipropionate 50 microgram per 1 dose. 200 dose inhaler costs £3.70.<br>(this was also used for unidentified steroids)
Dexamethasone	£42.30	Assume oral solution.<br>Dexamethasone (as Dexamethasone sodium phosphate) 400 microgram per 1 ml. 150ml cost £42.30
Fluticasone	£6.53	Fluticasone propionate 25 microgram per 1 dose. 120 dose inhaler costs £6.53
Fluticasone	£21.26	Inhaler is used.<br>Fluticasone propionate 125 microgram per 1 dose. £21.26 for 120 doses.
Ibuprofen	£4.20	Assume oral suspension. Ibuprofen 40 mg per 1 ml. 100 ml cost £4.20 (NHS indicative price, no drug tariff available)
Ipratropium bromide 20 mcg	£5.56	Inhaler: Ipratropium bromide 20 microgram per 1 dose. 200 doses cost £5.56.
Magnesium sulphate	£1.735	Magnesium sulfate heptahydrate 500 mg per 1 ml; 10 ampoule cost £17.35
Oxygen	£0.95	Supplemental oxygen per hour, provided by mask or nasal, was estimated to be £0.80 for NHS Lothian in 2013. Prices have been adjusted to their value in 2018 based on a discount rate of 3.5% per year, as recommended for the UK.
Paracetemol 300 mg	£3.78	Oral suspension paediatrics, 120mg/5ml. 200 ml cost £3.78
Prednisolone 5 mg	£11.41	Oral solution: Prednisolone 10 mg per 1 ml: £11.41 for 10 unit dose
Salbutamol/ ventolin	£1.50	Salbutamol (as Salbutamol sulfate) 100 microgram per 1 dose. 200 doses £1.50
Salmeterol 25 mcg	£29.26	Salmeterol (as Salmeterol xinafoate) 25 microgram per 1 dose; 120 doses £29.26
Seretide	£21.26	Fluticasone – costed as above

Supplemental File 2: Availability of data reported on behalf of the children, and by the children

Availability of EQ-5D-Y data

For the EQ-5D-Y, the versions completed by the proxy on behalf of the children are more complete. The trial team considered substituting by available information from the EQ-5D-Y completed by the children themselves if data were missing from the EQ-5D-Y proxy, to enable a more complete analysis dataset. However, this approach yielded only an additional 1, 2, 3, 4 and 2 observations at days 0, 4, 7, 14 and 28, and was therefore abandoned.

For 54 participants (20%), proxy-completed EQ-5D-Y index data are available at baseline and all follow-up time points.

Supplemental Table S2a: Available EQ-5D-Y data for participants aged less than 2 years

	Co-amoxiclav (N=45)	Placebo (N=44)	Total (N=89)
**Availability of EQ-5D-Y index completed on behalf of child**			
**day 0**	18 (40%)	24 (55%)	42 (47%)
**day 4**	9 (20%)	12 (27%)	21 (24%)
**day 7**	9 (20%)	14 (32%)	23 (26%)
**day 14**	10 (22%)	13 (30%)	23 (26%)
**day 28**	11 (24%)	11 (25%)	22 (25%)
**Availability of EQ-5D VAS index completed by child**			
**day 0**	39 (87%)	43 (98%)	82 (92%)
**day 4**	27 (60%)	29 (66%)	56 (63%)
**day 7**	27 (60%)	28 (64%)	55 (62%)
**day 14**	22 (49%)	24 (55%)	46 (52%)
**day 28**	19 (42%)	19 (43%)	38 (43%)
**Availability of EQ-5D-Y index completed by child**			
**day 0**	0 (0%)	0 (0%)	0 (0%)
**day 4**	0 (0%)	1 (2%)	1 (1%)
**day 7**	0 (0%)	1 (2%)	1 (1%)
**day 14**	0 (0%)	1 (2%)	1 (1%)
**day 28**	0 (0%)	1 (2%)	1 (1%)
**Availability of EQ-5D VAS index completed by child**			
**day 0**	0 (0%)	1 (2%)	1 (1%)
**day 4**	3 (7%)	1 (2%)	4 (4%)
**day 7**	3 (7%)	1 (2%)	4 (4%)
**day 14**	3 (7%)	1 (2%)	4 (4%)
**day 28**	2 (4%)	1 (2%)	3 (3%)

Rombach I, *et al. BMJ Open* 2022; 12:e049373. doi: 10.1136/bmjopen-2021-049373

**Availability of CARIFS**			
**day 0**	27 (60%)	29 (66%)	56 (63%)
**day 7**	21 (47%)	23 (52%)	44 (49%)
**Availability of CARIFS VAS**			
**day 0**	44 (98%)	44 (100%)	88 (99%)
**day 7**	26 (58%)	28 (64%)	54 (61%)

Supplemental Table S2b: Available EQ-5D-Y data for participants aged 2+ years

	Co-amoxiclav (N=91)	Placebo (N=91)	Total (N=182)
**Availability of EQ-5D-Y index completed on behalf of child**			
**day 0**	78 (86%)	84 (92%)	162 (89%)
**day 4**	43 (47%)	40 (44%)	83 (46%)
**day 7**	43 (47%)	40 (44%)	83 (46%)
**day 14**	34 (37%)	33 (36%)	67 (37%)
**day 28**	30 (33%)	20 (22%)	50 (27%)
**Availability of EQ-5D-Y index completed by child**			
**day 0**	81 (89%)	86 (95%)	167 (92%)
**day 4**	50 (55%)	47 (52%)	97 (53%)
**day 7**	51 (56%)	45 (49%)	96 (53%)
**day 14**	39 (43%)	33 (36%)	72 (40%)
**day 28**	34 (37%)	21 (23%)	55 (30%)
**Availability of EQ-5D-Y index completed by child**			
**day 0**	32 (35%)	29 (32%)	61 (34%)
**day 4**	16 (18%)	12 (13%)	28 (15%)
**day 7**	17 (19%)	10 (11%)	27 (15%)
**day 14**	17 (19%)	9 (10%)	26 (14%)
**day 28**	17 (19%)	9 (10%)	26 (14%)
**Availability of EQ-5D VAS index completed by child**			
**day 0**	28 (31%)	24 (26%)	52 (29%)
**day 4**	16 (18%)	12 (13%)	28 (15%)
**day 7**	16 (18%)	10 (11%)	26 (14%)
**day 14**	17 (19%)	9 (10%)	26 (14%)
**day 28**	16 (18%)	10 (11%)	26 (14%)
**Availability of CARIFS**			
**day 0**	72 (79%)	68 (75%)	140 (77%)
**day 7**	45 (49%)	34 (37%)	79 (43%)
**Availability of CARIFS VAS**			
**day 0**	89 (98%)	91 (100%)	180 (99%)
**day 7**	50 (55%)	46 (51%)	96 (53%)

Rombach I, *et al. BMJ Open* 2022; 12:e049373. doi: 10.1136/bmjopen-2021-049373

Supplemental Table S2c: Missing data pattern over time for the EQ-5D-Y proxy, completed on behalf of the child

Missing data pattern*	N	%
+....	104	38.4%
+++++	54	19.9%
.....	48	17.7%
++++.	19	7.0%
+++..	19	7.0%
.++++	5	1.8%
...++	3	1.1%
..+..	3	1.1%
....+	3	1.1%
+++.+	2	0.7%
+..++	2	0.7%
.+++.	2	0.7%
...+.	2	0.7%
+.+++	1	0.4%
++.+.	1	0.4%
++..+	1	0.4%
..+++	1	0.4%
++...	1	0.4%

**+ indicates data are available, . indicates data are missing. The first character refers to the baseline EQ-5D-Y, the second to the 4 day assessment etc.*
For example, the first line of the table (+….) describes participants for whom baseline data, but no data for the subsequent four follow-up visits are available.

Supplemental Table S2d: Proxy-reported EQ-5D-Y scores by data availability at the subsequent follow-up

This table shows EQ-5D-Y values (as reported by their proxies on the children's behalf) at each time point spit by whether or not data was available for the subsequent time point. We hypothesised that those with unavailable data at the subsequent time point would be likely to have worth health states.

There was no consistent pattern to support this hypothesis.

	Participants for whom subsequent follow-up is available	Participants for whom subsequent follow-up is not available
**Day 0**	0.549 (0.396), N=97	0.606 (0.335), N=107
**Day 4**	0.671 (0.318), N=101	0.286 (0.671), N=3
**Day 7**	0.780 (0.339), N=82	0.759 (0.394), N=24
**Day 14**	0.882 (0.280), N=66	0.888 (0.217), N=24

Supplemental Table S2e: Missing data pattern for the EQ-5D-Y domains (proxy data, completed on behalf of the child)

	**Baseline**		**Day 4**		**Day 7**		**Day 14**		**Day 28**	
	Frequency	%	Frequency	%	Frequency	%	Frequency	%	Frequency	%
+++++	204	75.3%	104	38.4%	106	39.1%	90	33.2%	72	26.6%
. . . . .	5	1.8%	117	43.2%	122	45%	153	56.5%	179	66.1%
+.+++	38	14%	25	9.2%	21	7.7%	13	4.8%	8	3%
. .+++	15	5.5%	16	5.9%	16	5.9%	13	4.8%	9	3.3%
.++++	2	0.7%	3	1.1%	2	0.7%	1	0.4%	1	0.4%
+.++.	2	0.7%	1	0.4%	-	-	-	-	-	-
. . .++	2	0.7%	3	1.1%	-	-	-	-	-	-
++++.	1	0.4%	-	-	-	-	-	-	-	-
. .++.	1	0.4%	-	-	-	-	-	-	-	-
. . .+.	1	0.4%	-	-	-	-	-	-	-	-
+++. .	-	-	1	0.4%	-	-	-	-	-	-
+. .++	-	-	1	0.4%	-	-	-	-	-	-
+.+.+	-	-	-	-	1	0.4%	-	-	-	-
+. . .+	-	-	-	-	1	0.4%	-	-	-	-
. . .++	-	-	-	-	1	0.4%	1	0.4%	-	-
. . . .+	-	-	-	-	1	0.4%	-	-	-	-
++++.	-	-	-	-	-	-	-	-	2	0.7%

**+ indicates data are available, . indicates data are missing. The first character refers to the first EQ-5D item (mobility), the second to the second question (self-care) etc. The pattern +.+++ indicates that the answer to the self-care question is missing, while all other responses are available.*

Particularly the items on self-care and mobility are left incomplete when other questions have been answered.

Rombach I, *et al. BMJ Open* 2022; 12:e049373. doi: 10.1136/bmjopen-2021-049373

Supplemental Table S2f: Available EQ-5D-Y items

	Co-amoxiclav (N=136)						Placebo (N=135)						Total (N=271)					
	**M**	**SC**	**UA**	**PD**	**AD**	**VAS**	**M**	**SC**	**UA**	**PD**	**AD**	**VAS**	**M**	**SC**	**UA**	**PD**	**AD**	**VAS**
**Availability of EQ-5D-3L index proxy, completed on behalf of child**																		
Day 0	120 (88%)	**97 (71%)**	128 (94%)	131 (96%)	127 (93%)	120 (88%)	125 (93%)	**110 (81%)**	135 (100%)	135 (100%)	134 (99%)	129 (96%)	245 (90%)	**207 (76%)**	263 (97%)	266 (98%)	261 (96%)	249 (92%)
Day 4	69 (51%)	**54 (40%)**	75 (55%)	76 (56%)	76 (56%)	77 (57%)	63 (47%)	**54 (40%)**	75 (56%)	77 (57%)	76 (56%)	76 (56%)	132 (49%)	**108 (40%)**	150 (55%)	153 (56%)	152 (56%)	153 (56%)
Day 7	68 (50%)	**52 (38%)**	74 (54%)	75 (55%)	76 (56%)	78 (57%)	61 (45%)	**56 (41%)**	72 (53%)	71 (53%)	73 (54%)	73 (54%)	129 (48%)	**108 (40%)**	146 (54%)	146 (54%)	149 (55%)	151 (56%)
Day 14	53 (39%)	**44 (32%)**	59 (43%)	59 (43%)	59 (43%)	61 (45%)	50 (37%)	**47 (35%)**	58 (43%)	59 (44%)	59 (44%)	57 (42%)	103 (38%)	**91 (34%)**	117 (43%)	118 (44%)	118 (44%)	118 (44%)
Day 28	48 (35%)	**43 (32%)**	53 (39%)	53 (39%)	51 (38%)	53 (39%)	34 (25%)	**32 (24%)**	39 (29%)	39 (29%)	39 (29%)	40 (30%)	82 (30%)	**75 (28%)**	92 (34%)	92 (34%)	90 (33%)	93 (34%)
**Availability of EQ-5D-3L index completed by child**																		
Day 0	32 (24%)	32 (24%)	32 (24%)	32 (24%)	32 (24%)	32 (24%)	30 (22%)	30 (22%)	31 (23%)	31 (23%)	30 (22%)	32 (24%)	62 (23%)	62 (23%)	63 (23%)	63 (23%)	62 (23%)	64 (24%)
Day 4	17 (13%)	16 (12%)	19 (14%)	19 (14%)	19 (14%)	19 (14%)	13 (10%)	13 (10%)	13 (10%)	13 (10%)	13 (10%)	13 (10%)	30 (11%)	29 (11%)	32 (12%)	32 (12%)	32 (12%)	32 (12%)
Day 7	18 (13%)	17 (13%)	20 (15%)	20 (15%)	20 (15%)	19 (14%)	11 (8%)	11 (8%)	11 (8%)	11 (8%)	11 (8%)	11 (8%)	29 (11%)	28 (10%)	31 (11%)	31 (11%)	31 (11%)	30 (11%)
Day 14	18 (13%)	17 (13%)	20 (15%)	20 (15%)	20 (15%)	20 (15%)	10 (7%)	10 (7%)	10 (7%)	10 (7%)	10 (7%)	10 (7%)	28 (10%)	27 (10%)	30 (11%)	30 (11%)	30 (11%)	30 (11%)
Day 28	17 (13%)	17 (13%)	19 (14%)	19 (14%)	19 (14%)	18 (13%)	10 (7%)	10 (7%)	10 (7%)	10 (7%)	10 (7%)	11 (8%)	27 (10%)	27 (10%)	29 (11%)	29 (11%)	29 (11%)	29 (11%)

M – mobility, SC – Self-care, UA – Usual activities, PD – Pain/ discomfort, AD – Anxiety/ depression
Items with higher rates of missing data are highlighted in bold

Supplemental Table S2g: Available EQ-5D-Y items for children aged less than 2 years

	**Co-amoxiclav (N=45)**						**Placebo (N=44)**						**Total (N=89)**					
	**M**	**SC**	**UA**	**PD**	**AD**	**VAS**	**M**	**SC**	**UA**	**PD**	**AD**	**VAS**	**M**	**SC**	**UA**	**PD**	**AD**	**VAS**
**Availability of EQ-5D-3L index proxy, completed on behalf of child**																		
Day 0	34 (76%)	**18 (40%)**	40 (89%)	43 (96%)	39 (87%)	39 (87%)	**35 (80%)**	**25 (57%)**	44 (100%)	44 (100%)	44 (100%)	43 (98%)	69 (78%)	**43 (48%)**	84 (94%)	87 (98%)	83 (93%)	82 (92%)
Day 4	20 (44%)	**10 (22%)**	25 (56%)	27 (60%)	27 (60%)	27 (60%)	**19 (43%)**	**13 (30%)**	27 (61%)	29 (66%)	28 (64%)	29 (66%)	39 (44%)	**23 (26%)**	52 (58%)	56 (63%)	55 (62%)	56 (63%)
Day 7	19 (42%)	**9 (20%)**	24 (53%)	25 (56%)	26 (58%)	27 (60%)	**18 (41%)**	**15 (34%)**	26 (59%)	26 (59%)	27 (61%)	28 (64%)	37 (42%)	**24 (27%)**	50 (56%)	51 (57%)	53 (60%)	55 (62%)
Day 14	17 (38%)	**10 (22%)**	22 (49%)	22 (49%)	22 (49%)	22 (49%)	**16 (36%)**	**14 (32%)**	24 (55%)	25 (57%)	25 (57%)	24 (55%)	33 (37%)	**24 (27%)**	46 (52%)	47 (53%)	47 (53%)	46 (52%)
Day 28	15 (33%)	**11 (24%)**	20 (44%)	20 (44%)	20 (44%)	19 (42%)	**14 (32%)**	**12 (27%)**	19 (43%)	19 (43%)	19 (43%)	19 (43%)	29 (33%)	**23 (26%)**	39 (44%)	39 (44%)	39 (44%)	38 (43%)
**Availability of EQ-5D-3L index completed by child**																		
Day 0	0 (0%)	0 (0%)	0 (0%)	0 (0%)	0 (0%)	0 (0%)	0 (0%)	0 (0%)	1 (2%)	1 (2%)	1 (2%)	1 (2%)	0 (0%)	0 (0%)	1 (1%)	1 (1%)	1 (1%)	1 (1%)
Day 4	1 (2%)	0 (0%)	3 (7%)	3 (7%)	3 (7%)	3 (7%)	1 (2%)	1 (2%)	1 (2%)	1 (2%)	1 (2%)	1 (2%)	2 (2%)	1 (1%)	4 (4%)	4 (4%)	4 (4%)	4 (4%)
Day 7	1 (2%)	0 (0%)	3 (7%)	3 (7%)	3 (7%)	3 (7%)	1 (2%)	1 (2%)	1 (2%)	1 (2%)	1 (2%)	1 (2%)	2 (2%)	1 (1%)	4 (4%)	4 (4%)	4 (4%)	4 (4%)
Day 14	1 (2%)	0 (0%)	3 (7%)	3 (7%)	3 (7%)	3 (7%)	1 (2%)	1 (2%)	1 (2%)	1 (2%)	1 (2%)	1 (2%)	2 (2%)	1 (1%)	4 (4%)	4 (4%)	4 (4%)	4 (4%)
Day 28	0 (0%)	0 (0%)	2 (4%)	2 (4%)	2 (4%)	2 (4%)	1 (2%)	1 (2%)	1 (2%)	1 (2%)	1 (2%)	1 (2%)	1 (1%)	1 (1%)	3 (3%)	3 (3%)	3 (3%)	3 (3%)

M – mobility, SC – Self-care, UA – Usual activities, PD – Pain/ discomfort, AD – Anxiety/ depression
Items with higher rates of missing data are highlighted in bold

Rombach I, *et al. BMJ Open* 2022; 12:e049373. doi: 10.1136/bmjopen-2021-049373

Supplemental Table S2h: Available EQ-5D-Y items for children aged 2+ years (proxy)

	**Co-amoxiclav (N=91)**						**Placebo (N=91)**						**Total (N=182)**					
	**M**	**SC**	**UA**	**PD**	**AD**	**VAS**	**M**	**SC**	**UA**	**PD**	**AD**	**VAS**	**M**	**SC**	**UA**	**PD**	**AD**	**VAS**
**Availability of EQ-5D-3L index proxy, completed on behalf of child**																		
Day 0	86 (95%)	**79 (87%)**	88 (97%)	88 (97%)	88 (97%)	81 (89%)	90 (99%)	**85 (93%)**	91 (100%)	91 (100%)	90 (99%)	86 (95%)	176 (97%)	**164 (90%)**	179 (98%)	179 (98%)	178 (98%)	**167 (92%)**
Day 4	49 (54%)	**44 (48%)**	50 (55%)	49 (54%)	49 (54%)	50 (55%)	44 (48%)	**41 (45%)**	48 (53%)	48 (53%)	48 (53%)	47 (52%)	93 (51%)	**85 (47%)**	98 (54%)	97 (53%)	97 (53%)	97 (53%)
Day 7	49 (54%)	**43 (47%)**	50 (55%)	50 (55%)	50 (55%)	51 (56%)	43 (47%)	41 (45%)	46 (51%)	45 (49%)	46 (51%)	45 (49%)	92 (51%)	84 (46%)	96 (53%)	95 (52%)	96 (53%)	96 (53%)
Day 14	36 (40%)	34 (37%)	37 (41%)	37 (41%)	37 (41%)	39 (43%)	34 (37%)	33 (36%)	34 (37%)	34 (37%)	34 (37%)	33 (36%)	70 (38%)	67 (37%)	71 (39%)	71 (39%)	71 (39%)	72 (40%)
Day 28	33 (36%)	32 (35%)	33 (36%)	33 (36%)	31 (34%)	34 (37%)	20 (22%)	20 (22%)	20 (22%)	20 (22%)	20 (22%)	21 (23%)	53 (29%)	52 (29%)	53 (29%)	53 (29%)	51 (28%)	55 (30%)
**Availability of EQ-5D-3L index completed by child**																		
Day 0	32 (35%)	32 (35%)	32 (35%)	32 (35%)	32 (35%)	32 (35%)	30 (33%)	30 (33%)	30 (33%)	30 (33%)	29 (32%)	31 (34%)	62 (34%)	62 (34%)	62 (34%)	62 (34%)	61 (34%)	63 (35%)
Day 4	16 (18%)	16 (18%)	16 (18%)	16 (18%)	16 (18%)	16 (18%)	12 (13%)	12 (13%)	12 (13%)	12 (13%)	12 (13%)	12 (13%)	28 (15%)	28 (15%)	28 (15%)	28 (15%)	28 (15%)	28 (15%)
Day 7	17 (19%)	17 (19%)	17 (19%)	17 (19%)	17 (19%)	16 (18%)	10 (11%)	10 (11%)	10 (11%)	10 (11%)	10 (11%)	10 (11%)	27 (15%)	27 (15%)	27 (15%)	27 (15%)	27 (15%)	26 (14%)
Day 14	17 (19%)	17 (19%)	17 (19%)	17 (19%)	17 (19%)	17 (19%)	9 (10%)	9 (10%)	9 (10%)	9 (10%)	9 (10%)	9 (10%)	26 (14%)	26 (14%)	26 (14%)	26 (14%)	26 (14%)	26 (14%)
Day 28	17 (19%)	17 (19%)	17 (19%)	17 (19%)	17 (19%)	16 (18%)	9 (10%)	9 (10%)	9 (10%)	9 (10%)	9 (10%)	10 (11%)	26 (14%)	26 (14%)	26 (14%)	26 (14%)	26 (14%)	26 (14%)

M – mobility, SC – Self-care, UA – Usual activities, PD – Pain/ discomfort, AD – Anxiety/ depression
Items with higher rates of missing data are highlighted in bold

Rombach I, *et al. BMJ Open* 2022; 12:e049373. doi: 10.1136/bmjopen-2021-049373

Supplemental File 3: Reponses to the EQ-5D-Y items

The following tables present the responses to the EQ-5D-Y items at the relevant time points, both for proxy-completed and self-completed questionnaires. Additional tables also show responses separately for children aged less than 2 years, and 2+ years. The summaries include participants for whom the EQ-5D-Y index is available, i.e. for whom all five responses were available.

Supplemental Table S3a: Responses to EQ-5D-Y domains (proxy, completed on behalf of child)

	M	SC	UA	PD	AD	M	SC	UA	PD	AD	M	SC	UA	PD	AD
**Baseline**	Co-amoxiclav (N = 96)					Placebo (N = 108)					Total (N = 204)				
**No Problems**	78 (81%)	65 (68%)	37 (39%)	22 (23%)	35 (36%)	80 (74%)	67 (62%)	44 (41%)	33 (31%)	49 (45%)	158 (77%)	132 (65%)	81 (40%)	55 (27%)	84 (41%)
**Moderate problems**	11 (11%)	22 (23%)	44 (46%)	67 (70%)	55 (57%)	21 (19%)	22 (20%)	52 (48%)	63 (58%)	51 (47%)	32 (16%)	44 (22%)	96 (47%)	130 (64%)	106 (52%)
**Extreme problems**	7 (7%)	9 (9%)	15 (16%)	7 (7%)	6 (6%)	7 (6%)	19 (18%)	12 (11%)	12 (11%)	8 (7%)	14 (7%)	28 (14%)	27 (13%)	19 (9%)	14 (7%)
**Day 4**	Co-amoxiclav (N = 52)					Placebo (N = 52)					Total (N = 104)				
**No Problems**	38 (73%)	40 (77%)	22 (42%)	18 (35%)	26 (50%)	39 (75%)	34 (65%)	24 (46%)	22 (42%)	32 (62%)	77 (74%)	74 (71%)	46 (44%)	40 (38%)	58 (56%)
**Moderate problems**	10 (19%)	8 (15%)	20 (38%)	31 (60%)	24 (46%)	12 (23%)	14 (27%)	22 (42%)	28 (54%)	19 (37%)	22 (21%)	22 (21%)	42 (40%)	59 (57%)	43 (41%)
**Extreme problems**	4 (8%)	4 (8%)	10 (19%)	3 (6%)	2 (4%)	1 (2%)	4 (8%)	6 (12%)	2 (4%)	1 (2%)	5 (5%)	8 (8%)	16 (15%)	5 (5%)	3 (3%)
**Day 7**	Co-amoxiclav (N = 52)					Placebo (N = 54)					Total (N = 106)				
**No Problems**	44 (85%)	46 (88%)	38 (73%)	32 (62%)	43 (83%)	46 (85%)	41 (76%)	33 (61%)	33 (61%)	41 (76%)	90 (85%)	87 (82%)	71 (67%)	65 (61%)	84 (79%)
**Moderate problems**	3 (6%)	2 (4%)	11 (21%)	17 (33%)	8 (15%)	6 (11%)	8 (15%)	18 (33%)	18 (33%)	12 (22%)	9 (8%)	10 (9%)	29 (27%)	35 (33%)	20 (19%)
**Extreme problems**	5 (10%)	4 (8%)	3 (6%)	3 (6%)	1 (2%)	2 (4%)	5 (9%)	3 (6%)	3 (6%)	1 (2%)	7 (7%)	9 (8%)	6 (6%)	6 (6%)	2 (2%)
**Day 14**	Co-amoxiclav (N = 44)					Placebo (N = 46)					Total (N = 90)				
**No Problems**	41 (93%)	40 (91%)	35 (80%)	35 (80%)	38 (86%)	43 (93%)	40 (87%)	40 (87%)	39 (85%)	40 (87%)	84 (93%)	80 (89%)	75 (83%)	74 (82%)	78 (87%)
**Moderate problems**	0 (0%)	2 (5%)	7 (16%)	6 (14%)	5 (11%)	3 (7%)	5 (11%)	6 (13%)	7 (15%)	6 (13%)	3 (3%)	7 (8%)	13 (14%)	13 (14%)	11 (12%)
**Extreme problems**	3 (7%)	2 (5%)	2 (5%)	3 (7%)	1 (2%)	0 (0%)	1 (2%)	0 (0%)	0 (0%)	0 (0%)	3 (3%)	3 (3%)	2 (2%)	3 (3%)	1 (1%)
**Day 28**	Co-amoxiclav (N = 41)					Placebo (N = 31)					Total (N = 72)				
**No Problems**	39 (95%)	39 (95%)	37 (90%)	35 (85%)	36 (88%)	28 (90%)	28 (90%)	28 (90%)	26 (84%)	28 (90%)	67 (93%)	67 (93%)	65 (90%)	61 (85%)	64 (89%)
**Moderate problems**	0 (0%)	0 (0%)	4 (10%)	5 (12%)	5 (12%)	3 (10%)	2 (6%)	3 (10%)	5 (16%)	3 (10%)	3 (4%)	2 (3%)	7 (10%)	10 (14%)	8 (11%)
**Extreme problems**	2 (5%)	2 (5%)	0 (0%)	1 (2%)	0 (0%)	0 (0%)	1 (3%)	0 (0%)	0 (0%)	0 (0%)	2 (3%)	3 (4%)	0 (0%)	1 (1%)	0 (0%)

M – mobility, SC – Self-care, UA – Usual activities, PD – Pain/ discomfort, AD – Anxiety/ depression

Rombach I, *et al. BMJ Open* 2022; 12:e049373. doi: 10.1136/bmjopen-2021-049373

Supplemental Table S3b: Responses to EQ-5D-Y domains (proxy, completed on behalf of child for children aged less than 2 years)

	**M**	**SC**	**UA**	**PD**	**AD**	**M**	**SC**	**UA**	**PD**	**AD**	**M**	**SC**	**UA**	**PD**	**AD**
**Baseline**	Co-amoxiclav (N = 18)					Placebo (N = 24)					Total (N = 42)				
**No Problems**	14 (78%)	15 (83%)	11 (61%)	4 (22%)	6 (33%)	19 (79%)	13 (54%)	13 (54%)	8 (33%)	7 (29%)	33 (79%)	28 (67%)	24 (57%)	12 (29%)	13 (31%)
**Moderate problems**	2 (11%)	0 (0%)	5 (28%)	14 (78%)	11 (61%)	4 (17%)	5 (21%)	11 (46%)	16 (67%)	15 (63%)	6 (14%)	5 (12%)	16 (38%)	30 (71%)	26 (62%)
**Extreme problems**	2 (11%)	3 (17%)	2 (11%)	0 (0%)	1 (6%)	1 (4%)	6 (25%)	0 (0%)	0 (0%)	2 (8%)	3 (7%)	9 (21%)	2 (5%)	0 (0%)	3 (7%)
**Day 4**	Co-amoxiclav (N = 9)					Placebo (N = 12)					Total (N = 21)				
**No Problems**	7 (78%)	7 (78%)	6 (67%)	4 (44%)	6 (67%)	10 (83%)	9 (75%)	7 (58%)	4 (33%)	6 (50%)	17 (81%)	16 (76%)	13 (62%)	8 (38%)	12 (57%)
**Moderate problems**	1 (11%)	1 (11%)	2 (22%)	5 (56%)	3 (33%)	2 (17%)	2 (17%)	5 (42%)	8 (67%)	6 (50%)	3 (14%)	3 (14%)	7 (33%)	13 (62%)	9 (43%)
**Extreme problems**	1 (11%)	1 (11%)	1 (11%)	0 (0%)	0 (0%)	0 (0%)	1 (8%)	0 (0%)	0 (0%)	0 (0%)	1 (5%)	2 (10%)	1 (5%)	0 (0%)	0 (0%)
**Day 7**	Co-amoxiclav (N = 9)					Placebo (N = 14)					Total (N = 23)				
**No Problems**	8 (89%)	8 (89%)	7 (78%)	7 (78%)	9 (100%)	11 (79%)	10 (71%)	9 (64%)	8 (57%)	10 (71%)	19 (83%)	18 (78%)	16 (70%)	15 (65%)	19 (83%)
**Moderate problems**	0 (0%)	0 (0%)	1 (11%)	2 (22%)	0 (0%)	3 (21%)	3 (21%)	5 (36%)	6 (43%)	4 (29%)	3 (13%)	3 (13%)	6 (26%)	8 (35%)	4 (17%)
**Extreme problems**	1 (11%)	1 (11%)	1 (11%)	0 (0%)	0 (0%)	0 (0%)	1 (7%)	0 (0%)	0 (0%)	0 (0%)	1 (4%)	2 (9%)	1 (4%)	0 (0%)	0 (0%)
**Day 14**	Co-amoxiclav (N = 10)					Placebo (N = 13)					Total (N = 23)				
**No Problems**	10 (100%)	9 (90%)	8 (80%)	7 (70%)	8 (80%)	11 (85%)	10 (77%)	10 (77%)	10 (77%)	10 (77%)	21 (91%)	19 (83%)	18 (78%)	17 (74%)	18 (78%)
**Moderate problems**	0 (0%)	1 (10%)	2 (20%)	2 (20%)	2 (20%)	2 (15%)	3 (23%)	3 (23%)	3 (23%)	3 (23%)	2 (9%)	4 (17%)	5 (22%)	5 (22%)	5 (22%)
**Extreme problems**	0 (0%)	0 (0%)	0 (0%)	1 (10%)	0 (0%)	0 (0%)	0 (0%)	0 (0%)	0 (0%)	0 (0%)	0 (0%)	0 (0%)	0 (0%)	1 (4%)	0 (0%)
**Day 28**	Co-amoxiclav (N = 11)					Placebo (N = 11)					Total (N = 22)				
**No Problems**	11 (100%)	11 (100%)	11 (100%)	10 (91%)	11 (100%)	10 (91%)	10 (91%)	10 (91%)	8 (73%)	9 (82%)	21 (95%)	21 (95%)	21 (95%)	18 (82%)	20 (91%)
**Moderate problems**	0 (0%)	0 (0%)	0 (0%)	1 (9%)	0 (0%)	1 (9%)	1 (9%)	1 (9%)	3 (27%)	2 (18%)	1 (5%)	1 (5%)	1 (5%)	4 (18%)	2 (9%)
**Extreme problems**	0 (0%)	0 (0%)	0 (0%)	0 (0%)	0 (0%)	0 (0%)	0 (0%)	0 (0%)	0 (0%)	0 (0%)	0 (0%)	0 (0%)	0 (0%)	0 (0%)	0 (0%)

M – mobility, SC – Self-care, UA – Usual activities, PD – Pain/ discomfort, AD – Anxiety/ depression

Rombach I, *et al. BMJ Open* 2022; 12:e049373. doi: 10.1136/bmjopen-2021-049373

Supplemental Table S3c: Responses to EQ-5D-Y domains (proxy, completed on behalf of child for children aged 2+ years)

	**M**	**SC**	**UA**	**PD**	**AD**	**M**	**SC**	**UA**	**PD**	**AD**	**M**	**SC**	**UA**	**PD**	**AD**
**Baseline**	Co-amoxiclav (N = 78)					Placebo (N = 84)					Total (N = 162)				
**No Problems**	64 (82%)	50 (64%)	26 (33%)	18 (23%)	29 (37%)	61 (73%)	54 (64%)	31 (37%)	25 (30%)	42 (50%)	125 (77%)	104 (64%)	57 (35%)	43 (27%)	71 (44%)
**Moderate problems**	9 (12%)	22 (28%)	39 (50%)	53 (68%)	44 (56%)	17 (20%)	17 (20%)	41 (49%)	47 (56%)	36 (43%)	26 (16%)	39 (24%)	80 (49%)	100 (62%)	80 (49%)
**Extreme problems**	5 (6%)	6 (8%)	13 (17%)	7 (9%)	5 (6%)	6 (7%)	13 (15%)	12 (14%)	12 (14%)	6 (7%)	11 (7%)	19 (12%)	25 (15%)	19 (12%)	11 (7%)
**Day 4**	Co-amoxiclav (N = 43)					Placebo (N = 40)					Total (N = 83)				
**No Problems**	31 (72%)	33 (77%)	16 (37%)	14 (33%)	20 (47%)	29 (73%)	25 (63%)	17 (43%)	18 (45%)	26 (65%)	60 (72%)	58 (70%)	33 (40%)	32 (39%)	46 (55%)
**Moderate problems**	9 (21%)	7 (16%)	18 (42%)	26 (60%)	21 (49%)	10 (25%)	12 (30%)	17 (43%)	20 (50%)	13 (33%)	19 (23%)	19 (23%)	35 (42%)	46 (55%)	34 (41%)
**Extreme problems**	3 (7%)	3 (7%)	9 (21%)	3 (7%)	2 (5%)	1 (3%)	3 (8%)	6 (15%)	2 (5%)	1 (3%)	4 (5%)	6 (7%)	15 (18%)	5 (6%)	3 (4%)
**Day 7**	Co-amoxiclav (N = 43)					Placebo (N = 40)					Total (N = 83)				
**No Problems**	36 (84%)	38 (88%)	31 (72%)	25 (58%)	34 (79%)	35 (88%)	31 (78%)	24 (60%)	25 (63%)	31 (78%)	71 (86%)	69 (83%)	55 (66%)	50 (60%)	65 (78%)
**Moderate problems**	3 (7%)	2 (5%)	10 (23%)	15 (35%)	8 (19%)	3 (8%)	5 (13%)	13 (33%)	12 (30%)	8 (20%)	6 (7%)	7 (8%)	23 (28%)	27 (33%)	16 (19%)
**Extreme problems**	4 (9%)	3 (7%)	2 (5%)	3 (7%)	1 (2%)	2 (5%)	4 (10%)	3 (8%)	3 (8%)	1 (3%)	6 (7%)	7 (8%)	5 (6%)	6 (7%)	2 (2%)
**Day 14**	Co-amoxiclav (N = 34)					Placebo (N = 33)					Total (N = 67)				
**No Problems**	31 (91%)	31 (91%)	27 (79%)	28 (82%)	30 (88%)	32 (97%)	30 (91%)	30 (91%)	29 (88%)	30 (91%)	63 (94%)	61 (91%)	57 (85%)	57 (85%)	60 (90%)
**Moderate problems**	0 (0%)	1 (3%)	5 (15%)	4 (12%)	3 (9%)	1 (3%)	2 (6%)	3 (9%)	4 (12%)	3 (9%)	1 (1%)	3 (4%)	8 (12%)	8 (12%)	6 (9%)
**Extreme problems**	3 (9%)	2 (6%)	2 (6%)	2 (6%)	1 (3%)	0 (0%)	1 (3%)	0 (0%)	0 (0%)	0 (0%)	3 (4%)	3 (4%)	2 (3%)	2 (3%)	1 (1%)
**Day 28**	Co-amoxiclav (N = 30)					Placebo (N = 20)					Total (N = 50)				
**No Problems**	28 (93%)	28 (93%)	26 (87%)	25 (83%)	25 (83%)	18 (90%)	18 (90%)	18 (90%)	18 (90%)	19 (95%)	46 (92%)	46 (92%)	44 (88%)	43 (86%)	44 (88%)
**Moderate problems**	0 (0%)	0 (0%)	4 (13%)	4 (13%)	5 (17%)	2 (10%)	1 (5%)	2 (10%)	2 (10%)	1 (5%)	2 (4%)	1 (2%)	6 (12%)	6 (12%)	6 (12%)
**Extreme problems**	2 (7%)	2 (7%)	0 (0%)	1 (3%)	0 (0%)	0 (0%)	1 (5%)	0 (0%)	0 (0%)	0 (0%)	2 (4%)	3 (6%)	0 (0%)	1 (2%)	0 (0%)

M – mobility, SC – Self-care, UA – Usual activities, PD – Pain/ discomfort, AD – Anxiety/ depression

Rombach I, *et al. BMJ Open* 2022; 12:e049373. doi: 10.1136/bmjopen-2021-049373

Supplemental Table S3d: Responses to EQ-5D-Y domains (completed by child)

	M	SC	UA	PD	AD	M	SC	UA	PD	AD	M	SC	UA	PD	AD
**Baseline**	Co-amoxiclav (N = 32)					Placebo (N = 29)					Total (N = 61)				
**No Problems**	20 (63%)	24 (75%)	4 (13%)	6 (19%)	17 (53%)	24 (83%)	23 (79%)	9 (31%)	5 (17%)	13 (45%)	44 (72%)	47 (77%)	13 (21%)	11 (18%)	30 (49%)
**Moderate problems**	10 (31%)	6 (19%)	22 (69%)	16 (50%)	14 (44%)	5 (17%)	5 (17%)	15 (52%)	16 (55%)	14 (48%)	15 (25%)	11 (18%)	37 (61%)	32 (52%)	28 (46%)
**Extreme problems**	2 (6%)	2 (6%)	6 (19%)	10 (31%)	1 (3%)	0 (0%)	1 (3%)	5 (17%)	8 (28%)	2 (7%)	2 (3%)	3 (5%)	11 (18%)	18 (30%)	3 (5%)
**Day 4**	Co-amoxiclav (N = 16)					Placebo (N = 13)					Total (N = 29)				
**No Problems**	13 (81%)	13 (81%)	7 (44%)	5 (31%)	7 (44%)	10 (77%)	10 (77%)	5 (38%)	4 (31%)	7 (54%)	23 (79%)	23 (79%)	12 (41%)	9 (31%)	14 (48%)
**Moderate problems**	2 (13%)	2 (13%)	5 (31%)	10 (63%)	8 (50%)	3 (23%)	3 (23%)	8 (62%)	8 (62%)	6 (46%)	5 (17%)	5 (17%)	13 (45%)	18 (62%)	14 (48%)
**Extreme problems**	1 (6%)	1 (6%)	4 (25%)	1 (6%)	1 (6%)	0 (0%)	0 (0%)	0 (0%)	1 (8%)	0 (0%)	1 (3%)	1 (3%)	4 (14%)	2 (7%)	1 (3%)
**Day 7**	Co-amoxiclav (N = 17)					Placebo (N = 11)					Total (N = 28)				
**No Problems**	14 (82%)	16 (94%)	10 (59%)	12 (71%)	14 (82%)	10 (91%)	8 (73%)	6 (55%)	5 (45%)	7 (64%)	24 (86%)	24 (86%)	16 (57%)	17 (61%)	21 (75%)
**Moderate problems**	2 (12%)	0 (0%)	6 (35%)	4 (24%)	2 (12%)	1 (9%)	3 (27%)	5 (45%)	6 (55%)	4 (36%)	3 (11%)	3 (11%)	11 (39%)	10 (36%)	6 (21%)
**Extreme problems**	1 (6%)	1 (6%)	1 (6%)	1 (6%)	1 (6%)	0 (0%)	0 (0%)	0 (0%)	0 (0%)	0 (0%)	1 (4%)	1 (4%)	1 (4%)	1 (4%)	1 (4%)
**Day 14**	Co-amoxiclav (N = 17)					Placebo (N = 10)					Total (N = 27)				
**No Problems**	16 (94%)	16 (94%)	14 (82%)	16 (94%)	17 (100%)	10 (100%)	9 (90%)	9 (90%)	7 (70%)	9 (90%)	26 (96%)	25 (93%)	23 (85%)	23 (85%)	26 (96%)
**Moderate problems**	1 (6%)	1 (6%)	3 (18%)	1 (6%)	0 (0%)	0 (0%)	1 (10%)	1 (10%)	3 (30%)	1 (10%)	1 (4%)	2 (7%)	4 (15%)	4 (15%)	1 (4%)
**Extreme problems**	0 (0%)	0 (0%)	0 (0%)	0 (0%)	0 (0%)	0 (0%)	0 (0%)	0 (0%)	0 (0%)	0 (0%)	0 (0%)	0 (0%)	0 (0%)	0 (0%)	0 (0%)
**Day 28**	Co-amoxiclav (N = 17)					Placebo (N = 10)					Total (N = 27)				
**No Problems**	17 (100%)	17 (100%)	15 (88%)	17 (100%)	17 (100%)	10 (100%)	10 (100%)	9 (90%)	8 (80%)	8 (80%)	27 (100%)	27 (100%)	24 (89%)	25 (93%)	25 (93%)
**Moderate problems**	0 (0%)	0 (0%)	2 (12%)	0 (0%)	0 (0%)	0 (0%)	0 (0%)	1 (10%)	2 (20%)	2 (20%)	0 (0%)	0 (0%)	3 (11%)	2 (7%)	2 (7%)
**Extreme problems**	0 (0%)	0 (0%)	0 (0%)	0 (0%)	0 (0%)	0 (0%)	0 (0%)	0 (0%)	0 (0%)	0 (0%)	0 (0%)	0 (0%)	0 (0%)	0 (0%)	0 (0%)

M – mobility, SC – Self-care, UA – Usual activities, PD – Pain/ discomfort, AD – Anxiety/ depression

Supplemental Table S3e: Responses to EQ-5D-Y domains (completed by child for children aged less than 2 years)

	M	SC	UA	PD	AD	M	SC	UA	PD	AD	M	SC	UA	PD	AD
**Baseline**	Co-amoxiclav (N = 0)					Placebo (N = 0)					Total (N = 0)				
**No Problems**	0 (.%)	0 (.%)	0 (.%)	0 (.%)	0 (.%)	0 (.%)	0 (.%)	0 (.%)	0 (.%)	0 (.%)	0 (.%)	0 (.%)	0 (.%)	0 (.%)	0 (.%)
**Moderate problems**	0 (.%)	0 (.%)	0 (.%)	0 (.%)	0 (.%)	0 (.%)	0 (.%)	0 (.%)	0 (.%)	0 (.%)	0 (.%)	0 (.%)	0 (.%)	0 (.%)	0 (.%)
**Extreme problems**	0 (.%)	0 (.%)	0 (.%)	0 (.%)	0 (.%)	0 (.%)	0 (.%)	0 (.%)	0 (.%)	0 (.%)	0 (.%)	0 (.%)	0 (.%)	0 (.%)	0 (.%)
**Day 4**	Co-amoxiclav (N = 0)					Placebo (N = 1)					Total (N = 1)				
**No Problems**	0 (.%)	0 (.%)	0 (.%)	0 (.%)	0 (.%)	1 (100%)	0 (0%)	1 (100%)	0 (0%)	0 (0%)	1 (100%)	0 (0%)	1 (100%)	0 (0%)	0 (0%)
**Moderate problems**	0 (.%)	0 (.%)	0 (.%)	0 (.%)	0 (.%)	0 (0%)	1 (100%)	0 (0%)	1 (100%)	1 (100%)	0 (0%)	1 (100%)	0 (0%)	1 (100%)	1 (100%)
**Extreme problems**	0 (.%)	0 (.%)	0 (.%)	0 (.%)	0 (.%)	0 (0%)	0 (0%)	0 (0%)	0 (0%)	0 (0%)	0 (0%)	0 (0%)	0 (0%)	0 (0%)	0 (0%)
**Day 7**	Co-amoxiclav (N = 0)					Placebo (N = 1)					Total (N = 1)				
**No Problems**	0 (.%)	0 (.%)	0 (.%)	0 (.%)	0 (.%)	1 (100%)	0 (0%)	0 (0%)	0 (0%)	0 (0%)	1 (100%)	0 (0%)	0 (0%)	0 (0%)	0 (0%)
**Moderate problems**	0 (.%)	0 (.%)	0 (.%)	0 (.%)	0 (.%)	0 (0%)	1 (100%)	1 (100%)	1 (100%)	1 (100%)	0 (0%)	1 (100%)	1 (100%)	1 (100%)	1 (100%)
**Extreme problems**	0 (.%)	0 (.%)	0 (.%)	0 (.%)	0 (.%)	0 (0%)	0 (0%)	0 (0%)	0 (0%)	0 (0%)	0 (0%)	0 (0%)	0 (0%)	0 (0%)	0 (0%)
**Day 14**	Co-amoxiclav (N = 0)					Placebo (N = 1)					Total (N = 1)				
**No Problems**	0 (.%)	0 (.%)	0 (.%)	0 (.%)	0 (.%)	1 (100%)	0 (0%)	0 (0%)	0 (0%)	0 (0%)	1 (100%)	0 (0%)	0 (0%)	0 (0%)	0 (0%)
**Moderate problems**	0 (.%)	0 (.%)	0 (.%)	0 (.%)	0 (.%)	0 (0%)	1 (100%)	1 (100%)	1 (100%)	1 (100%)	0 (0%)	1 (100%)	1 (100%)	1 (100%)	1 (100%)
**Extreme problems**	0 (.%)	0 (.%)	0 (.%)	0 (.%)	0 (.%)	0 (0%)	0 (0%)	0 (0%)	0 (0%)	0 (0%)	0 (0%)	0 (0%)	0 (0%)	0 (0%)	0 (0%)
**Day 28**	Co-amoxiclav (N = 0)					Placebo (N = 1)					Total (N = 1)				
**No Problems**	0 (.%)	0 (.%)	0 (.%)	0 (.%)	0 (.%)	1 (100%)	1 (100%)	1 (100%)	0 (0%)	0 (0%)	1 (100%)	1 (100%)	1 (100%)	0 (0%)	0 (0%)
**Moderate problems**	0 (.%)	0 (.%)	0 (.%)	0 (.%)	0 (.%)	0 (0%)	0 (0%)	0 (0%)	1 (100%)	1 (100%)	0 (0%)	0 (0%)	0 (0%)	1 (100%)	1 (100%)
**Extreme problems**	0 (.%)	0 (.%)	0 (.%)	0 (.%)	0 (.%)	0 (0%)	0 (0%)	0 (0%)	0 (0%)	0 (0%)	0 (0%)	0 (0%)	0 (0%)	0 (0%)	0 (0%)

M – mobility, SC – Self-care, UA – Usual activities, PD – Pain/ discomfort, AD – Anxiety/ depression

Rombach I, *et al. BMJ Open* 2022; 12:e049373. doi: 10.1136/bmjopen-2021-049373

Supplemental Table S3f: Responses to EQ-5D-Y domains (completed by child for children aged 2+ years)

	M	SC	UA	PD	AD	M	SC	UA	PD	AD	M	SC	UA	PD	AD
**Baseline**	Co-amoxiclav (N = 32)					Placebo (N = 29)					Total (N = 61)				
**No Problems**	20 (63%)	24 (75%)	4 (13%)	6 (19%)	10 (31%)	24 (83%)	23 (79%)	9 (31%)	5 (17%)	11 (38%)	44 (72%)	47 (77%)	13 (21%)	11 (18%)	21 (34%)
**Moderate problems**	10 (31%)	6 (19%)	22 (69%)	16 (50%)	20 (63%)	5 (17%)	5 (17%)	15 (52%)	16 (55%)	15 (52%)	15 (25%)	11 (18%)	37 (61%)	32 (52%)	35 (57%)
**Extreme problems**	2 (6%)	2 (6%)	6 (19%)	10 (31%)	2 (6%)	0 (0%)	1 (3%)	5 (17%)	8 (28%)	3 (10%)	2 (3%)	3 (5%)	11 (18%)	18 (30%)	5 (8%)
**Day 4**	Co-amoxiclav (N = 16)					Placebo (N = 12)					Total (N = 28)				
**No Problems**	13 (81%)	13 (81%)	7 (44%)	5 (31%)	7 (47%)	9 (75%)	10 (83%)	4 (33%)	4 (33%)	7 (64%)	22 (79%)	23 (82%)	11 (39%)	9 (32%)	14 (54%)
**Moderate problems**	2 (13%)	2 (13%)	5 (31%)	10 (63%)	7 (47%)	3 (25%)	2 (17%)	8 (67%)	7 (58%)	4 (36%)	5 (18%)	4 (14%)	13 (46%)	17 (61%)	11 (42%)
**Extreme problems**	1 (6%)	1 (6%)	4 (25%)	1 (6%)	1 (7%)	0 (0%)	0 (0%)	0 (0%)	1 (8%)	0 (0%)	1 (4%)	1 (4%)	4 (14%)	2 (7%)	1 (4%)
**Day 7**	Co-amoxiclav (N = 17)					Placebo (N = 10)					Total (N = 27)				
**No Problems**	14 (82%)	16 (94%)	10 (59%)	12 (71%)	13 (81%)	9 (90%)	8 (80%)	6 (60%)	5 (50%)	7 (78%)	23 (85%)	24 (89%)	16 (59%)	17 (63%)	20 (80%)
**Moderate problems**	2 (12%)	0 (0%)	6 (35%)	4 (24%)	3 (19%)	1 (10%)	2 (20%)	4 (40%)	5 (50%)	2 (22%)	3 (11%)	2 (7%)	10 (37%)	9 (33%)	5 (20%)
**Extreme problems**	1 (6%)	1 (6%)	1 (6%)	1 (6%)	0 (0%)	0 (0%)	0 (0%)	0 (0%)	0 (0%)	0 (0%)	1 (4%)	1 (4%)	1 (4%)	1 (4%)	0 (0%)
**Day 14**	Co-amoxiclav (N = 17)					Placebo (N = 9)					Total (N = 26)				
**No Problems**	16 (94%)	16 (94%)	14 (82%)	16 (94%)	14 (93%)	9 (100%)	9 (100%)	9 (100%)	7 (78%)	8 (100%)	25 (96%)	25 (96%)	23 (88%)	23 (88%)	22 (96%)
**Moderate problems**	1 (6%)	1 (6%)	3 (18%)	1 (6%)	1 (7%)	0 (0%)	0 (0%)	0 (0%)	2 (22%)	0 (0%)	1 (4%)	1 (4%)	3 (12%)	3 (12%)	1 (4%)
**Extreme problems**	0 (0%)	0 (0%)	0 (0%)	0 (0%)	0 (0%)	0 (0%)	0 (0%)	0 (0%)	0 (0%)	0 (0%)	0 (0%)	0 (0%)	0 (0%)	0 (0%)	0 (0%)
**Day 28**	Co-amoxiclav (N = 17)					Placebo (N = 9)					Total (N = 26)				
**No Problems**	17 (100%)	17 (100%)	15 (88%)	17 (100%)	15 (94%)	9 (100%)	9 (100%)	8 (89%)	8 (89%)	7 (88%)	26 (100%)	26 (100%)	23 (88%)	25 (96%)	22 (92%)
**Moderate problems**	0 (0%)	0 (0%)	2 (12%)	0 (0%)	1 (6%)	0 (0%)	0 (0%)	1 (11%)	1 (11%)	1 (13%)	0 (0%)	0 (0%)	3 (12%)	1 (4%)	2 (8%)
**Extreme problems**	0 (0%)	0 (0%)	0 (0%)	0 (0%)	0 (0%)	0 (0%)	0 (0%)	0 (0%)	0 (0%)	0 (0%)	0 (0%)	0 (0%)	0 (0%)	0 (0%)	0 (0%)

M – mobility, SC – Self-care, UA – Usual activities, PD – Pain/ discomfort, AD – Anxiety/ depression

Supplemental Table 3g: Responses to CARIFS domains (only including questionnaires with sufficient data to calculate the overall CARIFS score)

		Co-amoxiclav Baseline (N = 99) Day 7 (N = 66)					Placebo Baseline (N = 97) Day 7 (N = 57)				
		No Problem	Minor Problem	Moderate Problem	Major Problem	Missing	No Problem	Minor Problem	Moderate Problem	Major Problem	Missing
**Poor appetite**	**Baseline**	21 (21%)	36 (36%)	32 (32%)	10 (10%)	0 (0%)	16 (16%)	33 (34%)	39 (40%)	9 (9%)	0 (0%)
	**Day 7**	38 (58%)	21 (32%)	6 (9%)	1 (2%)	0 (0%)	23 (40%)	23 (40%)	7 (12%)	4 (7%)	0 (0%)
**Not sleeping well**	**Baseline**	13 (13%)	30 (30%)	40 (40%)	16 (16%)	0 (0%)	16 (16%)	24 (25%)	38 (39%)	19 (20%)	0 (0%)
	**Day 7**	44 (67%)	12 (18%)	8 (12%)	2 (3%)	0 (0%)	23 (40%)	19 (33%)	8 (14%)	7 (12%)	0 (0%)
**Irritable, cranky, fussy**	**Baseline**	18 (18%)	32 (32%)	40 (40%)	9 (9%)	0 (0%)	15 (15%)	24 (25%)	45 (46%)	13 (13%)	0 (0%)
	**Day 7**	30 (45%)	26 (39%)	7 (11%)	3 (5%)	0 (0%)	23 (40%)	18 (32%)	11 (19%)	5 (9%)	0 (0%)
**Feels unwell**	**Baseline**	4 (4%)	29 (29%)	46 (46%)	20 (20%)	0 (0%)	9 (9%)	22 (23%)	48 (49%)	18 (19%)	0 (0%)
	**Day 7**	39 (59%)	18 (27%)	7 (11%)	2 (3%)	0 (0%)	28 (49%)	16 (28%)	10 (18%)	3 (5%)	0 (0%)
**Low energy tired**	**Baseline**	14 (14%)	30 (30%)	41 (41%)	14 (14%)	0 (0%)	13 (13%)	31 (32%)	41 (42%)	12 (12%)	0 (0%)
	**Day 7**	38 (58%)	20 (30%)	7 (11%)	1 (2%)	0 (0%)	28 (49%)	16 (28%)	10 (18%)	3 (5%)	0 (0%)
**Not playing well**	**Baseline**	27 (27%)	36 (36%)	29 (29%)	7 (7%)	0 (0%)	34 (35%)	28 (29%)	25 (26%)	10 (10%)	0 (0%)
	**Day 7**	50 (76%)	13 (20%)	2 (3%)	1 (2%)	0 (0%)	38 (67%)	9 (16%)	9 (16%)	1 (2%)	0 (0%)
**Crying more than usual**	**Baseline**	37 (37%)	28 (28%)	25 (25%)	9 (9%)	0 (0%)	32 (33%)	26 (27%)	27 (28%)	12 (12%)	0 (0%)
	**Day 7**	40 (61%)	17 (26%)	6 (9%)	3 (5%)	0 (0%)	33 (58%)	16 (28%)	6 (11%)	2 (4%)	0 (0%)
**Needing extra care**	**Baseline**	23 (23%)	34 (34%)	36 (36%)	6 (6%)	0 (0%)	20 (21%)	33 (34%)	36 (37%)	8 (8%)	0 (0%)
	**Day 7**	43 (65%)	19 (29%)	2 (3%)	2 (3%)	0 (0%)	32 (56%)	17 (30%)	6 (11%)	2 (4%)	0 (0%)

		**Co-amoxiclav Baseline (N = 99) Day 7 (N = 66)**					**Placebo Baseline (N = 97) Day 7 (N = 57)**				
		**No Problem**	**Minor Problem**	**Moderate Problem**	**Major Problem**	**Missing**	**No Problem**	**Minor Problem**	**Moderate Problem**	**Major Problem**	**Missing**
**Clinginess**	**Baseline**	16 (16%)	32 (32%)	38 (38%)	13 (13%)	0 (0%)	23 (24%)	25 (26%)	34 (35%)	15 (15%)	0 (0%)
	**Day 7**	40 (61%)	17 (26%)	7 (11%)	2 (3%)	0 (0%)	29 (51%)	16 (28%)	9 (16%)	3 (5%)	0 (0%)
**Headache**	**Baseline**	38 (38%)	20 (20%)	24 (24%)	1 (1%)	16 (16%)	44 (45%)	17 (18%)	10 (10%)	8 (8%)	18 (19%)
	**Day 7**	47 (71%)	7 (11%)	2 (3%)	1 (2%)	9 (14%)	43 (75%)	4 (7%)	3 (5%)	2 (4%)	5 (9%)
**Sore throat**	**Baseline**	29 (29%)	21 (21%)	31 (31%)	8 (8%)	10 (10%)	32 (33%)	21 (22%)	16 (16%)	15 (15%)	13 (13%)
	**Day 7**	47 (71%)	8 (12%)	3 (5%)	1 (2%)	7 (11%)	33 (58%)	12 (21%)	4 (7%)	1 (2%)	7 (12%)
**Muscle aches and pains**	**Baseline**	41 (41%)	18 (18%)	21 (21%)	5 (5%)	14 (14%)	38 (39%)	19 (20%)	11 (11%)	11 (11%)	18 (19%)
	**Day 7**	44 (67%)	10 (15%)	0 (0%)	2 (3%)	10 (15%)	38 (67%)	8 (14%)	4 (7%)	1 (2%)	6 (11%)
**Fever**	**Baseline**	15 (15%)	35 (35%)	35 (35%)	14 (14%)	0 (0%)	16 (16%)	30 (31%)	34 (35%)	17 (18%)	0 (0%)
	**Day 7**	61 (92%)	4 (6%)	1 (2%)	0 (0%)	0 (0%)	48 (84%)	4 (7%)	3 (5%)	2 (4%)	0 (0%)
**Cough**	**Baseline**	4 (4%)	18 (18%)	52 (53%)	25 (25%)	0 (0%)	3 (3%)	19 (20%)	38 (39%)	37 (38%)	0 (0%)
	**Day 7**	21 (32%)	29 (44%)	11 (17%)	5 (8%)	0 (0%)	13 (23%)	18 (32%)	15 (26%)	11 (19%)	0 (0%)
**Nasal congestion, runny nose**	**Baseline**	5 (5%)	22 (22%)	50 (51%)	22 (22%)	0 (0%)	7 (7%)	24 (25%)	41 (42%)	25 (26%)	0 (0%)
	**Day 7**	21 (32%)	33 (50%)	11 (17%)	1 (2%)	0 (0%)	16 (28%)	19 (33%)	16 (28%)	6 (11%)	0 (0%)
**Vomiting**	**Baseline**	73 (74%)	21 (21%)	3 (3%)	2 (2%)	0 (0%)	80 (82%)	9 (9%)	5 (5%)	3 (3%)	0 (0%)
	**Day 7**	62 (94%)	3 (5%)	0 (0%)	1 (2%)	0 (0%)	51 (89%)	6 (11%)	0 (0%)	0 (0%)	0 (0%)
**Not interested in what's going on**	**Baseline**	60 (61%)	26 (26%)	12 (12%)	1 (1%)	0 (0%)	55 (57%)	26 (27%)	11 (11%)	5 (5%)	0 (0%)
	**Day 7**	58 (88%)	7 (11%)	0 (0%)	1 (2%)	0 (0%)	47 (82%)	6 (11%)	3 (5%)	1 (2%)	0 (0%)

		Co-amoxiclav Baseline (N = 99) Day 7 (N = 66)					Placebo Baseline (N = 97) Day 7 (N = 57)				
		**No Problem**	**Minor Problem**	**Moderate Problem**	**Major Problem**	**Missing**	**No Problem**	**Minor Problem**	**Moderate Problem**	**Major Problem**	**Missing**
**Unable to get out of bed**	**Baseline**	79 (80%)	16 (16%)	4 (4%)	0 (0%)	0 (0%)	74 (76%)	14 (14%)	8 (8%)	1 (1%)	0 (0%)
	**Day 7**	62 (94%)	3 (5%)	1 (2%)	0 (0%)	0 (0%)	52 (91%)	3 (5%)	2 (4%)	0 (0%)	0 (0%)

Note: The scoring algorithm used allowed for the total score to be calculated if there was missing data in items 10-12, but not in any other items.

Rombach I, *et al. BMJ Open* 2022; 12:e049373. doi: 10.1136/bmjopen-2021-049373

Supplemental Table 3h: Responses to CARIFS domains for children less than 2 years old (only including questionnaires with sufficient data to calculate the overall CARIFS score)

		**Co-amoxiclav** Baseline (N = 27) Day 7 (N = 21)					**Placebo** Baseline (N = 29) Day 7 (N = 23)				
		**No Problem**	**Minor Problem**	**Moderate Problem**	**Major Problem**	**Missing**	**No Problem**	**Minor Problem**	**Moderate Problem**	**Major Problem**	**Missing**
**Poor appetite**	**Baseline**	7 (26%)	10 (37%)	7 (26%)	3 (11%)	0 (0%)	4 (14%)	13 (45%)	9 (31%)	3 (10%)	0 (0%)
	**Day 7**	14 (67%)	4 (19%)	3 (14%)	0 (0%)	0 (0%)	11 (48%)	7 (30%)	3 (13%)	2 (9%)	0 (0%)
**Not sleeping well**	**Baseline**	3 (11%)	12 (44%)	7 (26%)	5 (19%)	0 (0%)	4 (14%)	7 (24%)	12 (41%)	6 (21%)	0 (0%)
	**Day 7**	13 (62%)	5 (24%)	3 (14%)	0 (0%)	0 (0%)	10 (43%)	7 (30%)	3 (13%)	3 (13%)	0 (0%)
**Irritable, cranky, fussy**	**Baseline**	5 (19%)	10 (37%)	10 (37%)	2 (7%)	0 (0%)	2 (7%)	5 (17%)	17 (59%)	5 (17%)	0 (0%)
	**Day 7**	12 (57%)	6 (29%)	3 (14%)	0 (0%)	0 (0%)	8 (35%)	7 (30%)	7 (30%)	1 (4%)	0 (0%)
**Feels unwell**	**Baseline**	3 (11%)	11 (41%)	7 (26%)	6 (22%)	0 (0%)	2 (7%)	6 (21%)	18 (62%)	3 (10%)	0 (0%)
	**Day 7**	12 (57%)	6 (29%)	3 (14%)	0 (0%)	0 (0%)	9 (39%)	9 (39%)	5 (22%)	0 (0%)	0 (0%)
**Low energy tired**	**Baseline**	7 (26%)	12 (44%)	5 (19%)	3 (11%)	0 (0%)	2 (7%)	12 (41%)	14 (48%)	1 (3%)	0 (0%)
	**Day 7**	15 (71%)	6 (29%)	0 (0%)	0 (0%)	0 (0%)	11 (48%)	6 (26%)	6 (26%)	0 (0%)	0 (0%)
**Not playing well**	**Baseline**	12 (44%)	9 (33%)	3 (11%)	3 (11%)	0 (0%)	10 (34%)	11 (38%)	7 (24%)	1 (3%)	0 (0%)
	**Day 7**	16 (76%)	4 (19%)	1 (5%)	0 (0%)	0 (0%)	16 (70%)	3 (13%)	4 (17%)	0 (0%)	0 (0%)
**Crying more than usual**	**Baseline**	7 (26%)	10 (37%)	5 (19%)	5 (19%)	0 (0%)	4 (14%)	8 (28%)	13 (45%)	4 (14%)	0 (0%)
	**Day 7**	12 (57%)	7 (33%)	1 (5%)	1 (5%)	0 (0%)	12 (52%)	7 (30%)	4 (17%)	0 (0%)	0 (0%)
**Needing extra care**	**Baseline**	7 (26%)	10 (37%)	8 (30%)	2 (7%)	0 (0%)	4 (14%)	11 (38%)	13 (45%)	1 (3%)	0 (0%)
	**Day 7**	14 (67%)	7 (33%)	0 (0%)	0 (0%)	0 (0%)	11 (48%)	6 (26%)	5 (22%)	1 (4%)	0 (0%)
**Clinginess**	**Baseline**	2 (7%)	12 (44%)	9 (33%)	4 (15%)	0 (0%)	2 (7%)	6 (21%)	14 (48%)	7 (24%)	0 (0%)
	**Day 7**	10 (48%)	7 (33%)	4 (19%)	0 (0%)	0 (0%)	6 (26%)	8 (35%)	7 (30%)	2 (9%)	0 (0%)
**Headache**	**Baseline**	14 (52%)	2 (7%)	1 (4%)	0 (0%)	10 (37%)	14 (48%)	2 (7%)	1 (3%)	1 (3%)	11 (38%)
	**Day 7**	15 (71%)	2 (10%)	0 (0%)	0 (0%)	4 (19%)	15 (65%)	1 (4%)	2 (9%)	0 (0%)	5 (22%)
**Sore throat**	**Baseline**	11 (41%)	3 (11%)	5 (19%)	0 (0%)	8 (30%)	8 (28%)	4 (14%)	4 (14%)	4 (14%)	9 (31%)
	**Day 7**	15 (71%)	1 (5%)	1 (5%)	0 (0%)	4 (19%)	12 (52%)	5 (22%)	1 (4%)	0 (0%)	5 (22%)

Rombach I, *et al. BMJ Open* 2022; 12:e049373. doi: 10.1136/bmjopen-2021-049373

		Co-amoxiclav Baseline (N = 27) Day 7 (N = 21)					Placebo Baseline (N = 29) Day 7 (N = 23)				
		**No Problem**	**Minor Problem**	**Moderate Problem**	**Major Problem**	**Missing**	**No Problem**	**Minor Problem**	**Moderate Problem**	**Major Problem**	**Missing**
**Muscle aches and pains**	**Baseline**	14 (52%)	3 (11%)	1 (4%)	0 (0%)	9 (33%)	10 (34%)	2 (7%)	3 (10%)	1 (3%)	13 (45%)
	**Day 7**	16 (76%)	1 (5%)	0 (0%)	0 (0%)	4 (19%)	15 (65%)	1 (4%)	1 (4%)	0 (0%)	6 (26%)
**Fever**	**Baseline**	9 (33%)	6 (22%)	7 (26%)	5 (19%)	0 (0%)	5 (17%)	10 (34%)	11 (38%)	3 (10%)	0 (0%)
	**Day 7**	19 (90%)	2 (10%)	0 (0%)	0 (0%)	0 (0%)	20 (87%)	3 (13%)	0 (0%)	0 (0%)	0 (0%)
**Cough**	**Baseline**	3 (11%)	6 (22%)	9 (33%)	9 (33%)	0 (0%)	1 (3%)	6 (21%)	14 (48%)	8 (28%)	0 (0%)
	**Day 7**	7 (33%)	11 (52%)	2 (10%)	1 (5%)	0 (0%)	5 (22%)	4 (17%)	9 (39%)	5 (22%)	0 (0%)
**Nasal congestion, runny nose**	**Baseline**	1 (4%)	8 (30%)	13 (48%)	5 (19%)	0 (0%)	1 (3%)	6 (21%)	15 (52%)	7 (24%)	0 (0%)
	**Day 7**	9 (43%)	8 (38%)	3 (14%)	1 (5%)	0 (0%)	3 (13%)	4 (17%)	12 (52%)	4 (17%)	0 (0%)
**Vomiting**	**Baseline**	21 (78%)	4 (15%)	0 (0%)	2 (7%)	0 (0%)	22 (76%)	3 (10%)	3 (10%)	1 (3%)	0 (0%)
	**Day 7**	20 (95%)	1 (5%)	0 (0%)	0 (0%)	0 (0%)	20 (87%)	3 (13%)	0 (0%)	0 (0%)	0 (0%)
**Not interested in what's going on**	**Baseline**	19 (70%)	4 (15%)	4 (15%)	0 (0%)	0 (0%)	17 (59%)	10 (34%)	2 (7%)	0 (0%)	0 (0%)
	**Day 7**	18 (86%)	3 (14%)	0 (0%)	0 (0%)	0 (0%)	18 (78%)	3 (13%)	2 (9%)	0 (0%)	0 (0%)
**Unable to get out of bed**	**Baseline**	23 (85%)	3 (11%)	1 (4%)	0 (0%)	0 (0%)	26 (90%)	2 (7%)	1 (3%)	0 (0%)	0 (0%)
	**Day 7**	21 (100%)	0 (0%)	0 (0%)	0 (0%)	0 (0%)	22 (96%)	1 (4%)	0 (0%)	0 (0%)	0 (0%)

Note: The scoring algorithm used allowed for the total score to be calculated if there was missing data in items 10-12, but not in any other items.

Rombach I, *et al. BMJ Open* 2022; 12:e049373. doi: 10.1136/bmjopen-2021-049373

Supplemental Table 3i: Responses to CARIFS domains for children aged 2+ years (only including questionnaires with sufficient data to calculate the overall CARIFS score)

		**Co-amoxiclav** Baseline (N = 72) Day 7 (N = 45)					**Placebo** Baseline (N = 68) Day 7 (N = 34)				
		**No Problem**	**Minor Problem**	**Moderate Problem**	**Major Problem**	**Missing**	**No Problem**	**Minor Problem**	**Moderate Problem**	**Major Problem**	**Missing**
**Poor appetite**	**Baseline**	14 (19%)	26 (36%)	25 (35%)	7 (10%)	0 (0%)	12 (18%)	20 (29%)	30 (44%)	6 (9%)	0 (0%)
	**Day 7**	24 (53%)	17 (38%)	3 (7%)	1 (2%)	0 (0%)	12 (35%)	16 (47%)	4 (12%)	2 (6%)	0 (0%)
**Not sleeping well**	**Baseline**	10 (14%)	18 (25%)	33 (46%)	11 (15%)	0 (0%)	12 (18%)	17 (25%)	26 (38%)	13 (19%)	0 (0%)
	**Day 7**	31 (69%)	7 (16%)	5 (11%)	2 (4%)	0 (0%)	13 (38%)	12 (35%)	5 (15%)	4 (12%)	0 (0%)
**Irritable, cranky, fussy**	**Baseline**	13 (18%)	22 (31%)	30 (42%)	7 (10%)	0 (0%)	13 (19%)	19 (28%)	28 (41%)	8 (12%)	0 (0%)
	**Day 7**	18 (40%)	20 (44%)	4 (9%)	3 (7%)	0 (0%)	15 (44%)	11 (32%)	4 (12%)	4 (12%)	0 (0%)
**Feels unwell**	**Baseline**	1 (1%)	18 (25%)	39 (54%)	14 (19%)	0 (0%)	7 (10%)	16 (24%)	30 (44%)	15 (22%)	0 (0%)
	**Day 7**	27 (60%)	12 (27%)	4 (9%)	2 (4%)	0 (0%)	19 (56%)	7 (21%)	5 (15%)	3 (9%)	0 (0%)
**Low energy tired**	**Baseline**	7 (10%)	18 (25%)	36 (50%)	11 (15%)	0 (0%)	11 (16%)	19 (28%)	27 (40%)	11 (16%)	0 (0%)
	**Day 7**	23 (51%)	14 (31%)	7 (16%)	1 (2%)	0 (0%)	17 (50%)	10 (29%)	4 (12%)	3 (9%)	0 (0%)
**Not playing well**	**Baseline**	15 (21%)	27 (38%)	26 (36%)	4 (6%)	0 (0%)	24 (35%)	17 (25%)	18 (26%)	9 (13%)	0 (0%)
	**Day 7**	34 (76%)	9 (20%)	1 (2%)	1 (2%)	0 (0%)	22 (65%)	6 (18%)	5 (15%)	1 (3%)	0 (0%)
**Crying more than usual**	**Baseline**	30 (42%)	18 (25%)	20 (28%)	4 (6%)	0 (0%)	28 (41%)	18 (26%)	14 (21%)	8 (12%)	0 (0%)
	**Day 7**	28 (62%)	10 (22%)	5 (11%)	2 (4%)	0 (0%)	21 (62%)	9 (26%)	2 (6%)	2 (6%)	0 (0%)
**Needing extra care**	**Baseline**	16 (22%)	24 (33%)	28 (39%)	4 (6%)	0 (0%)	16 (24%)	22 (32%)	23 (34%)	7 (10%)	0 (0%)
	**Day 7**	29 (64%)	12 (27%)	2 (4%)	2 (4%)	0 (0%)	21 (62%)	11 (32%)	1 (3%)	1 (3%)	0 (0%)

Rombach I, *et al. BMJ Open* 2022; 12:e049373. doi: 10.1136/bmjopen-2021-049373

		**Co-amoxiclav** Baseline (N = 72) Day 7 (N = 45)					**Placebo** Baseline (N = 68) Day 7 (N = 34)				
		**No Problem**	**Minor Problem**	**Moderate Problem**	**Major Problem**	**Missing**	**No Problem**	**Minor Problem**	**Moderate Problem**	**Major Problem**	**Missing**
**Clinginess**	**Baseline**	14 (19%)	20 (28%)	29 (40%)	9 (13%)	0 (0%)	21 (31%)	19 (28%)	20 (29%)	8 (12%)	0 (0%)
	**Day 7**	30 (67%)	10 (22%)	3 (7%)	2 (4%)	0 (0%)	23 (68%)	8 (24%)	2 (6%)	1 (3%)	0 (0%)
**Headache**	**Baseline**	24 (33%)	18 (25%)	23 (32%)	1 (1%)	6 (8%)	30 (44%)	15 (22%)	9 (13%)	7 (10%)	7 (10%)
	**Day 7**	32 (71%)	5 (11%)	2 (4%)	1 (2%)	5 (11%)	28 (82%)	3 (9%)	1 (3%)	2 (6%)	0 (0%)
**Sore throat**	**Baseline**	18 (25%)	18 (25%)	26 (36%)	8 (11%)	2 (3%)	24 (35%)	17 (25%)	12 (18%)	11 (16%)	4 (6%)
	**Day 7**	32 (71%)	7 (16%)	2 (4%)	1 (2%)	3 (7%)	21 (62%)	7 (21%)	3 (9%)	1 (3%)	2 (6%)
**Muscle aches and pains**	**Baseline**	27 (38%)	15 (21%)	20 (28%)	5 (7%)	5 (7%)	28 (41%)	17 (25%)	8 (12%)	10 (15%)	5 (7%)
	**Day 7**	28 (62%)	9 (20%)	0 (0%)	2 (4%)	6 (13%)	23 (68%)	7 (21%)	3 (9%)	1 (3%)	0 (0%)
**Fever**	**Baseline**	6 (8%)	29 (40%)	28 (39%)	9 (13%)	0 (0%)	11 (16%)	20 (29%)	23 (34%)	14 (21%)	0 (0%)
	**Day 7**	42 (93%)	2 (4%)	1 (2%)	0 (0%)	0 (0%)	28 (82%)	1 (3%)	3 (9%)	2 (6%)	0 (0%)
**Cough**	**Baseline**	1 (1%)	12 (17%)	43 (60%)	16 (22%)	0 (0%)	2 (3%)	13 (19%)	24 (35%)	29 (43%)	0 (0%)
	**Day 7**	14 (31%)	18 (40%)	9 (20%)	4 (9%)	0 (0%)	8 (24%)	14 (41%)	6 (18%)	6 (18%)	0 (0%)
**Nasal congestion, runny nose**	**Baseline**	4 (6%)	14 (19%)	37 (51%)	17 (24%)	0 (0%)	6 (9%)	18 (26%)	26 (38%)	18 (26%)	0 (0%)
	**Day 7**	12 (27%)	25 (56%)	8 (18%)	0 (0%)	0 (0%)	13 (38%)	15 (44%)	4 (12%)	2 (6%)	0 (0%)
**Vomiting**	**Baseline**	52 (72%)	17 (24%)	3 (4%)	0 (0%)	0 (0%)	58 (85%)	6 (9%)	2 (3%)	2 (3%)	0 (0%)
	**Day 7**	42 (93%)	2 (4%)	0 (0%)	1 (2%)	0 (0%)	31 (91%)	3 (9%)	0 (0%)	0 (0%)	0 (0%)
**Not interested in what's going on**	**Baseline**	41 (57%)	22 (31%)	8 (11%)	1 (1%)	0 (0%)	38 (56%)	16 (24%)	9 (13%)	5 (7%)	0 (0%)
	**Day 7**	40 (89%)	4 (9%)	0 (0%)	1 (2%)	0 (0%)	29 (85%)	3 (9%)	1 (3%)	1 (3%)	0 (0%)

		Co-amoxiclav Baseline (N = 72) Day 7 (N = 45)					Placebo Baseline (N = 68) Day 7 (N = 34)				
		**No Problem**	**Minor Problem**	**Moderate Problem**	**Major Problem**	**Missing**	**No Problem**	**Minor Problem**	**Moderate Problem**	**Major Problem**	**Missing**
**Unable to get out of bed**	**Baseline**	56 (78%)	13 (18%)	3 (4%)	0 (0%)	0 (0%)	48 (71%)	12 (18%)	7 (10%)	1 (1%)	0 (0%)
	**Day 7**	41 (91%)	3 (7%)	1 (2%)	0 (0%)	0 (0%)	30 (88%)	2 (6%)	2 (6%)	0 (0%)	0 (0%)

Note: The scoring algorithm used allowed for the total score to be calculated if there was missing data in items 10-12, but not in any other items.

Supplemental File 4: Additional summaries of research use

Supplemental Table 4a: Details of all reported hospital admissions

	Co-amoxiclav (N=133)	Placebo (N=132)	Total (N=265)
**Hospital admissions**			
**0**	126 (95%)	125 (95%)	251 (95%)
**1**	6 (5%)	6 (5%)	12 (5%)
**2**	1 (1%)	1 (1%)	2 (1%)
**Total nights in hospital***			
**0**	126 (95%)	125 (95%)	251 (95%)
**1**	4 (3%)	1 (1%)	5 (2%)
**2**	1 (1%)	3 (2%)	4 (2%)
**3**	0 (0%)	1 (1%)	1 (0%)
**7**	1 (1%)	1 (1%)	2 (1%)
**9**	1 (1%)	0 (0%)	1 (0%)
**10**	0 (0%)	1 (1%)	1 (0%)
**Antibiotics received during hospital admission***			
**0**	128 (96%)	128 (97%)	256 (97%)
**1**	4 (3%)	4 (3%)	8 (3%)
**2**	1 (1%)	0 (0%)	1 (0%)
**Other drugs received during hospital admission***			
**0**	128 (96%)	126 (95%)	254 (96%)
**1**	2 (2%)	1 (1%)	3 (1%)
**2**	1 (1%)	3 (2%)	4 (2%)
**3**	1 (1%)	2 (2%)	3 (1%)
**6**	1 (1%)	0 (0%)	1 (0%)
**X-rays during hospital admission***			
**0**	131 (98%)	129 (98%)	260 (98%)
**1**	1 (1%)	3 (2%)	4 (2%)
**2**	1 (1%)	0 (0%)	1 (0%)
**Other investigations during hospital admission****			
**0**	131 (98%)	130 (98%)	261 (98%)
**1**	2 (2%)	2 (2%)	4 (2%)
**Emergency department visits**			
**0**	128 (96%)	127 (96%)	255 (96%)
**1**	5 (4%)	5 (4%)	10 (4%)

**The summaries encompass the entire 28-day follow-up period, i.e. participants may have had a total of two x-rays made up of x-rays during two separate hospital admissions. Similarly, the total nights in hospital may have accumulated from more than one hospital admission.*

[a] Participants who were reported to have been referred to the hospital team/ emergency department for acute admission during a re-consultation for the same illness episode for which the child was randomised, but for whom no hospital admission is recorded at this date, are classed as having visited emergency department.

Hospital admission episodes for at least one night were collected during the notes review.

The trial did not report any admissions to intensive care units (ICU).

Two participants were reported to have a hospital admission on the same day as their discharge day; these were counted as emergency department visits.

***Other investigations include blood samples (2), throat swap and nasal aspirate.*

Supplemental Table 4b: Overview of healthcare use for re-consultations due to clinical deterioration and hospitalisations related to the illness episode for which the child was randomised to ARCHIE

Number of	N (Co-amoxiclav)	Co-amoxiclav: Mean (S.D.)	N (Placebo)	Placebo: Mean (S.D.)	N (Difference)	Difference (95% CI)
Re-consultations	133	0.27 (0.49)	132	0.23 (0.48)	265	0.04 (95% CI -0.07, 0.15), p-value = 0.495
Antibiotics received at re-consultation	133	0.10 (0.30)	132	0.10 (0.30)	265	0.00 (95% CI -0.07, 0.07), p-value = 0.993
Other drugs received at re-consultation	133	0.06 (0.27)	132	0.09 (0.49)	265	-0.03 (95% CI -0.12, 0.07), p-value = 0.535
Chest x-rays at re-consultation	133	0.03 (0.17)	132	0.01 (0.09)	265	0.02 (95% CI -0.01, 0.06), p-value = 0.178
Other interventions at re-consultation	133	0.02 (0.12)	132	0.00 (0.00)	265	0.01 (95% CI -0.01, 0.04), p-value = 0.163
Hospital admissions	133	0.04 (0.19)	132	0.05 (0.22)	265	-0.01 (95% CI -0.06, 0.03), p-value = 0.511
Total nights in hospital	133	0.09 (0.65)	132	0.14 (0.74)	265	-0.05 (95% CI -0.18, 0.08), p-value = 0.432
Antibiotics received during hospital admission	133	0.02 (0.15)	132	0.02 (0.15)	265	0.00 (95% CI -0.03, 0.03), p-value = 0.976
Other drugs received during hospital admission	133	0.05 (0.33)	132	0.09 (0.45)	265	-0.04 (95% CI -0.13, 0.05), p-value = 0.398
X-rays during hospital admission	133	0.01 (0.09)	132	0.02 (0.12)	265	-0.01 (95% CI -0.03, 0.02), p-value = 0.596
Other investigations during hospital admission	133	0.00 (0.00)	132	0.02 (0.12)	265	-0.01 (95% CI -0.04, 0.01), p-value = 0.168
Emergency department visits	133	0.03 (0.17)	132	0.03 (0.17)	265	0.00 (95% CI -0.04, 0.04), p-value = 0.983

**Differences have been adjusted for the stratification factors age (used as continuous variable) and seasonal influenza vaccination status. Clustering by centre has been accounted for using the 'cluster' option in Stata's 'regress' command and robust standard errors were generated.*

Supplemental File 5: Number of days children were unable to attend school or nursery, and subsequent changes to childcare requirements

Supplemental Table 5: Daily activity and child care

	Co-amoxiclav					Placebo					Difference		
	N	Mean (S.D.)	Median	IQR	Range	N	Mean (S.D.)	Median	IQR	Range	N	Difference (95% CI)*	p-value
**Days of school/ nursery missed**													
**week 1**	53	2.6 (2.2)	3	1, 4	0, 7	49	2.4 (2.2)	2	1, 3	0, 12	102	0.1 (95% CI -0.8, 1.0)	0.832
**week 2**	44	0.5 (1.5)	0	0, 0	0, 7	39	0.6 (1.5)	0	0, 0	0, 7	83	-0.2 (95% CI -0.8, 0.5)	0.630
**week 3**	36	0.3 (1.0)	0	0, 0	0, 5	33	0.1 (0.5)	0	0, 0	0, 3	69	0.1 (95% CI -0.2, 0.5)	0.492
**week 4**	38	0.0 (0.2)	0	0, 0	0, 1	26	0.2 (1.0)	0	0, 0	0, 5	64	-0.2 (95% CI -0.6, 0.2)	0.404
**Days of work missed****													
**week 1**	45	1.1 (1.3)	0	0, 2	0, 4	35	1.3 (1.4)	1	0, 2	0, 5	80	-0.2 (95% CI -0.8, 0.4)	0.463
**week 2**	41	0.1 (0.3)	0	0, 0	0, 1	33	0.2 (0.7)	0	0, 0	0, 3	74	-0.1 (95% CI -0.3, 0.1)	0.345
**week 3**	34	0.1 (0.5)	0	0, 0	0, 2	27	0.0 (0.0)	0	0, 0	0, 0	61	0.1 (95% CI -0.1, 0.3)	0.205
**week 4**	33	0.0 (0.0)	0	0, 0	0, 0	22	0.3 (1.1)	0	0, 0	0, 5	55	-0.3 (95% CI -0.7, 0.2)	0.246
**Days on which usual activities were changed*****													
**week 1**	37	2.0 (2.4)	2	0, 4	0, 7	34	2.8 (2.0)	3	1, 4	0, 7	71	-0.8 (95% CI -1.8, 0.2)	0.132
**week 2**	33	0.8 (2.1)	0	0, 0	0, 7	37	0.8 (1.6)	0	0, 0	0, 5	70	0.1 (95% CI -0.8, 1.0)	0.833
**week 3**	26	0.2 (0.7)	0	0, 0	0, 3	32	0.5 (1.7)	0	0, 0	0, 7	58	0.0 (95% CI -0.6, 0.5)	0.877
**week 4**	26	0.0 (0.2)	0	0, 0	0, 1	23	0.3 (0.8)	0	0, 0	0, 3	49	-0.2 (95% CI -0.6, 0.1)	0.150
**Days outside carer required**													
**week 1**	40	0.6 (1.2)	0	0, 1	0, 4	31	0.8 (2.1)	0	0, 1	0, 11	71	-0.3 (95% CI -1.2, 0.6)	0.520
**week 2**	40	0.3 (1.0)	0	0, 0	0, 5	34	0.1 (0.3)	0	0, 0	0, 2	74	0.2 (95% CI -0.2, 0.5)	0.344
**week 3**	34	0.1 (0.5)	0	0, 0	0, 2	34	0.1 (0.5)	0	0, 0	0, 3	68	0.0 (95% CI -0.2, 0.2)	0.933
**week 4**	34	0.2 (1.0)	0	0, 0	0, 6	30	0.1 (0.5)	0	0, 0	0, 3	64	0.1 (95% CI -0.3, 0.5)	0.637

**Differences have been adjusted for the stratification factors age (used as continuous variable) and seasonal influence vaccination status. Clustering by centre has been accounted for using the 'cluster' option in Stata's 'regress' command and robust standard errors were generated.*

***Collected for parents/ carers in paid employment; *** Collected for parents/ carers not in paid employment*

SD: Standard deviation; IQR: Interquartile range
