# Supplementary material for: Quality of life, healthcare use and costs in ‘at-risk’ children after early antibiotic treatment versus placebo for influenza-like illness: within-trial descriptive economic analyses of the ARCHIE randomised controlled trial
Source: BMJ Open. 2022 Apr 15;12(4):e049373. doi: 10.1136/bmjopen-2021-049373 (PMC9014043; doi:10.1136/bmjopen-2021-049373)
Supplement: Author's manuscript [file bmjopen-2021-049373.draft_revisions.pdf]

BMJ Open

BMJ Open is committed to open peer review. As part of this commitment we make the peer review history of every article we publish publicly available.

When an article is published we post the peer reviewers' comments and the authors' responses online. We also post the versions of the paper that were used during peer review. These are the versions that the peer review comments apply to.

The versions of the paper that follow are the versions that were submitted during the peer review process. They are not the versions of record or the final published versions. They should not be cited or distributed as the published version of this manuscript.

BMJ Open is an open access journal and the full, final, typeset and author-corrected version of record of the manuscript is available on our site with no access controls, subscription charges or pay-per-view fees (http://bmjopen.bmj.com).

If you have any questions on BMJ Open's open peer review process please email info.bmjopen@bmj.com

BMJ Open

Quality of life, healthcare use and costs in 'at risk' children after early antibiotic treatment vs. placebo for influenza-like illness: Within-trial economic analyses of the ARCHIE randomised controlled trial

Journal:	*BMJ Open*
Manuscript ID	bmjopen-2021-049373
Article Type:	Original research
Date Submitted by the Author:	25-Jan-2021
Complete List of Authors:	Rombach, Ines; Oxford University, Nuffield Department of Population Health<br>Wang, Kay; University of Oxford Nuffield Department of Primary Care Health Sciences, Nuffield Department of Primary Care Health Sciences<br>Tonner, Sharon; University of Oxford Nuffield Department of Primary Care Health Sciences, Nuffield Department of Primary Care Health Sciences<br>Grabey, Jenna; University of Oxford Nuffield Department of Primary Care Health Sciences<br>Harnden, Anthony; University of Oxford Nuffield Department of Primary Care Health Sciences, Dept of Primary Health Care<br>Wolstenholm, Jane L; Oxford University, Nuffield Department of Population Health
Keywords:	HEALTH ECONOMICS, INFECTIOUS DISEASES, Paediatric infectious disease & immunisation < PAEDIATRICS, PAEDIATRICS, PRIMARY CARE, RESPIRATORY MEDICINE (see Thoracic Medicine)

SCHOLARONE™
Manuscripts

I, the Submitting Author has the right to grant and does grant on behalf of all authors of the Work (as defined in the below author licence), an exclusive licence and/or a non-exclusive licence for contributions from authors who are: i) UK Crown employees; ii) where BMJ has agreed a CC-BY licence shall apply, and/or iii) in accordance with the terms applicable for US Federal Government officers or employees acting as part of their official duties; on a worldwide, perpetual, irrevocable, royalty-free basis to BMJ Publishing Group Ltd ("BMJ") its licensees and where the relevant Journal is co-owned by BMJ to the co-owners of the Journal, to publish the Work in this journal and any other BMJ products and to exploit all rights, as set out in our licence.

The Submitting Author accepts and understands that any supply made under these terms is made by BMJ to the Submitting Author unless you are acting as an employee on behalf of your employer or a postgraduate student of an affiliated institution which is paying any applicable article publishing charge ("APC") for Open Access articles. Where the Submitting Author wishes to make the Work available on an Open Access basis (and intends to pay the relevant APC), the terms of reuse of such Open Access shall be governed by a Creative Commons licence – details of these licences and which Creative Commons licence will apply to this Work are set out in our licence referred to above.

Other than as permitted in any relevant BMJ Author's Self Archiving Policies, I confirm this Work has not been accepted for publication elsewhere, is not being considered for publication elsewhere and does not duplicate material already published. I confirm all authors consent to publication of this Work and authorise the granting of this licence.

For peer review only

Quality of life, healthcare use and costs in 'at risk' children after early antibiotic treatment vs. placebo for influenza-like illness: Within-trial economic analyses of the ARCHIE randomised controlled trial

Authors

Ines Rombach (ines.rombach@ndph.ox.ac.uk)[1],

Kay Wang (kay.wang@phc.ox.ac.uk)[2],

Sharon Tonner (sharon.tonner@phc.ox.ac.uk)[2],

Jenna Grabey (jenna.grabey@phc.ox.ac.uk)[2],

Anthony Harnden (anthony.harnden@phc.ox.ac.uk)[2],

Jane L Wolstenholme (jane.wolstenholme@ndph.ox.ac.uk)[1],

for the ARCHIE Investigators*

*A complete list of ARCHIE investigators can be found at the end of the manuscript

Affiliations

[1]Health Economics Research Centre, Nuffield Department of Population Health, Oxford University, Oxford, UK

[2]Nuffield Department of Primary Care Health Sciences, University of Oxford, Oxford, UK

For peer review only - http://bmjopen.bmj.com/site/about/guidelines.xhtml

Corresponding author:

Ines Rombach

Health Economics Research Centre

Nuffield Department of Population Health

Old Road Campus

University of Oxford

Oxford

OX3 7LF

United Kingdom

ines.rombach@ndorms.ox.ac.uk

https://orcid.org/0000-0003-3464-3867

Keywords

Antibiotic use; healthcare resource use; cost; quality of life (QoL); children; paediatric research; influenza-like illness; economic analysis

Word count:

Abstract: 295

Main text (excluding figures and tables): 3969

For peer review only

Abstract

Objectives: To characterise the quality of life, healthcare use and costs associated with early antibiotic treatment of influenza-like illness (ILI) in 'at risk' children.

Design: Economic analysis of a two-arm double-blind parallel group pragmatic randomised controlled trial.

Setting: Children were recruited from community-based health care settings, including general practices, walk-in centres and hospital ambulatory care.

Participants: Children with risk factors for influenza-related complications, including respiratory, cardiac and neurological conditions, who presented within the first five days of an ILI.

Interventions: Co-amoxiclav 400/57 suspension or placebo.

Outcome measures: This economic analysis focussed on quality of life measured by the EQ-5D-Y, symptoms assessed by the Canadian Acute Respiratory Infection and Flu Scale (CARIFS), and healthcare use and costs including medication, hospital visits and admissions, GP and nurse contacts. Outcomes were assessed for up to 28 days post randomisation.

Results: Information on resource use, EQ-5D-Y (day 28) and CARIFS (day 7) was available for 265 (98%), 72 (27%) and 123 (45%) out of 271 participants, respectively. Average costs in the co-amoxiclav group were £25 lower (95% confidence interval [CI] -113 to 65), but this difference was not statistically significant (p-value: 0.566). The difference in EQ-5D-Y scores between groups was also not statistically significant (-0.014 (95% CI -0.124, 0.096), p-value: 0.798). However, day 7 CARIFS scores were 3.5 points lower in the co-amoxiclav arm (95% CI -6.9 to -0.1, p-value: 0.044).

Conclusions: Our findings did not show evidence that early co-amoxiclav treatment improves quality of life or reduces healthcare use and costs in 'at risk' children with ILI, but may reduce symptom severity. Reliable data collection from children's parents/carers was challenging, and

For peer review only - http://bmjopen.bmj.com/site/about/guidelines.xhtml

resulted in high levels of missing data, which is common in pragmatic trials involving children with acute respiratory tract infections.

Trial registration: ISRCTN 70714783; EudraCT 2013-002822-21

For peer review only

For peer review only - http://bmjopen.bmj.com/site/about/guidelines.xhtml

Article Summary

Strength and limitations

- This study aimed to assess quality of life, healthcare use and costs in children with influenza-like illness (ILI) and known risk factors for complications.
- The study used pragmatic inclusion criteria, recruiting children with ILI, which reflects current clinical practice but cannot reliably distinguish between influenza infection and ILI due to other infections.
- Subgroup analyses examined differential costs between children with and without laboratory-confirmed influenza.
- Reviews of medical records provided robust information on healthcare use with negligible levels of missing data.
- Given the nature of the participant group, proxy-reported completion of instruments and questionnaires was required to inform the quality of life, healthcare use and costs, however this was subject to high levels of missing data, making a formal cost-effectiveness analysis impossible to undertake.

For peer review only

For peer review only - http://bmjopen.bmj.com/site/about/guidelines.xhtml

Background

The clinical results of the 'Early use of Antibiotics for at Risk CHildren with InfluEnza-like illness' (ARCHIE) trial were recently published (ISRCTN 70714783). This randomised controlled trial (RCT) aimed to assess uncertainty in the treatment of influenza-like illness (ILI) in 'at risk' children, i.e. children with pre-existing conditions such as asthma, diabetes mellitus and cerebral palsy.

The RCT compared the early treatment with oral co-amoxiclav with placebo, and used proportion of children re-consulting due to clinical deterioration within 28 days of randomisation as the primary outcome measure.

Full details of the trial are published elsewhere[1]. The primary intention to treat (ITT) analysis did not show a statistically significant difference between the co-amoxiclav and placebo groups, as measured by the proportion of children re-consulting due to clinical deterioration within 28 days. A re-consultation rate of 24.8% was observed in the co-amoxiclav arm, and 21.2% in the placebo arm (adjusted risk ratio 0.86 (95% CI 0.56, 1.34). An exploratory analysis in the subgroup with laboratory-confirmed influenza showed that a lower proportion of children in the co-amoxiclav group re-consulted due to clinical deterioration, although this subgroup did not have sufficient power to demonstrate whether this difference was statistically significant.

The trial also investigated health-related quality of life (QoL), healthcare use and cost associated with the early co-amoxiclav use in 'at risk' children. This research is important due to the absence of quality of life and healthcare use data associated with early antibiotic use associated with influenza or ILI in this important patient population. Our literature review for our pre-trial modelling work demonstrated a lack of observational data in these 'at risk' children, and we were unable to identify any clinical trials in this paediatric cohort[2-4]. Previous UK based cost-effectiveness models of antivirals for treatment of influenza in children used adult utility values based on derived values from adults treated with oseltamivir[5,6]. An

For peer review only - http://bmjopen.bmj.com/site/about/guidelines.xhtml

alternative source of child-specific proxy EQ-5D data was from a trial of corticosteroids for otitis media[7].

Methods

Design and planned analyses for the ARCHIE trial, including the health economic analysis, are described in the published protocol[1]. Specific analyses of the quality of life, healthcare use and cost data are described below.

Quality of life

Quality of life was measured using the EQ-5D 3-Level youth version (EQ-5D-Y), which was confirmed in pre-trial focus groups as most applicable in this patient population, compared to other questionnaires (i.e. Child Health Utility-9D)[8]. The EQ-5D-Y was collected at baseline, days 4, 7, 14 and 28. For all children, parents or carers (i.e. proxies) completed the data on the children's behalf. Data were also collected from the participants directly, where appropriate. No restrictions on which children should complete the EQ-5D-Y themselves were included in the protocol. This was due to some of the participants having complex needs and therefore being unable to complete questionnaires regardless of their age. Equally, severe illness may have prevented some children who would usually be able to complete the data from doing so at some trial follow-up time points. Whether or not children were asked to complete questionnaires was left to their proxy's discretion.

The EQ-5D-Y consists of five questions covering mobility, self-care, usual activities, pain/ discomfort, and anxiety/ depression. Each question has three response levels; 'no', 'some', 'a lot'. As the scoring for the youth-version had not been published at the time of the analysis of this study[8,9], the responses to the five questions were converted into utilities using the EQ-5D-3L algorithm for the UK value set[10-12], ranging from -0.594 to 1, where 1 indicates perfect health.

The health state utilities calculated from the responses to EQ-5D-Y were used to estimate mean QALYs rated on behalf of the child and by the child for both trial arms at 28 days. This

For peer review only - http://bmjopen.bmj.com/site/about/guidelines.xhtml

was done using an area under the curve (AUC) approach, whereby utilities were assumed to change linearly between each follow-up time point, weighted by the time periods the questionnaires were administered, i.e. days 4, 7, 14 and 28. QALYs were generated only for participants who had EQ-5D-Y data available at baseline and all follow-up time points to 28 days.

Canadian Acute Respiratory Infection and Flu Scale (CARIFS)

The Canadian Acute Respiratory Infection and Flu Scale (CARIFS)[13] consists of 18 questions covering three domains, i.e. symptoms, function and parental impact. The questions have 4-point ordinal scale, from no problems to major problems, scored 0 to 3. A total score ranging from 0 (best possible health) to 54 (worst possible health) is calculated by adding up all 18 items. Missing data in the three questions about headache, sore throat, muscle aches or pains were handled by mean imputation, in line with the scoring instructions[13]. The CARIFS was collected at baseline and 1 week.

Healthcare use

Proxy-reported healthcare use was collected in the study diaries with regards to medication use, hospital visits and admissions, GP and nurse contacts. Information on the number of days children were unable to attend school or nursery, and subsequent changes to childcare requirements were also collected. These data were collected in weekly diaries on days 7, 14, 21 and 28, requiring recall of health care resource use over the previous week.

A notes review was conducted to obtain clinical data for the primary outcome (re-consultation due to clinical deterioration), medication prescriptions and/or further investigations, and hospitalisations or deaths within 28 days of randomisation. The number of re-consultations, antibiotics and other drugs given, chest x-rays and other investigations performed were included in this review and were used in the estimation of healthcare use.

For peer review only - http://bmjopen.bmj.com/site/about/guidelines.xhtml

For the purposes of this analysis, only re-consultations due to clinical deterioration considered as related to the initial illness episode for which the child was randomised were included, in line with the primary endpoint of the trial. Re-consultations had to take place within 28 days from randomisation and in relation to worsening symptoms, new symptoms or complications for which the child required medication, investigation or referral to hospital.

Referrals to hospital or emergency departments for acute admission were recorded from the notes review including information on hospital inpatient admissions, medications and investigations undertaken.

The study's clinical lead reviewed all hospital admissions, and distinguished between those considered to be related to the illness episode the child was randomised for, and those not related. They also reviewed all medication recorded. Medications deemed unrelated to the illness for which the child was recruited into the trial were identified and excluded from this analysis.

Information on healthcare use was restricted to the information obtained via the notes review. Information from the carer-completed diaries was considered insufficient for inclusion in this analysis (details on data availability for the self-reported questionnaires are provided in the results section for information). Serious Adverse Events as reported by the recruiting sites were reviewed to ensure all relevant health care use was captured through the notes review.

For peer review only

Unit costs

Participating children were recruited from GP practices and hospital-based ambulatory assessment units. Unit costs for GP practice visits or re-consultations with a GP were obtained from the PSSRU Unit Costs of Health and Social Care compendium for 2017/2018[14]. Unit costs for all other health service contacts were derived from the NHS National Schedule of Reference Costs 2017 to 2018[15], including re-consultations for participants recruited in

For peer review only - http://bmjopen.bmj.com/site/about/guidelines.xhtml

paediatric ambulatory assessment units, which were costed as a visit to a paediatric ambulatory assessment unit.

Investigations, including blood tests, nasal aspirate, and throat swabs were costed based on information on Directly Accessed Pathology Services in the NHS National Schedule of Reference Costs 2017 to 2018[15]. Costs of urine tests were based on information published by Fenwick et al[16].

Antibiotics and other prescription drug costs were based on the British National Formulary (BNF for children) and UK tariff costs[17], assuming the minimum value reported. Where possible, unit costs were applied based on the dose reported, or a median dose if no dose was reported; cost for inhalers and eye/nose drops were based on the minimum unit sold.

Information on the unit costs included in this trial are presented in Supplemental File 1.

Analysis

Within-trial analysis

Summaries were generated on an intention-to-treat basis. All available data were used, only participants whose parents or carers withdrew consent for extraction of data from their children's medical record, or where access to medical notes was not possible for other reasons were excluded from the summaries.

Data availability was summarised for the EQ-5D-3L (completed by the child, and on their behalf (proxy)), CARIFS, availability of daily activity and childcare questionnaire, and availability of health service contact (proxy-reported).

EQ-5D-Y-3-level, QALYs and CARIFS data were summarised descriptively by data collection time point. Healthcare use was summarised using number of events and frequencies to indicate how often participants had used each service. Mean number of contacts with different health services were also calculated. Costs incurred for the different health service contacts

For peer review only - http://bmjopen.bmj.com/site/about/guidelines.xhtml

were calculated by applying the unit costs, described in Supplemental File 1. Information on the number of days children were unable to attend school or nursery, and subsequent changes to childcare requirements reported during each of the follow-up weeks were also summarised.

Differences between trial arms were estimated using linear regression models, adjusted for the minimisation factors; age (used as continuous variable) and seasonal influenza vaccination status. Clustering by the recruiting site (i.e. the GP practices or hospital ambulatory units at which participants were randomised) was accounted for using the 'cluster' option in Stata's 'regress' command and robust standard errors were generated.

Additional analyses and sensitivity analyses

Subgroup analyses explored if treatment effects varied depending on whether the children's illness at baseline was laboratory-confirmed to be influenza, by adding an interaction term between treatment allocation and laboratory-confirmed influenza at baseline to the above described analysis model. Subgroup analyses were performed for the total costs incurred over the trial follow-up period, and separately for the costs incurred through medication use, and the costs related to re-consultations, hospital admissions and referrals. Results from this investigation were presented as forest plots. As this analysis was exploratory, no p-values were displayed.

Sensitivity analysis examined the effect of including costs associated with hospitalisations unrelated to the illness episode for which the child was recruited to ARCHIE.

Extrapolation of findings to national scale

The costs were extrapolated to estimate the impact of either intervention on a national scale. The number of children within the relevant age category in the UK were estimated from information for 2018 from the Office for National Statistics[18]. The number of GP consultations

For peer review only - http://bmjopen.bmj.com/site/about/guidelines.xhtml

during 2018/2019 was estimated from the 'Surveillance of influenza and other respiratory viruses in the UK'[19]. Finally, the proportions of children who consult with GPs for influenza like illnesses who correspond to the ARCHIE trial population were estimated from the current literature[20].

From these figures, the total number of 'at risk' children likely to consult a GP with influenza like illnesses will be estimated for the UK. This figure was combined with the estimations of cost differences between the co-amoxiclav and placebo groups, to estimate the likely monetary impact of the co-amoxiclav intervention on a national scale.

For peer review only

Patient and public involvement

The research team conducted consultations with parents of 'at risk' children and young people from the National Institute for Health Research Generation R Young Person's Advisory Group to inform the design and conduct of the ARCHIE programme. The ARCHIE Programme Steering Committee also included a patient representative.

Results

Data availability

In total, 271 children were randomised into the trial, 136 to co-amoxiclav, 135 to placebo. Six children were excluded. Of these, five children were withdrawn from the trial and all follow-up, and medical notes of one child could not be accessed. As such, 265 children were included in the analysis (133 randomised to co-amoxiclav, 132 to placebo). All 265 children received their allocated intervention, and there were no cross-overs between the treatment arms. Data on healthcare resource use based on note reviews were available for all 265 children.

For peer review only - http://bmjopen.bmj.com/site/about/guidelines.xhtml

Proxy-completed EQ-5D-Y data were available for 75% (204/271) of children at baseline, and 27% (72/271) at 28 days. Self-completed EQ-5D-Y data were available for 23% (61/271) at baseline, and 10% (27/271) at 28 days. CARIFS data were completed for 72% (196/271) of children at baseline and 45% (123/271) at day 7. Proxy-reported data for daily activity and childcare questionnaire, and the health service contact questions were available for 54% (146/271) at baseline, and less than 35% at 28 days respectively (92/271 daily activity and childcare questionnaires and 87/271 healthcare use questionnaires were received). Table 1 shows this information by treatment arm, and for all follow-up points. Additional information on data availability, including by age group, and EQ-5D-Y missing data pattern over time are available in Supplemental File 2.

For peer review only

For peer review only - http://bmjopen.bmj.com/site/about/guidelines.xhtml

Table 1: Overview of availability for self-reported data

	Co-amoxiclav (N=136)	Placebo (N=135)	Total (N=271)
**Availability of EQ-5D-Y index completed on behalf of child**			
**day 0**	96 (71%)	108 (80%)	204 (75%)
**day 4**	52 (38%)	52 (39%)	104 (38%)
**day 7**	52 (38%)	54 (40%)	106 (39%)
**day 14**	44 (32%)	46 (34%)	90 (33%)
**day 28**	41 (30%)	31 (23%)	72 (27%)
**Availability of EQ-5D VAS completed on behalf child**			
**day 0**	120 (88%)	129 (96%)	249 (92%)
**day 4**	77 (57%)	76 (56%)	153 (56%)
**day 7**	78 (57%)	73 (54%)	151 (56%)
**day 14**	61 (45%)	57 (42%)	118 (44%)
**day 28**	53 (39%)	40 (30%)	93 (34%)
**Availability of EQ-5D-Y index completed by child**			
**day 0**	32 (24%)	29 (21%)	61 (23%)
**day 4**	16 (12%)	13 (10%)	29 (11%)
**day 7**	17 (13%)	11 (8%)	28 (10%)
**day 14**	17 (13%)	10 (7%)	27 (10%)
**day 28**	17 (13%)	10 (7%)	27 (10%)
**Availability of EQ-5D VAS completed by child**			
**day 0**	28 (21%)	25 (19%)	53 (20%)
**day 4**	19 (14%)	13 (10%)	32 (12%)
**day 7**	19 (14%)	11 (8%)	30 (11%)
**day 14**	20 (15%)	10 (7%)	30 (11%)
**day 28**	18 (13%)	11 (8%)	29 (11%)
**Availability of CARIFS**			
**day 0**	99 (73%)	97 (72%)	196 (72%)
**day 7**	66 (49%)	57 (42%)	123 (45%)
**Availability of CARIFS VAS**			
**day 0**	133 (98%)	135 (100%)	268 (99%)
**day 7**	76 (56%)	74 (55%)	150 (55%)
**Availability of daily activity and childcare questionnaire**			
**day 7**	75 (55%)	71 (53%)	146 (54%)
**day 14**	61 (45%)	57 (42%)	118 (44%)
**day 21**	54 (40%)	46 (34%)	100 (37%)
**day 28**	53 (39%)	39 (29%)	92 (34%)
**Availability of healthcare use questionnaire**			
**day 7**	76 (56%)	70 (52%)	146 (54%)
**day 14**	56 (41%)	52 (39%)	108 (40%)

For peer review only

For peer review only - http://bmjopen.bmj.com/site/about/guidelines.xhtml

**day 21**	51 (38%)	45 (33%)	96 (35%)
**day 21**	51 (38%)	45 (33%)	96 (35%)
**day 28**	49 (36%)	38 (28%)	87 (32%)

Note: EQ-5D-Y and CARIFS were included as 'available' if all relevant questions were completed; the daily activities and childcare questionnaire, and the health service contact questionnaire were considered 'available' if at least one question had been answered, including indicating that the daily activities and childcare questions were not applicable.

For peer review only

For peer review only - http://bmjopen.bmj.com/site/about/guidelines.xhtml

Quality of life and healthcare use

Data for the EQ-5D-Y, QALYs and CARIFS are shown in Table 2. There were no statistically significant differences between the treatment arms for the EQ-5D-Y and QALY. The EQ-5D VAS completed on behalf of the children was statistically significantly higher, indicating higher quality of life, in the co-amoxiclav arm at 7 days post randomisation only. The CARIFS score was significantly lower in the co-amoxiclav arm, indicating lower severity of respiratory symptoms in those children. Reponses to the individual EQ-5D-Y and CARIFS items, for the whole cohort and split by age group, are presented in Supplemental File 3.

Details of healthcare use for related re-consultations due to clinical deterioration, and hospital admissions related to the illness episode for which the child was randomised into ARCHIE are shown in Table 3. Overall, 23% of children (61/265) had at least one re-consultation due to clinical deterioration, and hospital admissions related to the illness episode for which the child was randomised into ARCHIE were reported for 5% of children (12/265). Additional information on all hospital admissions and average healthcare use, as typically presented in cost-effectiveness analyses, are shown in Supplemental File 4. Resource use was similar between the treatment arms.

Costs associated with related re-consultations and hospital admissions are shown in table 4. Average total costs over 28-day follow-up period were estimated as £82 (SD 481) in the co-amoxiclav arm, and £117 (SD 540) in the placebo arm. The medians and IQRs of zero for most costing items reflect the fact that 75% of the participants did not use the relevant resources and hence did not incur corresponding costs. There were no statistically significant differences in total costs, or any of the cost-components, between the treatment groups. Hospital stays appear to be the main cost drivers.

For peer review only - http://bmjopen.bmj.com/site/about/guidelines.xhtml

Information on the number of days children were unable to attend school or nursery, and subsequent changes to childcare requirements are summarised in supplemental File 5. There were no statistically significant differences between the groups.

For peer review only

For peer review only - http://bmjopen.bmj.com/site/about/guidelines.xhtml

Table 2: EQ-5D-Y, quality adjusted life scores and CARIF outcomes

	Co-amoxiclav:		Placebo		Difference	
	N	Mean (S.D.)	N	Mean (S.D.)	Total N	Difference (95% CI)*
**EQ-5D-Y proxy, completed on behalf of child**						
Day 0	96	0.591 (0.342)	108	0.568 (0.386)		
Day 4	52	0.630 (0.349)	52	0.690 (0.318)	104	-0.057 (95% CI -0.198, 0.085), p-value = 0.425
Day 7	52	0.788 (0.360)	54	0.762 (0.343)	106	0.037 (95% CI -0.102, 0.176), p-value = 0.596
Day 14	44	0.841 (0.338)	46	0.923 (0.157)	90	-0.086 (95% CI -0.200, 0.028), p-value = 0.135
Day 28	41	0.905 (0.246)	31	0.923 (0.169)	72	-0.014 (95% CI -0.124, 0.096), p-value = 0.798
EQ-5D VAS completed on behalf of child						
Day 0	120	60 (17)	129	58 (19)		
Day 4	77	72 (18)	76	68 (20)	153	4.1 (95% CI -1.5, 9.7), p-value = 0.146
Day 7	78	83 (18)	73	76 (22)	151	7.9 (95% CI 1.3, 14.4), p-value = 0.019
Day 14	61	87 (19)	57	90 (13)	118	-3.7 (95% CI -8.7, 1.3), p-value = 0.139
Day 28	53	89 (17)	40	86 (21)	93	4.1 (95% CI -5.0, 13.3), p-value = 0.368
**EQ-5D-Y completed by child**						
Day 0	32	0.469 (0.388)	29	0.531 (0.363)		
Day 4	16	0.626 (0.386)	13	0.717 (0.227)	29	-0.107 (95% CI -0.372, 0.157), p-value = 0.408
Day 7	17	0.815 (0.386)	11	0.805 (0.180)	28	-0.034 (95% CI -0.336, 0.269), p-value = 0.819
Day 14	17	0.957 (0.086)	10	0.918 (0.144)	27	0.028 (95% CI -0.073, 0.130), p-value = 0.565
Day 28	17	0.986 (0.039)	10	0.941 (0.124)	27	0.035 (95% CI -0.039, 0.109), p-value = 0.330
EQ-5D completed by child						
Day 0	28	54 (22)	25	55 (21)		
Day 4	19	62 (22)	13	59 (21)	32	4.2 (95% CI -11.2, 19.7), p-value = 0.577
Day 7	19	73 (21)	11	73 (25)	30	-0.7 (95% CI -24.9, 23.4), p-value = 0.952
Day 14	20	84 (19)	10	83 (26)	30	1.4 (95% CI -19.8, 22.6), p-value = 0.893
Day 28	18	88 (21)	11	84 (25)	29	3.0 (95% CI -9.2, 15.2), p-value = 0.612
**QALYs - rated on behalf of child**	29	0.061 (0.022)	25	0.065 (0.011)	54	-0.003 (95% CI -0.012, 0.007), p-value = 0.573

For peer review only - http://bmjopen.bmj.com/site/about/guidelines.xhtml

**QALYs - rated by child**	9	0.069 (0.004)	4	0.065 (0.007)	13	0.003 (95% CI -0.004, 0.009), p-value = 0.357
**CARIFS**						
**Day 0**	99	22 (10)	97	23 (11)		
**Day 7**	66	8 (8)	57	12 (11)	123	-3.5 (95% CI -6.9, -0.1), p-value = 0.044
**CARIFS VAS**						
Day 0	133	4.64 (1.90)	135	4.78 (1.92)		
Day 7	76	1.91 (1.97)	74	2.53 (2.45)	150	-0.63 (95% CI -1.38, 0.11), p-value = 0.096

**Differences have been adjusted for the stratification factors age (used as continuous variable) and seasonal influence vaccination status. Clustering by centre has been accounted for using the 'cluster' option in Stata's 'regress' command and robust standard errors were generated.*
EQ-5D-Y utilities: range -0.594 to 1, where 1 indicates perfect health
EQ-5D VAS: range 0 to 100, where higher values indicate better health.
QALYs (Quality Adjusted Life Years) were calculated only for children with data available at all relevant time points.
CARIFS (Canadian Acute Respiratory Infection and Flu Scale): range 0 to 54 points, with lower scores indicating fewer respiratory illness and flu symptoms.
CARIFS VAS: range 0 to 10, with higher scores indicating worse health states
CI – confidence interval; SD – standard deviation; VAS – visual analogue score

For peer review only

For peer review only - http://bmjopen.bmj.com/site/about/guidelines.xhtml

Table 3: Details of healthcare use for related re-consultations due to clinical deterioration and hospital admissions related to the illness episode for which the child was randomised into ARCHIE over the 28-day follow-up period

	Co-amoxiclav (N=133)	Placebo (N=132)	Total (N=265)
**RELATED RE-CONSULTATIONS**			
**Number of participants with at least one related re-consultation due to clinical deterioration**	33 (25%)	28 (21%)	61 (23%)
**Number of related re-consultations due to clinical deterioration**			
**0**	100 (75%)	104 (79%)	204 (77%)
**1**	30 (23%)	25 (19%)	55 (21%)
**2**	3 (2%)	3 (2%)	6 (2%)
**Number of participants with at least one related re-consultation due to clinical deterioration in a primary care or equivalent ambulatory care setting (excluding hospital admissions)***	28 (21%)	23 (17%)	51 (19%)
**Related re-consultation due to clinical deterioration in a primary care or equivalent ambulatory care setting (excluding hospital admissions)***			
**0**	105 (79%)	109 (83%)	214 (81%)
**1**	25 (19%)	21 (16%)	46 (17%)
**2**	3 (2%)	2 (2%)	5 (2%)
**Number of antibiotics received at related re-consultation***			
**0**	120 (90%)	119 (90%)	239 (90%)
**1**	13 (10%)	13 (10%)	26 (10%)
**Number of other drugs received at related re-consultation***			
**0**	126 (95%)	127 (96%)	253 (95%)
**1**	6 (5%)	1 (1%)	7 (3%)
**2**	1 (1%)	1 (1%)	2 (1%)
**3**	0 (0%)	3 (2%)	3 (1%)
**Number of chest x-rays at related re-consultation***			
**0**	129 (97%)	131 (99%)	260 (98%)
**1**	4 (3%)	1 (1%)	5 (2%)
**Number of other interventions at related re-consultation*, ****			
**0**	131 (98%)	132 (100%)	263 (99%)
**1**	2 (2%)	0 (0%)	2 (1%)
**RELATED HOSPITAL ADMISSIONS**			
**Number of hospital admissions**			
**0**	128 (96%)	125 (95%)	253 (95%)
**1**	5 (4%)	7 (5%)	12 (5%)

For peer review only - http://bmjopen.bmj.com/site/about/guidelines.xhtml

	Co-amoxiclav (N=133)	Placebo (N=132)	Total (N=265)
**Total nights in hospital**			
**0**	128 (96%)	125 (95%)	253 (95%)
**1**	3 (2%)	1 (1%)	4 (2%)
**2**	1 (1%)	4 (3%)	5 (2%)
**3**	0 (0%)	1 (1%)	1 (0%)
**7**	1 (1%)	1 (1%)	2 (1%)
**Number of antibiotics received during hospital admission**			
**0**	131 (98%)	126 (95%)	257 (97%)
**1**	2 (2%)	6 (5%)	8 (3%)
**Number of other drugs received during hospital admission**			
**0**	129 (97%)	126 (95%)	255 (96%)
**1**	1 (1%)	0 (0%)	1 (0%)
**2**	2 (2%)	2 (2%)	4 (2%)
**3**	1 (1%)	4 (3%)	5 (2%)
**Number of X-rays during hospital admission**			
**0**	132 (99%)	130 (98%)	262 (99%)
**1**	1 (1%)	2 (2%)	3 (1%)
**Number of blood tests during hospital admission**			
**0**	133 (100%)	130 (98%)	263 (99%)
**1**	0 (0%)	2 (2%)	2 (1%)
**Number of emergency department visits****			
**0**	129 (97%)	128 (97%)	257 (97%)
**1**	4 (3%)	4 (3%)	8 (3%)

Note: The summaries encompass the entire 28-day follow-up period, and may refer to more than one re-consultation episode.
**These summaries only include re-consultations that were not reported elsewhere in the form of hospital or emergency department admissions*
***Other interventions included blood and/or urine samples*
*** Participants who were reported to have been referred to the hospital team/ emergency department for acute admission during a re-consultation for clinical deterioration for the same illness episode for which the child was randomised, but for whom no hospital admission is recorded at this date, were classed as having visited emergency department. Hospital admission episodes for at least one night were collected during the notes review.*
The trial did not report any admissions to intensive care units (ICU).

For peer review only - http://bmjopen.bmj.com/site/about/guidelines.xhtml

Table 4: Overview of costs for re-consultations and hospital admissions related to index influenza-like illness episode[+]

	Co-amoxiclav: Cost in £ (N = 133)				Placebo: Cost in £ (N = 132)				Difference* in cost in £ (95% CI) N = 265	
Items	Mean (S.D.)	Median	IQR	Range	Mean (S.D.)	Median	IQR	Range	Mean difference (95% CI)	p-value
Intervention	5 (1)	4	4, 6	4, 6	0 (0)	0	0, 0	0, 0	5 (95% CI 4, 5)	<0.001
Re-consultations	16 (46)	0	0, 0	0, 201	16 (54)	0	0, 0	0, 402	1 (95% CI -9, 10)	0.910
Antibiotics at re-consultation	0 (1)	0	0, 0	0, 10	0 (1)	0	0, 0	0, 5	0 (95% CI 0, 0)	0.920
Other drugs at re-consultation	0 (2)	0	0, 0	0, 13	1 (4)	0	0, 0	0, 34	0 (95% CI -1, 1)	0.593
X-rays at re-consultation	7 (40)	0	0, 0	0, 231	2 (20)	0	0, 0	0, 231	5 (95% CI -2, 13)	0.178
Other interventions at re-consultation	0 (0)	0	0, 0	0, 4	0 (0)	0	0, 0	0, 0	0 (95% CI 0, 0)	0.210
Hospital stay	59 (421)	0	0, 0	0, 4564	94 (484)	0	0, 0	0, 4564	-34 (95% CI -118, 51)	0.432
Antibiotics during hospital stay	0 (2)	0	0, 0	0, 21	0 (0)	0	0, 0	0, 3	0 (95% CI 0, 1)	0.247
Other drugs during hospital stay	0 (1)	0	0, 0	0, 13	1 (3)	0	0, 0	0, 34	0 (95% CI -1, 0)	0.283
X-rays in hospital	2 (20)	0	0, 0	0, 231	4 (28)	0	0, 0	0, 231	-2 (95% CI -8, 5)	0.596
Other interventions in hospital	0 (0)	0	0, 0	0, 0	0 (0)	0	0, 0	0, 1	0 (95% CI 0, 0)	0.168
Emergency department	5 (1)	0	0, 0	0, 160	5 (28)	0	0, 0	0, 160	0 (95% CI -6, 6)	0.983
**Total cost**	94 (480)	4	4, 6	4, 5258	122 (539)	0	0, 0	0, 5177	-25 (95% CI -113, 62)	0.566
Total primary care costs	8 (29)	0	0, 0	0, 300	4 (12)	0	0, 0	0, 63	3 (95% CI -2, 8)	0.215
Total secondary care costs	82 (481)	0	0, 0	0, 5254	117 (540)	0	0, 0	0, 5177	-33 (95% CI -120, 54)	0.453

**Differences have been adjusted for the minimisation factors age (used as continuous variable) and seasonal influenza vaccination status. Clustering by centre has been accounted for using the 'cluster' option in Stata's 'regress' command and robust standard errors were generated.*

CI – confidence interval; IQR - Interquartile range; SD – standard deviation

[+]related to the illness episode for which the child was randomised into ARCHIE over the 28-day follow-up period

For peer review only - http://bmjopen.bmj.com/site/about/guidelines.xhtml

Note: re-consultations and related costs were attributed to primary care costs for children who had been recruited in a GP setting, and to secondary care costs for children who had been recruited in a paediatric ambulatory assessment unit. The primary and secondary care costs shown relate to the trial follow-up period, and do not include the trial intervention.

For peer review only

For peer review only - http://bmjopen.bmj.com/site/about/guidelines.xhtml

Subgroup analysis

Figure 1 shows the differences in costs between the treatment arms by whether or not influenza was confirmed through nasal swabs taken at baseline. Two hundred and sixty-two children for whom the results of nasal swabs were available are included in these summaries. Of these, 21 participants in the co-amoxiclav group (15%), and 16 participants in the placebo group (12%) had laboratory-confirmed influenza. There was a trend towards lower costs in the co-amoxiclav arm for children with laboratory-confirmed influenza, though these differences were not statistically significant.

Extrapolation of findings to national scale

According to the Office for National Statistics, in mid-2018[18], there were approximately 9.7 million children aged 6 months to 12 years in the UK. Information from the 'Surveillance of influenza and other respiratory viruses in the UK' indicates that maximum weekly rates of GP consultations for ILI in 2018/2019 were around 21 per 100,000 for children aged <1 years, 25 per 100,000 for children aged 1-4 years and 19 per 100,000 for children aged 5 to 14[19].

We assume approximately 21 per 100,000 GP consultations were for influenza-like symptoms over an 8-week period in children. Weekly GP consultations for the remaining 44 weeks of the year were assumed to be 5 per 100,000.

Finally, Lee et al[20] estimate that 15.3% of children with influenza like symptoms suffer from at least one of the underlying conditions that were used to classify children as 'at risk', and hence eligible for the ARCHIE trial.

This means that UK-wide, there were around 37,821 GP consultations for influenza like symptoms, 15.3% (5,787) of which would have been expected to occur in the 'at risk' groups included in the ARCHIE study. Using the estimated cost difference between the treatment arms of -£25 (95% CI -113, 62), the potential cost saving of early co-amoxiclav treatment

For peer review only - http://bmjopen.bmj.com/site/about/guidelines.xhtml

compared with not providing early antibiotics is estimated as £-144,675 (95% CI £-358,794 to £653,931).

Discussion

In this work, we have summarised the health-related quality of life, healthcare use and costs associated with treating 'at risk' children with ILI with co-amoxiclav versus placebo.

Our findings, which present within-trial comparisons of costs and outcomes separately, are an important addition to the literature, due to the paucity of observational data and no previous clinical trials performed in this cohort of 'at risk' children. Due to the low completion rate of the EQ-5D-Y, we did not perform a full cost-effectiveness analysis. Data on school/ nursery attendance and childcare arrangements were also subject to low completion rates, and prevented a more robust assessment form a societal perspective.

The low response rates for the EQ-5D-Y and CARIFS data demonstrates the difficulties in assessing health-related quality of life in this patient population. Low questionnaire return rates are a common limitation of paediatric trials; for example, return rates at 14 days from two trials in respiratory tract infections were reported as 41%[21]. In our study, there was anecdotal evidence of parents and carers struggling to complete the questionnaires, especially the EQ-5D-Y questions on walking, usual activities and dressing, if the items were not appropriate to the expected capabilities of their children, either because of their age, especially for very young children, or their underlying condition or disability. This was reflected in the lower proxy completion rates of the EQ-5D-Y for children below the age of two years, compared to those aged 2 years and over, and the fact that the VAS scales of the EQ-5D-Y and the CARIFS had higher completion rates than the Likert-style questions of the questionnaires, as shown in the Supplemental Material. Generally, there is a need for validated questionnaires that can be completed easily in this patient population.

For peer review only - http://bmjopen.bmj.com/site/about/guidelines.xhtml

The EQ-5D-Y was expected to be completed by proxy, i.e. by the children's parents or carers on their behalf, and/or by the children themselves, as appropriate. There appeared to be some overlap between the answers provided on the proxy and self-completed EQ-5D-Y questionnaires, particularly for very young children. It is possible that some parents or carers completed the questionnaires supposed to be filled in by the children. All EQ-5D-Y data were analysed as received, without exclusions.

The proxy-completed EQ-5D-Y values were not statistically significantly different between the two groups at any of the time points, although there was an improvement in health-related quality of life (HRQOL) as indicated by the EQ-5D-Y scores over time (day 0 to day 28) for both trial arms. There was a trend towards lower EQ-5D-Y values, indicating worse quality of life, in the co-amoxiclav arm on days 4, 14 and 28. The CARIFS score in the co-amoxiclav arm at seven days was significantly lower than the score in the placebo arm, indicating less severe symptoms. This might suggest that the CARIFS score is more sensitive to subtle disease specific changes in children's health than the EQ-5D-Y. Interestingly, the EQ-5D VAS also showed a statistically significant benefit of co-amoxiclav at seven days, and the EQ-5D-Y values were higher (not statistically significant) in the co-amoxiclav arm at seven days compared with the placebo arm, indicating higher HRQOL. Studies of common cold or non-specific respiratory tract infections in general paediatric populations suggest that symptoms in 50% of children had improved by day seven to eight, and for 80% by day 14[22]. It may therefore be that any differences between groups will be more apparent at a stage in the illness where more children are likely to still be symptomatic than later on, when the majority will have resolved due to the natural course of the illness. Both questionnaires are subject to high levels of missing data, and should be interpreted cautiously. The high rates of missing data and anecdotal evidence of parents' and carers' difficulties of completing the questionnaires demonstrate a need for additional validated tools to reliably measure quality of life in this patient population.

For peer review only

For peer review only - http://bmjopen.bmj.com/site/about/guidelines.xhtml

Overall, only 5% of children were admitted to hospital for reasons related to the illness episode for which the child was randomised into ARCHIE over the 28-day follow-up period. Admissions were often only for one or two nights. Three percent of participants reported one emergency department visit each. Fewer than 25% of all randomised children had a re-consultation. This was lower than the anticipated 40% re-consultation rate based on published data available at the time the study was designed[1], but still significantly higher than the 4% observed in a primary care cohort of children with acute respiratory tract infection who did not have known risk factors for complications from influenza or ILI[23]. As such, the use of primary and secondary care facilities during the trial follow-up for reasons related to the initial illness episode for which the child was randomised into ARCHIE was generally low and the limited related hospital admissions were the main cost drivers for the average cost per randomised child. In the co-amoxiclav arm, costs related to hospital admissions and emergency department visits accounted for 70% of total costs; in the placebo arm, this was 85%.

Fourteen per cent of the study population had laboratory-confirmed influenza. The subgroup analysis indicated that there was a trend that the early co-amoxiclav treatment may be more cost-saving in participants with laboratory-confirmed influenza, although this subgroup does not have sufficient statistical power to draw definitive conclusions. Also, this trial assessed the benefit of treating at risk children as early as possible after the onset of ILI. While there is a possibility that treatment may be more beneficial in those with laboratory-confirmed influenza, evaluating the nasal swabs takes time, and may make it impossible to treat these children early. Nasal swabs were costed at £8 per child, and all children presenting with ILI would need to be tested. This outweighs the costs of co-amoxiclav, which was costed at £4.13 for children below 7 year of age, and at £5.79 children above 7 years of age. As such, there is no advantage in terms of costs to confirm influenza prior to commencing of treatment outside influenza pandemic periods. Based on the findings of the subgroup analysis, early co-amoxiclav treatment without prior nasal swab testing may be cost-effective during periods of

For peer review only - http://bmjopen.bmj.com/site/about/guidelines.xhtml

high influenza activity, such as influenza pandemics. However, further trials conducted during such periods would be needed to confirm this.

Finally, in line with the study protocol, we aimed to extrapolate the results of the study to a national level. With regards to the ARCHIE trial, this extrapolation has a number of limitations. Firstly, the difference in costs between the co-amoxiclav and placebo was not statistically significant. Therefore, it is unclear if extrapolation to a national level would result in a cost-saving or increase in spending. Finally, influenza is an infectious disease, and rates of infections vary greatly between epidemics. Our current extrapolation uses GP consultations for ILI in 2018/2019. However, it is unclear how many children may be affected in the upcoming flu seasons. This is particularly important as the rates of influenza were low during the recruitment period of ARCHIE[24-27], but more children may be affected in coming years. Our extrapolations were based on recent data, but may have to be revised in the light of SARS-CoV-2, and its impact on influenza and ILI in future influenza seasons.

Conclusions

Our findings did not show evidence that early treatment with co-amoxiclav in 'at risk' children with influenza-like illness improves quality of life, reduces healthcare use or increases cost savings over 28 days. However, early co-amoxiclav treatment was associated with decreased symptom severity at day 7. Collection of proxy-reported data was challenging in this patient population. Suitable validated quality of life instruments are therefore needed to support more robust evaluations in this group.

List of abbreviations

CARIFS: Canadian Acute Respiratory Infection and Flu Scale

CI: Confidence Interval

HRQL: Health-Related Quality of Life

For peer review only - http://bmjopen.bmj.com/site/about/guidelines.xhtml

ICU: Intensive Care Unit

ILI: Influenza-Like Illness

IQR: Interquartile Range

QALYs: Quality Adjusted Life Years

SD: Standard Deviation

VAS: Visual Analogue Scale

Acknowledgements

The research team acknowledges the support of the National Institute for Health Research Clinical Research Network (NIHR CRN). We would like to thank the ARCHIE Investigators for their support throughout the study, in particular: Malcolm G Semple PhD, Michael Moore FRCGP, Alastair D Hay FRCGP, Ushma Galal MSc, Tricia Taffe Carver BS, Rafael Perera-Salazar DPhil, Ly-Mee Yu DPhil, Jill Mollison PhD, Paul Little FRCGP, Andrew Farmer DM and Christopher Butler FMedSci.

We thank Mandy Wan (Paediatric Research Pharmacist, NIHR CRN Children speciality) for her support and guidance regarding trial medication, from procurement to disposal. Staff in Oxford University's Primary Care Clinical Trials Unit were responsible for randomisation software design (David Judge), database development (Sadie Kelly), trial co-ordination (Sonya Beecher) and patient recruitment (Heather Rutter, Karen Madronal, Irene Noel, Luisa Saldana Ortega, Pippa Whitbread, Bernadette Mundy, Belinda I'Anson, Samantha Spires). We thank our Programme Steering Committee (Professor Willie Hamilton, Professor Judy Breuer, Professor Maureen Baker, Robert Newby), and Data and Safety Monitoring Committee (Professor Lorcan McGarvey, Mike Bradburn, Professor Surinder Birring, Professor Nick Francis), and our regional trial coordinators (Lyn Liddiard, Tammy Thomas, Clare McIntyre) for their valuable advice and support with delivering the trial. We thank Mildred Foster and Dan Richards-Doran for developing our study website and other study-related publicity materials. We also thank Dr Oliver van Hecke for his assistance with monitoring data

For peer review only - http://bmjopen.bmj.com/site/about/guidelines.xhtml

re-consultation data extracted from medical records. Last but not least, we thank all the health care professionals, general practice and hospital staff, children and parents/guardians who participated in our study.

Funding statement

This project was funded by the National Institute for Health Research (NIHR) Programme Grants for Applied Research Programme (project reference RP-PG-1210-12012). The views expressed are those of the authors and not necessarily those of the NIHR or the Department of Health and Social Care. The funder of the study had no role in design and conduct of the study; collection, management, analysis, and interpretation of the data; preparation, review, or approval of the manuscript; and decision to submit the manuscript for publication.

Competing interests

KW held a National Institute for Health Research (NIHR) Academic Clinical Lectureship during the conduct of the study and currently holds a NIHR Postdoctoral Fellowship.

The other authors declare that they have no competing interests.

Author contributions

Kay Wang was Chief Investigator of the trial until going on maternity leave in September 2018. She reviewed clinical data on outcome events to guide collation and analysis of data for this study.

Sharon Tonner was Trial Manager of the trial from 2017 until its conclusion. She managed the day to day data collection activities.

Jenna Grabey was the data manager on the trial from January 2018 until its conclusion.

For peer review only - http://bmjopen.bmj.com/site/about/guidelines.xhtml

For peer review only

Anthony Harnden was Chief Investigator on the trial from September 2018 until its conclusion. He reviewed clinical data on outcome events to guide collation and analysis of data for this study.

Jane Wolstenholme was the senior health economist on the trial. She developed the economic evaluation plan, and led the economic analysis of the trial findings.

Ines Rombach conducted the analyses for this paper.

Ines Rombach and Jane Wolstenholme drafted the first version of the manuscript.

All authors reviewed the manuscript, contributed comments and edits, and approved the final version.

Collaborators

ARCHIE (The early use of antibiotics for At-Risk CHildren with InfluEnza in primary care) investigators: Professor Anthony Harnden (Nuffield Department of Primary Care Health Sciences, University of Oxford, Oxford, UK), Dr Kay Wang (Nuffield Department of Primary Care Health Sciences, University of Oxford, Oxford, UK), Professor Malcolm G Semple (National Institute for Health Research (NIHR) Health Protection Research Unit in Emerging and Zoonotic Infections, University of Liverpool, Liverpool, UK), Dr Jane Wolstenholme (Health Economics Research Centre, University of Oxford, Oxford, UK), Professor Rafael Perera-Salazar (Nuffield Department of Primary Care Health Sciences, University of Oxford, Oxford, UK), Dr Ly-Mee Yu (Nuffield Department of Primary Care Health Sciences, University of Oxford, Oxford, UK), Professor Alastair D Hay (Centre for Academic Primary Care, School of Social and Community Medicine, University of Bristol, Bristol, UK), Professor Paul Little (Primary Care Population Sciences and Medical Education, University of Southampton, Southampton, UK), Professor Michael Moore (Primary Care Population Sciences and Medical Education, University of Southampton, Southampton, UK), Professor Chris Butler (Nuffield

For peer review only - http://bmjopen.bmj.com/site/about/guidelines.xhtml

Department of Primary Care Health Sciences, University of Oxford, Oxford, UK), Tricia Taffe Carver (Nuffield Department of Primary Care Health Sciences, University of Oxford, Oxford, UK).

Ethical approval

The ARCHIE trial has been reviewed and approved by the North West-Liverpool East Research Ethics Committee, Health Research Authority and Medicines and Healthcare Products Regulatory Agency. Full written informed consent was obtained from a parent or guardian for each study participant. Children were also invited to give written assent if appropriate.

Data sharing:

The trial protocol and de-identified participant level data collected for the trial are available on request. Research data requests should be submitted to the corresponding author for consideration by the research team.

For peer review only - http://bmjopen.bmj.com/site/about/guidelines.xhtml

List of Figures

Figure 1: Forest plot for differences in costs by laboratory-confirmed influenza (in £)

**Subgroup effects were obtained by adding an interaction term between treatment allocation and laboratory-confirmed influenza and have been adjusted for the stratification factors age (used as continuous variable) and seasonal influenza vaccination status. Clustering by centre has been accounted for using the 'cluster' option in Stata's 'regress' command and robust standard errors were generated.*

For peer review only

For peer review only - http://bmjopen.bmj.com/site/about/guidelines.xhtml

References

1. Wang K, Carver T, Tonner S, et al. Early use of Antibiotics for at Risk CHildren with InfluEnza (ARCHIE): protocol for a double-blind, randomised, placebo-controlled trial. *BMJ Open* 2018; **8**(5): e021144.
2. Burch J, Paulden M, Conti S, et al. Antiviral drugs for the treatment of influenza: a systematic review and economic evaluation. *Health Technol Assess* 2009; **13**(58): 1-265, iii-iv.
3. Tappenden P, Jackson R, Cooper K, et al. Amantadine, oseltamivir and zanamivir for the prophylaxis of influenza (including a review of existing guidance no. 67): a systematic review and economic evaluation. *Health Technol Assess* 2009; **13**(11): iii, ix-xii, 1-246.
4. Turner D, Wailoo A, Nicholson K, Cooper N, Sutton A, Abrams K. Systematic review and economic decision modelling for the prevention and treatment of influenza A and B. *Health Technol Assess* 2003; **7**(35): iii-iv, xi-xiii, 1-170.
5. Nicholson KG, Aoki FY, Osterhaus AD, et al. Efficacy and safety of oseltamivir in treatment of acute influenza: a randomised controlled trial. Neuraminidase Inhibitor Flu Treatment Investigator Group. *Lancet* 2000; **355**(9218): 1845-50.
6. Treanor JJ, Hayden FG, Vrooman PS, et al. Efficacy and safety of the oral neuraminidase inhibitor oseltamivir in treating acute influenza: a randomized controlled trial. US Oral Neuraminidase Study Group. *JAMA* 2000; **283**(8): 1016-24.
7. Williamson I, Benge S, Barton S, et al. A double-blind randomised placebo-controlled trial of topical intranasal corticosteroids in 4- to 11-year-old children with persistent bilateral otitis media with effusion in primary care. *Health Technol Assess* 2009; **13**(37): 1-144.
8. Wolstenholme JL, Bargo D, Wang K, Harnden A, Raisanen U, Abel L. Preference-based measures to obtain health state utility values for use in economic evaluations with child-based populations: a review and UK-based focus group assessment of patient and parent choices. *Qual Life Res* 2018; **27**(7): 1769-80.
9. Kreimeier S, Oppe M, Ramos-Goni JM, et al. Valuation of EuroQol Five-Dimensional Questionnaire, Youth Version (EQ-5D-Y) and EuroQol Five-Dimensional Questionnaire, Three-Level Version (EQ-5D-3L) Health States: The Impact of Wording and Perspective. *Value Health* 2018; **21**(11): 1291-8.
10. Brooks R. EuroQol: the current state of play. *Health Policy* 1996; **37**(1): 53-72.
11. EuroQol G. EuroQol--a new facility for the measurement of health-related quality of life. *Health Policy* 1990; **16**(3): 199-208.
12. Rabin R, de Charro F. EQ-5D: a measure of health status from the EuroQol Group. *Ann Med* 2001; **33**(5): 337-43.
13. Jacobs B, Young NL, Dick PT, et al. Canadian Acute Respiratory Illness and Flu Scale (CARIFS): development of a valid measure for childhood respiratory infections. *J Clin Epidemiol* 2000; **53**(8): 793-9.
14. Personal Social Services Research Unit (PSSRU). Unit Costs of Health and Social Care 2018. 23/05/2019). https://www.pssru.ac.uk/project-pages/unit-costs/unit-costs-2018/.
15. NHS Improvement. Reference Cost Collection: National Schedule of Reference Costs - Year 2017-18. https://improvement.nhs.uk/resources/reference-costs/#rc1718 (accessed 23/05 2019).
16. Fenwick EA, Briggs AH, Hawke CI. Management of urinary tract infection in general practice: a cost-effectiveness analysis. *Br J Gen Pract* 2000; **50**(457): 635-9.
17. Paediatric Formulary Committee. BNF for Children (online) London: BMJ Group, Pharmaceutical Press, and RCPCH Publications. https://www.medicinescomplete.com/ (accessed 23 November 2019).
18. Office for National Statistics. Dataset: Estimates of the population for the UK, England and Wales, Scotland and Northern Ireland. 2019. https://www.ons.gov.uk/peoplepopulationandcommunity/populationandmigration/populationesti

For peer review only - http://bmjopen.bmj.com/site/about/guidelines.xhtml

mates/datasets/populationestimatesforukenglandandwalesscotlandandnorthernireland (accessed 05/12/ 2019).
19. Public Health England. Surveillance of influenza and other respiratory viruses in the UK: Winter 2018 to 2019. *Influenza Surveillance Team, Immunisation and Countermeasures Division, National Infection Service, PHE,* 2019.
20. Lee JJ, Bankhead C, Smith M, Kousoulis AA, Butler CC, Wang K. Risk factors for influenza-related complications in children during the 2009/10 pandemic: a UK primary care cohort study using linked routinely collected data. *Epidemiol Infect* 2018; **146**(7): 817-23.
21. Schot MJC, Dekker ARJ, van Werkhoven CH, et al. Burden of disease in children with respiratory tract infections in primary care: diary-based cohort study. *Fam Pract* 2019; **36**(6): 723-9.
22. Thompson M, Vodicka TA, Blair PS, et al. Duration of symptoms of respiratory tract infections in children: systematic review. *BMJ* 2013; **347**: f7027.
23. Redmond NM, Turnbull S, Stuart B, et al. Impact of antibiotics for children presenting to general practice with cough on adverse outcomes: secondary analysis from a multicentre prospective cohort study. *Br J Gen Pract* 2018; **68**(675): e682-e93.
24. Public Health England. Surveillance of influenza and other respiratory viruses in the United Kingdom: winter 2014 to 2015. https://assets.publishing.service.gov.uk/government/uploads/system/uploads/attachment_data/file/429617/Annualreport_March2015_ver4.pdf (accessed 26th May 2020).
25. Public Health England. Surveillance of influenza and other respiratory viruses in the United Kingdom: Winter 2015 to 2016. https://assets.publishing.service.gov.uk/government/uploads/system/uploads/attachment_data/file/526405/Flu_Annual_Report_2015_2016.pdf (accessed 26th May 2020).
26. Public Health England. Surveillance of influenza and other respiratory viruses in the UK: Winter 2016 to 2017. https://assets.publishing.service.gov.uk/government/uploads/system/uploads/attachment_data/file/613493/Surveillance_of_influenza_and_other_respiratory_viruses_in_the_UK_2016_to_2017.pdf (accessed 26th May 2020).
27. Public Health England. Surveillance of influenza and other respiratory viruses in the UK: Winter 2017 to 2018. https://assets.publishing.service.gov.uk/government/uploads/system/uploads/attachment_data/file/740606/Surveillance_of_influenza_and_other_respiratory_viruses_in_the_UK_2017_to_2018.pdf (accessed 26th May 2020).

For peer review only

For peer review only - http://bmjopen.bmj.com/site/about/guidelines.xhtml

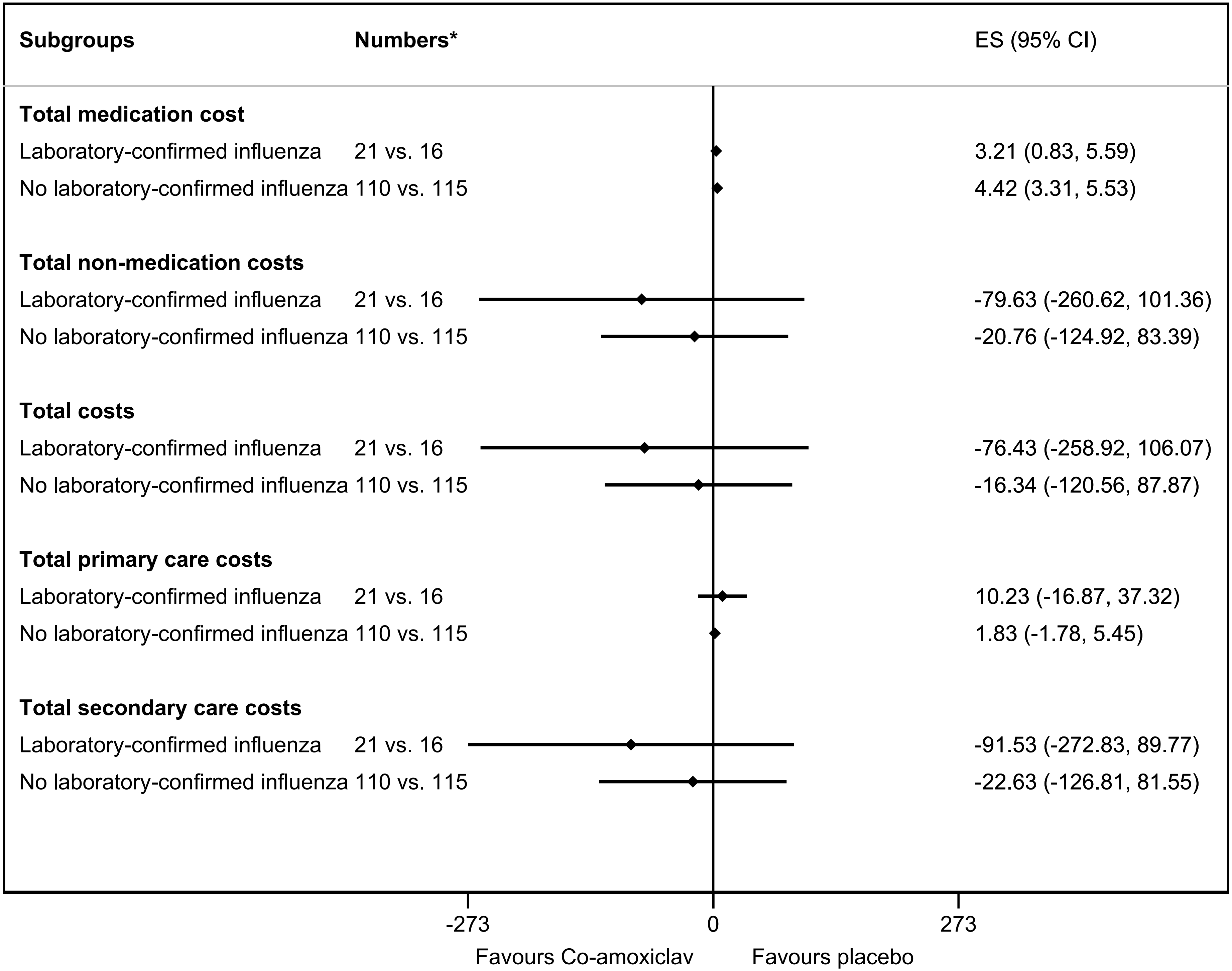

* n in active vs. n in placebo arm
ES - effect size

Supplemental material for:

Quality of life, healthcare use and costs in 'at risk' children after early antibiotic treatment vs. placebo for for influenza-like illness: Within-trial economic analyses of the ARCHIE randomised controlled trial

Authors

Ines Rombach (ines.rombach@ndph.ox.ac.uk)[1],
Kay Wang (kay.wang@phc.ox.ac.uk)[2],
Sharon Tonner (sharon.tonner@phc.ox.ac.uk)[2],
Jenna Grabey (jenna.grabey@phc.ox.ac.uk)[2],
Anthony Harnden (anthony.harnden@phc.ox.ac.uk)[2],
Jane L Wolstenholme (jane.wolstenholme@ndph.ox.ac.uk)[1],
ARCHIE Investigators

Affiliations

[1]Health Economics Research Centre, Nuffield Department of Population Health, Oxford University, Oxford, UK

[2]Nuffield Department of Primary Care Health Sciences, University of Oxford, Oxford, UK

Corresponding author:
Ines Rombach
Health Economics Research Centre
Nuffield Department of Population Health
Old Road Campus
University of Oxford
Oxford
OX3 7LF
United Kingdom
ines.rombach@ndorms.ox.ac.uk
https://orcid.org/0000-0003-3464-3867

Published in BMJ Open

For peer review only - http://bmjopen.bmj.com/site/about/guidelines.xhtml

Table of Contents

Supplemental File 1: Unit costs 3

Supplemental Table S1: Unit costs 3

Supplemental File 2: Availability of data reported on behalf of the children, and by the children 6

Supplemental Table S2a: Available EQ-5D-Y data for participants aged less than 2 years 6

Supplemental Table S2b: Available EQ-5D-Y data for participants aged 2+ years 8

Supplemental Table S2c: Missing data pattern over time for the EQ-5D-Y proxy, completed on behalf of the child 9

Supplemental Table S2d: Proxy-reported EQ-5D-Y scores by data availability at the subsequent follow-up 9

Supplemental Table S2e: Missing data pattern for the EQ-5D-Y domains (proxy data, completed on behalf of the child) 10

Supplemental Table S2f: Available EQ-5D-Y items 11

Supplemental Table S2g: Available EQ-5D-Y items for children aged less than 2 years 12

Supplemental Table S2h: Available EQ-5D-Y items for children aged 2+ years (proxy) 13

Supplemental File 3: Reponses to the EQ-5D-Y items 14

Supplemental Table S3a: Responses to EQ-5D-Y domains (proxy, completed on behalf of child) 15

Supplemental Table S3b: Responses to EQ-5D-Y domains (proxy, completed on behalf of child for children aged less than 2 years) 16

Supplemental Table S3c: Responses to EQ-5D-Y domains (proxy, completed on behalf of child for children aged 2+ years) 17

Supplemental Table S3d: Responses to EQ-5D-Y domains (completed by child) 18

Supplemental Table S3e: Responses to EQ-5D-Y domains (completed by child for children aged less than 2 years) 19

Supplemental Table S3f: Responses to EQ-5D-Y domains (completed by child for children aged 2+ years) 20

Supplemental Table 3g: Responses to CARIFS domains (only including questionnaires with sufficient data to calculate the overall CARIFS score) 21

Supplemental Table 3h: Responses to CARIFS domains for children less than 2 years old (only including questionnaires with sufficient data to calculate the overall CARIFS score) 24

Supplemental Table 3i: Responses to CARIFS domains for children aged 2+ years (only including questionnaires with sufficient data to calculate the overall CARIFS score) 26

Supplemental File 4: Additional summaries of research use 29

Supplemental Table 4a: Details of all reported hospital admissions 29

Supplemental Table 4b: Overview of healthcare use for re-consultations due to clinical deterioration and hospitalisations related to the illness episode for which the child was randomised to ARCHIE 30

Supplemental File 5: Number of days children were unable to attend school or nursery, and subsequent changes to childcare requirements 31

Supplemental Table 5: Daily activity and child care 31

For peer review only

Supplemental File 1: Unit costs

Supplemental Table S1: Unit costs

Cost item	Cost	Data Source
Re-consultations primary care (GP)	£31	Personal Social Services Research Unit (PSSRU), online unit cost database of health and social care professionals 2017/2018: GP including direct care, cost per surgery lasting 9.22 minutes.
Re-consultation in paediatric ambulatory assessment units	£201	Weighted average of consultant-led and non-consultant led non-admitted face-to-face follow-up attendance for paediatric respiratory medicine; National Schedule of reference costs 2017/2018, CL and NCL tab.
Chest x-ray	£231	Outpatient procedures, paediatric same-day diagnostic imaging; National Schedule of reference costs 2017/2018, OPROC tab. (Note: the same cost was applied to chest x-rays at re-consultation and during hospital admission, as it was assumed that x-rays for the ARCHIE participants would have to be performed on the same day)
Night in hospital	£652	Weighted average of cost per day for non-elective paediatric hospital stays; National Schedule of reference costs 2017/2018, NEL, NEL_XS and NES tab.
Emergency department	£160	Weighted average of all emergency department attendances excluding dental care and patient dead on arrival, National Schedule of reference costs 2017/2018, AE tab.
**Day case**	£610	
**Investigations performed**		
Blood test	£1	Cost of clinical biochemistry, National Schedule of reference costs 2017/2018, DAPS tab.
Nasal aspirate/ npa	£8	Cost of microbiological diagnostic test, National Schedule of reference costs 2017/2018, DAPS tab.
Throat swab	£8	Cost of microbiological diagnostic test, National Schedule of reference costs 2017/2018, DAPS tab.
Urine test	£2.80	Dipstick £0.20 + Laboratory culture £2.60, Fenwick et al[10]

For peer review only - http://bmjopen.bmj.com/site/about/guidelines.xhtml

Antibiotics and other drugs prescribed		Costed in line with British National Formulary for Children – accessed 22Aug2019.<br>The minimum Drug Tariff is used for all costs.
**Antibiotics**		
Amoxicillin	£1.26	Assume all amoxicillin given as oral suspension.<br>Amoxicillin (as Amoxicillin trihydrate) 50 mg per 1 ml – 100 ml cost £1.26
Azithromycin	£4.06	Assume all AZITHROMYCIN given as oral suspension.<br>Azithromycin 40 mg per 1 ml – 15 ml cost £4.06
Ceftriaxone	£9.58	By IV. One vial of Ceftriaxone (as Ceftriaxone sodium) 1g. 1 vial £9.58
Chloramphenicol	£1.55	Assume used as eye drops. Cost by vial.<br>Chloramphenicol 5 mg per 1 ml. 10 ml cost £1.55
Clarithromycin	£3.36	Assume oral suspension used, as tablet doses too high.<br>• Clarithromycin 25 mg per 1 ml. 70 ml cost £3.36
**Study intervention: Co-amoxiclav 400/57**	£4.13 children below 7<br>£5.79 children 7+	Amoxicillin (as Amoxicillin trihydrate) 80 mg per 1 ml, Clavulanic acid (as Potassium clavulanate) 11.4 mg per 1 ml<br>35 ml: £4.13, 70 ml: £5.79<br>Note: in the trial, co-amoxiclav 400/57 was provided by Brown & Burk UK Ltd. This was the only company who was able to procure a placebo suitable for use in this study. Recommendations from the study will not be restricted to this manufacturer. Therefore, the costing will also include other manufacturers, whose products are available in 35ml and 70 ml.
Co-amoxiclav	£1.65	Assume oral suspension.<br>Amoxicillin (as Amoxicillin trihydrate) 25 mg per 1 ml, Clavulanic acid (as Potassium clavulanate) 6.25 mg per 1 ml. (lower dose than study drug)<br>➔100ml cost £1.65
Dexamethasone	£21.15	Dexamethasone (as Dexamethasone sodium phosphate) 400 microgram per 1 ml. 150 ml cost £42.30.
Erythromycin ethyl succinate	£5.21	Assume oral suspension.<br>Erythromycin (as Erythromycin ethyl succinate) 25 mg per 1 ml. 100ml cost £5.21
Phenoxymethylpenicillin	£4.28	Assume oral solution.<br>Phenoxymethylpenicillin (as Phenoxymethylpenicillin potassium) 25 mg per 1 ml. 100 ml costs £4.28
**Other drugs**		

For peer review only - http://bmjopen.bmj.com/site/about/guidelines.xhtml

Atrovent	£5.56	Ipratropium bromide 20 microgram per 1 dose. Inhaler with 200 doses costs £5.56
Beclomethasone /unidentified steroids	£3.70	Beclometasone dipropionate 50 microgram per 1 dose. 200 dose inhaler costs £3.70.<br>(this was also used for unidentified steroids)
Dexamethasone	£42.30	Assume oral solution.<br>Dexamethasone (as Dexamethasone sodium phosphate) 400 microgram per 1 ml. 150ml cost £42.30
Fluticasone	£6.53	Fluticasone propionate 25 microgram per 1 dose. 120 dose inhaler costs £6.53
Fluticasone	£21.26	Inhaler is used.<br>Fluticasone propionate 125 microgram per 1 dose. £21.26 for 120 doses.
Ibuprofen	£4.20	Assume oral suspension. Ibuprofen 40 mg per 1 ml. 100 ml cost £4.20 (NHS indicative price, no drug tariff available)
Ipratropium bromide 20 mcg	£5.56	Inhaler: Ipratropium bromide 20 microgram per 1 dose. 200 doses cost £5.56.
Magnesium sulphate	£1.735	Magnesium sulfate heptahydrate 500 mg per 1 ml; 10 ampoule cost £17.35
Oxygen	£0.95	Supplemental oxygen per hour, provided by mask or nasal, was estimated to be £0.80 for NHS Lothian in 2013. Prices have been adjusted to their value in 2018 based on a discount rate of 3.5% per year, as recommended for the UK.
Paracetemol 300 mg	£3.78	Oral suspension paediatrics, 120mg/5ml. 200 ml cost £3.78
Prednisolone 5 mg	£11.41	Oral solution: Prednisolone 10 mg per 1 ml: £11.41 for 10 unit dose
Salbutamol/ ventolin	£1.50	Salbutamol (as Salbutamol sulfate) 100 microgram per 1 dose. 200 doses £1.50
Salmeterol 25 mcg	£29.26	Salmeterol (as Salmeterol xinafoate) 25 microgram per 1 dose; 120 doses £29.26
Seretide	£21.26	Fluticasone – costed as above

For peer review only

Supplemental File 2: Availability of data reported on behalf of the children, and by the children

Availability of EQ-5D-Y data

For the EQ-5D-Y, the versions completed by the proxy on behalf of the children are more complete. The trial team considered substituting by available information from the EQ-5D-Y completed by the children themselves if data were missing from the EQ-5D-Y proxy, to enable a more complete analysis dataset. However, this approach yielded only an additional 1, 2, 3, 4 and 2 observations at days 0, 4, 7, 14 and 28, and was therefore abandoned.

For 54 participants (20%), proxy-completed EQ-5D-Y index data are available at baseline and all follow-up time points.

For peer review only

Supplemental Table S2a: Available EQ-5D-Y data for participants aged less than 2 years

	Co-amoxiclav (N=45)	Placebo (N=44)	Total (N=89)
**Availability of EQ-5D-Y index completed on behalf of child**			
**day 0**	18 (40%)	24 (55%)	42 (47%)
**day 4**	9 (20%)	12 (27%)	21 (24%)
**day 7**	9 (20%)	14 (32%)	23 (26%)
**day 14**	10 (22%)	13 (30%)	23 (26%)
**day 28**	11 (24%)	11 (25%)	22 (25%)
**Availability of EQ-5D VAS index completed by child**			
**day 0**	39 (87%)	43 (98%)	82 (92%)
**day 4**	27 (60%)	29 (66%)	56 (63%)
**day 7**	27 (60%)	28 (64%)	55 (62%)
**day 14**	22 (49%)	24 (55%)	46 (52%)
**day 28**	19 (42%)	19 (43%)	38 (43%)
**Availability of EQ-5D-Y index completed by child**			
**day 0**	0 (0%)	0 (0%)	0 (0%)
**day 4**	0 (0%)	1 (2%)	1 (1%)
**day 7**	0 (0%)	1 (2%)	1 (1%)
**day 14**	0 (0%)	1 (2%)	1 (1%)
**day 28**	0 (0%)	1 (2%)	1 (1%)
**Availability of EQ-5D VAS index completed by child**			
**day 0**	0 (0%)	1 (2%)	1 (1%)
**day 4**	3 (7%)	1 (2%)	4 (4%)
**day 7**	3 (7%)	1 (2%)	4 (4%)
**day 14**	3 (7%)	1 (2%)	4 (4%)
**day 28**	2 (4%)	1 (2%)	3 (3%)

For peer review only - http://bmjopen.bmj.com/site/about/guidelines.xhtml

**Availability of CARIFS**			
**day 0**	27 (60%)	29 (66%)	56 (63%)
**day 7**	21 (47%)	23 (52%)	44 (49%)
**Availability of CARIFS VAS**			
**day 0**	44 (98%)	44 (100%)	88 (99%)
**day 7**	26 (58%)	28 (64%)	54 (61%)

For peer review only

Supplemental Table S2b: Available EQ-5D-Y data for participants aged 2+ years

	Co-amoxiclav (N=91)	Placebo (N=91)	Total (N=182)
**Availability of EQ-5D-Y index completed on behalf of child**			
**day 0**	78 (86%)	84 (92%)	162 (89%)
**day 4**	43 (47%)	40 (44%)	83 (46%)
**day 7**	43 (47%)	40 (44%)	83 (46%)
**day 14**	34 (37%)	33 (36%)	67 (37%)
**day 28**	30 (33%)	20 (22%)	50 (27%)
**Availability of EQ-5D-Y index completed by child**			
**day 0**	81 (89%)	86 (95%)	167 (92%)
**day 4**	50 (55%)	47 (52%)	97 (53%)
**day 7**	51 (56%)	45 (49%)	96 (53%)
**day 14**	39 (43%)	33 (36%)	72 (40%)
**day 28**	34 (37%)	21 (23%)	55 (30%)
**Availability of EQ-5D-Y index completed by child**			
**day 0**	32 (35%)	29 (32%)	61 (34%)
**day 4**	16 (18%)	12 (13%)	28 (15%)
**day 7**	17 (19%)	10 (11%)	27 (15%)
**day 14**	17 (19%)	9 (10%)	26 (14%)
**day 28**	17 (19%)	9 (10%)	26 (14%)
**Availability of EQ-5D VAS index completed by child**			
**day 0**	28 (31%)	24 (26%)	52 (29%)
**day 4**	16 (18%)	12 (13%)	28 (15%)
**day 7**	16 (18%)	10 (11%)	26 (14%)
**day 14**	17 (19%)	9 (10%)	26 (14%)
**day 28**	16 (18%)	10 (11%)	26 (14%)
**Availability of CARIFS**			
**day 0**	72 (79%)	68 (75%)	140 (77%)
**day 7**	45 (49%)	34 (37%)	79 (43%)
**Availability of CARIFS VAS**			
**day 0**	89 (98%)	91 (100%)	180 (99%)
**day 7**	50 (55%)	46 (51%)	96 (53%)

For peer review only

For peer review only - http://bmjopen.bmj.com/site/about/guidelines.xhtml

Supplemental Table S2c: Missing data pattern over time for the EQ-5D-Y proxy, completed on behalf of the child

Missing data pattern*	N	%
+....	104	38.4%
+++++	54	19.9%
.....	48	17.7%
++++.	19	7.0%
+++..	19	7.0%
.++++	5	1.8%
...++	3	1.1%
..+..	3	1.1%
....+	3	1.1%
+++.+	2	0.7%
+..++	2	0.7%
.+++.	2	0.7%
...+.	2	0.7%
+.+++	1	0.4%
++.+.	1	0.4%
++..+	1	0.4%
..+++	1	0.4%
++...	1	0.4%

*****+ indicates data are available, . indicates data are missing. The first character refers to the baseline EQ-5D-Y, the second to the 4 day assessment etc.*
For example, the first line of the table (+….) describes participants for whom baseline data, but no data for the subsequent four follow-up visits are available.

Supplemental Table S2d: Proxy-reported EQ-5D-Y scores by data availability at the subsequent follow-up

This table shows EQ-5D-Y values (as reported by their proxies on the children's behalf) at each time point spit by whether or not data was available for the subsequent time point. We hypothesised that those with unavailable data at the subsequent time point would be likely to have worth health states.

There was no consistent pattern to support this hypothesis.

	Participants for whom subsequent follow-up is available	Participants for whom subsequent follow-up is not available
**Day 0**	0.549 (0.396), N=97	0.606 (0.335), N=107
**Day 4**	0.671 (0.318), N=101	0.286 (0.671), N=3
**Day 7**	0.780 (0.339), N=82	0.759 (0.394), N=24
**Day 14**	0.882 (0.280), N=66	0.888 (0.217), N=24

For peer review only - http://bmjopen.bmj.com/site/about/guidelines.xhtml

Supplemental Table S2e: Missing data pattern for the EQ-5D-Y domains (proxy data, completed on behalf of the child)

	**Baseline**		**Day 4**		**Day 7**		**Day 14**		**Day 28**	
	Frequency	%	Frequency	%	Frequency	%	Frequency	%	Frequency	%
+++++	204	75.3%	104	38.4%	106	39.1%	90	33.2%	72	26.6%
.....	5	1.8%	117	43.2%	122	45%	153	56.5%	179	66.1%
+.+++	38	14%	25	9.2%	21	7.7%	13	4.8%	8	3%
..+++	15	5.5%	16	5.9%	16	5.9%	13	4.8%	9	3.3%
.++++	2	0.7%	3	1.1%	2	0.7%	1	0.4%	1	0.4%
+.++.	2	0.7%	1	0.4%	-	-	-	-	-	-
...++	2	0.7%	3	1.1%	-	-	-	-	-	-
++++.	1	0.4%	-	-	-	-	-	-	-	-
..++.	1	0.4%	-	-	-	-	-	-	-	-
...+.	1	0.4%	-	-	-	-	-	-	-	-
+++..	-	-	1	0.4%	-	-	-	-	-	-
+..++	-	-	1	0.4%	-	-	-	-	-	-
+.+.+	-	-	-	-	1	0.4%	-	-	-	-
+...+	-	-	-	-	1	0.4%	-	-	-	-
...++	-	-	-	-	1	0.4%	1	0.4%	-	-
....+	-	-	-	-	1	0.4%	-	-	-	-
++++.	-	-	-	-	-	-	-	-	2	0.7%

**+* indicates data are available, . indicates data are missing. The first character refers to the first EQ-5D item (mobility), the second to the second question (self-care) etc. The pattern +.+++ indicates that the answer to the self-care question is missing, while all other responses are available.*

Particularly the items on self-care and mobility are left incomplete when other questions have been answered.

For peer review only - http://bmjopen.bmj.com/site/about/guidelines.xhtml

Supplemental Table S2f: Available EQ-5D-Y items

	Co-amoxiclav (N=136)						Placebo (N=135)						Total (N=271)					
	**M**	**SC**	**UA**	**PD**	**AD**	**VAS**	**M**	**SC**	**UA**	**PD**	**AD**	**VAS**	**M**	**SC**	**UA**	**PD**	**AD**	**VAS**
**Availability of EQ-5D-3L index proxy, completed on behalf of child**																		
Day 0	120 (88%)	**97 (71%)**	128 (94%)	131 (96%)	127 (93%)	120 (88%)	125 (93%)	**110 (81%)**	135 (100%)	135 (100%)	134 (99%)	129 (96%)	245 (90%)	**207 (76%)**	263 (97%)	266 (98%)	261 (96%)	249 (92%)
Day 4	69 (51%)	**54 (40%)**	75 (55%)	76 (56%)	76 (56%)	77 (57%)	63 (47%)	**54 (40%)**	75 (56%)	77 (57%)	76 (56%)	76 (56%)	132 (49%)	**108 (40%)**	150 (55%)	153 (56%)	152 (56%)	153 (56%)
Day 7	68 (50%)	**52 (38%)**	74 (54%)	75 (55%)	76 (56%)	78 (57%)	61 (45%)	**56 (41%)**	72 (53%)	71 (53%)	73 (54%)	73 (54%)	129 (48%)	**108 (40%)**	146 (54%)	146 (54%)	149 (55%)	151 (56%)
Day 14	53 (39%)	**44 (32%)**	59 (43%)	59 (43%)	59 (43%)	61 (45%)	50 (37%)	**47 (35%)**	58 (43%)	59 (44%)	59 (44%)	57 (42%)	103 (38%)	**91 (34%)**	117 (43%)	118 (44%)	118 (44%)	118 (44%)
Day 28	48 (35%)	**43 (32%)**	53 (39%)	53 (39%)	51 (38%)	53 (39%)	34 (25%)	**32 (24%)**	39 (29%)	39 (29%)	39 (29%)	40 (30%)	82 (30%)	**75 (28%)**	92 (34%)	92 (34%)	90 (33%)	93 (34%)
**Availability of EQ-5D-3L index completed by child**																		
Day 0	32 (24%)	32 (24%)	32 (24%)	32 (24%)	32 (24%)	32 (24%)	30 (22%)	30 (22%)	31 (23%)	31 (23%)	30 (22%)	32 (24%)	62 (23%)	62 (23%)	63 (23%)	63 (23%)	62 (23%)	64 (24%)
Day 4	17 (13%)	16 (12%)	19 (14%)	19 (14%)	19 (14%)	19 (14%)	13 (10%)	13 (10%)	13 (10%)	13 (10%)	13 (10%)	13 (10%)	30 (11%)	29 (11%)	32 (12%)	32 (12%)	32 (12%)	32 (12%)
Day 7	18 (13%)	17 (13%)	20 (15%)	20 (15%)	20 (15%)	19 (14%)	11 (8%)	11 (8%)	11 (8%)	11 (8%)	11 (8%)	11 (8%)	29 (11%)	28 (10%)	31 (11%)	31 (11%)	31 (11%)	30 (11%)
Day 14	18 (13%)	17 (13%)	20 (15%)	20 (15%)	20 (15%)	20 (15%)	10 (7%)	10 (7%)	10 (7%)	10 (7%)	10 (7%)	10 (7%)	28 (10%)	27 (10%)	30 (11%)	30 (11%)	30 (11%)	30 (11%)
Day 28	17 (13%)	17 (13%)	19 (14%)	19 (14%)	19 (14%)	18 (13%)	10 (7%)	10 (7%)	10 (7%)	10 (7%)	10 (7%)	11 (8%)	27 (10%)	27 (10%)	29 (11%)	29 (11%)	29 (11%)	29 (11%)

M – mobility, SC – Self-care, UA – Usual activities, PD – Pain/ discomfort, AD – Anxiety/ depression

Items with higher rates of missing data are highlighted in bold

For peer review only - http://bmjopen.bmj.com/site/about/guidelines.xhtml

Supplemental Table S2g: Available EQ-5D-Y items for children aged less than 2 years

	Co-amoxiclav (N=45)						Placebo (N=44)						Total (N=89)					
	**M**	**SC**	**UA**	**PD**	**AD**	**VAS**	**M**	**SC**	**UA**	**PD**	**AD**	**VAS**	**M**	**SC**	**UA**	**PD**	**AD**	**VAS**
**Availability of EQ-5D-3L index proxy, completed on behalf of child**																		
Day 0	34 (76%)	**18 (40%)**	40 (89%)	43 (96%)	39 (87%)	39 (87%)	**35 (80%)**	**25 (57%)**	44 (100%)	44 (100%)	44 (100%)	43 (98%)	69 (78%)	**43 (48%)**	84 (94%)	87 (98%)	83 (93%)	82 (92%)
Day 4	20 (44%)	**10 (22%)**	25 (56%)	27 (60%)	27 (60%)	27 (60%)	**19 (43%)**	**13 (30%)**	27 (61%)	29 (66%)	28 (64%)	29 (66%)	39 (44%)	**23 (26%)**	52 (58%)	56 (63%)	55 (62%)	56 (63%)
Day 7	19 (42%)	**9 (20%)**	24 (53%)	25 (56%)	26 (58%)	27 (60%)	**18 (41%)**	**15 (34%)**	26 (59%)	26 (59%)	27 (61%)	28 (64%)	37 (42%)	**24 (27%)**	50 (56%)	51 (57%)	53 (60%)	55 (62%)
Day 14	17 (38%)	**10 (22%)**	22 (49%)	22 (49%)	22 (49%)	22 (49%)	**16 (36%)**	**14 (32%)**	24 (55%)	25 (57%)	25 (57%)	24 (55%)	33 (37%)	**24 (27%)**	46 (52%)	47 (53%)	47 (53%)	46 (52%)
Day 28	15 (33%)	**11 (24%)**	20 (44%)	20 (44%)	20 (44%)	19 (42%)	**14 (32%)**	**12 (27%)**	19 (43%)	19 (43%)	19 (43%)	19 (43%)	29 (33%)	**23 (26%)**	39 (44%)	39 (44%)	39 (44%)	38 (43%)
**Availability of EQ-5D-3L index completed by child**																		
Day 0	0 (0%)	0 (0%)	0 (0%)	0 (0%)	0 (0%)	0 (0%)	0 (0%)	0 (0%)	1 (2%)	1 (2%)	1 (2%)	1 (2%)	0 (0%)	0 (0%)	1 (1%)	1 (1%)	1 (1%)	1 (1%)
Day 4	1 (2%)	0 (0%)	3 (7%)	3 (7%)	3 (7%)	3 (7%)	1 (2%)	1 (2%)	1 (2%)	1 (2%)	1 (2%)	1 (2%)	2 (2%)	1 (1%)	4 (4%)	4 (4%)	4 (4%)	4 (4%)
Day 7	1 (2%)	0 (0%)	3 (7%)	3 (7%)	3 (7%)	3 (7%)	1 (2%)	1 (2%)	1 (2%)	1 (2%)	1 (2%)	1 (2%)	2 (2%)	1 (1%)	4 (4%)	4 (4%)	4 (4%)	4 (4%)
Day 14	1 (2%)	0 (0%)	3 (7%)	3 (7%)	3 (7%)	3 (7%)	1 (2%)	1 (2%)	1 (2%)	1 (2%)	1 (2%)	1 (2%)	2 (2%)	1 (1%)	4 (4%)	4 (4%)	4 (4%)	4 (4%)
Day 28	0 (0%)	0 (0%)	2 (4%)	2 (4%)	2 (4%)	2 (4%)	1 (2%)	1 (2%)	1 (2%)	1 (2%)	1 (2%)	1 (2%)	1 (1%)	1 (1%)	3 (3%)	3 (3%)	3 (3%)	3 (3%)

M – mobility, SC – Self-care, UA – Usual activities, PD – Pain/ discomfort, AD – Anxiety/ depression
Items with higher rates of missing data are highlighted in bold

For peer review only - http://bmjopen.bmj.com/site/about/guidelines.xhtml

Supplemental Table S2h: Available EQ-5D-Y items for children aged 2+ years (proxy)

	Co-amoxiclav (N=91)						Placebo (N=91)						Total (N=182)					
	**M**	**SC**	**UA**	**PD**	**AD**	**VAS**	**M**	**SC**	**UA**	**PD**	**AD**	**VAS**	**M**	**SC**	**UA**	**PD**	**AD**	**VAS**
**Availability of EQ-5D-3L index proxy, completed on behalf of child**																		
Day 0	86 (95%)	**79 (87%)**	88 (97%)	88 (97%)	88 (97%)	81 (89%)	90 (99%)	**85 (93%)**	91 (100%)	91 (100%)	90 (99%)	86 (95%)	176 (97%)	**164 (90%)**	179 (98%)	179 (98%)	178 (98%)	**167 (92%)**
Day 4	49 (54%)	**44 (48%)**	50 (55%)	49 (54%)	49 (54%)	50 (55%)	44 (48%)	**41 (45%)**	48 (53%)	48 (53%)	48 (53%)	47 (52%)	93 (51%)	**85 (47%)**	98 (54%)	97 (53%)	97 (53%)	97 (53%)
Day 7	49 (54%)	**43 (47%)**	50 (55%)	50 (55%)	50 (55%)	51 (56%)	43 (47%)	41 (45%)	46 (51%)	45 (49%)	46 (51%)	45 (49%)	92 (51%)	84 (46%)	96 (53%)	95 (52%)	96 (53%)	96 (53%)
Day 14	36 (40%)	34 (37%)	37 (41%)	37 (41%)	37 (41%)	39 (43%)	34 (37%)	33 (36%)	34 (37%)	34 (37%)	34 (37%)	33 (36%)	70 (38%)	67 (37%)	71 (39%)	71 (39%)	71 (39%)	72 (40%)
Day 28	33 (36%)	32 (35%)	33 (36%)	33 (36%)	31 (34%)	34 (37%)	20 (22%)	20 (22%)	20 (22%)	20 (22%)	20 (22%)	21 (23%)	53 (29%)	52 (29%)	53 (29%)	53 (29%)	51 (28%)	55 (30%)
**Availability of EQ-5D-3L index completed by child**																		
Day 0	32 (35%)	32 (35%)	32 (35%)	32 (35%)	32 (35%)	32 (35%)	30 (33%)	30 (33%)	30 (33%)	30 (33%)	29 (32%)	31 (34%)	62 (34%)	62 (34%)	62 (34%)	62 (34%)	61 (34%)	63 (35%)
Day 4	16 (18%)	16 (18%)	16 (18%)	16 (18%)	16 (18%)	16 (18%)	12 (13%)	12 (13%)	12 (13%)	12 (13%)	12 (13%)	12 (13%)	28 (15%)	28 (15%)	28 (15%)	28 (15%)	28 (15%)	28 (15%)
Day 7	17 (19%)	17 (19%)	17 (19%)	17 (19%)	17 (19%)	16 (18%)	10 (11%)	10 (11%)	10 (11%)	10 (11%)	10 (11%)	10 (11%)	27 (15%)	27 (15%)	27 (15%)	27 (15%)	27 (15%)	26 (14%)
Day 14	17 (19%)	17 (19%)	17 (19%)	17 (19%)	17 (19%)	17 (19%)	9 (10%)	9 (10%)	9 (10%)	9 (10%)	9 (10%)	9 (10%)	26 (14%)	26 (14%)	26 (14%)	26 (14%)	26 (14%)	26 (14%)
Day 28	17 (19%)	17 (19%)	17 (19%)	17 (19%)	17 (19%)	16 (18%)	9 (10%)	9 (10%)	9 (10%)	9 (10%)	9 (10%)	10 (11%)	26 (14%)	26 (14%)	26 (14%)	26 (14%)	26 (14%)	26 (14%)

M – mobility, SC – Self-care, UA – Usual activities, PD – Pain/ discomfort, AD – Anxiety/ depression
Items with higher rates of missing data are highlighted in bold

For peer review only - http://bmjopen.bmj.com/site/about/guidelines.xhtml

Supplemental File 3: Reponses to the EQ-5D-Y items

The following tables present the responses to the EQ-5D-Y items at the relevant time points, both for proxy-completed and self-completed questionnaires. Additional tables also show responses separately for children aged less than 2 years, and 2+ years. The summaries include participants for whom the EQ-5D-Y index is available, i.e. for whom all five responses were available.

For peer review only

For peer review only - http://bmjopen.bmj.com/site/about/guidelines.xhtml

Supplemental Table S3a: Responses to EQ-5D-Y domains (proxy, completed on behalf of child)

	M	SC	UA	PD	AD	M	SC	UA	PD	AD	M	SC	UA	PD	AD
**Baseline**	Co-amoxiclav (N = 96)					Placebo (N = 108)					Total (N = 204)				
**No Problems**	78 (81%)	65 (68%)	37 (39%)	22 (23%)	35 (36%)	80 (74%)	67 (62%)	44 (41%)	33 (31%)	49 (45%)	158 (77%)	132 (65%)	81 (40%)	55 (27%)	84 (41%)
**Moderate problems**	11 (11%)	22 (23%)	44 (46%)	67 (70%)	55 (57%)	21 (19%)	22 (20%)	52 (48%)	63 (58%)	51 (47%)	32 (16%)	44 (22%)	96 (47%)	130 (64%)	106 (52%)
**Extreme problems**	7 (7%)	9 (9%)	15 (16%)	7 (7%)	6 (6%)	7 (6%)	19 (18%)	12 (11%)	12 (11%)	8 (7%)	14 (7%)	28 (14%)	27 (13%)	19 (9%)	14 (7%)
**Day 4**	Co-amoxiclav (N = 52)					Placebo (N = 52)					Total (N = 104)				
**No Problems**	38 (73%)	40 (77%)	22 (42%)	18 (35%)	26 (50%)	39 (75%)	34 (65%)	24 (46%)	22 (42%)	32 (62%)	77 (74%)	74 (71%)	46 (44%)	40 (38%)	58 (56%)
**Moderate problems**	10 (19%)	8 (15%)	20 (38%)	31 (60%)	24 (46%)	12 (23%)	14 (27%)	22 (42%)	28 (54%)	19 (37%)	22 (21%)	22 (21%)	42 (40%)	59 (57%)	43 (41%)
**Extreme problems**	4 (8%)	4 (8%)	10 (19%)	3 (6%)	2 (4%)	1 (2%)	4 (8%)	6 (12%)	2 (4%)	1 (2%)	5 (5%)	8 (8%)	16 (15%)	5 (5%)	3 (3%)
**Day 7**	Co-amoxiclav (N = 52)					Placebo (N = 54)					Total (N = 106)				
**No Problems**	44 (85%)	46 (88%)	38 (73%)	32 (62%)	43 (83%)	46 (85%)	41 (76%)	33 (61%)	33 (61%)	41 (76%)	90 (85%)	87 (82%)	71 (67%)	65 (61%)	84 (79%)
**Moderate problems**	3 (6%)	2 (4%)	11 (21%)	17 (33%)	8 (15%)	6 (11%)	8 (15%)	18 (33%)	18 (33%)	12 (22%)	9 (8%)	10 (9%)	29 (27%)	35 (33%)	20 (19%)
**Extreme problems**	5 (10%)	4 (8%)	3 (6%)	3 (6%)	1 (2%)	2 (4%)	5 (9%)	3 (6%)	3 (6%)	1 (2%)	7 (7%)	9 (8%)	6 (6%)	6 (6%)	2 (2%)
**Day 14**	Co-amoxiclav (N = 44)					Placebo (N = 46)					Total (N = 90)				
**No Problems**	41 (93%)	40 (91%)	35 (80%)	35 (80%)	38 (86%)	43 (93%)	40 (87%)	40 (87%)	39 (85%)	40 (87%)	84 (93%)	80 (89%)	75 (83%)	74 (82%)	78 (87%)
**Moderate problems**	0 (0%)	2 (5%)	7 (16%)	6 (14%)	5 (11%)	3 (7%)	5 (11%)	6 (13%)	7 (15%)	6 (13%)	3 (3%)	7 (8%)	13 (14%)	13 (14%)	11 (12%)
**Extreme problems**	3 (7%)	2 (5%)	2 (5%)	3 (7%)	1 (2%)	0 (0%)	1 (2%)	0 (0%)	0 (0%)	0 (0%)	3 (3%)	3 (3%)	2 (2%)	3 (3%)	1 (1%)
**Day 28**	Co-amoxiclav (N = 41)					Placebo (N = 31)					Total (N = 72)				
**No Problems**	39 (95%)	39 (95%)	37 (90%)	35 (85%)	36 (88%)	28 (90%)	28 (90%)	28 (90%)	26 (84%)	28 (90%)	67 (93%)	67 (93%)	65 (90%)	61 (85%)	64 (89%)
**Moderate problems**	0 (0%)	0 (0%)	4 (10%)	5 (12%)	5 (12%)	3 (10%)	2 (6%)	3 (10%)	5 (16%)	3 (10%)	3 (4%)	2 (3%)	7 (10%)	10 (14%)	8 (11%)
**Extreme problems**	2 (5%)	2 (5%)	0 (0%)	1 (2%)	0 (0%)	0 (0%)	1 (3%)	0 (0%)	0 (0%)	0 (0%)	2 (3%)	3 (4%)	0 (0%)	1 (1%)	0 (0%)

M – mobility, SC – Self-care, UA – Usual activities, PD – Pain/ discomfort, AD – Anxiety/ depression

For peer review only

For peer review only - http://bmjopen.bmj.com/site/about/guidelines.xhtml

Supplemental Table S3b: Responses to EQ-5D-Y domains (proxy, completed on behalf of child for children aged less than 2 years)

	M	SC	UA	PD	AD	M	SC	UA	PD	AD	M	SC	UA	PD	AD
**Baseline**	Co-amoxiclav (N = 18)					Placebo (N = 24)					Total (N = 42)				
**No Problems**	14 (78%)	15 (83%)	11 (61%)	4 (22%)	6 (33%)	19 (79%)	13 (54%)	13 (54%)	8 (33%)	7 (29%)	33 (79%)	28 (67%)	24 (57%)	12 (29%)	13 (31%)
**Moderate problems**	2 (11%)	0 (0%)	5 (28%)	14 (78%)	11 (61%)	4 (17%)	5 (21%)	11 (46%)	16 (67%)	15 (63%)	6 (14%)	5 (12%)	16 (38%)	30 (71%)	26 (62%)
**Extreme problems**	2 (11%)	3 (17%)	2 (11%)	0 (0%)	1 (6%)	1 (4%)	6 (25%)	0 (0%)	0 (0%)	2 (8%)	3 (7%)	9 (21%)	2 (5%)	0 (0%)	3 (7%)
**Day 4**	Co-amoxiclav (N = 9)					Placebo (N = 12)					Total (N = 21)				
**No Problems**	7 (78%)	7 (78%)	6 (67%)	4 (44%)	6 (67%)	10 (83%)	9 (75%)	7 (58%)	4 (33%)	6 (50%)	17 (81%)	16 (76%)	13 (62%)	8 (38%)	12 (57%)
**Moderate problems**	1 (11%)	1 (11%)	2 (22%)	5 (56%)	3 (33%)	2 (17%)	2 (17%)	5 (42%)	8 (67%)	6 (50%)	3 (14%)	3 (14%)	7 (33%)	13 (62%)	9 (43%)
**Extreme problems**	1 (11%)	1 (11%)	1 (11%)	0 (0%)	0 (0%)	0 (0%)	1 (8%)	0 (0%)	0 (0%)	0 (0%)	1 (5%)	2 (10%)	1 (5%)	0 (0%)	0 (0%)
**Day 7**	Co-amoxiclav (N = 9)					Placebo (N = 14)					Total (N = 23)				
**No Problems**	8 (89%)	8 (89%)	7 (78%)	7 (78%)	9 (100%)	11 (79%)	10 (71%)	9 (64%)	8 (57%)	10 (71%)	19 (83%)	18 (78%)	16 (70%)	15 (65%)	19 (83%)
**Moderate problems**	0 (0%)	0 (0%)	1 (11%)	2 (22%)	0 (0%)	3 (21%)	3 (21%)	5 (36%)	6 (43%)	4 (29%)	3 (13%)	3 (13%)	6 (26%)	8 (35%)	4 (17%)
**Extreme problems**	1 (11%)	1 (11%)	1 (11%)	0 (0%)	0 (0%)	0 (0%)	1 (7%)	0 (0%)	0 (0%)	0 (0%)	1 (4%)	2 (9%)	1 (4%)	0 (0%)	0 (0%)
**Day 14**	Co-amoxiclav (N = 10)					Placebo (N = 13)					Total (N = 23)				
**No Problems**	10 (100%)	9 (90%)	8 (80%)	7 (70%)	8 (80%)	11 (85%)	10 (77%)	10 (77%)	10 (77%)	10 (77%)	21 (91%)	19 (83%)	18 (78%)	17 (74%)	18 (78%)
**Moderate problems**	0 (0%)	1 (10%)	2 (20%)	2 (20%)	2 (20%)	2 (15%)	3 (23%)	3 (23%)	3 (23%)	3 (23%)	2 (9%)	4 (17%)	5 (22%)	5 (22%)	5 (22%)
**Extreme problems**	0 (0%)	0 (0%)	0 (0%)	1 (10%)	0 (0%)	0 (0%)	0 (0%)	0 (0%)	0 (0%)	0 (0%)	0 (0%)	0 (0%)	0 (0%)	1 (4%)	0 (0%)
**Day 28**	Co-amoxiclav (N = 11)					Placebo (N = 11)					Total (N = 22)				
**No Problems**	11 (100%)	11 (100%)	11 (100%)	10 (91%)	11 (100%)	10 (91%)	10 (91%)	10 (91%)	8 (73%)	9 (82%)	21 (95%)	21 (95%)	21 (95%)	18 (82%)	20 (91%)
**Moderate problems**	0 (0%)	0 (0%)	0 (0%)	1 (9%)	0 (0%)	1 (9%)	1 (9%)	1 (9%)	3 (27%)	2 (18%)	1 (5%)	1 (5%)	1 (5%)	4 (18%)	2 (9%)
**Extreme problems**	0 (0%)	0 (0%)	0 (0%)	0 (0%)	0 (0%)	0 (0%)	0 (0%)	0 (0%)	0 (0%)	0 (0%)	0 (0%)	0 (0%)	0 (0%)	0 (0%)	0 (0%)

M – mobility, SC – Self-care, UA – Usual activities, PD – Pain/ discomfort, AD – Anxiety/ depression

For peer review only

For peer review only - http://bmjopen.bmj.com/site/about/guidelines.xhtml

Supplemental Table S3c: Responses to EQ-5D-Y domains (proxy, completed on behalf of child for children aged 2+ years)

	M	SC	UA	PD	AD	M	SC	UA	PD	AD	M	SC	UA	PD	AD
**Baseline**	Co-amoxiclav (N = 78)					Placebo (N = 84)					Total (N = 162)				
**No Problems**	64 (82%)	50 (64%)	26 (33%)	18 (23%)	29 (37%)	61 (73%)	54 (64%)	31 (37%)	25 (30%)	42 (50%)	125 (77%)	104 (64%)	57 (35%)	43 (27%)	71 (44%)
**Moderate problems**	9 (12%)	22 (28%)	39 (50%)	53 (68%)	44 (56%)	17 (20%)	17 (20%)	41 (49%)	47 (56%)	36 (43%)	26 (16%)	39 (24%)	80 (49%)	100 (62%)	80 (49%)
**Extreme problems**	5 (6%)	6 (8%)	13 (17%)	7 (9%)	5 (6%)	6 (7%)	13 (15%)	12 (14%)	12 (14%)	6 (7%)	11 (7%)	19 (12%)	25 (15%)	19 (12%)	11 (7%)
**Day 4**	Co-amoxiclav (N = 43)					Placebo (N = 40)					Total (N = 83)				
**No Problems**	31 (72%)	33 (77%)	16 (37%)	14 (33%)	20 (47%)	29 (73%)	25 (63%)	17 (43%)	18 (45%)	26 (65%)	60 (72%)	58 (70%)	33 (40%)	32 (39%)	46 (55%)
**Moderate problems**	9 (21%)	7 (16%)	18 (42%)	26 (60%)	21 (49%)	10 (25%)	12 (30%)	17 (43%)	20 (50%)	13 (33%)	19 (23%)	19 (23%)	35 (42%)	46 (55%)	34 (41%)
**Extreme problems**	3 (7%)	3 (7%)	9 (21%)	3 (7%)	2 (5%)	1 (3%)	3 (8%)	6 (15%)	2 (5%)	1 (3%)	4 (5%)	6 (7%)	15 (18%)	5 (6%)	3 (4%)
**Day 7**	Co-amoxiclav (N = 43)					Placebo (N = 40)					Total (N = 83)				
**No Problems**	36 (84%)	38 (88%)	31 (72%)	25 (58%)	34 (79%)	35 (88%)	31 (78%)	24 (60%)	25 (63%)	31 (78%)	71 (86%)	69 (83%)	55 (66%)	50 (60%)	65 (78%)
**Moderate problems**	3 (7%)	2 (5%)	10 (23%)	15 (35%)	8 (19%)	3 (8%)	5 (13%)	13 (33%)	12 (30%)	8 (20%)	6 (7%)	7 (8%)	23 (28%)	27 (33%)	16 (19%)
**Extreme problems**	4 (9%)	3 (7%)	2 (5%)	3 (7%)	1 (2%)	2 (5%)	4 (10%)	3 (8%)	3 (8%)	1 (3%)	6 (7%)	7 (8%)	5 (6%)	6 (7%)	2 (2%)
**Day 14**	Co-amoxiclav (N = 34)					Placebo (N = 33)					Total (N = 67)				
**No Problems**	31 (91%)	31 (91%)	27 (79%)	28 (82%)	30 (88%)	32 (97%)	30 (91%)	30 (91%)	29 (88%)	30 (91%)	63 (94%)	61 (91%)	57 (85%)	57 (85%)	60 (90%)
**Moderate problems**	0 (0%)	1 (3%)	5 (15%)	4 (12%)	3 (9%)	1 (3%)	2 (6%)	3 (9%)	4 (12%)	3 (9%)	1 (1%)	3 (4%)	8 (12%)	8 (12%)	6 (9%)
**Extreme problems**	3 (9%)	2 (6%)	2 (6%)	2 (6%)	1 (3%)	0 (0%)	1 (3%)	0 (0%)	0 (0%)	0 (0%)	3 (4%)	3 (4%)	2 (3%)	2 (3%)	1 (1%)
**Day 28**	Co-amoxiclav (N = 30)					Placebo (N = 20)					Total (N = 50)				
**No Problems**	28 (93%)	28 (93%)	26 (87%)	25 (83%)	25 (83%)	18 (90%)	18 (90%)	18 (90%)	18 (90%)	19 (95%)	46 (92%)	46 (92%)	44 (88%)	43 (86%)	44 (88%)
**Moderate problems**	0 (0%)	0 (0%)	4 (13%)	4 (13%)	5 (17%)	2 (10%)	1 (5%)	2 (10%)	2 (10%)	1 (5%)	2 (4%)	1 (2%)	6 (12%)	6 (12%)	6 (12%)
**Extreme problems**	2 (7%)	2 (7%)	0 (0%)	1 (3%)	0 (0%)	0 (0%)	1 (5%)	0 (0%)	0 (0%)	0 (0%)	2 (4%)	3 (6%)	0 (0%)	1 (2%)	0 (0%)

M – mobility, SC – Self-care, UA – Usual activities, PD – Pain/ discomfort, AD – Anxiety/ depression

For peer review only

Supplemental Table S3d: Responses to EQ-5D-Y domains (completed by child)

	M	SC	UA	PD	AD	M	SC	UA	PD	AD	M	SC	UA	PD	AD
**Baseline**	Co-amoxiclav (N = 32)					Placebo (N = 29)					Total (N = 61)				
**No Problems**	20 (63%)	24 (75%)	4 (13%)	6 (19%)	17 (53%)	24 (83%)	23 (79%)	9 (31%)	5 (17%)	13 (45%)	44 (72%)	47 (77%)	13 (21%)	11 (18%)	30 (49%)
**Moderate problems**	10 (31%)	6 (19%)	22 (69%)	16 (50%)	14 (44%)	5 (17%)	5 (17%)	15 (52%)	16 (55%)	14 (48%)	15 (25%)	11 (18%)	37 (61%)	32 (52%)	28 (46%)
**Extreme problems**	2 (6%)	2 (6%)	6 (19%)	10 (31%)	1 (3%)	0 (0%)	1 (3%)	5 (17%)	8 (28%)	2 (7%)	2 (3%)	3 (5%)	11 (18%)	18 (30%)	3 (5%)
**Day 4**	Co-amoxiclav (N = 16)					Placebo (N = 13)					Total (N = 29)				
**No Problems**	13 (81%)	13 (81%)	7 (44%)	5 (31%)	7 (44%)	10 (77%)	10 (77%)	5 (38%)	4 (31%)	7 (54%)	23 (79%)	23 (79%)	12 (41%)	9 (31%)	14 (48%)
**Moderate problems**	2 (13%)	2 (13%)	5 (31%)	10 (63%)	8 (50%)	3 (23%)	3 (23%)	8 (62%)	8 (62%)	6 (46%)	5 (17%)	5 (17%)	13 (45%)	18 (62%)	14 (48%)
**Extreme problems**	1 (6%)	1 (6%)	4 (25%)	1 (6%)	1 (6%)	0 (0%)	0 (0%)	0 (0%)	1 (8%)	0 (0%)	1 (3%)	1 (3%)	4 (14%)	2 (7%)	1 (3%)
**Day 7**	Co-amoxiclav (N = 17)					Placebo (N = 11)					Total (N = 28)				
**No Problems**	14 (82%)	16 (94%)	10 (59%)	12 (71%)	14 (82%)	10 (91%)	8 (73%)	6 (55%)	5 (45%)	7 (64%)	24 (86%)	24 (86%)	16 (57%)	17 (61%)	21 (75%)
**Moderate problems**	2 (12%)	0 (0%)	6 (35%)	4 (24%)	2 (12%)	1 (9%)	3 (27%)	5 (45%)	6 (55%)	4 (36%)	3 (11%)	3 (11%)	11 (39%)	10 (36%)	6 (21%)
**Extreme problems**	1 (6%)	1 (6%)	1 (6%)	1 (6%)	1 (6%)	0 (0%)	0 (0%)	0 (0%)	0 (0%)	0 (0%)	1 (4%)	1 (4%)	1 (4%)	1 (4%)	1 (4%)
**Day 14**	Co-amoxiclav (N = 17)					Placebo (N = 10)					Total (N = 27)				
**No Problems**	16 (94%)	16 (94%)	14 (82%)	16 (94%)	17 (100%)	10 (100%)	9 (90%)	9 (90%)	7 (70%)	9 (90%)	26 (96%)	25 (93%)	23 (85%)	23 (85%)	26 (96%)
**Moderate problems**	1 (6%)	1 (6%)	3 (18%)	1 (6%)	0 (0%)	0 (0%)	1 (10%)	1 (10%)	3 (30%)	1 (10%)	1 (4%)	2 (7%)	4 (15%)	4 (15%)	1 (4%)
**Extreme problems**	0 (0%)	0 (0%)	0 (0%)	0 (0%)	0 (0%)	0 (0%)	0 (0%)	0 (0%)	0 (0%)	0 (0%)	0 (0%)	0 (0%)	0 (0%)	0 (0%)	0 (0%)
**Day 28**	Co-amoxiclav (N = 17)					Placebo (N = 10)					Total (N = 27)				
**No Problems**	17 (100%)	17 (100%)	15 (88%)	17 (100%)	17 (100%)	10 (100%)	10 (100%)	9 (90%)	8 (80%)	8 (80%)	27 (100%)	27 (100%)	24 (89%)	25 (93%)	25 (93%)
**Moderate problems**	0 (0%)	0 (0%)	2 (12%)	0 (0%)	0 (0%)	0 (0%)	0 (0%)	1 (10%)	2 (20%)	2 (20%)	0 (0%)	0 (0%)	3 (11%)	2 (7%)	2 (7%)
**Extreme problems**	0 (0%)	0 (0%)	0 (0%)	0 (0%)	0 (0%)	0 (0%)	0 (0%)	0 (0%)	0 (0%)	0 (0%)	0 (0%)	0 (0%)	0 (0%)	0 (0%)	0 (0%)

M – mobility, SC – Self-care, UA – Usual activities, PD – Pain/ discomfort, AD – Anxiety/ depression

For peer review only - http://bmjopen.bmj.com/site/about/guidelines.xhtml

Supplemental Table S3e: Responses to EQ-5D-Y domains (completed by child for children aged less than 2 years)

	M	SC	UA	PD	AD	M	SC	UA	PD	AD	M	SC	UA	PD	AD
**Baseline**	Co-amoxiclav (N = 0)					Placebo (N = 0)					Total (N = 0)				
**No Problems**	0 (.%)	0 (.%)	0 (.%)	0 (.%)	0 (.%)	0 (.%)	0 (.%)	0 (.%)	0 (.%)	0 (.%)	0 (.%)	0 (.%)	0 (.%)	0 (.%)	0 (.%)
**Moderate problems**	0 (.%)	0 (.%)	0 (.%)	0 (.%)	0 (.%)	0 (.%)	0 (.%)	0 (.%)	0 (.%)	0 (.%)	0 (.%)	0 (.%)	0 (.%)	0 (.%)	0 (.%)
**Extreme problems**	0 (.%)	0 (.%)	0 (.%)	0 (.%)	0 (.%)	0 (.%)	0 (.%)	0 (.%)	0 (.%)	0 (.%)	0 (.%)	0 (.%)	0 (.%)	0 (.%)	0 (.%)
**Day 4**	Co-amoxiclav (N = 0)					Placebo (N = 1)					Total (N = 1)				
**No Problems**	0 (.%)	0 (.%)	0 (.%)	0 (.%)	0 (.%)	1 (100%)	0 (0%)	1 (100%)	0 (0%)	0 (0%)	1 (100%)	0 (0%)	1 (100%)	0 (0%)	0 (0%)
**Moderate problems**	0 (.%)	0 (.%)	0 (.%)	0 (.%)	0 (.%)	0 (0%)	1 (100%)	0 (0%)	1 (100%)	1 (100%)	0 (0%)	1 (100%)	0 (0%)	1 (100%)	1 (100%)
**Extreme problems**	0 (.%)	0 (.%)	0 (.%)	0 (.%)	0 (.%)	0 (0%)	0 (0%)	0 (0%)	0 (0%)	0 (0%)	0 (0%)	0 (0%)	0 (0%)	0 (0%)	0 (0%)
**Day 7**	Co-amoxiclav (N = 0)					Placebo (N = 1)					Total (N = 1)				
**No Problems**	0 (.%)	0 (.%)	0 (.%)	0 (.%)	0 (.%)	1 (100%)	0 (0%)	0 (0%)	0 (0%)	0 (0%)	1 (100%)	0 (0%)	0 (0%)	0 (0%)	0 (0%)
**Moderate problems**	0 (.%)	0 (.%)	0 (.%)	0 (.%)	0 (.%)	0 (0%)	1 (100%)	1 (100%)	1 (100%)	1 (100%)	0 (0%)	1 (100%)	1 (100%)	1 (100%)	1 (100%)
**Extreme problems**	0 (.%)	0 (.%)	0 (.%)	0 (.%)	0 (.%)	0 (0%)	0 (0%)	0 (0%)	0 (0%)	0 (0%)	0 (0%)	0 (0%)	0 (0%)	0 (0%)	0 (0%)
**Day 14**	Co-amoxiclav (N = 0)					Placebo (N = 1)					Total (N = 1)				
**No Problems**	0 (.%)	0 (.%)	0 (.%)	0 (.%)	0 (.%)	1 (100%)	0 (0%)	0 (0%)	0 (0%)	0 (0%)	1 (100%)	0 (0%)	0 (0%)	0 (0%)	0 (0%)
**Moderate problems**	0 (.%)	0 (.%)	0 (.%)	0 (.%)	0 (.%)	0 (0%)	1 (100%)	1 (100%)	1 (100%)	1 (100%)	0 (0%)	1 (100%)	1 (100%)	1 (100%)	1 (100%)
**Extreme problems**	0 (.%)	0 (.%)	0 (.%)	0 (.%)	0 (.%)	0 (0%)	0 (0%)	0 (0%)	0 (0%)	0 (0%)	0 (0%)	0 (0%)	0 (0%)	0 (0%)	0 (0%)
**Day 28**	Co-amoxiclav (N = 0)					Placebo (N = 1)					Total (N = 1)				
**No Problems**	0 (.%)	0 (.%)	0 (.%)	0 (.%)	0 (.%)	1 (100%)	1 (100%)	1 (100%)	0 (0%)	0 (0%)	1 (100%)	1 (100%)	1 (100%)	0 (0%)	0 (0%)
**Moderate problems**	0 (.%)	0 (.%)	0 (.%)	0 (.%)	0 (.%)	0 (0%)	0 (0%)	0 (0%)	1 (100%)	1 (100%)	0 (0%)	0 (0%)	0 (0%)	1 (100%)	1 (100%)
**Extreme problems**	0 (.%)	0 (.%)	0 (.%)	0 (.%)	0 (.%)	0 (0%)	0 (0%)	0 (0%)	0 (0%)	0 (0%)	0 (0%)	0 (0%)	0 (0%)	0 (0%)	0 (0%)

M – mobility, SC – Self-care, UA – Usual activities, PD – Pain/ discomfort, AD – Anxiety/ depression

For peer review only

For peer review only - http://bmjopen.bmj.com/site/about/guidelines.xhtml

Supplemental Table S3f: Responses to EQ-5D-Y domains (completed by child for children aged 2+ years)

	M	SC	UA	PD	AD	M	SC	UA	PD	AD	M	SC	UA	PD	AD
**Baseline**	Co-amoxiclav (N = 32)					Placebo (N = 29)					Total (N = 61)				
**No Problems**	20 (63%)	24 (75%)	4 (13%)	6 (19%)	10 (31%)	24 (83%)	23 (79%)	9 (31%)	5 (17%)	11 (38%)	44 (72%)	47 (77%)	13 (21%)	11 (18%)	21 (34%)
**Moderate problems**	10 (31%)	6 (19%)	22 (69%)	16 (50%)	20 (63%)	5 (17%)	5 (17%)	15 (52%)	16 (55%)	15 (52%)	15 (25%)	11 (18%)	37 (61%)	32 (52%)	35 (57%)
**Extreme problems**	2 (6%)	2 (6%)	6 (19%)	10 (31%)	2 (6%)	0 (0%)	1 (3%)	5 (17%)	8 (28%)	3 (10%)	2 (3%)	3 (5%)	11 (18%)	18 (30%)	5 (8%)
**Day 4**	Co-amoxiclav (N = 16)					Placebo (N = 12)					Total (N = 28)				
**No Problems**	13 (81%)	13 (81%)	7 (44%)	5 (31%)	7 (47%)	9 (75%)	10 (83%)	4 (33%)	4 (33%)	7 (64%)	22 (79%)	23 (82%)	11 (39%)	9 (32%)	14 (54%)
**Moderate problems**	2 (13%)	2 (13%)	5 (31%)	10 (63%)	7 (47%)	3 (25%)	2 (17%)	8 (67%)	7 (58%)	4 (36%)	5 (18%)	4 (14%)	13 (46%)	17 (61%)	11 (42%)
**Extreme problems**	1 (6%)	1 (6%)	4 (25%)	1 (6%)	1 (7%)	0 (0%)	0 (0%)	0 (0%)	1 (8%)	0 (0%)	1 (4%)	1 (4%)	4 (14%)	2 (7%)	1 (4%)
**Day 7**	Co-amoxiclav (N = 17)					Placebo (N = 10)					Total (N = 27)				
**No Problems**	14 (82%)	16 (94%)	10 (59%)	12 (71%)	13 (81%)	9 (90%)	8 (80%)	6 (60%)	5 (50%)	7 (78%)	23 (85%)	24 (89%)	16 (59%)	17 (63%)	20 (80%)
**Moderate problems**	2 (12%)	0 (0%)	6 (35%)	4 (24%)	3 (19%)	1 (10%)	2 (20%)	4 (40%)	5 (50%)	2 (22%)	3 (11%)	2 (7%)	10 (37%)	9 (33%)	5 (20%)
**Extreme problems**	1 (6%)	1 (6%)	1 (6%)	1 (6%)	0 (0%)	0 (0%)	0 (0%)	0 (0%)	0 (0%)	0 (0%)	1 (4%)	1 (4%)	1 (4%)	1 (4%)	0 (0%)
**Day 14**	Co-amoxiclav (N = 17)					Placebo (N = 9)					Total (N = 26)				
**No Problems**	16 (94%)	16 (94%)	14 (82%)	16 (94%)	14 (93%)	9 (100%)	9 (100%)	9 (100%)	7 (78%)	8 (100%)	25 (96%)	25 (96%)	23 (88%)	23 (88%)	22 (96%)
**Moderate problems**	1 (6%)	1 (6%)	3 (18%)	1 (6%)	1 (7%)	0 (0%)	0 (0%)	0 (0%)	2 (22%)	0 (0%)	1 (4%)	1 (4%)	3 (12%)	3 (12%)	1 (4%)
**Extreme problems**	0 (0%)	0 (0%)	0 (0%)	0 (0%)	0 (0%)	0 (0%)	0 (0%)	0 (0%)	0 (0%)	0 (0%)	0 (0%)	0 (0%)	0 (0%)	0 (0%)	0 (0%)
**Day 28**	Co-amoxiclav (N = 17)					Placebo (N = 9)					Total (N = 26)				
**No Problems**	17 (100%)	17 (100%)	15 (88%)	17 (100%)	15 (94%)	9 (100%)	9 (100%)	8 (89%)	8 (89%)	7 (88%)	26 (100%)	26 (100%)	23 (88%)	25 (96%)	22 (92%)
**Moderate problems**	0 (0%)	0 (0%)	2 (12%)	0 (0%)	1 (6%)	0 (0%)	0 (0%)	1 (11%)	1 (11%)	1 (13%)	0 (0%)	0 (0%)	3 (12%)	1 (4%)	2 (8%)
**Extreme problems**	0 (0%)	0 (0%)	0 (0%)	0 (0%)	0 (0%)	0 (0%)	0 (0%)	0 (0%)	0 (0%)	0 (0%)	0 (0%)	0 (0%)	0 (0%)	0 (0%)	0 (0%)

M – mobility, SC – Self-care, UA – Usual activities, PD – Pain/ discomfort, AD – Anxiety/ depression

For peer review only - http://bmjopen.bmj.com/site/about/guidelines.xhtml

Supplemental Table 3g: Responses to CARIFS domains (only including questionnaires with sufficient data to calculate the overall CARIFS score)

		Co-amoxiclav Baseline (N = 99) Day 7 (N = 66)					Placebo Baseline (N = 97) Day 7 (N = 57)				
		No Problem	Minor Problem	Moderate Problem	Major Problem	Missing	No Problem	Minor Problem	Moderate Problem	Major Problem	Missing
Poor appetite	Baseline	21 (21%)	36 (36%)	32 (32%)	10 (10%)	0 (0%)	16 (16%)	33 (34%)	39 (40%)	9 (9%)	0 (0%)
	Day 7	38 (58%)	21 (32%)	6 (9%)	1 (2%)	0 (0%)	23 (40%)	23 (40%)	7 (12%)	4 (7%)	0 (0%)
Not sleeping well	Baseline	13 (13%)	30 (30%)	40 (40%)	16 (16%)	0 (0%)	16 (16%)	24 (25%)	38 (39%)	19 (20%)	0 (0%)
	Day 7	44 (67%)	12 (18%)	8 (12%)	2 (3%)	0 (0%)	23 (40%)	19 (33%)	8 (14%)	7 (12%)	0 (0%)
Irritable, cranky, fussy	Baseline	18 (18%)	32 (32%)	40 (40%)	9 (9%)	0 (0%)	15 (15%)	24 (25%)	45 (46%)	13 (13%)	0 (0%)
	Day 7	30 (45%)	26 (39%)	7 (11%)	3 (5%)	0 (0%)	23 (40%)	18 (32%)	11 (19%)	5 (9%)	0 (0%)
Feels unwell	Baseline	4 (4%)	29 (29%)	46 (46%)	20 (20%)	0 (0%)	9 (9%)	22 (23%)	48 (49%)	18 (19%)	0 (0%)
	Day 7	39 (59%)	18 (27%)	7 (11%)	2 (3%)	0 (0%)	28 (49%)	16 (28%)	10 (18%)	3 (5%)	0 (0%)
Low energy tired	Baseline	14 (14%)	30 (30%)	41 (41%)	14 (14%)	0 (0%)	13 (13%)	31 (32%)	41 (42%)	12 (12%)	0 (0%)
	Day 7	38 (58%)	20 (30%)	7 (11%)	1 (2%)	0 (0%)	28 (49%)	16 (28%)	10 (18%)	3 (5%)	0 (0%)
Not playing well	Baseline	27 (27%)	36 (36%)	29 (29%)	7 (7%)	0 (0%)	34 (35%)	28 (29%)	25 (26%)	10 (10%)	0 (0%)
	Day 7	50 (76%)	13 (20%)	2 (3%)	1 (2%)	0 (0%)	38 (67%)	9 (16%)	9 (16%)	1 (2%)	0 (0%)
Crying more than usual	Baseline	37 (37%)	28 (28%)	25 (25%)	9 (9%)	0 (0%)	32 (33%)	26 (27%)	27 (28%)	12 (12%)	0 (0%)
	Day 7	40 (61%)	17 (26%)	6 (9%)	3 (5%)	0 (0%)	33 (58%)	16 (28%)	6 (11%)	2 (4%)	0 (0%)
Needing extra care	Baseline	23 (23%)	34 (34%)	36 (36%)	6 (6%)	0 (0%)	20 (21%)	33 (34%)	36 (37%)	8 (8%)	0 (0%)
	Day 7	43 (65%)	19 (29%)	2 (3%)	2 (3%)	0 (0%)	32 (56%)	17 (30%)	6 (11%)	2 (4%)	0 (0%)

For peer review only - http://bmjopen.bmj.com/site/about/guidelines.xhtml

		Co-amoxiclav Baseline (N = 99) Day 7 (N = 66)					Placebo Baseline (N = 97) Day 7 (N = 57)				
		No Problem	Minor Problem	Moderate Problem	Major Problem	Missing	No Problem	Minor Problem	Moderate Problem	Major Problem	Missing
**Clinginess**	**Baseline**	16 (16%)	32 (32%)	38 (38%)	13 (13%)	0 (0%)	23 (24%)	25 (26%)	34 (35%)	15 (15%)	0 (0%)
	**Day 7**	40 (61%)	17 (26%)	7 (11%)	2 (3%)	0 (0%)	29 (51%)	16 (28%)	9 (16%)	3 (5%)	0 (0%)
**Headache**	**Baseline**	38 (38%)	20 (20%)	24 (24%)	1 (1%)	16 (16%)	44 (45%)	17 (18%)	10 (10%)	8 (8%)	18 (19%)
	**Day 7**	47 (71%)	7 (11%)	2 (3%)	1 (2%)	9 (14%)	43 (75%)	4 (7%)	3 (5%)	2 (4%)	5 (9%)
**Sore throat**	**Baseline**	29 (29%)	21 (21%)	31 (31%)	8 (8%)	10 (10%)	32 (33%)	21 (22%)	16 (16%)	15 (15%)	13 (13%)
	**Day 7**	47 (71%)	8 (12%)	3 (5%)	1 (2%)	7 (11%)	33 (58%)	12 (21%)	4 (7%)	1 (2%)	7 (12%)
**Muscle aches and pains**	**Baseline**	41 (41%)	18 (18%)	21 (21%)	5 (5%)	14 (14%)	38 (39%)	19 (20%)	11 (11%)	11 (11%)	18 (19%)
	**Day 7**	44 (67%)	10 (15%)	0 (0%)	2 (3%)	10 (15%)	38 (67%)	8 (14%)	4 (7%)	1 (2%)	6 (11%)
**Fever**	**Baseline**	15 (15%)	35 (35%)	35 (35%)	14 (14%)	0 (0%)	16 (16%)	30 (31%)	34 (35%)	17 (18%)	0 (0%)
	**Day 7**	61 (92%)	4 (6%)	1 (2%)	0 (0%)	0 (0%)	48 (84%)	4 (7%)	3 (5%)	2 (4%)	0 (0%)
**Cough**	**Baseline**	4 (4%)	18 (18%)	52 (53%)	25 (25%)	0 (0%)	3 (3%)	19 (20%)	38 (39%)	37 (38%)	0 (0%)
	**Day 7**	21 (32%)	29 (44%)	11 (17%)	5 (8%)	0 (0%)	13 (23%)	18 (32%)	15 (26%)	11 (19%)	0 (0%)
**Nasal congestion, runny nose**	**Baseline**	5 (5%)	22 (22%)	50 (51%)	22 (22%)	0 (0%)	7 (7%)	24 (25%)	41 (42%)	25 (26%)	0 (0%)
	**Day 7**	21 (32%)	33 (50%)	11 (17%)	1 (2%)	0 (0%)	16 (28%)	19 (33%)	16 (28%)	6 (11%)	0 (0%)
**Vomiting**	**Baseline**	73 (74%)	21 (21%)	3 (3%)	2 (2%)	0 (0%)	80 (82%)	9 (9%)	5 (5%)	3 (3%)	0 (0%)
	**Day 7**	62 (94%)	3 (5%)	0 (0%)	1 (2%)	0 (0%)	51 (89%)	6 (11%)	0 (0%)	0 (0%)	0 (0%)
**Not interested in what's going on**	**Baseline**	60 (61%)	26 (26%)	12 (12%)	1 (1%)	0 (0%)	55 (57%)	26 (27%)	11 (11%)	5 (5%)	0 (0%)
	**Day 7**	58 (88%)	7 (11%)	0 (0%)	1 (2%)	0 (0%)	47 (82%)	6 (11%)	3 (5%)	1 (2%)	0 (0%)

For peer review only - http://bmjopen.bmj.com/site/about/guidelines.xhtml

		Co-amoxiclav Baseline (N = 99) Day 7 (N = 66)					Placebo Baseline (N = 97) Day 7 (N = 57)				
		**No Problem**	**Minor Problem**	**Moderate Problem**	**Major Problem**	**Missing**	**No Problem**	**Minor Problem**	**Moderate Problem**	**Major Problem**	**Missing**
**Unable to get out of bed**	**Baseline**	79 (80%)	16 (16%)	4 (4%)	0 (0%)	0 (0%)	74 (76%)	14 (14%)	8 (8%)	1 (1%)	0 (0%)
	**Day 7**	62 (94%)	3 (5%)	1 (2%)	0 (0%)	0 (0%)	52 (91%)	3 (5%)	2 (4%)	0 (0%)	0 (0%)

Note: The scoring algorithm used allowed for the total score to be calculated if there was missing data in items 10-12, but not in any other items.

For peer review only

For peer review only - http://bmjopen.bmj.com/site/about/guidelines.xhtml

Supplemental Table 3h: Responses to CARIFS domains for children less than 2 years old (only including questionnaires with sufficient data to calculate the overall CARIFS score)

		**Co-amoxiclav** Baseline (N = 27) Day 7 (N = 21)					**Placebo** Baseline (N = 29) Day 7 (N = 23)				
		**No Problem**	**Minor Problem**	**Moderate Problem**	**Major Problem**	**Missing**	**No Problem**	**Minor Problem**	**Moderate Problem**	**Major Problem**	**Missing**
**Poor appetite**	**Baseline**	7 (26%)	10 (37%)	7 (26%)	3 (11%)	0 (0%)	4 (14%)	13 (45%)	9 (31%)	3 (10%)	0 (0%)
	**Day 7**	14 (67%)	4 (19%)	3 (14%)	0 (0%)	0 (0%)	11 (48%)	7 (30%)	3 (13%)	2 (9%)	0 (0%)
**Not sleeping well**	**Baseline**	3 (11%)	12 (44%)	7 (26%)	5 (19%)	0 (0%)	4 (14%)	7 (24%)	12 (41%)	6 (21%)	0 (0%)
	**Day 7**	13 (62%)	5 (24%)	3 (14%)	0 (0%)	0 (0%)	10 (43%)	7 (30%)	3 (13%)	3 (13%)	0 (0%)
**Irritable, cranky, fussy**	**Baseline**	5 (19%)	10 (37%)	10 (37%)	2 (7%)	0 (0%)	2 (7%)	5 (17%)	17 (59%)	5 (17%)	0 (0%)
	**Day 7**	12 (57%)	6 (29%)	3 (14%)	0 (0%)	0 (0%)	8 (35%)	7 (30%)	7 (30%)	1 (4%)	0 (0%)
**Feels unwell**	**Baseline**	3 (11%)	11 (41%)	7 (26%)	6 (22%)	0 (0%)	2 (7%)	6 (21%)	18 (62%)	3 (10%)	0 (0%)
	**Day 7**	12 (57%)	6 (29%)	3 (14%)	0 (0%)	0 (0%)	9 (39%)	9 (39%)	5 (22%)	0 (0%)	0 (0%)
**Low energy tired**	**Baseline**	7 (26%)	12 (44%)	5 (19%)	3 (11%)	0 (0%)	2 (7%)	12 (41%)	14 (48%)	1 (3%)	0 (0%)
	**Day 7**	15 (71%)	6 (29%)	0 (0%)	0 (0%)	0 (0%)	11 (48%)	6 (26%)	6 (26%)	0 (0%)	0 (0%)
**Not playing well**	**Baseline**	12 (44%)	9 (33%)	3 (11%)	3 (11%)	0 (0%)	10 (34%)	11 (38%)	7 (24%)	1 (3%)	0 (0%)
	**Day 7**	16 (76%)	4 (19%)	1 (5%)	0 (0%)	0 (0%)	16 (70%)	3 (13%)	4 (17%)	0 (0%)	0 (0%)
**Crying more than usual**	**Baseline**	7 (26%)	10 (37%)	5 (19%)	5 (19%)	0 (0%)	4 (14%)	8 (28%)	13 (45%)	4 (14%)	0 (0%)
	**Day 7**	12 (57%)	7 (33%)	1 (5%)	1 (5%)	0 (0%)	12 (52%)	7 (30%)	4 (17%)	0 (0%)	0 (0%)
**Needing extra care**	**Baseline**	7 (26%)	10 (37%)	8 (30%)	2 (7%)	0 (0%)	4 (14%)	11 (38%)	13 (45%)	1 (3%)	0 (0%)
	**Day 7**	14 (67%)	7 (33%)	0 (0%)	0 (0%)	0 (0%)	11 (48%)	6 (26%)	5 (22%)	1 (4%)	0 (0%)
**Clinginess**	**Baseline**	2 (7%)	12 (44%)	9 (33%)	4 (15%)	0 (0%)	2 (7%)	6 (21%)	14 (48%)	7 (24%)	0 (0%)
	**Day 7**	10 (48%)	7 (33%)	4 (19%)	0 (0%)	0 (0%)	6 (26%)	8 (35%)	7 (30%)	2 (9%)	0 (0%)
**Headache**	**Baseline**	14 (52%)	2 (7%)	1 (4%)	0 (0%)	10 (37%)	14 (48%)	2 (7%)	1 (3%)	1 (3%)	11 (38%)
	**Day 7**	15 (71%)	2 (10%)	0 (0%)	0 (0%)	4 (19%)	15 (65%)	1 (4%)	2 (9%)	0 (0%)	5 (22%)
**Sore throat**	**Baseline**	11 (41%)	3 (11%)	5 (19%)	0 (0%)	8 (30%)	8 (28%)	4 (14%)	4 (14%)	4 (14%)	9 (31%)
	**Day 7**	15 (71%)	1 (5%)	1 (5%)	0 (0%)	4 (19%)	12 (52%)	5 (22%)	1 (4%)	0 (0%)	5 (22%)

For peer review only

For peer review only - http://bmjopen.bmj.com/site/about/guidelines.xhtml

		Co-amoxiclav<br>Baseline (N = 27)<br>Day 7 (N = 21)					Placebo<br>Baseline (N = 29)<br>Day 7 (N = 23)				
		**No Problem**	**Minor Problem**	**Moderate Problem**	**Major Problem**	**Missing**	**No Problem**	**Minor Problem**	**Moderate Problem**	**Major Problem**	**Missing**
**Muscle aches and pains**	**Baseline**	14 (52%)	3 (11%)	1 (4%)	0 (0%)	9 (33%)	10 (34%)	2 (7%)	3 (10%)	1 (3%)	13 (45%)
	**Day 7**	16 (76%)	1 (5%)	0 (0%)	0 (0%)	4 (19%)	15 (65%)	1 (4%)	1 (4%)	0 (0%)	6 (26%)
**Fever**	**Baseline**	9 (33%)	6 (22%)	7 (26%)	5 (19%)	0 (0%)	5 (17%)	10 (34%)	11 (38%)	3 (10%)	0 (0%)
	**Day 7**	19 (90%)	2 (10%)	0 (0%)	0 (0%)	0 (0%)	20 (87%)	3 (13%)	0 (0%)	0 (0%)	0 (0%)
**Cough**	**Baseline**	3 (11%)	6 (22%)	9 (33%)	9 (33%)	0 (0%)	1 (3%)	6 (21%)	14 (48%)	8 (28%)	0 (0%)
	**Day 7**	7 (33%)	11 (52%)	2 (10%)	1 (5%)	0 (0%)	5 (22%)	4 (17%)	9 (39%)	5 (22%)	0 (0%)
**Nasal congestion, runny nose**	**Baseline**	1 (4%)	8 (30%)	13 (48%)	5 (19%)	0 (0%)	1 (3%)	6 (21%)	15 (52%)	7 (24%)	0 (0%)
	**Day 7**	9 (43%)	8 (38%)	3 (14%)	1 (5%)	0 (0%)	3 (13%)	4 (17%)	12 (52%)	4 (17%)	0 (0%)
**Vomiting**	**Baseline**	21 (78%)	4 (15%)	0 (0%)	2 (7%)	0 (0%)	22 (76%)	3 (10%)	3 (10%)	1 (3%)	0 (0%)
	**Day 7**	20 (95%)	1 (5%)	0 (0%)	0 (0%)	0 (0%)	20 (87%)	3 (13%)	0 (0%)	0 (0%)	0 (0%)
**Not interested in what's going on**	**Baseline**	19 (70%)	4 (15%)	4 (15%)	0 (0%)	0 (0%)	17 (59%)	10 (34%)	2 (7%)	0 (0%)	0 (0%)
	**Day 7**	18 (86%)	3 (14%)	0 (0%)	0 (0%)	0 (0%)	18 (78%)	3 (13%)	2 (9%)	0 (0%)	0 (0%)
**Unable to get out of bed**	**Baseline**	23 (85%)	3 (11%)	1 (4%)	0 (0%)	0 (0%)	26 (90%)	2 (7%)	1 (3%)	0 (0%)	0 (0%)
	**Day 7**	21 (100%)	0 (0%)	0 (0%)	0 (0%)	0 (0%)	22 (96%)	1 (4%)	0 (0%)	0 (0%)	0 (0%)

Note: The scoring algorithm used allowed for the total score to be calculated if there was missing data in items 10-12, but not in any other items.

For peer review only - http://bmjopen.bmj.com/site/about/guidelines.xhtml

Supplemental Table 3i: Responses to CARIFS domains for children aged 2+ years (only including questionnaires with sufficient data to calculate the overall CARIFS score)

		**Co-amoxiclav** Baseline (N = 72) Day 7 (N = 45)					**Placebo** Baseline (N = 68) Day 7 (N = 34)				
		**No Problem**	**Minor Problem**	**Moderate Problem**	**Major Problem**	**Missing**	**No Problem**	**Minor Problem**	**Moderate Problem**	**Major Problem**	**Missing**
**Poor appetite**	**Baseline**	14 (19%)	26 (36%)	25 (35%)	7 (10%)	0 (0%)	12 (18%)	20 (29%)	30 (44%)	6 (9%)	0 (0%)
	**Day 7**	24 (53%)	17 (38%)	3 (7%)	1 (2%)	0 (0%)	12 (35%)	16 (47%)	4 (12%)	2 (6%)	0 (0%)
**Not sleeping well**	**Baseline**	10 (14%)	18 (25%)	33 (46%)	11 (15%)	0 (0%)	12 (18%)	17 (25%)	26 (38%)	13 (19%)	0 (0%)
	**Day 7**	31 (69%)	7 (16%)	5 (11%)	2 (4%)	0 (0%)	13 (38%)	12 (35%)	5 (15%)	4 (12%)	0 (0%)
**Irritable, cranky, fussy**	**Baseline**	13 (18%)	22 (31%)	30 (42%)	7 (10%)	0 (0%)	13 (19%)	19 (28%)	28 (41%)	8 (12%)	0 (0%)
	**Day 7**	18 (40%)	20 (44%)	4 (9%)	3 (7%)	0 (0%)	15 (44%)	11 (32%)	4 (12%)	4 (12%)	0 (0%)
**Feels unwell**	**Baseline**	1 (1%)	18 (25%)	39 (54%)	14 (19%)	0 (0%)	7 (10%)	16 (24%)	30 (44%)	15 (22%)	0 (0%)
	**Day 7**	27 (60%)	12 (27%)	4 (9%)	2 (4%)	0 (0%)	19 (56%)	7 (21%)	5 (15%)	3 (9%)	0 (0%)
**Low energy tired**	**Baseline**	7 (10%)	18 (25%)	36 (50%)	11 (15%)	0 (0%)	11 (16%)	19 (28%)	27 (40%)	11 (16%)	0 (0%)
	**Day 7**	23 (51%)	14 (31%)	7 (16%)	1 (2%)	0 (0%)	17 (50%)	10 (29%)	4 (12%)	3 (9%)	0 (0%)
**Not playing well**	**Baseline**	15 (21%)	27 (38%)	26 (36%)	4 (6%)	0 (0%)	24 (35%)	17 (25%)	18 (26%)	9 (13%)	0 (0%)
	**Day 7**	34 (76%)	9 (20%)	1 (2%)	1 (2%)	0 (0%)	22 (65%)	6 (18%)	5 (15%)	1 (3%)	0 (0%)
**Crying more than usual**	**Baseline**	30 (42%)	18 (25%)	20 (28%)	4 (6%)	0 (0%)	28 (41%)	18 (26%)	14 (21%)	8 (12%)	0 (0%)
	**Day 7**	28 (62%)	10 (22%)	5 (11%)	2 (4%)	0 (0%)	21 (62%)	9 (26%)	2 (6%)	2 (6%)	0 (0%)
**Needing extra care**	**Baseline**	16 (22%)	24 (33%)	28 (39%)	4 (6%)	0 (0%)	16 (24%)	22 (32%)	23 (34%)	7 (10%)	0 (0%)
	**Day 7**	29 (64%)	12 (27%)	2 (4%)	2 (4%)	0 (0%)	21 (62%)	11 (32%)	1 (3%)	1 (3%)	0 (0%)

For peer review only

		Co-amoxiclav<br>Baseline (N = 72)<br>Day 7 (N = 45)					Placebo<br>Baseline (N = 68)<br>Day 7 (N = 34)				
		**No Problem**	**Minor Problem**	**Moderate Problem**	**Major Problem**	**Missing**	**No Problem**	**Minor Problem**	**Moderate Problem**	**Major Problem**	**Missing**
**Clinginess**	**Baseline**	14 (19%)	20 (28%)	29 (40%)	9 (13%)	0 (0%)	21 (31%)	19 (28%)	20 (29%)	8 (12%)	0 (0%)
	**Day 7**	30 (67%)	10 (22%)	3 (7%)	2 (4%)	0 (0%)	23 (68%)	8 (24%)	2 (6%)	1 (3%)	0 (0%)
**Headache**	**Baseline**	24 (33%)	18 (25%)	23 (32%)	1 (1%)	6 (8%)	30 (44%)	15 (22%)	9 (13%)	7 (10%)	7 (10%)
	**Day 7**	32 (71%)	5 (11%)	2 (4%)	1 (2%)	5 (11%)	28 (82%)	3 (9%)	1 (3%)	2 (6%)	0 (0%)
**Sore throat**	**Baseline**	18 (25%)	18 (25%)	26 (36%)	8 (11%)	2 (3%)	24 (35%)	17 (25%)	12 (18%)	11 (16%)	4 (6%)
	**Day 7**	32 (71%)	7 (16%)	2 (4%)	1 (2%)	3 (7%)	21 (62%)	7 (21%)	3 (9%)	1 (3%)	2 (6%)
**Muscle aches and pains**	**Baseline**	27 (38%)	15 (21%)	20 (28%)	5 (7%)	5 (7%)	28 (41%)	17 (25%)	8 (12%)	10 (15%)	5 (7%)
	**Day 7**	28 (62%)	9 (20%)	0 (0%)	2 (4%)	6 (13%)	23 (68%)	7 (21%)	3 (9%)	1 (3%)	0 (0%)
**Fever**	**Baseline**	6 (8%)	29 (40%)	28 (39%)	9 (13%)	0 (0%)	11 (16%)	20 (29%)	23 (34%)	14 (21%)	0 (0%)
	**Day 7**	42 (93%)	2 (4%)	1 (2%)	0 (0%)	0 (0%)	28 (82%)	1 (3%)	3 (9%)	2 (6%)	0 (0%)
**Cough**	**Baseline**	1 (1%)	12 (17%)	43 (60%)	16 (22%)	0 (0%)	2 (3%)	13 (19%)	24 (35%)	29 (43%)	0 (0%)
	**Day 7**	14 (31%)	18 (40%)	9 (20%)	4 (9%)	0 (0%)	8 (24%)	14 (41%)	6 (18%)	6 (18%)	0 (0%)
**Nasal congestion, runny nose**	**Baseline**	4 (6%)	14 (19%)	37 (51%)	17 (24%)	0 (0%)	6 (9%)	18 (26%)	26 (38%)	18 (26%)	0 (0%)
	**Day 7**	12 (27%)	25 (56%)	8 (18%)	0 (0%)	0 (0%)	13 (38%)	15 (44%)	4 (12%)	2 (6%)	0 (0%)
**Vomiting**	**Baseline**	52 (72%)	17 (24%)	3 (4%)	0 (0%)	0 (0%)	58 (85%)	6 (9%)	2 (3%)	2 (3%)	0 (0%)
	**Day 7**	42 (93%)	2 (4%)	0 (0%)	1 (2%)	0 (0%)	31 (91%)	3 (9%)	0 (0%)	0 (0%)	0 (0%)
**Not interested in what's going on**	**Baseline**	41 (57%)	22 (31%)	8 (11%)	1 (1%)	0 (0%)	38 (56%)	16 (24%)	9 (13%)	5 (7%)	0 (0%)
	**Day 7**	40 (89%)	4 (9%)	0 (0%)	1 (2%)	0 (0%)	29 (85%)	3 (9%)	1 (3%)	1 (3%)	0 (0%)

For peer review only

For peer review only - http://bmjopen.bmj.com/site/about/guidelines.xhtml

		Co-amoxiclav<br>Baseline (N = 72)<br>Day 7 (N = 45)					Placebo<br>Baseline (N = 68)<br>Day 7 (N = 34)				
		**No Problem**	**Minor Problem**	**Moderate Problem**	**Major Problem**	**Missing**	**No Problem**	**Minor Problem**	**Moderate Problem**	**Major Problem**	**Missing**
**Unable to get out of bed**	**Baseline**	56 (78%)	13 (18%)	3 (4%)	0 (0%)	0 (0%)	48 (71%)	12 (18%)	7 (10%)	1 (1%)	0 (0%)
	**Day 7**	41 (91%)	3 (7%)	1 (2%)	0 (0%)	0 (0%)	30 (88%)	2 (6%)	2 (6%)	0 (0%)	0 (0%)

Note: The scoring algorithm used allowed for the total score to be calculated if there was missing data in items 10-12, but not in any other items.

For peer review only - http://bmjopen.bmj.com/site/about/guidelines.xhtml

Supplemental File 4: Additional summaries of research use

Supplemental Table 4a: Details of all reported hospital admissions

	Co-amoxiclav (N=133)	Placebo (N=132)	Total (N=265)
**Hospital admissions**			
**0**	126 (95%)	125 (95%)	251 (95%)
**1**	6 (5%)	6 (5%)	12 (5%)
**2**	1 (1%)	1 (1%)	2 (1%)
**Total nights in hospital***			
**0**	126 (95%)	125 (95%)	251 (95%)
**1**	4 (3%)	1 (1%)	5 (2%)
**2**	1 (1%)	3 (2%)	4 (2%)
**3**	0 (0%)	1 (1%)	1 (0%)
**7**	1 (1%)	1 (1%)	2 (1%)
**9**	1 (1%)	0 (0%)	1 (0%)
**10**	0 (0%)	1 (1%)	1 (0%)
**Antibiotics received during hospital admission***			
**0**	128 (96%)	128 (97%)	256 (97%)
**1**	4 (3%)	4 (3%)	8 (3%)
**2**	1 (1%)	0 (0%)	1 (0%)
**Other drugs received during hospital admission***			
**0**	128 (96%)	126 (95%)	254 (96%)
**1**	2 (2%)	1 (1%)	3 (1%)
**2**	1 (1%)	3 (2%)	4 (2%)
**3**	1 (1%)	2 (2%)	3 (1%)
**6**	1 (1%)	0 (0%)	1 (0%)
**X-rays during hospital admission***			
**0**	131 (98%)	129 (98%)	260 (98%)
**1**	1 (1%)	3 (2%)	4 (2%)
**2**	1 (1%)	0 (0%)	1 (0%)
**Other investigations during hospital admission****			
**0**	131 (98%)	130 (98%)	261 (98%)
**1**	2 (2%)	2 (2%)	4 (2%)
**Emergency department visits**			
**0**	128 (96%)	127 (96%)	255 (96%)
**1**	5 (4%)	5 (4%)	10 (4%)

**The summaries encompass the entire 28-day follow-up period, i.e. participants may have had a total of two x-rays made up of x-rays during two separate hospital admissions. Similarly, the total nights in hospital may have accumulated from more than one hospital admission.*

[a] Participants who were reported to have been referred to the hospital team/ emergency department for acute admission during a re-consultation for the same illness episode for which the child was randomised, but for whom no hospital admission is recorded at this date, are classed as having visited emergency department.
Hospital admission episodes for at least one night were collected during the notes review.
The trial did not report any admissions to intensive care units (ICU).
Two participants were reported to have a hospital admission on the same day as their discharge day; these were counted as emergency department visits.
***Other investigations include blood samples (2), throat swap and nasal aspirate.*

For peer review only - http://bmjopen.bmj.com/site/about/guidelines.xhtml

Supplemental Table 4b: Overview of healthcare use for re-consultations due to clinical deterioration and hospitalisations related to the illness episode for which the child was randomised to ARCHIE

Number of	N (Co-amoxiclav)	Co-amoxiclav: Mean (S.D.)	N (Placebo)	Placebo: Mean (S.D.)	N (Difference)	Difference (95% CI)
Re-consultations	133	0.27 (0.49)	132	0.23 (0.48)	265	0.04 (95% CI -0.07, 0.15), p-value = 0.495
Antibiotics received at re-consultation	133	0.10 (0.30)	132	0.10 (0.30)	265	0.00 (95% CI -0.07, 0.07), p-value = 0.993
Other drugs received at re-consultation	133	0.06 (0.27)	132	0.09 (0.49)	265	-0.03 (95% CI -0.12, 0.07), p-value = 0.535
Chest x-rays at re-consultation	133	0.03 (0.17)	132	0.01 (0.09)	265	0.02 (95% CI -0.01, 0.06), p-value = 0.178
Other interventions at re-consultation	133	0.02 (0.12)	132	0.00 (0.00)	265	0.01 (95% CI -0.01, 0.04), p-value = 0.163
Hospital admissions	133	0.04 (0.19)	132	0.05 (0.22)	265	-0.01 (95% CI -0.06, 0.03), p-value = 0.511
Total nights in hospital	133	0.09 (0.65)	132	0.14 (0.74)	265	-0.05 (95% CI -0.18, 0.08), p-value = 0.432
Antibiotics received during hospital admission	133	0.02 (0.15)	132	0.02 (0.15)	265	0.00 (95% CI -0.03, 0.03), p-value = 0.976
Other drugs received during hospital admission	133	0.05 (0.33)	132	0.09 (0.45)	265	-0.04 (95% CI -0.13, 0.05), p-value = 0.398
X-rays during hospital admission	133	0.01 (0.09)	132	0.02 (0.12)	265	-0.01 (95% CI -0.03, 0.02), p-value = 0.596
Other investigations during hospital admission	133	0.00 (0.00)	132	0.02 (0.12)	265	-0.01 (95% CI -0.04, 0.01), p-value = 0.168
Emergency department visits	133	0.03 (0.17)	132	0.03 (0.17)	265	0.00 (95% CI -0.04, 0.04), p-value = 0.983

**Differences have been adjusted for the stratification factors age (used as continuous variable) and seasonal influenza vaccination status. Clustering by centre has been accounted for using the 'cluster' option in Stata's 'regress' command and robust standard errors were generated.*

For peer review only - http://bmjopen.bmj.com/site/about/guidelines.xhtml

Supplemental File 5: Number of days children were unable to attend school or nursery, and subsequent changes to childcare requirements

Supplemental Table 5: Daily activity and child care

	Co-amoxiclav					Placebo					Difference		
	N	Mean (S.D.)	Median	IQR	Range	N	Mean (S.D.)	Median	IQR	Range	N	Difference (95% CI)*	p-value
**Days of school/ nursery missed**													
**week 1**	53	2.6 (2.2)	3	1, 4	0, 7	49	2.4 (2.2)	2	1, 3	0, 12	102	0.1 (95% CI -0.8, 1.0)	0.832
**week 2**	44	0.5 (1.5)	0	0, 0	0, 7	39	0.6 (1.5)	0	0, 0	0, 7	83	-0.2 (95% CI -0.8, 0.5)	0.630
**week 3**	36	0.3 (1.0)	0	0, 0	0, 5	33	0.1 (0.5)	0	0, 0	0, 3	69	0.1 (95% CI -0.2, 0.5)	0.492
**week 4**	38	0.0 (0.2)	0	0, 0	0, 1	26	0.2 (1.0)	0	0, 0	0, 5	64	-0.2 (95% CI -0.6, 0.2)	0.404
**Days of work missed****													
**week 1**	45	1.1 (1.3)	0	0, 2	0, 4	35	1.3 (1.4)	1	0, 2	0, 5	80	-0.2 (95% CI -0.8, 0.4)	0.463
**week 2**	41	0.1 (0.3)	0	0, 0	0, 1	33	0.2 (0.7)	0	0, 0	0, 3	74	-0.1 (95% CI -0.3, 0.1)	0.345
**week 3**	34	0.1 (0.5)	0	0, 0	0, 2	27	0.0 (0.0)	0	0, 0	0, 0	61	0.1 (95% CI -0.1, 0.3)	0.205
**week 4**	33	0.0 (0.0)	0	0, 0	0, 0	22	0.3 (1.1)	0	0, 0	0, 5	55	-0.3 (95% CI -0.7, 0.2)	0.246
**Days on which usual activities were changed*****													
**week 1**	37	2.0 (2.4)	2	0, 4	0, 7	34	2.8 (2.0)	3	1, 4	0, 7	71	-0.8 (95% CI -1.8, 0.2)	0.132
**week 2**	33	0.8 (2.1)	0	0, 0	0, 7	37	0.8 (1.6)	0	0, 0	0, 5	70	0.1 (95% CI -0.8, 1.0)	0.833
**week 3**	26	0.2 (0.7)	0	0, 0	0, 3	32	0.5 (1.7)	0	0, 0	0, 7	58	0.0 (95% CI -0.6, 0.5)	0.877
**week 4**	26	0.0 (0.2)	0	0, 0	0, 1	23	0.3 (0.8)	0	0, 0	0, 3	49	-0.2 (95% CI -0.6, 0.1)	0.150
**Days outside carer required**													
**week 1**	40	0.6 (1.2)	0	0, 1	0, 4	31	0.8 (2.1)	0	0, 1	0, 11	71	-0.3 (95% CI -1.2, 0.6)	0.520
**week 2**	40	0.3 (1.0)	0	0, 0	0, 5	34	0.1 (0.3)	0	0, 0	0, 2	74	0.2 (95% CI -0.2, 0.5)	0.344
**week 3**	34	0.1 (0.5)	0	0, 0	0, 2	34	0.1 (0.5)	0	0, 0	0, 3	68	0.0 (95% CI -0.2, 0.2)	0.933
**week 4**	34	0.2 (1.0)	0	0, 0	0, 6	30	0.1 (0.5)	0	0, 0	0, 3	64	0.1 (95% CI -0.3, 0.5)	0.637

**Differences have been adjusted for the stratification factors age (used as continuous variable) and seasonal influence vaccination status. Clustering by centre has been accounted for using the 'cluster' option in Stata's 'regress' command and robust standard errors were generated.*

***Collected for parents/ carers in paid employment; *** Collected for parents/ carers not in paid employment*

SD: Standard deviation; IQR: Interquartile range

For peer review only - http://bmjopen.bmj.com/site/about/guidelines.xhtml

BMJ Open

Quality of life, healthcare use and costs in 'at risk' children after early antibiotic treatment vs. placebo for influenza-like illness: Within-trial descriptive economic analyses of the ARCHIE randomised controlled trial

Journal:	*BMJ Open*
Manuscript ID	bmjopen-2021-049373.R1
Article Type:	Original research
Date Submitted by the Author:	24-Sep-2021

Complete List of Authors:	Rombach, Ines; University of Oxford, Nuffield Department of Population Health<br>Wang, Kay; University of Oxford Nuffield Department of Primary Care Health Sciences<br>Tonner, Sharon; University of Oxford Nuffield Department of Primary Care Health Sciences<br>Grabey, Jenna; University of Oxford Nuffield Department of Primary Care Health Sciences<br>Harnden, Anthony; University of Oxford Nuffield Department of Primary Care Health Sciences<br>Wolstenholme, Jane; Oxford University, HERC, Nuffield Department of Population Health

**Primary Subject Heading**:	Respiratory medicine
Secondary Subject Heading:	Health economics, Infectious diseases, Paediatrics
Keywords:	HEALTH ECONOMICS, INFECTIOUS DISEASES, Paediatric infectious disease & immunisation < PAEDIATRICS, PAEDIATRICS, PRIMARY CARE, RESPIRATORY MEDICINE (see Thoracic Medicine)

SCHOLARONE™
Manuscripts

I, the Submitting Author has the right to grant and does grant on behalf of all authors of the Work (as defined in the below author licence), an exclusive licence and/or a non-exclusive licence for contributions from authors who are: i) UK Crown employees; ii) where BMJ has agreed a CC-BY licence shall apply, and/or iii) in accordance with the terms applicable for US Federal Government officers or employees acting as part of their official duties; on a worldwide, perpetual, irrevocable, royalty-free basis to BMJ Publishing Group Ltd ("BMJ") its licensees and where the relevant Journal is co-owned by BMJ to the co-owners of the Journal, to publish the Work in this journal and any other BMJ products and to exploit all rights, as set out in our licence.

The Submitting Author accepts and understands that any supply made under these terms is made by BMJ to the Submitting Author unless you are acting as an employee on behalf of your employer or a postgraduate student of an affiliated institution which is paying any applicable article publishing charge ("APC") for Open Access articles. Where the Submitting Author wishes to make the Work available on an Open Access basis (and intends to pay the relevant APC), the terms of reuse of such Open Access shall be governed by a Creative Commons licence – details of these licences and which Creative Commons licence will apply to this Work are set out in our licence referred to above.

Other than as permitted in any relevant BMJ Author's Self Archiving Policies, I confirm this Work has not been accepted for publication elsewhere, is not being considered for publication elsewhere and does not duplicate material already published. I confirm all authors consent to publication of this Work and authorise the granting of this licence.

For peer review only

Quality of life, healthcare use and costs in 'at risk' children after early antibiotic treatment vs. placebo for influenza-like illness: Within-trial descriptive economic analyses of the ARCHIE randomised controlled trial

Authors

Ines Rombach (ines.rombach@ndorms.ox.ac.uk)[1],

Kay Wang (kay.wang@phc.ox.ac.uk)[2],

Sharon Tonner (sharon.tonner@phc.ox.ac.uk)[2],

Jenna Grabey (jenna.grabey@phc.ox.ac.uk)[2],

Anthony Harnden (anthony.harnden@phc.ox.ac.uk)[2],

Jane Wolstenholme (jane.wolstenholme@ndph.ox.ac.uk)[1],

for the ARCHIE Investigators*

*A complete list of ARCHIE investigators can be found at the end of the manuscript

Affiliations

[1]Health Economics Research Centre, Nuffield Department of Population Health, Oxford University, Oxford, UK

[2]Nuffield Department of Primary Care Health Sciences, University of Oxford, Oxford, UK

For peer review only

Corresponding author:

Ines Rombach

Health Economics Research Centre

Nuffield Department of Population Health

Old Road Campus

University of Oxford

Oxford

OX3 7LF

United Kingdom

ines.rombach@ndorms.ox.ac.uk

https://orcid.org/0000-0003-3464-3867

Keywords

Antibiotic use; healthcare resource use; cost; quality of life (QoL); children; paediatric research; influenza-like illness; economic analysis

Word count:

Abstract: 305

Main text (excluding figures and tables): 5500

For peer review only

Abstract

Objectives: To characterise the quality of life, healthcare use and costs associated with early antibiotic treatment of influenza-like illness (ILI) in 'at risk' children.

Design: Economic analysis of a two-arm double-blind parallel group pragmatic randomised controlled trial.

Setting: Children were recruited from community-based health care settings, including general practices, walk-in centres and hospital ambulatory care.

Participants: Children with risk factors for influenza-related complications, including respiratory, cardiac and neurological conditions, who presented within the first five days of an ILI.

Interventions: Co-amoxiclav 400/57 suspension or placebo.

Outcome measures: This economic analysis focussed on quality of life measured by the EQ-5D-Y, symptoms assessed by the Canadian Acute Respiratory Infection and Flu Scale (CARIFS), and healthcare use and costs including medication, hospital visits and admissions, GP and nurse contacts. Outcomes were assessed for up to 28 days post randomisation.

Results: Information on resource use, EQ-5D-Y (day 28) and CARIFS (day 7) was available for 265 (98%), 72 (27%) and 123 (45%) out of 271 participants, respectively. Average costs in the co-amoxiclav group were £25 lower (95% confidence interval [CI] -113 to 65), but this difference was not statistically significant (p-value: 0.566). The difference in EQ-5D-Y scores between groups was also not statistically significant (-0.014 (95% CI -0.124, 0.096), p-value: 0.798). However, day 7 CARIFS scores were 3.5 points lower in the co-amoxiclav arm (95% CI -6.9 to -0.1, p-value: 0.044).

Conclusions: Our findings did not show evidence that early co-amoxiclav treatment improves quality of life or reduces healthcare use and costs in 'at risk' children with ILI, but may reduce symptom severity though confirmation from further research would be important. Reliable data collection from children's parents/carers was challenging, and resulted in high levels of

For peer review only - http://bmjopen.bmj.com/site/about/guidelines.xhtml

missing data, which is common in pragmatic trials involving children with acute respiratory tract infections.

Trial registration: ISRCTN 70714783; EudraCT 2013-002822-21

For peer review only

For peer review only - http://bmjopen.bmj.com/site/about/guidelines.xhtml

Article Summary

Strength and limitations

- This study aimed to describe quality of life, healthcare use and costs in children with influenza-like illness (ILI) and known risk factors for complications.
- The study used pragmatic inclusion criteria, recruiting children with ILI, which reflects current clinical practice but cannot reliably distinguish between influenza infection and ILI due to other infections.
- Subgroup analyses examined differential costs between children with and without laboratory-confirmed influenza.
- Reviews of medical records provided robust information on healthcare use with negligible levels of missing data.
- Given the nature of the participant group, proxy-reported completion of instruments and questionnaires was required to inform the quality of life, healthcare use and costs, however this was subject to high levels of missing data, making a formal cost-effectiveness analysis impossible to undertake.

For peer review only

For peer review only - http://bmjopen.bmj.com/site/about/guidelines.xhtml

Background

The clinical results of the 'Early use of Antibiotics for at Risk CHildren with InfluEnza-like illness' (ARCHIE) trial were recently published (ISRCTN 70714783)[1]. This randomised controlled trial (RCT) aimed to assess uncertainty in the treatment of influenza-like illness (ILI) in 'at risk' children, i.e. children with pre-existing conditions such as asthma, diabetes mellitus and cerebral palsy.

The RCT compared the early treatment with oral co-amoxiclav with placebo, and used proportion of children re-consulting due to clinical deterioration within 28 days of randomisation as the primary outcome measure.

Full details of the trial are published elsewhere[1,2]. The primary intention to treat (ITT) analysis did not show a statistically significant difference between the co-amoxiclav and placebo groups, as measured by the proportion of children re-consulting due to clinical deterioration within 28 days. A re-consultation rate of 24.8% was observed in the co-amoxiclav arm, and 21.2% in the placebo arm (adjusted risk ratio 1.16 (95% CI 0.75, 1.80). An exploratory analysis in the subgroup with laboratory-confirmed influenza showed that a lower proportion of children in the co-amoxiclav group re-consulted due to clinical deterioration, although this subgroup did not have sufficient power to demonstrate whether this difference was statistically significant.

The trial also investigated health-related quality of life (QoL), healthcare use and cost associated with the early co-amoxiclav use in 'at risk' children. This research is important due to the absence of quality of life and healthcare use data associated with early antibiotic use associated with influenza or ILI in this important patient population. Our literature review for our pre-trial modelling work demonstrated a lack of observational data in these 'at risk' children, and we were unable to identify any clinical trials in this paediatric cohort[3-5]. Previous UK based cost-effectiveness models of antivirals for treatment of influenza in children used adult utility values based on derived values from adults treated with oseltamivir[6,7]. An

For peer review only - http://bmjopen.bmj.com/site/about/guidelines.xhtml

alternative source of child-specific proxy EQ-5D data was from a trial of corticosteroids for otitis media[8].

Methods

Design and planned analyses for the ARCHIE trial, including the health economic analysis, are described in the published protocol[2]. Specific analyses of the quality of life, healthcare use and cost data are described below. Data were collected for a UK based NHS perspective, with additional data collected to provide a wider societal perspective. The time horizon of this study is 28 days.

Study population

The ARCHIE trial recruited 271 children at increased risk of influenza-related clinical deterioration or complications. Recruited children were between the ages of six months to 12 years within the first five days of in ILI. In the remit of this pragmatic trial, ILI was defined as the presence of both cough and fever, whereby fever could either be child-reported, parent-reported or temperature >37.8°C (axillary or tympanic temperature measurement). ‘At risk’ was based on healthcare professionals’ clinical judgement and included children with pre-existing conditions such as asthma, diabetes mellitus and cerebral palsy. Children were ineligible if they had known contra-indications to co-amoxiclav, required immediate antibiotics based on clinical judgement, required immediate hospital admission or an influenza-related complication (clinical judgement), had been observed on a hospital ward or ambulatory care unit for longer than 24 hours. Full details of all inclusion and exclusion criteria are given in the trial protocol[9].

Participants were recruited from general practices, walk-in centres and hospitals in the UK with a view to making healthcare decisions in the NHS and other healthcare settings.

For peer review only - http://bmjopen.bmj.com/site/about/guidelines.xhtml

The planned sample size for the trial was 650, based on an absolute risk reduction in re-consultations due to clinical deterioration of 14% (reduction from 40% to 26%), using 90% power, a two-sided 5% significance level an inflation factor of 1.041. The full sample size was not reached, and recruitment was closed after 271 children had been randomised.

Children were randomised 1:1 to either co-amoxiclav 400/57 (amoxicillin 400 mg as trihydrate and clavulanic acid 57 mg as potassium salt/5 mL when reconstituted with water) or a matching placebo, to be taken orally twice daily for 5 days. Co-amoxiclav was used due to its susceptibility in treating lower respiratory tract bacterial isolates associated with influenza in primary care [10], and is stockpiled by the UK government for use in influenza pandemics; a matching placebo was chosen to obtain unbiased results for treatment effects[1].

Quality of life

Quality of life was measured using the EQ-5D 3-Level youth version (EQ-5D-Y), which was confirmed in pre-trial focus groups as most applicable in this patient population, compared to other questionnaires (i.e. Child Health Utility-9D)[11]. The EQ-5D-Y was collected at baseline, days 4, 7, 14 and 28. For all children, parents or carers (i.e. proxies) completed the data on the children's behalf. Data were also collected from the participants directly, where appropriate. No restrictions on which children should complete the EQ-5D-Y themselves were included in the protocol. This was due to some of the participants having complex needs and therefore being unable to complete questionnaires regardless of their age. Equally, severe illness may have prevented some children who would usually be able to complete the data from doing so at some trial follow-up time points. Whether or not children were asked to complete questionnaires was left to their proxy's discretion.

The EQ-5D-Y consists of five questions covering mobility, self-care, usual activities, pain/ discomfort, and anxiety/ depression. Each question has three response levels; 'no', 'some', 'a

For peer review only - http://bmjopen.bmj.com/site/about/guidelines.xhtml

lot'. As the scoring for the youth-version had not been published at the time of the analysis of this study[11,12], the responses to the five questions were converted into utilities using the EQ-5D-3L algorithm for the UK value set[13-15], ranging from -0.594 to 1, where 1 indicates perfect health.

The health state utilities calculated from the responses to EQ-5D-Y were used to estimate mean QALYs rated on behalf of the child and by the child for both trial arms at 28 days. This was done using an area under the curve (AUC) approach, whereby utilities were assumed to change linearly between each follow-up time point, weighted by the time periods the questionnaires were administered, i.e. days 4, 7, 14 and 28. QALYs were generated only for participants who had EQ-5D-Y data available at baseline and all follow-up time points to 28 days.

Canadian Acute Respiratory Infection and Flu Scale (CARIFS)

The Canadian Acute Respiratory Infection and Flu Scale (CARIFS)[16] consists of 18 questions covering three domains, i.e. symptoms, function and parental impact. The questions have 4-point ordinal scale, from no problems to major problems, scored 0 to 3. A total score ranging from 0 (best possible health) to 54 (worst possible health) is calculated by adding up all 18 items. Missing data in the three questions about headache, sore throat, muscle aches or pains were handled by mean imputation, in line with the scoring instructions[16]. The CARIFS was collected at baseline and 1 week.

Healthcare use

Proxy-reported healthcare use was collected in the study diaries with regards to medication use, hospital visits and admissions, GP and nurse contacts. Information on the number of days children were unable to attend school or nursery, and subsequent changes to childcare

For peer review only - http://bmjopen.bmj.com/site/about/guidelines.xhtml

requirements were also collected. These data were collected in weekly diaries on days 7, 14, 21 and 28, requiring recall of health care resource use over the previous week.

A notes review was conducted to obtain clinical data for the primary outcome (re-consultation due to clinical deterioration), medication prescriptions and/or further investigations, and hospitalisations or deaths within 28 days of randomisation. The number of re-consultations, antibiotics and other drugs given, chest x-rays and other investigations performed were included in this review and were used in the estimation of healthcare use.

For the purposes of this analysis, only re-consultations due to clinical deterioration considered as related to the initial illness episode for which the child was randomised were included, in line with the primary endpoint of the trial. Re-consultations had to take place within 28 days from randomisation and in relation to worsening symptoms, new symptoms or complications for which the child required medication, investigation or referral to hospital.

Referrals to hospital or emergency departments for acute admission were recorded from the notes review including information on hospital inpatient admissions, medications and investigations undertaken.

The study's clinical lead reviewed all hospital admissions, and distinguished between those considered to be related to the illness episode the child was randomised for, and those not related. They also reviewed all medication recorded. Medications deemed unrelated to the illness for which the child was recruited into the trial were identified and excluded from this analysis.

Information on healthcare use was restricted to the information obtained via the notes review. Information from the carer-completed diaries was considered insufficient for inclusion in this analysis (details on data availability for the self-reported questionnaires are provided in the results section for information). Serious Adverse Events as reported by the recruiting sites were reviewed to ensure all relevant health care use was captured through the notes review.

For peer review only - http://bmjopen.bmj.com/site/about/guidelines.xhtml

Unit costs

Costs were for the UK NHS perspective.

Participating children were recruited from GP practices and hospital-based ambulatory assessment units. Unit costs for GP practice visits or re-consultations with a GP were obtained from the PSSRU Unit Costs of Health and Social Care compendium for 2017/2018[17]. Unit costs for all other health service contacts were derived from the NHS National Schedule of Reference Costs 2017 to 2018[18], including re-consultations for participants recruited in paediatric ambulatory assessment units, which were costed as a visit to a paediatric ambulatory assessment unit.

Investigations, including blood tests, nasal aspirate, and throat swabs were costed based on information on Directly Accessed Pathology Services in the NHS National Schedule of Reference Costs 2017 to 2018[18]. Costs of urine tests were based on information published by Fenwick et al[19].

Antibiotics and other prescription drug costs were based on the British National Formulary (BNF for children) and UK tariff costs[20], assuming the minimum value reported. Where possible, unit costs were applied based on the dose reported, or a median dose if no dose was reported; cost for inhalers and eye/nose drops were based on the minimum unit sold.

Information on the unit costs included in this trial are presented in Supplemental File 1.

Due to the 28-day time horizon, no discounting was used.

Analysis

Within-trial analysis

Summaries were generated on an as randomised basis, with participants analysed within their randomised groups regardless of compliance with their allocated interventions. All available data were used, only participants whose parents or carers withdrew consent for extraction of

For peer review only - http://bmjopen.bmj.com/site/about/guidelines.xhtml

data from their children's medical record, or where access to medical notes was not possible for other reasons were excluded from the summaries. No imputations were performed for missing data.

Data availability was summarised for the EQ-5D-3L (completed by the child, and on their behalf (proxy)), CARIFS, availability of daily activity and childcare questionnaire, and availability of health service contact (proxy-reported).

EQ-5D-Y-3-level, QALYs and CARIFS data were summarised descriptively by data collection time point. Healthcare use was summarised using number of events and frequencies to indicate how often participants had used each service. Mean number of contacts with different health services were also calculated. Costs incurred for the different health service contacts were calculated by applying the unit costs, described in Supplemental File 1. Information on the number of days children were unable to attend school or nursery, and subsequent changes to childcare requirements reported during each of the follow-up weeks were also summarised.

Differences between trial arms were estimated using linear regression models, adjusted for the minimisation factors; age (used as continuous variable) and seasonal influenza vaccination status. Clustering by the recruiting site (i.e. the GP practices or hospital ambulatory units at which participants were randomised) was accounted for using the 'cluster' option in Stata's 'regress' command and robust standard errors were generated.

Additional analyses and sensitivity analyses

Subgroup analyses explored if treatment effects varied depending on whether the children's illness at baseline was laboratory-confirmed to be influenza, by adding an interaction term between treatment allocation and laboratory-confirmed influenza at baseline to the above described analysis model. Subgroup analyses were performed for the total costs incurred over the trial follow-up period, and separately for the costs incurred through medication use, and the costs related to re-consultations, hospital admissions and referrals. Results from this

For peer review only - http://bmjopen.bmj.com/site/about/guidelines.xhtml

investigation were presented as forest plots. As this analysis was exploratory, no p-values were displayed.

Sensitivity analysis examined the effect of including costs associated with hospitalisations unrelated to the illness episode for which the child was recruited to ARCHIE.

Extrapolation of findings to national scale

The costs were extrapolated to estimate the impact of either intervention on a national scale. The number of children within the relevant age category in the UK were estimated from information for 2018 from the Office for National Statistics[21]. The number of GP consultations during 2018/2019 was estimated from the 'Surveillance of influenza and other respiratory viruses in the UK'[22]. Finally, the proportions of children who consult with GPs for influenza like illnesses who correspond to the ARCHIE trial population were estimated from the current literature[9].

From these figures, the total number of 'at risk' children likely to consult a GP with influenza like illnesses will be estimated for the UK. This figure was combined with the estimations of cost differences between the co-amoxiclav and placebo groups, to estimate the likely monetary impact of the co-amoxiclav intervention on a national scale.

Patient and public involvement

The research team conducted consultations with parents of 'at risk' children and young people from the National Institute for Health Research Generation R Young Person's Advisory Group to inform the design and conduct of the ARCHIE programme. The ARCHIE Programme Steering Committee also included a patient representative.

For peer review only - http://bmjopen.bmj.com/site/about/guidelines.xhtml

Results

Data availability

In total, 271 children were randomised into the trial, 136 to co-amoxiclav, 135 to placebo. Details of the study population characteristics are provided in Table 1. Six children were excluded. Of these, five children were withdrawn from the trial and all follow-up, and medical notes of one child could not be accessed. As such, 265 children were included in the analysis (133 randomised to co-amoxiclav, 132 to placebo). All 265 children received their allocated intervention, and there were no cross-overs between the treatment arms. Data on healthcare resource use based on note reviews were available for all 265 children.

For peer review only - http://bmjopen.bmj.com/site/about/guidelines.xhtml

Table 1: Characteristics of the study population

	Co-amoxiclav (N=136)	Placebo (N=135)	Total (N=271)
**Age Category (by EudraCT guidelines)**[1]			
Infants (28 days-23 months)	45 (33%)	44 (33%)	89 (33%)
Children (2-11 years)	89 (65%)	90 (67%)	179 (66%)
Adolescents (12-17 years)	2 (1%)	1 (1%)	3 (3%)
**Age in months**[2]	36.4 (20.9, 70.8)	40.8 (19.4, 85.6)	39.3 (20.2, 78.2)
**Female**[1]	53 (39.0%)	55 (40.7%)	108 (39.9%)
**Number of acute consultations in the 12 month period before entering the study**[2]	6 (3 to 10); n=100	5 (3 to 9); n=102	6 (3 to 9); n=202
**Antibiotics prescribed in preceding 3 months**[1]	33/128 (%)	25/130 (%)	58/258 (%)
**'at risk' category (not mutually exclusive)**[1]			
Respiratory	99 (72.8%)	99 (73.3%)	198 (73%)
Neurological	6 (4.4%)	9 (6.7%)	15(6%)
Cardiac	12 (8.8%)	4 (3.0%)	16 (6%)
Renal	3 (2.2%)	0	3 (1%)
Immunodeficiency	1 (0.7%)	0	1 (0%)
Genetic	9 (28.1%)	9 (22.5%)	18 (25%)
Metabolic	1 (3.1%)	5 (12.5%)	6 (8%)
Premature birth	13 (40.6%)	15 (37.5%)	28 (39%)
Previous recurrent or serious respiratory problems	6 (18.8%)	8 (20.0%)	14 (19%)
Other (allergies, Hx astro astana)	3 (9.4%)	3 (7.5%)	6 (8%)
**Duration of illness (days)**[2]	2.7 (1.2)	2.7 (1.2)	2.7 (1.2)

Note: Denominators are given for variables with missing data.
[1]Frequency (%)
[2]median (interquartile range)

For peer review only

Proxy-completed EQ-5D-Y data were available for 75% (204/271) of children at baseline, and 27% (72/271) at 28 days. Self-completed EQ-5D-Y data were available for 23% (61/271) at baseline, and 10% (27/271) at 28 days. CARIFS data were completed for 72% (196/271) of children at baseline and 45% (123/271) at day 7. Proxy-reported data for daily activity and childcare questionnaire, and the health service contact questions were available for 54% (146/271) at baseline, and less than 35% at 28 days respectively (92/271 daily activity and childcare questionnaires and 87/271 healthcare use questionnaires were received). Table 2 shows this information by treatment arm, and for all follow-up points. Additional information on data availability, including by age group, and EQ-5D-Y missing data pattern over time are available in Supplemental File 2.

For peer review only

For peer review only - http://bmjopen.bmj.com/site/about/guidelines.xhtml

Table 2: Overview of availability for self-reported data

	Co-amoxiclav (N=136)	Placebo (N=135)	Total (N=271)
**Availability of EQ-5D-Y index completed on behalf of child**			
**day 0**	96 (71%)	108 (80%)	204 (75%)
**day 4**	52 (38%)	52 (39%)	104 (38%)
**day 7**	52 (38%)	54 (40%)	106 (39%)
**day 14**	44 (32%)	46 (34%)	90 (33%)
**day 28**	41 (30%)	31 (23%)	72 (27%)
**Availability of EQ-5D VAS completed on behalf child**			
**day 0**	120 (88%)	129 (96%)	249 (92%)
**day 4**	77 (57%)	76 (56%)	153 (56%)
**day 7**	78 (57%)	73 (54%)	151 (56%)
**day 14**	61 (45%)	57 (42%)	118 (44%)
**day 28**	53 (39%)	40 (30%)	93 (34%)
**Availability of EQ-5D-Y index completed by child**			
**day 0**	32 (24%)	29 (21%)	61 (23%)
**day 4**	16 (12%)	13 (10%)	29 (11%)
**day 7**	17 (13%)	11 (8%)	28 (10%)
**day 14**	17 (13%)	10 (7%)	27 (10%)
**day 28**	17 (13%)	10 (7%)	27 (10%)
**Availability of EQ-5D VAS completed by child**			
**day 0**	28 (21%)	25 (19%)	53 (20%)
**day 4**	19 (14%)	13 (10%)	32 (12%)
**day 7**	19 (14%)	11 (8%)	30 (11%)
**day 14**	20 (15%)	10 (7%)	30 (11%)
**day 28**	18 (13%)	11 (8%)	29 (11%)
**Availability of CARIFS**			
**day 0**	99 (73%)	97 (72%)	196 (72%)
**day 7**	66 (49%)	57 (42%)	123 (45%)
**Availability of CARIFS VAS**			
**day 0**	133 (98%)	135 (100%)	268 (99%)
**day 7**	76 (56%)	74 (55%)	150 (55%)
**Availability of daily activity and childcare questionnaire**			
**day 7**	75 (55%)	71 (53%)	146 (54%)
**day 14**	61 (45%)	57 (42%)	118 (44%)
**day 21**	54 (40%)	46 (34%)	100 (37%)
**day 28**	53 (39%)	39 (29%)	92 (34%)
**Availability of healthcare use questionnaire**			
**day 7**	76 (56%)	70 (52%)	146 (54%)
**day 14**	56 (41%)	52 (39%)	108 (40%)

For peer review only

For peer review only - http://bmjopen.bmj.com/site/about/guidelines.xhtml

**day 21**	51 (38%)	45 (33%)	96 (35%)
**day 21**	51 (38%)	45 (33%)	96 (35%)
**day 28**	49 (36%)	38 (28%)	87 (32%)

Note: EQ-5D-Y and CARIFS were included as 'available' if all relevant questions were completed; the daily activities and childcare questionnaire, and the health service contact questionnaire were considered 'available' if at least one question had been answered, including indicating that the daily activities and childcare questions were not applicable.

For peer review only

Quality of life and healthcare use

Data for the EQ-5D-Y, QALYs and CARIFS are shown in Table 3. There were no statistically significant differences between the treatment arms for the EQ-5D-Y and QALY. The EQ-5D VAS completed on behalf of the children was statistically significantly higher, indicating higher quality of life, in the co-amoxiclav arm at 7 days post randomisation only. The CARIFS score was significantly lower in the co-amoxiclav arm, indicating lower severity of respiratory symptoms in those children. Reponses to the individual EQ-5D-Y and CARIFS items, for the whole cohort and split by age group, are presented in Supplemental File 3.

Details of healthcare use for related re-consultations due to clinical deterioration, and hospital admissions related to the illness episode for which the child was randomised into ARCHIE are shown in Table 4. Overall, 23% of children (61/265) had at least one re-consultation due to clinical deterioration, and hospital admissions related to the illness episode for which the child was randomised into ARCHIE were reported for 5% of children (12/265). Additional information on all hospital admissions and average healthcare use, as typically presented in cost-effectiveness analyses, are shown in Supplemental File 4. Resource use was similar between the treatment arms.

Costs per participant associated with related re-consultations and hospital admissions are shown in Table 5. Average total costs over 28-day follow-up period were estimated as £82 (SD 481) in the co-amoxiclav arm, and £117 (SD 540) in the placebo arm. The medians and IQRs of zero for most costing items reflect the fact that 75% of the participants did not use the relevant resources and hence did not incur corresponding costs. There were no statistically significant differences in total costs, or any of the cost-components, between the treatment groups. Hospital stays appear to be the main cost drivers.

For peer review only

For peer review only - http://bmjopen.bmj.com/site/about/guidelines.xhtml

Information on the number of days children were unable to attend school or nursery, and subsequent changes to childcare requirements are summarised in supplemental File 5. There were no statistically significant differences between the groups.

For peer review only

For peer review only - http://bmjopen.bmj.com/site/about/guidelines.xhtml

Table 3: EQ-5D-Y, quality adjusted life scores and CARIF outcomes

	Co-amoxiclav:		Placebo		Difference	
	N	Mean (S.D.)	N	Mean (S.D.)	Total N	Difference (95% CI)*
**EQ-5D-Y proxy, completed on behalf of child**						
Day 0	96	0.591 (0.342)	108	0.568 (0.386)		
Day 4	52	0.630 (0.349)	52	0.690 (0.318)	104	-0.057 (95% CI -0.198, 0.085), p-value = 0.425
Day 7	52	0.788 (0.360)	54	0.762 (0.343)	106	0.037 (95% CI -0.102, 0.176), p-value = 0.596
Day 14	44	0.841 (0.338)	46	0.923 (0.157)	90	-0.086 (95% CI -0.200, 0.028), p-value = 0.135
Day 28	41	0.905 (0.246)	31	0.923 (0.169)	72	-0.014 (95% CI -0.124, 0.096), p-value = 0.798
EQ-5D VAS completed on behalf of child						
Day 0	120	60 (17)	129	58 (19)		
Day 4	77	72 (18)	76	68 (20)	153	4.1 (95% CI -1.5, 9.7), p-value = 0.146
Day 7	78	83 (18)	73	76 (22)	151	7.9 (95% CI 1.3, 14.4), p-value = 0.019
Day 14	61	87 (19)	57	90 (13)	118	-3.7 (95% CI -8.7, 1.3), p-value = 0.139
Day 28	53	89 (17)	40	86 (21)	93	4.1 (95% CI -5.0, 13.3), p-value = 0.368
**EQ-5D-Y completed by child**						
Day 0	32	0.469 (0.388)	29	0.531 (0.363)		
Day 4	16	0.626 (0.386)	13	0.717 (0.227)	29	-0.107 (95% CI -0.372, 0.157), p-value = 0.408
Day 7	17	0.815 (0.386)	11	0.805 (0.180)	28	-0.034 (95% CI -0.336, 0.269), p-value = 0.819
Day 14	17	0.957 (0.086)	10	0.918 (0.144)	27	0.028 (95% CI -0.073, 0.130), p-value = 0.565
Day 28	17	0.986 (0.039)	10	0.941 (0.124)	27	0.035 (95% CI -0.039, 0.109), p-value = 0.330
EQ-5D completed by child						
Day 0	28	54 (22)	25	55 (21)		
Day 4	19	62 (22)	13	59 (21)	32	4.2 (95% CI -11.2, 19.7), p-value = 0.577
Day 7	19	73 (21)	11	73 (25)	30	-0.7 (95% CI -24.9, 23.4), p-value = 0.952
Day 14	20	84 (19)	10	83 (26)	30	1.4 (95% CI -19.8, 22.6), p-value = 0.893
Day 28	18	88 (21)	11	84 (25)	29	3.0 (95% CI -9.2, 15.2), p-value = 0.612
**QALYs - rated on behalf of child**	29	0.061 (0.022)	25	0.065 (0.011)	54	-0.003 (95% CI -0.012, 0.007), p-value = 0.573

For peer review only - http://bmjopen.bmj.com/site/about/guidelines.xhtml

**QALYs - rated by child**	9	0.069 (0.004)	4	0.065 (0.007)	13	0.003 (95% CI -0.004, 0.009), p-value = 0.357
**CARIFS**						
**Day 0**	99	22 (10)	97	23 (11)		
**Day 7**	66	8 (8)	57	12 (11)	123	-3.5 (95% CI -6.9, -0.1), p-value = 0.044
**CARIFS VAS**						
Day 0	133	4.64 (1.90)	135	4.78 (1.92)		
Day 7	76	1.91 (1.97)	74	2.53 (2.45)	150	-0.63 (95% CI -1.38, 0.11), p-value = 0.096

**Differences have been adjusted for the stratification factors age (used as continuous variable) and seasonal influence vaccination status. Clustering by centre has been accounted for using the 'cluster' option in Stata's 'regress' command and robust standard errors were generated.*

EQ-5D-Y utilities: range -0.594 to 1, where 1 indicates perfect health

EQ-5D VAS: range 0 to 100, where higher values indicate better health.

QALYs (Quality Adjusted Life Years) were calculated only for children with data available at all relevant time points.

CARIFS (Canadian Acute Respiratory Infection and Flu Scale): range 0 to 54 points, with lower scores indicating fewer respiratory illness and flu symptoms.

CARIFS VAS: range 0 to 10, with higher scores indicating worse health states

CI – confidence interval; SD – standard deviation; VAS – visual analogue score

For peer review only - http://bmjopen.bmj.com/site/about/guidelines.xhtml

Table 4: Details of healthcare use for related re-consultations due to clinical deterioration and hospital admissions related to the illness episode for which the child was randomised into ARCHIE over the 28-day follow-up period

	Co-amoxiclav (N=133)	Placebo (N=132)	Total (N=265)
**RELATED RE-CONSULTATIONS**			
**Number of participants with at least one related re-consultation due to clinical deterioration**	33 (25%)	28 (21%)	61 (23%)
**Number of related re-consultations due to clinical deterioration**			
**0**	100 (75%)	104 (79%)	204 (77%)
**1**	30 (23%)	25 (19%)	55 (21%)
**2**	3 (2%)	3 (2%)	6 (2%)
**Number of participants with at least one related re-consultation due to clinical deterioration in a primary care or equivalent ambulatory care setting (excluding hospital admissions)***	28 (21%)	23 (17%)	51 (19%)
**Related re-consultation due to clinical deterioration in a primary care or equivalent ambulatory care setting (excluding hospital admissions)***			
**0**	105 (79%)	109 (83%)	214 (81%)
**1**	25 (19%)	21 (16%)	46 (17%)
**2**	3 (2%)	2 (2%)	5 (2%)
**Number of antibiotics received at related re-consultation***			
**0**	120 (90%)	119 (90%)	239 (90%)
**1**	13 (10%)	13 (10%)	26 (10%)
**Number of other drugs received at related re-consultation***			
**0**	126 (95%)	127 (96%)	253 (95%)
**1**	6 (5%)	1 (1%)	7 (3%)
**2**	1 (1%)	1 (1%)	2 (1%)
**3**	0 (0%)	3 (2%)	3 (1%)
**Number of chest x-rays at related re-consultation***			
**0**	129 (97%)	131 (99%)	260 (98%)
**1**	4 (3%)	1 (1%)	5 (2%)
**Number of other interventions at related re-consultation*, ****			
**0**	131 (98%)	132 (100%)	263 (99%)
**1**	2 (2%)	0 (0%)	2 (1%)
**RELATED HOSPITAL ADMISSIONS**			
**Number of hospital admissions**			
**0**	128 (96%)	125 (95%)	253 (95%)
**1**	5 (4%)	7 (5%)	12 (5%)

For peer review only - http://bmjopen.bmj.com/site/about/guidelines.xhtml

	Co-amoxiclav (N=133)	Placebo (N=132)	Total (N=265)
**Total nights in hospital**			
**0**	128 (96%)	125 (95%)	253 (95%)
**1**	3 (2%)	1 (1%)	4 (2%)
**2**	1 (1%)	4 (3%)	5 (2%)
**3**	0 (0%)	1 (1%)	1 (0%)
**7**	1 (1%)	1 (1%)	2 (1%)
**Number of antibiotics received during hospital admission**			
**0**	131 (98%)	126 (95%)	257 (97%)
**1**	2 (2%)	6 (5%)	8 (3%)
**Number of other drugs received during hospital admission**			
**0**	129 (97%)	126 (95%)	255 (96%)
**1**	1 (1%)	0 (0%)	1 (0%)
**2**	2 (2%)	2 (2%)	4 (2%)
**3**	1 (1%)	4 (3%)	5 (2%)
**Number of X-rays during hospital admission**			
**0**	132 (99%)	130 (98%)	262 (99%)
**1**	1 (1%)	2 (2%)	3 (1%)
**Number of blood tests during hospital admission**			
**0**	133 (100%)	130 (98%)	263 (99%)
**1**	0 (0%)	2 (2%)	2 (1%)
**Number of emergency department visits****			
**0**	129 (97%)	128 (97%)	257 (97%)
**1**	4 (3%)	4 (3%)	8 (3%)

Note: The summaries encompass the entire 28-day follow-up period, and may refer to more than one re-consultation episode.
**These summaries only include re-consultations that were not reported elsewhere in the form of hospital or emergency department admissions*
***Other interventions included blood and/or urine samples*
*** Participants who were reported to have been referred to the hospital team/ emergency department for acute admission during a re-consultation for clinical deterioration for the same illness episode for which the child was randomised, but for whom no hospital admission is recorded at this date, were classed as having visited emergency department. Hospital admission episodes for at least one night were collected during the notes review.*
The trial did not report any admissions to intensive care units (ICU).

For peer review only

Table 5: Overview of costs per participant for re-consultations and hospital admissions related to index influenza-like illness episode+

	Co-amoxiclav: Cost in £ (N = 133)				Placebo: Cost in £ (N = 132)				Difference* in cost in £ (95% CI) N = 265	
Items	Mean (S.D.)	Median	IQR	Range	Mean (S.D.)	Median	IQR	Range	Mean difference (95% CI)	p-value
Intervention	5 (1)	4	4, 6	4, 6	0 (0)	0	0, 0	0, 0	5 (95% CI 4, 5)	<0.001
Re-consultations	16 (46)	0	0, 0	0, 201	16 (54)	0	0, 0	0, 402	1 (95% CI -9, 10)	0.910
Antibiotics at re-consultation	0 (1)	0	0, 0	0, 10	0 (1)	0	0, 0	0, 5	0 (95% CI 0, 0)	0.920
Other drugs at re-consultation	0 (2)	0	0, 0	0, 13	1 (4)	0	0, 0	0, 34	0 (95% CI -1, 1)	0.593
X-rays at re-consultation	7 (40)	0	0, 0	0, 231	2 (20)	0	0, 0	0, 231	5 (95% CI -2, 13)	0.178
Other interventions at re-consultation	0 (0)	0	0, 0	0, 4	0 (0)	0	0, 0	0, 0	0 (95% CI 0, 0)	0.210
Hospital stay	59 (421)	0	0, 0	0, 4564	94 (484)	0	0, 0	0, 4564	-34 (95% CI -118, 51)	0.432
Antibiotics during hospital stay	0 (2)	0	0, 0	0, 21	0 (0)	0	0, 0	0, 3	0 (95% CI 0, 1)	0.247
Other drugs during hospital stay	0 (1)	0	0, 0	0, 13	1 (3)	0	0, 0	0, 34	0 (95% CI -1, 0)	0.283
X-rays in hospital	2 (20)	0	0, 0	0, 231	4 (28)	0	0, 0	0, 231	-2 (95% CI -8, 5)	0.596
Other interventions in hospital	0 (0)	0	0, 0	0, 0	0 (0)	0	0, 0	0, 1	0 (95% CI 0, 0)	0.168
Emergency department	5 (1)	0	0, 0	0, 160	5 (28)	0	0, 0	0, 160	0 (95% CI -6, 6)	0.983
**Total cost**	94 (480)	4	4, 6	4, 5258	122 (539)	0	0, 0	0, 5177	-25 (95% CI -113, 62)	0.566
Total primary care costs	8 (29)	0	0, 0	0, 300	4 (12)	0	0, 0	0, 63	3 (95% CI -2, 8)	0.215
Total secondary care costs	82 (481)	0	0, 0	0, 5254	117 (540)	0	0, 0	0, 5177	-33 (95% CI -120, 54)	0.453

**Differences have been adjusted for the minimisation factors age (used as continuous variable) and seasonal influenza vaccination status. Clustering by centre has been accounted for using the 'cluster' option in Stata's 'regress' command and robust standard errors were generated.*

CI – confidence interval; IQR - Interquartile range; SD – standard deviation

+related to the illness episode for which the child was randomised into ARCHIE over the 28-day follow-up period

For peer review only - http://bmjopen.bmj.com/site/about/guidelines.xhtml

Note: re-consultations and related costs were attributed to primary care costs for children who had been recruited in a GP setting, and to secondary care costs for children who had been recruited in a paediatric ambulatory assessment unit. The primary and secondary care costs shown relate to the trial follow-up period, and do not include the trial intervention.

For peer review only

For peer review only - http://bmjopen.bmj.com/site/about/guidelines.xhtml

Subgroup analysis

Figure 1 shows the difference in costs between the treatment arms by whether or not influenza was confirmed through nasal swabs taken at baseline. Two hundred and sixty-two children for whom the results of nasal swabs were available are included in these summaries. Of these, 21 participants in the co-amoxiclav group (15%), and 16 participants in the placebo group (12%) had laboratory-confirmed influenza. There was a trend towards lower costs in the co-amoxiclav arm for children with laboratory-confirmed influenza, though these differences were not statistically significant.

Extrapolation of findings to national scale

According to the Office for National Statistics, in mid-2018[21], there were approximately 9.7 million children aged 6 months to 12 years in the UK. Information from the 'Surveillance of influenza and other respiratory viruses in the UK' indicates that maximum weekly rates of GP consultations for ILI in 2018/2019 were around 21 per 100,000 for children aged <1 years, 25 per 100,000 for children aged 1-4 years and 19 per 100,000 for children aged 5 to 14[22].

We assume approximately 21 per 100,000 GP consultations were for influenza-like symptoms over an 8-week period in children. Weekly GP consultations for the remaining 44 weeks of the year were assumed to be 5 per 100,000.

Finally, Lee et al[9] estimate that 15.3% of children with influenza like symptoms suffer from at least one of the underlying conditions that were used to classify children as 'at risk', and hence eligible for the ARCHIE trial.

This means that UK-wide, there were around 37,821 GP consultations for influenza like symptoms, 15.3% (5,787) of which would have been expected to occur in the 'at risk' groups included in the ARCHIE study. Using the estimated cost difference between the treatment arms of -£25 (95% CI -113, 62), the potential cost saving of early co-amoxiclav treatment

For peer review only - http://bmjopen.bmj.com/site/about/guidelines.xhtml

compared with not providing early antibiotics is estimated as £-144,675 (95% CI £-358,794 to £653,931).

Discussion

In this work, we have summarised the health-related quality of life, healthcare use and costs associated with treating ‘at risk’ children with ILI with co-amoxiclav versus placebo.

Our findings, which present within-trial comparisons of costs and outcomes separately, are an important addition to the literature, due to the paucity of observational data and no previous clinical trials performed in this cohort of ‘at risk’ children. Recruitment from both primary and other ambulatory care settings with wide geographical coverage, a very pragmatic definition of ILI ensured that our study population was highly representative of ‘at risk’ children with ILI, High data availability rates for NHS costs as obtained via the notes review are also a strength of this study Due to the low completion rate of the EQ-5D-Y, we did not perform a full cost-effectiveness analysis. Data on school/ nursery attendance and childcare arrangements were also subject to low completion rates, and prevented a more robust assessment form a societal perspective.

The low response rates for the EQ-5D-Y and CARIFS data demonstrates the difficulties in assessing health-related quality of life in this patient population. Low questionnaire return rates are a common limitation of paediatric trials; for example, return rates at 14 days from two trials in respiratory tract infections were reported as 41%[23]. In our study, there was anecdotal evidence of parents and carers struggling to complete the questionnaires, especially the EQ-5D-Y questions on walking, usual activities and dressing, if the items were not appropriate to the expected capabilities of their children, either because of their age, especially for very young children, or their underlying condition or disability. This was reflected in the lower proxy completion rates of the EQ-5D-Y for children below the age of two years, compared to those

For peer review only - http://bmjopen.bmj.com/site/about/guidelines.xhtml

aged 2 years and over, and the fact that the VAS scales of the EQ-5D-Y and the CARIFS had higher completion rates than the Likert-style questions of the questionnaires, as shown in the Supplemental Material. Generally, there is a need for validated questionnaires that can be completed easily in this patient population.

The EQ-5D-Y was expected to be completed by proxy, i.e. by the children's parents or carers on their behalf, and/or by the children themselves, as appropriate. There appeared to be some overlap between the answers provided on the proxy and self-completed EQ-5D-Y questionnaires, particularly for very young children. It is possible that some parents or carers completed the questionnaires supposed to be filled in by the children. All EQ-5D-Y data were analysed as received, without exclusions.

The proxy-completed EQ-5D-Y values were not statistically significantly different between the two groups at any of the time points, although there was an improvement in health-related quality of life (HRQOL) as indicated by the EQ-5D-Y scores over time (day 0 to day 28) for both trial arms. There was a trend towards lower EQ-5D-Y values, indicating worse quality of life, in the co-amoxiclav arm on days 4, 14 and 28. The CARIFS score in the co-amoxiclav arm at seven days was significantly lower than the score in the placebo arm, indicating less severe symptoms. This might suggest that the CARIFS score is more sensitive to subtle disease specific changes in children's health than the EQ-5D-Y. Interestingly, the EQ-5D VAS also showed a statistically significant benefit of co-amoxiclav at seven days, and the EQ-5D-Y values were higher (not statistically significant) in the co-amoxiclav arm at seven days compared with the placebo arm, indicating higher HRQOL. Studies of common cold or non-specific respiratory tract infections in general paediatric populations suggest that symptoms in 50% of children had improved by day seven to eight, and for 80% by day 14[24]. It may therefore be that any differences between groups will be more apparent at a stage in the illness where more children are likely to still be symptomatic than later on, when the majority will have resolved due to the natural course of the illness. Both questionnaires are subject to high levels of missing data, and should be interpreted cautiously. The high rates of missing data and

For peer review only - http://bmjopen.bmj.com/site/about/guidelines.xhtml

anecdotal evidence of parents' and carers' difficulties of completing the questionnaires demonstrate a need for additional validated tools to reliably measure quality of life in this patient population. The EQ-5D-Y, EQ-5D-VAS and CARIFS were secondary endpoints of the ARCHIE trial, and the EQ-5D was measured at several follow-up time points. As such, the limited number of statistically significant p-values might be false positives due to multiple testing and should be interpreted carefully.

Overall, only 5% of children were admitted to hospital for reasons related to the illness episode for which the child was randomised into ARCHIE over the 28-day follow-up period. Admissions were often only for one or two nights. Three percent of participants reported one emergency department visit each. Fewer than 25% of all randomised children had a re-consultation. This was lower than the anticipated 40% re-consultation rate based on published data available at the time the study was designed[2], but still significantly higher than the 4% observed in a primary care cohort of children with acute respiratory tract infection who did not have known risk factors for complications from influenza or ILI[25]. As such, the use of primary and secondary care facilities during the trial follow-up for reasons related to the initial illness episode for which the child was randomised into ARCHIE was generally low and the limited related hospital admissions were the main cost drivers for the average cost per randomised child. In the co-amoxiclav arm, costs related to hospital admissions and emergency department visits accounted for 70% of total costs; in the placebo arm, this was 85%.

Fourteen per cent of the study population had laboratory-confirmed influenza. We conducted a pre-specified subgroup analysis in participants with laboratory-confirmed influenza because previous data suggesting a possible clinical benefit from early antibiotic use are reported in trials which recruited patients with confirmed influenza[26] or ILI presenting during an influenza epidemic[27], when a high proportion of ILI cases are likely to be due to influenza. This subgroup analysis indicated that there was a trend that the early co-amoxiclav treatment may be more cost-saving in participants with laboratory-confirmed influenza, although this subgroup does

For peer review only - http://bmjopen.bmj.com/site/about/guidelines.xhtml

not have sufficient statistical power to draw definitive conclusions. Also, this trial assessed the benefit of treating at risk children as early as possible after the onset of ILI. While there is a possibility that treatment may be more beneficial in those with laboratory-confirmed influenza, evaluating the nasal swabs takes time, and may make it impossible to treat these children early. Nasal swabs were costed at £8 per child, and all children presenting with ILI would need to be tested. This outweighs the costs of co-amoxiclav, which was costed at £4.13 for children below 7 year of age, and at £5.79 children above 7 years of age. As such, there is no advantage in terms of costs to confirm influenza prior to commencing of treatment outside influenza pandemic periods. Based on the findings of the subgroup analysis, early co-amoxiclav treatment without prior nasal swab testing may be cost-effective during periods of high influenza activity, such as influenza pandemics. However, further trials conducted during such periods would be needed to confirm this.

In line with the study protocol, we aimed to extrapolate the results of the study to a national level. With regards to the ARCHIE trial, this extrapolation has a number of limitations. Firstly, the difference in costs between the co-amoxiclav and placebo was not statistically significant. Therefore, it is unclear if extrapolation to a national level would result in a cost-saving or increase in spending. Finally, influenza is an infectious disease, and rates of infections vary greatly between epidemics. Our current extrapolation uses GP consultations for ILI in 2018/2019. However, it is unclear how many children may be affected in the upcoming flu seasons. This is particularly important as the rates of influenza were low during the recruitment period of ARCHIE[28-31], but more children may be affected in coming years. Our extrapolations were based on recent data, but may have to be revised in the light of SARS-CoV-2, and its impact on influenza and ILI in future influenza seasons.

Neither the findings of this economic evaluation nor the findings of our main trial[1] support early use of co-amoxiclav in 'at risk' children who present with ILI in primary or ambulatory care[1]. With increasing concerns about growing antimicrobial resistance[32,33], preserving antibiotic effectiveness by avoiding routine early co-amoxiclav in this group is therefore important. The

For peer review only - http://bmjopen.bmj.com/site/about/guidelines.xhtml

findings of a longitudinal study nested within the ARCHIE trial will report data on long-term bacterial carriage and antibiotic resistance in the co-amoxiclav versus placebo arms in a separate paper. These findings may help inform estimations relating to the potential implications of antibiotic resistance on the results reported in this economic evaluation. However, incorporating the potential cost of antimicrobial resistance to the health system and society into economic evaluations is challenging[32,34,35].

Conclusions

Our findings did not show evidence that early treatment with co-amoxiclav in 'at risk' children with influenza-like illness improves quality of life, reduces healthcare use or leads to cost savings over 28 days. However, there was a suggestion that early co-amoxiclav treatment might be associated with decreased symptom severity at day 7, though confirmation from additional research would be important. Collection of proxy-reported data was challenging in this patient population. Suitable validated quality of life instruments are therefore needed to support more robust evaluations in this group.

List of abbreviations

CARIFS: Canadian Acute Respiratory Infection and Flu Scale

CI: Confidence Interval

HRQL: Health-Related Quality of Life

ICU: Intensive Care Unit

ILI: Influenza-Like Illness

IQR: Interquartile Range

QALYs: Quality Adjusted Life Years

SD: Standard Deviation

VAS: Visual Analogue Scale

For peer review only - http://bmjopen.bmj.com/site/about/guidelines.xhtml

Acknowledgements

The research team acknowledges the support of the National Institute for Health Research Clinical Research Network (NIHR CRN). We would like to thank the ARCHIE Investigators for their support throughout the study, in particular: Malcolm G Semple PhD, Michael Moore FRCGP, Alastair D Hay FRCGP, Ushma Galal MSc, Tricia Taffe Carver BS, Rafael Perera-Salazar DPhil, Ly-Mee Yu DPhil, Jill Mollison PhD, Paul Little FRCGP, Andrew Farmer DM and Christopher Butler FMedSci.

We thank Mandy Wan (Paediatric Research Pharmacist, NIHR CRN Children speciality) for her support and guidance regarding trial medication, from procurement to disposal. Staff in Oxford University's Primary Care Clinical Trials Unit were responsible for randomisation software design (David Judge), database development (Sadie Kelly), trial co-ordination (Sonya Beecher) and patient recruitment (Heather Rutter, Karen Madronal, Irene Noel, Luisa Saldana Ortega, Pippa Whitbread, Bernadette Mundy, Belinda I'Anson, Samantha Spires). We thank our Programme Steering Committee (Professor Willie Hamilton, Professor Judy Breuer, Professor Maureen Baker, Robert Newby), and Data and Safety Monitoring Committee (Professor Lorcan McGarvey, Mike Bradburn, Professor Surinder Birring, Professor Nick Francis), and our regional trial coordinators (Lyn Liddiard, Tammy Thomas, Clare McIntyre) for their valuable advice and support with delivering the trial. We thank Mildred Foster and Dan Richards-Doran for developing our study website and other study-related publicity materials. We also thank Dr Oliver van Hecke for his assistance with monitoring data re-consultation data extracted from medical records. Last but not least, we thank all the health care professionals, general practice and hospital staff, children and parents/guardians who participated in our study.

Funding statement

For peer review only

This project was funded by the National Institute for Health Research (NIHR) Programme Grants for Applied Research Programme (project reference RP-PG-1210-12012). The views expressed are those of the authors and not necessarily those of the NIHR or the Department of Health and Social Care. The funder of the study had no role in design and conduct of the study; collection, management, analysis, and interpretation of the data; preparation, review, or approval of the manuscript; and decision to submit the manuscript for publication.

Competing interests

KW held a National Institute for Health Research (NIHR) Academic Clinical Lectureship during the conduct of the study and currently holds a NIHR Postdoctoral Fellowship.

The other authors declare that they have no competing interests.

Author contributions

Kay Wang was Chief Investigator of the trial until going on maternity leave in September 2018. She reviewed clinical data on outcome events to guide collation and analysis of data for this study.

Sharon Tonner was Trial Manager of the trial from 2017 until its conclusion. She managed the day to day data collection activities.

Jenna Grabey was the data manager on the trial from January 2018 until its conclusion.

Anthony Harnden was Chief Investigator on the trial from September 2018 until its conclusion. He reviewed clinical data on outcome events to guide collation and analysis of data for this study.

Jane Wolstenholme was the senior health economist on the trial. She developed the economic evaluation plan, and led the economic analysis of the trial findings.

For peer review only - http://bmjopen.bmj.com/site/about/guidelines.xhtml

Ines Rombach conducted the analyses for this paper.

Ines Rombach and Jane Wolstenholme drafted the first version of the manuscript.

All authors reviewed the manuscript, contributed comments and edits, and approved the final version.

Collaborators

ARCHIE (The early use of antibiotics for At-Risk CHildren with InfluEnza in primary care) investigators: Professor Anthony Harnden (Nuffield Department of Primary Care Health Sciences, University of Oxford, Oxford, UK), Dr Kay Wang (Nuffield Department of Primary Care Health Sciences, University of Oxford, Oxford, UK), Professor Malcolm G Semple (National Institute for Health Research (NIHR) Health Protection Research Unit in Emerging and Zoonotic Infections, University of Liverpool, Liverpool, UK), Dr Jane Wolstenholme (Health Economics Research Centre, University of Oxford, Oxford, UK), Professor Rafael Perera-Salazar (Nuffield Department of Primary Care Health Sciences, University of Oxford, Oxford, UK), Dr Ly-Mee Yu (Nuffield Department of Primary Care Health Sciences, University of Oxford, Oxford, UK), Professor Alastair D Hay (Centre for Academic Primary Care, School of Social and Community Medicine, University of Bristol, Bristol, UK), Professor Paul Little (Primary Care Population Sciences and Medical Education, University of Southampton, Southampton, UK), Professor Michael Moore (Primary Care Population Sciences and Medical Education, University of Southampton, Southampton, UK), Professor Chris Butler (Nuffield Department of Primary Care Health Sciences, University of Oxford, Oxford, UK), Tricia Taffe Carver (Nuffield Department of Primary Care Health Sciences, University of Oxford, Oxford, UK).

Ethical approval

For peer review only - http://bmjopen.bmj.com/site/about/guidelines.xhtml

The ARCHIE trial has been reviewed and approved by the North West-Liverpool East Research Ethics Committee (13/NW/0621), Health Research Authority and Medicines and Healthcare Products Regulatory Agency. Full written informed consent was obtained from a parent or guardian for each study participant. Children were also invited to give written assent if appropriate.

Data sharing:

The trial protocol and de-identified participant level data collected for the trial are available on request. Research data requests should be submitted to the corresponding author for consideration by the research team.

For peer review only

List of Figures

Figure 1: Forest plot for differences in costs by laboratory-confirmed influenza (in £)

**Subgroup effects were obtained by adding an interaction term between treatment allocation and laboratory-confirmed influenza and have been adjusted for the stratification factors age (used as continuous variable) and seasonal influenza vaccination status. Clustering by centre has been accounted for using the 'cluster' option in Stata's 'regress' command and robust standard errors were generated.*

For peer review only

References

1. Wang K, Semple MG, Moore M, et al. The early use of Antibiotics for at Risk CHildren with InfluEnza-like illness (ARCHIE): a double-blind randomised placebo-controlled trial. *Eur Respir J* 2021.
2. Wang K, Carver T, Tonner S, et al. Early use of Antibiotics for at Risk CHildren with InfluEnza (ARCHIE): protocol for a double-blind, randomised, placebo-controlled trial. *BMJ Open* 2018; **8**(5): e021144.
3. Burch J, Paulden M, Conti S, et al. Antiviral drugs for the treatment of influenza: a systematic review and economic evaluation. *Health Technol Assess* 2009; **13**(58): 1-265, iii-iv.
4. Tappenden P, Jackson R, Cooper K, et al. Amantadine, oseltamivir and zanamivir for the prophylaxis of influenza (including a review of existing guidance no. 67): a systematic review and economic evaluation. *Health Technol Assess* 2009; **13**(11): iii, ix-xii, 1-246.
5. Turner D, Wailoo A, Nicholson K, Cooper N, Sutton A, Abrams K. Systematic review and economic decision modelling for the prevention and treatment of influenza A and B. *Health Technol Assess* 2003; **7**(35): iii-iv, xi-xiii, 1-170.
6. Nicholson KG, Aoki FY, Osterhaus AD, et al. Efficacy and safety of oseltamivir in treatment of acute influenza: a randomised controlled trial. Neuraminidase Inhibitor Flu Treatment Investigator Group. *Lancet* 2000; **355**(9218): 1845-50.
7. Treanor JJ, Hayden FG, Vrooman PS, et al. Efficacy and safety of the oral neuraminidase inhibitor oseltamivir in treating acute influenza: a randomized controlled trial. US Oral Neuraminidase Study Group. *JAMA* 2000; **283**(8): 1016-24.
8. Williamson I, Benge S, Barton S, et al. A double-blind randomised placebo-controlled trial of topical intranasal corticosteroids in 4- to 11-year-old children with persistent bilateral otitis media with effusion in primary care. *Health Technol Assess* 2009; **13**(37): 1-144.
9. Lee JJ, Bankhead C, Smith M, Kousoulis AA, Butler CC, Wang K. Risk factors for influenza-related complications in children during the 2009/10 pandemic: a UK primary care cohort study using linked routinely collected data. *Epidemiol Infect* 2018; **146**(7): 817-23.
10. Blackburn RM, Henderson KL, Lillie M, et al. Empirical treatment of influenza-associated pneumonia in primary care: a descriptive study of the antimicrobial susceptibility of lower respiratory tract bacteria (England, Wales and Northern Ireland, January 2007-March 2010). *Thorax* 2011; **66**(5): 389-95.
11. Wolstenholme JL, Bargo D, Wang K, Harnden A, Raisanen U, Abel L. Preference-based measures to obtain health state utility values for use in economic evaluations with child-based populations: a review and UK-based focus group assessment of patient and parent choices. *Qual Life Res* 2018; **27**(7): 1769-80.
12. Kreimeier S, Oppe M, Ramos-Goni JM, et al. Valuation of EuroQol Five-Dimensional Questionnaire, Youth Version (EQ-5D-Y) and EuroQol Five-Dimensional Questionnaire, Three-Level Version (EQ-5D-3L) Health States: The Impact of Wording and Perspective. *Value Health* 2018; **21**(11): 1291-8.
13. Brooks R. EuroQol: the current state of play. *Health Policy* 1996; **37**(1): 53-72.
14. EuroQol G. EuroQol--a new facility for the measurement of health-related quality of life. *Health Policy* 1990; **16**(3): 199-208.
15. Rabin R, de Charro F. EQ-5D: a measure of health status from the EuroQol Group. *Ann Med* 2001; **33**(5): 337-43.
16. Jacobs B, Young NL, Dick PT, et al. Canadian Acute Respiratory Illness and Flu Scale (CARIFS): development of a valid measure for childhood respiratory infections. *J Clin Epidemiol* 2000; **53**(8): 793-9.
17. Personal Social Services Research Unit (PSSRU). Unit Costs of Health and Social Care 2018. 23/05/2019). https://www.pssru.ac.uk/project-pages/unit-costs/unit-costs-2018/.
18. NHS Improvement. Reference Cost Collection: National Schedule of Reference Costs - Year 2017-18. https://improvement.nhs.uk/resources/reference-costs/#rc1718 (accessed 23/05 2019).

For peer review only - http://bmjopen.bmj.com/site/about/guidelines.xhtml

19. Fenwick EA, Briggs AH, Hawke CI. Management of urinary tract infection in general practice: a cost-effectiveness analysis. *Br J Gen Pract* 2000; **50**(457): 635-9.
20. Paediatric Formulary Committee. BNF for Children (online) London: BMJ Group, Pharmaceutical Press, and RCPCH Publications. https://www.medicinescomplete.com/ (accessed 23 November 2019).
21. Office for National Statistics. Dataset: Estimates of the population for the UK, England and Wales, Scotland and Northern Ireland. 2019. https://www.ons.gov.uk/peoplepopulationandcommunity/populationandmigration/populationestimates/datasets/populationestimatesforukenglandandwalesscotlandandnorthernireland (accessed 05/12/ 2019).
22. Public Health England. Surveillance of influenza and other respiratory viruses in the UK: Winter 2018 to 2019. *Influenza Surveillance Team, Immunisation and Countermeasures Division, National Infection Service, PHE,* 2019.
23. Schot MJC, Dekker ARJ, van Werkhoven CH, et al. Burden of disease in children with respiratory tract infections in primary care: diary-based cohort study. *Fam Pract* 2019; **36**(6): 723-9.
24. Thompson M, Vodicka TA, Blair PS, et al. Duration of symptoms of respiratory tract infections in children: systematic review. *BMJ* 2013; **347**: f7027.
25. Redmond NM, Turnbull S, Stuart B, et al. Impact of antibiotics for children presenting to general practice with cough on adverse outcomes: secondary analysis from a multicentre prospective cohort study. *Br J Gen Pract* 2018; **68**(675): e682-e93.
26. Kakeya H, Seki M, Izumikawa K, et al. Efficacy of combination therapy with oseltamivir phosphate and azithromycin for influenza: a multicenter, open-label, randomized study. *PLoS One* 2014; **9**(3): e91293.
27. Maeda S, Yamada Y, Nakamura H, Maeda T. Efficacy of antibiotics against influenza-like illness in an influenza epidemic. *Pediatr Int* 1999; **41**(3): 274-6.
28. Public Health England. Surveillance of influenza and other respiratory viruses in the United Kingdom: winter 2014 to 2015. https://assets.publishing.service.gov.uk/government/uploads/system/uploads/attachment_data/file/429617/Annualreport_March2015_ver4.pdf (accessed 26th May 2020).
29. Public Health England. Surveillance of influenza and other respiratory viruses in the United Kingdom: Winter 2015 to 2016. https://assets.publishing.service.gov.uk/government/uploads/system/uploads/attachment_data/file/526405/Flu_Annual_Report_2015_2016.pdf (accessed 26th May 2020).
30. Public Health England. Surveillance of influenza and other respiratory viruses in the UK: Winter 2016 to 2017. https://assets.publishing.service.gov.uk/government/uploads/system/uploads/attachment_data/file/613493/Surveillance_of_influenza_and_other_respiratory_viruses_in_the_UK_2016_to_2017.pdf (accessed 26th May 2020).
31. Public Health England. Surveillance of influenza and other respiratory viruses in the UK: Winter 2017 to 2018. https://assets.publishing.service.gov.uk/government/uploads/system/uploads/attachment_data/file/740606/Surveillance_of_influenza_and_other_respiratory_viruses_in_the_UK_2017_to_2018.pdf (accessed 26th May 2020).
32. Coast J, Smith R, Karcher AM, Wilton P, Millar M. Superbugs II: how should economic evaluation be conducted for interventions which aim to contain antimicrobial resistance? *Health Econ* 2002; **11**(7): 637-47.
33. Roope LSJ, Smith RD, Pouwels KB, et al. The challenge of antimicrobial resistance: What economics can contribute. *Science* 2019; **364**(6435).
34. Coast J, Smith RD, Millar MR. Superbugs: should antimicrobial resistance be included as a cost in economic evaluation? *Health Econ* 1996; **5**(3): 217-26.

For peer review only - http://bmjopen.bmj.com/site/about/guidelines.xhtml

35. Jit M, Ng DHL, Luangasanatip N, et al. Quantifying the economic cost of antibiotic resistance and the impact of related interventions: rapid methodological review, conceptual framework and recommendations for future studies. *BMC Med* 2020; **18**(1): 38.

For peer review only

For peer review only - http://bmjopen.bmj.com/site/about/guidelines.xhtml

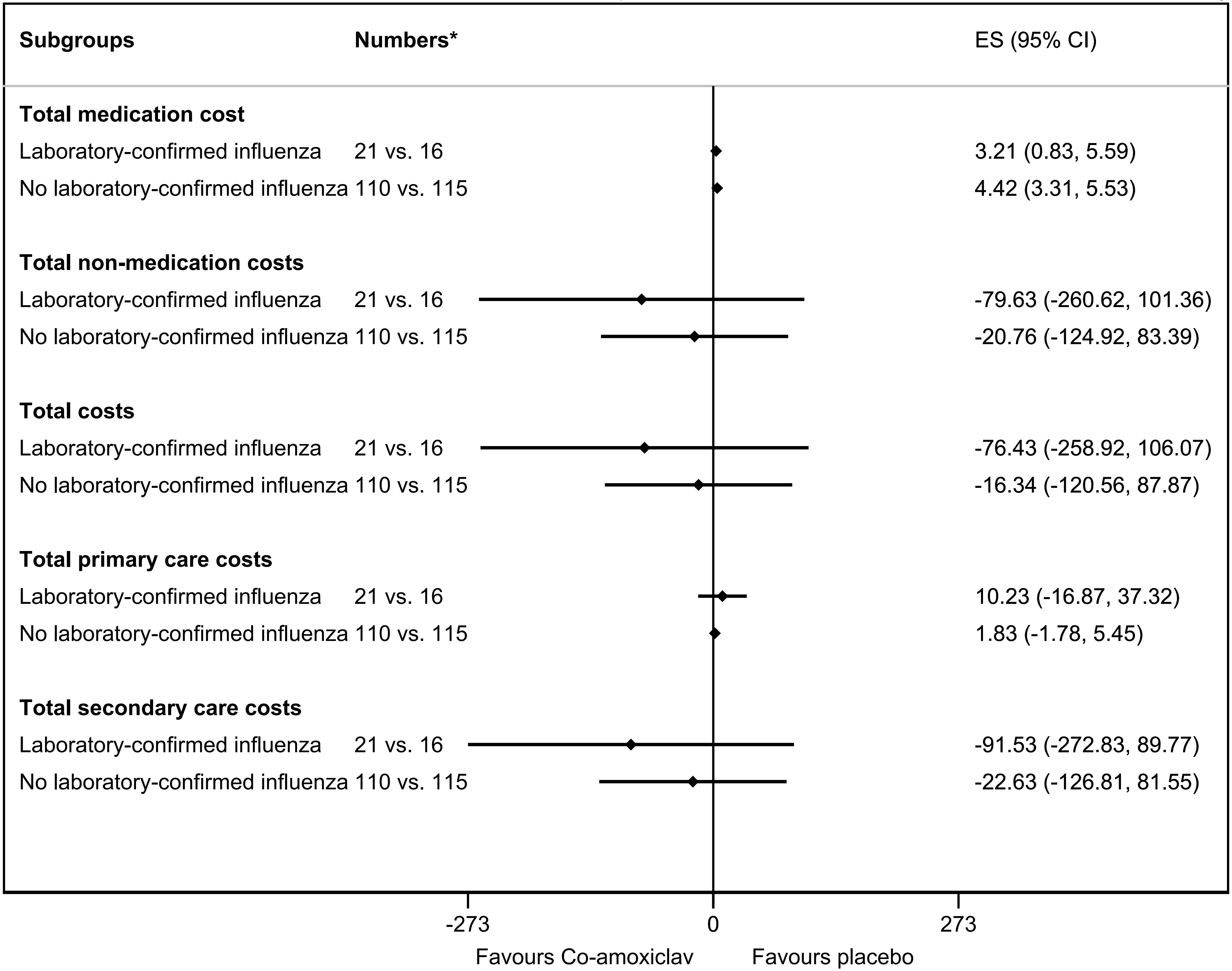

* n in active vs. n in placebo arm
ES - effect size

Supplemental material for:

Quality of life, healthcare use and costs in 'at risk' children after early antibiotic treatment vs. placebo for influenza-like illness: Within-trial descriptive economic analyses of the ARCHIE randomised controlled trial

Authors

Ines Rombach (ines.rombach@ndorms.ox.ac.uk)[1],

Kay Wang (kay.wang@phc.ox.ac.uk)[2],

Sharon Tonner (sharon.tonner@phc.ox.ac.uk)[2],

Jenna Grabey (jenna.grabey@phc.ox.ac.uk)[2],

Anthony Harnden (anthony.harnden@phc.ox.ac.uk)[2],

Jane Wolstenholme (jane.wolstenholme@ndph.ox.ac.uk)[1],

ARCHIE Investigators

Affilitations

[1]Health Economics Research Centre, Nuffield Department of Population Health, Oxford University, Oxford, UK

[2]Nuffield Department of Primary Care Health Sciences, University of Oxford, Oxford, UK

Corresponding author:
Ines Rombach
Health Economics Research Centre
Nuffield Department of Population Health
Old Road Campus
University of Oxford
Oxford
OX3 7LF
United Kingdom
ines.rombach@ndorms.ox.ac.uk
https://orcid.org/0000-0003-3464-3867

Published in BMJ Open

For peer review only - http://bmjopen.bmj.com/site/about/guidelines.xhtml

Table of Contents

Supplemental File 1: Unit costs ... 3
Supplemental Table S1: Unit costs ... 3
Supplemental File 2: Availability of data reported on behalf of the children, and by the children ... 6
Supplemental Table S2a: Available EQ-5D-Y data for participants aged less than 2 years ... 6
Supplemental Table S2b: Available EQ-5D-Y data for participants aged 2+ years ... 8
Supplemental Table S2c: Missing data pattern over time for the EQ-5D-Y proxy, completed on behalf of the child ... 9
Supplemental Table S2d: Proxy-reported EQ-5D-Y scores by data availability at the subsequent follow-up ... 9
Supplemental Table S2e: Missing data pattern for the EQ-5D-Y domains (proxy data, completed on behalf of the child) ... 10
Supplemental Table S2f: Available EQ-5D-Y items ... 11
Supplemental Table S2g: Available EQ-5D-Y items for children aged less than 2 years ... 12
Supplemental Table S2h: Available EQ-5D-Y items for children aged 2+ years (proxy) ... 13
Supplemental File 3: Reponses to the EQ-5D-Y items ... 14
Supplemental Table S3a: Responses to EQ-5D-Y domains (proxy, completed on behalf of child) 15
Supplemental Table S3b: Responses to EQ-5D-Y domains (proxy, completed on behalf of child for children aged less than 2 years) ... 16
Supplemental Table S3c: Responses to EQ-5D-Y domains (proxy, completed on behalf of child for children aged 2+ years) ... 17
Supplemental Table S3d: Responses to EQ-5D-Y domains (completed by child) ... 18
Supplemental Table S3e: Responses to EQ-5D-Y domains (completed by child for children aged less than 2 years) ... 19
Supplemental Table S3f: Responses to EQ-5D-Y domains (completed by child for children aged 2+ years) ... 20
Supplemental Table 3g: Responses to CARIFS domains (only including questionnaires with sufficient data to calculate the overall CARIFS score) ... 21
Supplemental Table 3h: Responses to CARIFS domains for children less than 2 years old (only including questionnaires with sufficient data to calculate the overall CARIFS score) ... 24
Supplemental Table 3i: Responses to CARIFS domains for children aged 2+ years (only including questionnaires with sufficient data to calculate the overall CARIFS score) ... 26
Supplemental File 4: Additional summaries of research use ... 29
Supplemental Table 4a: Details of all reported hospital admissions ... 29
Supplemental Table 4b: Overview of healthcare use for re-consultations due to clinical deterioration and hospitalisations related to the illness episode for which the child was randomised to ARCHIE 30
Supplemental File 5: Number of days children were unable to attend school or nursery, and subsequent changes to childcare requirements ... 31
Supplemental Table 5: Daily activity and child care ... 31

For peer review only - http://bmjopen.bmj.com/site/about/guidelines.xhtml

Supplemental File 1: Unit costs

Supplemental Table S1: Unit costs

Cost item	Cost	Data Source
Re-consultations primary care (GP)	£31	Personal Social Services Research Unit (PSSRU), online unit cost database of health and social care professionals 2017/2018: GP including direct care, cost per surgery lasting 9.22 minutes.
Re-consultation in paediatric ambulatory assessment units	£201	Weighted average of consultant-led and non-consultant led non-admitted face-to-face follow-up attendance for paediatric respiratory medicine; National Schedule of reference costs 2017/2018, CL and NCL tab.
Chest x-ray	£231	Outpatient procedures, paediatric same-day diagnostic imaging; National Schedule of reference costs 2017/2018, OPROC tab. (Note: the same cost was applied to chest x-rays at re-consultation and during hospital admission, as it was assumed that x-rays for the ARCHIE participants would have to be performed on the same day)
Night in hospital	£652	Weighted average of cost per day for non-elective paediatric hospital stays; National Schedule of reference costs 2017/2018, NEL, NEL_XS and NES tab.
Emergency department	£160	Weighted average of all emergency department attendances excluding dental care and patient dead on arrival, National Schedule of reference costs 2017/2018, AE tab.
**Day case**	£610	
**Investigations performed**		
Blood test	£1	Cost of clinical biochemistry, National Schedule of reference costs 2017/2018, DAPS tab.
Nasal aspirate/ npa	£8	Cost of microbiological diagnostic test, National Schedule of reference costs 2017/2018, DAPS tab.
Throat swab	£8	Cost of microbiological diagnostic test, National Schedule of reference costs 2017/2018, DAPS tab.
Urine test	£2.80	Dipstick £0.20 + Laboratory culture £2.60, Fenwick et al[10]

For peer review only - http://bmjopen.bmj.com/site/about/guidelines.xhtml

**Antibiotics and other drugs prescribed**		Costed in line with British National Formulary for Children – accessed 22Aug2019.<br>The minimum Drug Tariff is used for all costs.
**Antibiotics**		
Amoxicillin	£1.26	Assume all amoxicillin given as oral suspension.<br>Amoxicillin (as Amoxicillin trihydrate) 50 mg per 1 ml – 100 ml cost £1.26
Azithromycin	£4.06	Assume all AZITHROMYCIN given as oral suspension.<br>Azithromycin 40 mg per 1 ml – 15 ml cost £4.06
Ceftriaxone	£9.58	By IV. One vial of Ceftriaxone (as Ceftriaxone sodium) 1g. 1 vial £9.58
Chloramphenicol	£1.55	Assume used as eye drops. Cost by vial.<br>Chloramphenicol 5 mg per 1 ml. 10 ml cost £1.55
Clarithromycin	£3.36	Assume oral suspension used, as tablet doses too high.<br>• Clarithromycin 25 mg per 1 ml. 70 ml cost £3.36
**Study intervention: Co-amoxiclav 400/57**	£4.13 children below 7<br>£5.79 children 7+	Amoxicillin (as Amoxicillin trihydrate) 80 mg per 1 ml, Clavulanic acid (as Potassium clavulanate) 11.4 mg per 1 ml<br>35 ml: £4.13, 70 ml: £5.79<br>Note: in the trial, co-amoxiclav 400/57 was provided by Brown & Burk UK Ltd. This was the only company who was able to procure a placebo suitable for use in this study. Recommendations from the study will not be restricted to this manufacturer. Therefore, the costing will also include other manufacturers, whose products are available in 35ml and 70 ml.
Co-amoxiclav	£1.65	Assume oral suspension.<br>Amoxicillin (as Amoxicillin trihydrate) 25 mg per 1 ml, Clavulanic acid (as Potassium clavulanate) 6.25 mg per 1 ml. (lower dose than study drug)<br>➔100ml cost £1.65
Dexamethasone	£21.15	Dexamethasone (as Dexamethasone sodium phosphate) 400 microgram per 1 ml. 150 ml cost £42.30.
Erythromycin ethyl succinate	£5.21	Assume oral suspension.<br>Erythromycin (as Erythromycin ethyl succinate) 25 mg per 1 ml. 100ml cost £5.21
Phenoxymethylpenicillin	£4.28	Assume oral solution.<br>Phenoxymethylpenicillin (as Phenoxymethylpenicillin potassium) 25 mg per 1 ml. 100 ml costs £4.28
**Other drugs**		

For peer review only - http://bmjopen.bmj.com/site/about/guidelines.xhtml

Atrovent	£5.56	Ipratropium bromide 20 microgram per 1 dose. Inhaler with 200 doses costs £5.56
Beclomethasone /unidentified steroids	£3.70	Beclometasone dipropionate 50 microgram per 1 dose. 200 dose inhaler costs £3.70.<br>(this was also used for unidentified steroids)
Dexamethasone	£42.30	Assume oral solution.<br>Dexamethasone (as Dexamethasone sodium phosphate) 400 microgram per 1 ml. 150ml cost £42.30
Fluticasone	£6.53	Fluticasone propionate 25 microgram per 1 dose. 120 dose inhaler costs £6.53
Fluticasone	£21.26	Inhaler is used.<br>Fluticasone propionate 125 microgram per 1 dose. £21.26 for 120 doses.
Ibuprofen	£4.20	Assume oral suspension. Ibuprofen 40 mg per 1 ml. 100 ml cost £4.20 (NHS indicative price, no drug tariff available)
Ipratropium bromide 20 mcg	£5.56	Inhaler: Ipratropium bromide 20 microgram per 1 dose. 200 doses cost £5.56.
Magnesium sulphate	£1.735	Magnesium sulfate heptahydrate 500 mg per 1 ml; 10 ampoule cost £17.35
Oxygen	£0.95	Supplemental oxygen per hour, provided by mask or nasal, was estimated to be £0.80 for NHS Lothian in 2013. Prices have been adjusted to their value in 2018 based on a discount rate of 3.5% per year, as recommended for the UK.
Paracetemol 300 mg	£3.78	Oral suspension paediatrics, 120mg/5ml. 200 ml cost £3.78
Prednisolone 5 mg	£11.41	Oral solution: Prednisolone 10 mg per 1 ml: £11.41 for 10 unit dose
Salbutamol/ ventolin	£1.50	Salbutamol (as Salbutamol sulfate) 100 microgram per 1 dose. 200 doses £1.50
Salmeterol 25 mcg	£29.26	Salmeterol (as Salmeterol xinafoate) 25 microgram per 1 dose; 120 doses £29.26
Seretide	£21.26	Fluticasone – costed as above

For peer review only - http://bmjopen.bmj.com/site/about/guidelines.xhtml

Supplemental File 2: Availability of data reported on behalf of the children, and by the children

Availability of EQ-5D-Y data

For the EQ-5D-Y, the versions completed by the proxy on behalf of the children are more complete. The trial team considered substituting by available information from the EQ-5D-Y completed by the children themselves if data were missing from the EQ-5D-Y proxy, to enable a more complete analysis dataset. However, this approach yielded only an additional 1, 2, 3, 4 and 2 observations at days 0, 4, 7, 14 and 28, and was therefore abandoned.

For 54 participants (20%), proxy-completed EQ-5D-Y index data are available at baseline and all follow-up time points.

For peer review only

Supplemental Table S2a: Available EQ-5D-Y data for participants aged less than 2 years

	Co-amoxiclav (N=45)	Placebo (N=44)	Total (N=89)
**Availability of EQ-5D-Y index completed on behalf of child**			
**day 0**	18 (40%)	24 (55%)	42 (47%)
**day 4**	9 (20%)	12 (27%)	21 (24%)
**day 7**	9 (20%)	14 (32%)	23 (26%)
**day 14**	10 (22%)	13 (30%)	23 (26%)
**day 28**	11 (24%)	11 (25%)	22 (25%)
**Availability of EQ-5D VAS index completed by child**			
**day 0**	39 (87%)	43 (98%)	82 (92%)
**day 4**	27 (60%)	29 (66%)	56 (63%)
**day 7**	27 (60%)	28 (64%)	55 (62%)
**day 14**	22 (49%)	24 (55%)	46 (52%)
**day 28**	19 (42%)	19 (43%)	38 (43%)
**Availability of EQ-5D-Y index completed by child**			
**day 0**	0 (0%)	0 (0%)	0 (0%)
**day 4**	0 (0%)	1 (2%)	1 (1%)
**day 7**	0 (0%)	1 (2%)	1 (1%)
**day 14**	0 (0%)	1 (2%)	1 (1%)
**day 28**	0 (0%)	1 (2%)	1 (1%)
**Availability of EQ-5D VAS index completed by child**			
**day 0**	0 (0%)	1 (2%)	1 (1%)
**day 4**	3 (7%)	1 (2%)	4 (4%)
**day 7**	3 (7%)	1 (2%)	4 (4%)
**day 14**	3 (7%)	1 (2%)	4 (4%)
**day 28**	2 (4%)	1 (2%)	3 (3%)

For peer review only - http://bmjopen.bmj.com/site/about/guidelines.xhtml

**Availability of CARIFS**			
**day 0**	27 (60%)	29 (66%)	56 (63%)
**day 7**	21 (47%)	23 (52%)	44 (49%)
**Availability of CARIFS VAS**			
**day 0**	44 (98%)	44 (100%)	88 (99%)
**day 7**	26 (58%)	28 (64%)	54 (61%)

For peer review only

For peer review only - http://bmjopen.bmj.com/site/about/guidelines.xhtml

Supplemental Table S2b: Available EQ-5D-Y data for participants aged 2+ years

	Co-amoxiclav (N=91)	Placebo (N=91)	Total (N=182)
**Availability of EQ-5D-Y index completed on behalf of child**			
**day 0**	78 (86%)	84 (92%)	162 (89%)
**day 4**	43 (47%)	40 (44%)	83 (46%)
**day 7**	43 (47%)	40 (44%)	83 (46%)
**day 14**	34 (37%)	33 (36%)	67 (37%)
**day 28**	30 (33%)	20 (22%)	50 (27%)
**Availability of EQ-5D-Y index completed by child**			
**day 0**	81 (89%)	86 (95%)	167 (92%)
**day 4**	50 (55%)	47 (52%)	97 (53%)
**day 7**	51 (56%)	45 (49%)	96 (53%)
**day 14**	39 (43%)	33 (36%)	72 (40%)
**day 28**	34 (37%)	21 (23%)	55 (30%)
**Availability of EQ-5D-Y index completed by child**			
**day 0**	32 (35%)	29 (32%)	61 (34%)
**day 4**	16 (18%)	12 (13%)	28 (15%)
**day 7**	17 (19%)	10 (11%)	27 (15%)
**day 14**	17 (19%)	9 (10%)	26 (14%)
**day 28**	17 (19%)	9 (10%)	26 (14%)
**Availability of EQ-5D VAS index completed by child**			
**day 0**	28 (31%)	24 (26%)	52 (29%)
**day 4**	16 (18%)	12 (13%)	28 (15%)
**day 7**	16 (18%)	10 (11%)	26 (14%)
**day 14**	17 (19%)	9 (10%)	26 (14%)
**day 28**	16 (18%)	10 (11%)	26 (14%)
**Availability of CARIFS**			
**day 0**	72 (79%)	68 (75%)	140 (77%)
**day 7**	45 (49%)	34 (37%)	79 (43%)
**Availability of CARIFS VAS**			
**day 0**	89 (98%)	91 (100%)	180 (99%)
**day 7**	50 (55%)	46 (51%)	96 (53%)

For peer review only

Supplemental Table S2c: Missing data pattern over time for the EQ-5D-Y proxy, completed on behalf of the child

Missing data pattern*	N	%
+. . . .	104	38.4%
+++++	54	19.9%
. . . . .	48	17.7%
++++.	19	7.0%
+++. .	19	7.0%
.++++	5	1.8%
. . .++	3	1.1%
. .+. .	3	1.1%
. . . .+	3	1.1%
+++.+	2	0.7%
+. .++	2	0.7%
.+++.	2	0.7%
. . .+.	2	0.7%
+.+++	1	0.4%
++.+.	1	0.4%
++. .+	1	0.4%
. .+++	1	0.4%
++. . .	1	0.4%

**+ indicates data are available, . indicates data are missing. The first character refers to the baseline EQ-5D-Y, the second to the 4 day assessment etc.*
For example, the first line of the table (+….) describes participants for whom baseline data, but no data for the subsequent four follow-up visits are available.

Supplemental Table S2d: Proxy-reported EQ-5D-Y scores by data availability at the subsequent follow-up

This table shows EQ-5D-Y values (as reported by their proxies on the children's behalf) at each time point spit by whether or not data was available for the subsequent time point. We hypothesised that those with unavailable data at the subsequent time point would be likely to have worth health states.

There was no consistent pattern to support this hypothesis.

	Participants for whom subsequent follow-up is available	Participants for whom subsequent follow-up is not available
**Day 0**	0.549 (0.396), N=97	0.606 (0.335), N=107
**Day 4**	0.671 (0.318), N=101	0.286 (0.671), N=3
**Day 7**	0.780 (0.339), N=82	0.759 (0.394), N=24
**Day 14**	0.882 (0.280), N=66	0.888 (0.217), N=24

For peer review only - http://bmjopen.bmj.com/site/about/guidelines.xhtml

Supplemental Table S2e: Missing data pattern for the EQ-5D-Y domains (proxy data, completed on behalf of the child)

	**Baseline**		**Day 4**		**Day 7**		**Day 14**		**Day 28**	
	Frequency	%	Frequency	%	Frequency	%	Frequency	%	Frequency	%
+++++	204	75.3%	104	38.4%	106	39.1%	90	33.2%	72	26.6%
.....	5	1.8%	117	43.2%	122	45%	153	56.5%	179	66.1%
+.+++	38	14%	25	9.2%	21	7.7%	13	4.8%	8	3%
..+++	15	5.5%	16	5.9%	16	5.9%	13	4.8%	9	3.3%
.++++	2	0.7%	3	1.1%	2	0.7%	1	0.4%	1	0.4%
+.++.	2	0.7%	1	0.4%	-	-	-	-	-	-
...++	2	0.7%	3	1.1%	-	-	-	-	-	-
++++.	1	0.4%	-	-	-	-	-	-	-	-
..++.	1	0.4%	-	-	-	-	-	-	-	-
...+.	1	0.4%	-	-	-	-	-	-	-	-
+++..	-	-	1	0.4%	-	-	-	-	-	-
+..++	-	-	1	0.4%	-	-	-	-	-	-
+.+.+	-	-	-	-	1	0.4%	-	-	-	-
+...+	-	-	-	-	1	0.4%	-	-	-	-
...++	-	-	-	-	1	0.4%	1	0.4%	-	-
....+	-	-	-	-	1	0.4%	-	-	-	-
++++.	-	-	-	-	-	-	-	-	2	0.7%

**+ indicates data are available, . indicates data are missing. The first character refers to the first EQ-5D item (mobility), the second to the second question (self-care) etc. The pattern +.+++ indicates that the answer to the self-care question is missing, while all other responses are available.*

Particularly the items on self-care and mobility are left incomplete when other questions have been answered.

For peer review only - http://bmjopen.bmj.com/site/about/guidelines.xhtml

Supplemental Table S2f: Available EQ-5D-Y items

	Co-amoxiclav (N=136)						Placebo (N=135)						Total (N=271)					
	**M**	**SC**	**UA**	**PD**	**AD**	**VAS**	**M**	**SC**	**UA**	**PD**	**AD**	**VAS**	**M**	**SC**	**UA**	**PD**	**AD**	**VAS**
**Availability of EQ-5D-3L index proxy, completed on behalf of child**																		
Day 0	120 (88%)	**97 (71%)**	128 (94%)	131 (96%)	127 (93%)	120 (88%)	125 (93%)	**110 (81%)**	135 (100%)	135 (100%)	134 (99%)	129 (96%)	245 (90%)	**207 (76%)**	263 (97%)	266 (98%)	261 (96%)	249 (92%)
Day 4	69 (51%)	**54 (40%)**	75 (55%)	76 (56%)	76 (56%)	77 (57%)	63 (47%)	**54 (40%)**	75 (56%)	77 (57%)	76 (56%)	76 (56%)	132 (49%)	**108 (40%)**	150 (55%)	153 (56%)	152 (56%)	153 (56%)
Day 7	68 (50%)	**52 (38%)**	74 (54%)	75 (55%)	76 (56%)	78 (57%)	61 (45%)	**56 (41%)**	72 (53%)	71 (53%)	73 (54%)	73 (54%)	129 (48%)	**108 (40%)**	146 (54%)	146 (54%)	149 (55%)	151 (56%)
Day 14	53 (39%)	**44 (32%)**	59 (43%)	59 (43%)	59 (43%)	61 (45%)	50 (37%)	**47 (35%)**	58 (43%)	59 (44%)	59 (44%)	57 (42%)	103 (38%)	**91 (34%)**	117 (43%)	118 (44%)	118 (44%)	118 (44%)
Day 28	48 (35%)	**43 (32%)**	53 (39%)	53 (39%)	51 (38%)	53 (39%)	34 (25%)	**32 (24%)**	39 (29%)	39 (29%)	39 (29%)	40 (30%)	82 (30%)	**75 (28%)**	92 (34%)	92 (34%)	90 (33%)	93 (34%)
**Availability of EQ-5D-3L index completed by child**																		
Day 0	32 (24%)	32 (24%)	32 (24%)	32 (24%)	32 (24%)	32 (24%)	30 (22%)	30 (22%)	31 (23%)	31 (23%)	30 (22%)	32 (24%)	62 (23%)	62 (23%)	63 (23%)	63 (23%)	62 (23%)	64 (24%)
Day 4	17 (13%)	16 (12%)	19 (14%)	19 (14%)	19 (14%)	19 (14%)	13 (10%)	13 (10%)	13 (10%)	13 (10%)	13 (10%)	13 (10%)	30 (11%)	29 (11%)	32 (12%)	32 (12%)	32 (12%)	32 (12%)
Day 7	18 (13%)	17 (13%)	20 (15%)	20 (15%)	20 (15%)	19 (14%)	11 (8%)	11 (8%)	11 (8%)	11 (8%)	11 (8%)	11 (8%)	29 (11%)	28 (10%)	31 (11%)	31 (11%)	31 (11%)	30 (11%)
Day 14	18 (13%)	17 (13%)	20 (15%)	20 (15%)	20 (15%)	20 (15%)	10 (7%)	10 (7%)	10 (7%)	10 (7%)	10 (7%)	10 (7%)	28 (10%)	27 (10%)	30 (11%)	30 (11%)	30 (11%)	30 (11%)
Day 28	17 (13%)	17 (13%)	19 (14%)	19 (14%)	19 (14%)	18 (13%)	10 (7%)	10 (7%)	10 (7%)	10 (7%)	10 (7%)	11 (8%)	27 (10%)	27 (10%)	29 (11%)	29 (11%)	29 (11%)	29 (11%)

M – mobility, SC – Self-care, UA – Usual activities, PD – Pain/ discomfort, AD – Anxiety/ depression
Items with higher rates of missing data are highlighted in bold

For peer review only

Supplemental Table S2g: Available EQ-5D-Y items for children aged less than 2 years

	Co-amoxiclav (N=45)						Placebo (N=44)						Total (N=89)					
	M	SC	UA	PD	AD	VAS	M	SC	UA	PD	AD	VAS	M	SC	UA	PD	AD	VAS
**Availability of EQ-5D-3L index proxy, completed on behalf of child**																		
Day 0	34 (76%)	**18 (40%)**	40 (89%)	43 (96%)	39 (87%)	39 (87%)	**35 (80%)**	**25 (57%)**	44 (100%)	44 (100%)	44 (100%)	43 (98%)	69 (78%)	**43 (48%)**	84 (94%)	87 (98%)	83 (93%)	82 (92%)
Day 4	20 (44%)	**10 (22%)**	25 (56%)	27 (60%)	27 (60%)	27 (60%)	**19 (43%)**	**13 (30%)**	27 (61%)	29 (66%)	28 (64%)	29 (66%)	39 (44%)	**23 (26%)**	52 (58%)	56 (63%)	55 (62%)	56 (63%)
Day 7	19 (42%)	**9 (20%)**	24 (53%)	25 (56%)	26 (58%)	27 (60%)	**18 (41%)**	**15 (34%)**	26 (59%)	26 (59%)	27 (61%)	28 (64%)	37 (42%)	**24 (27%)**	50 (56%)	51 (57%)	53 (60%)	55 (62%)
Day 14	17 (38%)	**10 (22%)**	22 (49%)	22 (49%)	22 (49%)	22 (49%)	**16 (36%)**	**14 (32%)**	24 (55%)	25 (57%)	25 (57%)	24 (55%)	33 (37%)	**24 (27%)**	46 (52%)	47 (53%)	47 (53%)	46 (52%)
Day 28	15 (33%)	**11 (24%)**	20 (44%)	20 (44%)	20 (44%)	19 (42%)	**14 (32%)**	**12 (27%)**	19 (43%)	19 (43%)	19 (43%)	19 (43%)	29 (33%)	**23 (26%)**	39 (44%)	39 (44%)	39 (44%)	38 (43%)
**Availability of EQ-5D-3L index completed by child**																		
Day 0	0 (0%)	0 (0%)	0 (0%)	0 (0%)	0 (0%)	0 (0%)	0 (0%)	0 (0%)	1 (2%)	1 (2%)	1 (2%)	1 (2%)	0 (0%)	0 (0%)	1 (1%)	1 (1%)	1 (1%)	1 (1%)
Day 4	1 (2%)	0 (0%)	3 (7%)	3 (7%)	3 (7%)	3 (7%)	1 (2%)	1 (2%)	1 (2%)	1 (2%)	1 (2%)	1 (2%)	2 (2%)	1 (1%)	4 (4%)	4 (4%)	4 (4%)	4 (4%)
Day 7	1 (2%)	0 (0%)	3 (7%)	3 (7%)	3 (7%)	3 (7%)	1 (2%)	1 (2%)	1 (2%)	1 (2%)	1 (2%)	1 (2%)	2 (2%)	1 (1%)	4 (4%)	4 (4%)	4 (4%)	4 (4%)
Day 14	1 (2%)	0 (0%)	3 (7%)	3 (7%)	3 (7%)	3 (7%)	1 (2%)	1 (2%)	1 (2%)	1 (2%)	1 (2%)	1 (2%)	2 (2%)	1 (1%)	4 (4%)	4 (4%)	4 (4%)	4 (4%)
Day 28	0 (0%)	0 (0%)	2 (4%)	2 (4%)	2 (4%)	2 (4%)	1 (2%)	1 (2%)	1 (2%)	1 (2%)	1 (2%)	1 (2%)	1 (1%)	1 (1%)	3 (3%)	3 (3%)	3 (3%)	3 (3%)

M – mobility, SC – Self-care, UA – Usual activities, PD – Pain/ discomfort, AD – Anxiety/ depression
Items with higher rates of missing data are highlighted in bold

For peer review only - http://bmjopen.bmj.com/site/about/guidelines.xhtml

Supplemental Table S2h: Available EQ-5D-Y items for children aged 2+ years (proxy)

	Co-amoxiclav (N=91)						Placebo (N=91)						Total (N=182)					
	M	SC	UA	PD	AD	VAS	M	SC	UA	PD	AD	VAS	M	SC	UA	PD	AD	VAS
**Availability of EQ-5D-3L index proxy, completed on behalf of child**																		
Day 0	86 (95%)	**79 (87%)**	88 (97%)	88 (97%)	88 (97%)	81 (89%)	90 (99%)	**85 (93%)**	91 (100%)	91 (100%)	90 (99%)	86 (95%)	176 (97%)	**164 (90%)**	179 (98%)	179 (98%)	178 (98%)	**167 (92%)**
Day 4	49 (54%)	**44 (48%)**	50 (55%)	49 (54%)	49 (54%)	50 (55%)	44 (48%)	**41 (45%)**	48 (53%)	48 (53%)	48 (53%)	47 (52%)	93 (51%)	**85 (47%)**	98 (54%)	97 (53%)	97 (53%)	97 (53%)
Day 7	49 (54%)	**43 (47%)**	50 (55%)	50 (55%)	50 (55%)	51 (56%)	43 (47%)	41 (45%)	46 (51%)	45 (49%)	46 (51%)	45 (49%)	92 (51%)	84 (46%)	96 (53%)	95 (52%)	96 (53%)	96 (53%)
Day 14	36 (40%)	34 (37%)	37 (41%)	37 (41%)	37 (41%)	39 (43%)	34 (37%)	33 (36%)	34 (37%)	34 (37%)	34 (37%)	33 (36%)	70 (38%)	67 (37%)	71 (39%)	71 (39%)	71 (39%)	72 (40%)
Day 28	33 (36%)	32 (35%)	33 (36%)	33 (36%)	31 (34%)	34 (37%)	20 (22%)	20 (22%)	20 (22%)	20 (22%)	20 (22%)	21 (23%)	53 (29%)	52 (29%)	53 (29%)	53 (29%)	51 (28%)	55 (30%)
**Availability of EQ-5D-3L index completed by child**																		
Day 0	32 (35%)	32 (35%)	32 (35%)	32 (35%)	32 (35%)	32 (35%)	30 (33%)	30 (33%)	30 (33%)	30 (33%)	29 (32%)	31 (34%)	62 (34%)	62 (34%)	62 (34%)	62 (34%)	61 (34%)	63 (35%)
Day 4	16 (18%)	16 (18%)	16 (18%)	16 (18%)	16 (18%)	16 (18%)	12 (13%)	12 (13%)	12 (13%)	12 (13%)	12 (13%)	12 (13%)	28 (15%)	28 (15%)	28 (15%)	28 (15%)	28 (15%)	28 (15%)
Day 7	17 (19%)	17 (19%)	17 (19%)	17 (19%)	17 (19%)	16 (18%)	10 (11%)	10 (11%)	10 (11%)	10 (11%)	10 (11%)	10 (11%)	27 (15%)	27 (15%)	27 (15%)	27 (15%)	27 (15%)	26 (14%)
Day 14	17 (19%)	17 (19%)	17 (19%)	17 (19%)	17 (19%)	17 (19%)	9 (10%)	9 (10%)	9 (10%)	9 (10%)	9 (10%)	9 (10%)	26 (14%)	26 (14%)	26 (14%)	26 (14%)	26 (14%)	26 (14%)
Day 28	17 (19%)	17 (19%)	17 (19%)	17 (19%)	17 (19%)	16 (18%)	9 (10%)	9 (10%)	9 (10%)	9 (10%)	9 (10%)	10 (11%)	26 (14%)	26 (14%)	26 (14%)	26 (14%)	26 (14%)	26 (14%)

M – mobility, SC – Self-care, UA – Usual activities, PD – Pain/ discomfort, AD – Anxiety/ depression
Items with higher rates of missing data are highlighted in bold

For peer review only - http://bmjopen.bmj.com/site/about/guidelines.xhtml

Supplemental File 3: Reponses to the EQ-5D-Y items

The following tables present the responses to the EQ-5D-Y items at the relevant time points, both for proxy-completed and self-completed questionnaires. Additional tables also show responses separately for children aged less than 2 years, and 2+ years. The summaries include participants for whom the EQ-5D-Y index is available, i.e. for whom all five responses were available.

For peer review only

For peer review only - http://bmjopen.bmj.com/site/about/guidelines.xhtml

Supplemental Table S3a: Responses to EQ-5D-Y domains (proxy, completed on behalf of child)

	M	SC	UA	PD	AD	M	SC	UA	PD	AD	M	SC	UA	PD	AD
**Baseline**	Co-amoxiclav (N = 96)					Placebo (N = 108)					Total (N = 204)				
**No Problems**	78 (81%)	65 (68%)	37 (39%)	22 (23%)	35 (36%)	80 (74%)	67 (62%)	44 (41%)	33 (31%)	49 (45%)	158 (77%)	132 (65%)	81 (40%)	55 (27%)	84 (41%)
**Moderate problems**	11 (11%)	22 (23%)	44 (46%)	67 (70%)	55 (57%)	21 (19%)	22 (20%)	52 (48%)	63 (58%)	51 (47%)	32 (16%)	44 (22%)	96 (47%)	130 (64%)	106 (52%)
**Extreme problems**	7 (7%)	9 (9%)	15 (16%)	7 (7%)	6 (6%)	7 (6%)	19 (18%)	12 (11%)	12 (11%)	8 (7%)	14 (7%)	28 (14%)	27 (13%)	19 (9%)	14 (7%)
**Day 4**	Co-amoxiclav (N = 52)					Placebo (N = 52)					Total (N = 104)				
**No Problems**	38 (73%)	40 (77%)	22 (42%)	18 (35%)	26 (50%)	39 (75%)	34 (65%)	24 (46%)	22 (42%)	32 (62%)	77 (74%)	74 (71%)	46 (44%)	40 (38%)	58 (56%)
**Moderate problems**	10 (19%)	8 (15%)	20 (38%)	31 (60%)	24 (46%)	12 (23%)	14 (27%)	22 (42%)	28 (54%)	19 (37%)	22 (21%)	22 (21%)	42 (40%)	59 (57%)	43 (41%)
**Extreme problems**	4 (8%)	4 (8%)	10 (19%)	3 (6%)	2 (4%)	1 (2%)	4 (8%)	6 (12%)	2 (4%)	1 (2%)	5 (5%)	8 (8%)	16 (15%)	5 (5%)	3 (3%)
**Day 7**	Co-amoxiclav (N = 52)					Placebo (N = 54)					Total (N = 106)				
**No Problems**	44 (85%)	46 (88%)	38 (73%)	32 (62%)	43 (83%)	46 (85%)	41 (76%)	33 (61%)	33 (61%)	41 (76%)	90 (85%)	87 (82%)	71 (67%)	65 (61%)	84 (79%)
**Moderate problems**	3 (6%)	2 (4%)	11 (21%)	17 (33%)	8 (15%)	6 (11%)	8 (15%)	18 (33%)	18 (33%)	12 (22%)	9 (8%)	10 (9%)	29 (27%)	35 (33%)	20 (19%)
**Extreme problems**	5 (10%)	4 (8%)	3 (6%)	3 (6%)	1 (2%)	2 (4%)	5 (9%)	3 (6%)	3 (6%)	1 (2%)	7 (7%)	9 (8%)	6 (6%)	6 (6%)	2 (2%)
**Day 14**	Co-amoxiclav (N = 44)					Placebo (N = 46)					Total (N = 90)				
**No Problems**	41 (93%)	40 (91%)	35 (80%)	35 (80%)	38 (86%)	43 (93%)	40 (87%)	40 (87%)	39 (85%)	40 (87%)	84 (93%)	80 (89%)	75 (83%)	74 (82%)	78 (87%)
**Moderate problems**	0 (0%)	2 (5%)	7 (16%)	6 (14%)	5 (11%)	3 (7%)	5 (11%)	6 (13%)	7 (15%)	6 (13%)	3 (3%)	7 (8%)	13 (14%)	13 (14%)	11 (12%)
**Extreme problems**	3 (7%)	2 (5%)	2 (5%)	3 (7%)	1 (2%)	0 (0%)	1 (2%)	0 (0%)	0 (0%)	0 (0%)	3 (3%)	3 (3%)	2 (2%)	3 (3%)	1 (1%)
**Day 28**	Co-amoxiclav (N = 41)					Placebo (N = 31)					Total (N = 72)				
**No Problems**	39 (95%)	39 (95%)	37 (90%)	35 (85%)	36 (88%)	28 (90%)	28 (90%)	28 (90%)	26 (84%)	28 (90%)	67 (93%)	67 (93%)	65 (90%)	61 (85%)	64 (89%)
**Moderate problems**	0 (0%)	0 (0%)	4 (10%)	5 (12%)	5 (12%)	3 (10%)	2 (6%)	3 (10%)	5 (16%)	3 (10%)	3 (4%)	2 (3%)	7 (10%)	10 (14%)	8 (11%)
**Extreme problems**	2 (5%)	2 (5%)	0 (0%)	1 (2%)	0 (0%)	0 (0%)	1 (3%)	0 (0%)	0 (0%)	0 (0%)	2 (3%)	3 (4%)	0 (0%)	1 (1%)	0 (0%)

M – mobility, SC – Self-care, UA – Usual activities, PD – Pain/ discomfort, AD – Anxiety/ depression

For peer review only

For peer review only - http://bmjopen.bmj.com/site/about/guidelines.xhtml

Supplemental Table S3b: Responses to EQ-5D-Y domains (proxy, completed on behalf of child for children aged less than 2 years)

	M	SC	UA	PD	AD	M	SC	UA	PD	AD	M	SC	UA	PD	AD
**Baseline**	Co-amoxiclav (N = 18)					Placebo (N = 24)					Total (N = 42)				
**No Problems**	14 (78%)	15 (83%)	11 (61%)	4 (22%)	6 (33%)	19 (79%)	13 (54%)	13 (54%)	8 (33%)	7 (29%)	33 (79%)	28 (67%)	24 (57%)	12 (29%)	13 (31%)
**Moderate problems**	2 (11%)	0 (0%)	5 (28%)	14 (78%)	11 (61%)	4 (17%)	5 (21%)	11 (46%)	16 (67%)	15 (63%)	6 (14%)	5 (12%)	16 (38%)	30 (71%)	26 (62%)
**Extreme problems**	2 (11%)	3 (17%)	2 (11%)	0 (0%)	1 (6%)	1 (4%)	6 (25%)	0 (0%)	0 (0%)	2 (8%)	3 (7%)	9 (21%)	2 (5%)	0 (0%)	3 (7%)
**Day 4**	Co-amoxiclav (N = 9)					Placebo (N = 12)					Total (N = 21)				
**No Problems**	7 (78%)	7 (78%)	6 (67%)	4 (44%)	6 (67%)	10 (83%)	9 (75%)	7 (58%)	4 (33%)	6 (50%)	17 (81%)	16 (76%)	13 (62%)	8 (38%)	12 (57%)
**Moderate problems**	1 (11%)	1 (11%)	2 (22%)	5 (56%)	3 (33%)	2 (17%)	2 (17%)	5 (42%)	8 (67%)	6 (50%)	3 (14%)	3 (14%)	7 (33%)	13 (62%)	9 (43%)
**Extreme problems**	1 (11%)	1 (11%)	1 (11%)	0 (0%)	0 (0%)	0 (0%)	1 (8%)	0 (0%)	0 (0%)	0 (0%)	1 (5%)	2 (10%)	1 (5%)	0 (0%)	0 (0%)
**Day 7**	Co-amoxiclav (N = 9)					Placebo (N = 14)					Total (N = 23)				
**No Problems**	8 (89%)	8 (89%)	7 (78%)	7 (78%)	9 (100%)	11 (79%)	10 (71%)	9 (64%)	8 (57%)	10 (71%)	19 (83%)	18 (78%)	16 (70%)	15 (65%)	19 (83%)
**Moderate problems**	0 (0%)	0 (0%)	1 (11%)	2 (22%)	0 (0%)	3 (21%)	3 (21%)	5 (36%)	6 (43%)	4 (29%)	3 (13%)	3 (13%)	6 (26%)	8 (35%)	4 (17%)
**Extreme problems**	1 (11%)	1 (11%)	1 (11%)	0 (0%)	0 (0%)	0 (0%)	1 (7%)	0 (0%)	0 (0%)	0 (0%)	1 (4%)	2 (9%)	1 (4%)	0 (0%)	0 (0%)
**Day 14**	Co-amoxiclav (N = 10)					Placebo (N = 13)					Total (N = 23)				
**No Problems**	10 (100%)	9 (90%)	8 (80%)	7 (70%)	8 (80%)	11 (85%)	10 (77%)	10 (77%)	10 (77%)	10 (77%)	21 (91%)	19 (83%)	18 (78%)	17 (74%)	18 (78%)
**Moderate problems**	0 (0%)	1 (10%)	2 (20%)	2 (20%)	2 (20%)	2 (15%)	3 (23%)	3 (23%)	3 (23%)	3 (23%)	2 (9%)	4 (17%)	5 (22%)	5 (22%)	5 (22%)
**Extreme problems**	0 (0%)	0 (0%)	0 (0%)	1 (10%)	0 (0%)	0 (0%)	0 (0%)	0 (0%)	0 (0%)	0 (0%)	0 (0%)	0 (0%)	0 (0%)	1 (4%)	0 (0%)
**Day 28**	Co-amoxiclav (N = 11)					Placebo (N = 11)					Total (N = 22)				
**No Problems**	11 (100%)	11 (100%)	11 (100%)	10 (91%)	11 (100%)	10 (91%)	10 (91%)	10 (91%)	8 (73%)	9 (82%)	21 (95%)	21 (95%)	21 (95%)	18 (82%)	20 (91%)
**Moderate problems**	0 (0%)	0 (0%)	0 (0%)	1 (9%)	0 (0%)	1 (9%)	1 (9%)	1 (9%)	3 (27%)	2 (18%)	1 (5%)	1 (5%)	1 (5%)	4 (18%)	2 (9%)
**Extreme problems**	0 (0%)	0 (0%)	0 (0%)	0 (0%)	0 (0%)	0 (0%)	0 (0%)	0 (0%)	0 (0%)	0 (0%)	0 (0%)	0 (0%)	0 (0%)	0 (0%)	0 (0%)

M – mobility, SC – Self-care, UA – Usual activities, PD – Pain/ discomfort, AD – Anxiety/ depression

For peer review only

For peer review only - http://bmjopen.bmj.com/site/about/guidelines.xhtml

Supplemental Table S3c: Responses to EQ-5D-Y domains (proxy, completed on behalf of child for children aged 2+ years)

	M	SC	UA	PD	AD	M	SC	UA	PD	AD	M	SC	UA	PD	AD
**Baseline**	Co-amoxiclav (N = 78)					Placebo (N = 84)					Total (N = 162)				
**No Problems**	64 (82%)	50 (64%)	26 (33%)	18 (23%)	29 (37%)	61 (73%)	54 (64%)	31 (37%)	25 (30%)	42 (50%)	125 (77%)	104 (64%)	57 (35%)	43 (27%)	71 (44%)
**Moderate problems**	9 (12%)	22 (28%)	39 (50%)	53 (68%)	44 (56%)	17 (20%)	17 (20%)	41 (49%)	47 (56%)	36 (43%)	26 (16%)	39 (24%)	80 (49%)	100 (62%)	80 (49%)
**Extreme problems**	5 (6%)	6 (8%)	13 (17%)	7 (9%)	5 (6%)	6 (7%)	13 (15%)	12 (14%)	12 (14%)	6 (7%)	11 (7%)	19 (12%)	25 (15%)	19 (12%)	11 (7%)
**Day 4**	Co-amoxiclav (N = 43)					Placebo (N = 40)					Total (N = 83)				
**No Problems**	31 (72%)	33 (77%)	16 (37%)	14 (33%)	20 (47%)	29 (73%)	25 (63%)	17 (43%)	18 (45%)	26 (65%)	60 (72%)	58 (70%)	33 (40%)	32 (39%)	46 (55%)
**Moderate problems**	9 (21%)	7 (16%)	18 (42%)	26 (60%)	21 (49%)	10 (25%)	12 (30%)	17 (43%)	20 (50%)	13 (33%)	19 (23%)	19 (23%)	35 (42%)	46 (55%)	34 (41%)
**Extreme problems**	3 (7%)	3 (7%)	9 (21%)	3 (7%)	2 (5%)	1 (3%)	3 (8%)	6 (15%)	2 (5%)	1 (3%)	4 (5%)	6 (7%)	15 (18%)	5 (6%)	3 (4%)
**Day 7**	Co-amoxiclav (N = 43)					Placebo (N = 40)					Total (N = 83)				
**No Problems**	36 (84%)	38 (88%)	31 (72%)	25 (58%)	34 (79%)	35 (88%)	31 (78%)	24 (60%)	25 (63%)	31 (78%)	71 (86%)	69 (83%)	55 (66%)	50 (60%)	65 (78%)
**Moderate problems**	3 (7%)	2 (5%)	10 (23%)	15 (35%)	8 (19%)	3 (8%)	5 (13%)	13 (33%)	12 (30%)	8 (20%)	6 (7%)	7 (8%)	23 (28%)	27 (33%)	16 (19%)
**Extreme problems**	4 (9%)	3 (7%)	2 (5%)	3 (7%)	1 (2%)	2 (5%)	4 (10%)	3 (8%)	3 (8%)	1 (3%)	6 (7%)	7 (8%)	5 (6%)	6 (7%)	2 (2%)
**Day 14**	Co-amoxiclav (N = 34)					Placebo (N = 33)					Total (N = 67)				
**No Problems**	31 (91%)	31 (91%)	27 (79%)	28 (82%)	30 (88%)	32 (97%)	30 (91%)	30 (91%)	29 (88%)	30 (91%)	63 (94%)	61 (91%)	57 (85%)	57 (85%)	60 (90%)
**Moderate problems**	0 (0%)	1 (3%)	5 (15%)	4 (12%)	3 (9%)	1 (3%)	2 (6%)	3 (9%)	4 (12%)	3 (9%)	1 (1%)	3 (4%)	8 (12%)	8 (12%)	6 (9%)
**Extreme problems**	3 (9%)	2 (6%)	2 (6%)	2 (6%)	1 (3%)	0 (0%)	1 (3%)	0 (0%)	0 (0%)	0 (0%)	3 (4%)	3 (4%)	2 (3%)	2 (3%)	1 (1%)
**Day 28**	Co-amoxiclav (N = 30)					Placebo (N = 20)					Total (N = 50)				
**No Problems**	28 (93%)	28 (93%)	26 (87%)	25 (83%)	25 (83%)	18 (90%)	18 (90%)	18 (90%)	18 (90%)	19 (95%)	46 (92%)	46 (92%)	44 (88%)	43 (86%)	44 (88%)
**Moderate problems**	0 (0%)	0 (0%)	4 (13%)	4 (13%)	5 (17%)	2 (10%)	1 (5%)	2 (10%)	2 (10%)	1 (5%)	2 (4%)	1 (2%)	6 (12%)	6 (12%)	6 (12%)
**Extreme problems**	2 (7%)	2 (7%)	0 (0%)	1 (3%)	0 (0%)	0 (0%)	1 (5%)	0 (0%)	0 (0%)	0 (0%)	2 (4%)	3 (6%)	0 (0%)	1 (2%)	0 (0%)

M – mobility, SC – Self-care, UA – Usual activities, PD – Pain/ discomfort, AD – Anxiety/ depression

For peer review only

For peer review only - http://bmjopen.bmj.com/site/about/guidelines.xhtml

Supplemental Table S3d: Responses to EQ-5D-Y domains (completed by child)

	M	SC	UA	PD	AD	M	SC	UA	PD	AD	M	SC	UA	PD	AD
**Baseline**	Co-amoxiclav (N = 32)					Placebo (N = 29)					Total (N = 61)				
**No Problems**	20 (63%)	24 (75%)	4 (13%)	6 (19%)	17 (53%)	24 (83%)	23 (79%)	9 (31%)	5 (17%)	13 (45%)	44 (72%)	47 (77%)	13 (21%)	11 (18%)	30 (49%)
**Moderate problems**	10 (31%)	6 (19%)	22 (69%)	16 (50%)	14 (44%)	5 (17%)	5 (17%)	15 (52%)	16 (55%)	14 (48%)	15 (25%)	11 (18%)	37 (61%)	32 (52%)	28 (46%)
**Extreme problems**	2 (6%)	2 (6%)	6 (19%)	10 (31%)	1 (3%)	0 (0%)	1 (3%)	5 (17%)	8 (28%)	2 (7%)	2 (3%)	3 (5%)	11 (18%)	18 (30%)	3 (5%)
**Day 4**	Co-amoxiclav (N = 16)					Placebo (N = 13)					Total (N = 29)				
**No Problems**	13 (81%)	13 (81%)	7 (44%)	5 (31%)	7 (44%)	10 (77%)	10 (77%)	5 (38%)	4 (31%)	7 (54%)	23 (79%)	23 (79%)	12 (41%)	9 (31%)	14 (48%)
**Moderate problems**	2 (13%)	2 (13%)	5 (31%)	10 (63%)	8 (50%)	3 (23%)	3 (23%)	8 (62%)	8 (62%)	6 (46%)	5 (17%)	5 (17%)	13 (45%)	18 (62%)	14 (48%)
**Extreme problems**	1 (6%)	1 (6%)	4 (25%)	1 (6%)	1 (6%)	0 (0%)	0 (0%)	0 (0%)	1 (8%)	0 (0%)	1 (3%)	1 (3%)	4 (14%)	2 (7%)	1 (3%)
**Day 7**	Co-amoxiclav (N = 17)					Placebo (N = 11)					Total (N = 28)				
**No Problems**	14 (82%)	16 (94%)	10 (59%)	12 (71%)	14 (82%)	10 (91%)	8 (73%)	6 (55%)	5 (45%)	7 (64%)	24 (86%)	24 (86%)	16 (57%)	17 (61%)	21 (75%)
**Moderate problems**	2 (12%)	0 (0%)	6 (35%)	4 (24%)	2 (12%)	1 (9%)	3 (27%)	5 (45%)	6 (55%)	4 (36%)	3 (11%)	3 (11%)	11 (39%)	10 (36%)	6 (21%)
**Extreme problems**	1 (6%)	1 (6%)	1 (6%)	1 (6%)	1 (6%)	0 (0%)	0 (0%)	0 (0%)	0 (0%)	0 (0%)	1 (4%)	1 (4%)	1 (4%)	1 (4%)	1 (4%)
**Day 14**	Co-amoxiclav (N = 17)					Placebo (N = 10)					Total (N = 27)				
**No Problems**	16 (94%)	16 (94%)	14 (82%)	16 (94%)	17 (100%)	10 (100%)	9 (90%)	9 (90%)	7 (70%)	9 (90%)	26 (96%)	25 (93%)	23 (85%)	23 (85%)	26 (96%)
**Moderate problems**	1 (6%)	1 (6%)	3 (18%)	1 (6%)	0 (0%)	0 (0%)	1 (10%)	1 (10%)	3 (30%)	1 (10%)	1 (4%)	2 (7%)	4 (15%)	4 (15%)	1 (4%)
**Extreme problems**	0 (0%)	0 (0%)	0 (0%)	0 (0%)	0 (0%)	0 (0%)	0 (0%)	0 (0%)	0 (0%)	0 (0%)	0 (0%)	0 (0%)	0 (0%)	0 (0%)	0 (0%)
**Day 28**	Co-amoxiclav (N = 17)					Placebo (N = 10)					Total (N = 27)				
**No Problems**	17 (100%)	17 (100%)	15 (88%)	17 (100%)	17 (100%)	10 (100%)	10 (100%)	9 (90%)	8 (80%)	8 (80%)	27 (100%)	27 (100%)	24 (89%)	25 (93%)	25 (93%)
**Moderate problems**	0 (0%)	0 (0%)	2 (12%)	0 (0%)	0 (0%)	0 (0%)	0 (0%)	1 (10%)	2 (20%)	2 (20%)	0 (0%)	0 (0%)	3 (11%)	2 (7%)	2 (7%)
**Extreme problems**	0 (0%)	0 (0%)	0 (0%)	0 (0%)	0 (0%)	0 (0%)	0 (0%)	0 (0%)	0 (0%)	0 (0%)	0 (0%)	0 (0%)	0 (0%)	0 (0%)	0 (0%)

M – mobility, SC – Self-care, UA – Usual activities, PD – Pain/ discomfort, AD – Anxiety/ depression

For peer review only

For peer review only - http://bmjopen.bmj.com/site/about/guidelines.xhtml

Supplemental Table S3e: Responses to EQ-5D-Y domains (completed by child for children aged less than 2 years)

	M	SC	UA	PD	AD	M	SC	UA	PD	AD	M	SC	UA	PD	AD
**Baseline**	Co-amoxiclav (N = 0)					Placebo (N = 0)					Total (N = 0)				
**No Problems**	0 (.%)	0 (.%)	0 (.%)	0 (.%)	0 (.%)	0 (.%)	0 (.%)	0 (.%)	0 (.%)	0 (.%)	0 (.%)	0 (.%)	0 (.%)	0 (.%)	0 (.%)
**Moderate problems**	0 (.%)	0 (.%)	0 (.%)	0 (.%)	0 (.%)	0 (.%)	0 (.%)	0 (.%)	0 (.%)	0 (.%)	0 (.%)	0 (.%)	0 (.%)	0 (.%)	0 (.%)
**Extreme problems**	0 (.%)	0 (.%)	0 (.%)	0 (.%)	0 (.%)	0 (.%)	0 (.%)	0 (.%)	0 (.%)	0 (.%)	0 (.%)	0 (.%)	0 (.%)	0 (.%)	0 (.%)
**Day 4**	Co-amoxiclav (N = 0)					Placebo (N = 1)					Total (N = 1)				
**No Problems**	0 (.%)	0 (.%)	0 (.%)	0 (.%)	0 (.%)	1 (100%)	0 (0%)	1 (100%)	0 (0%)	0 (0%)	1 (100%)	0 (0%)	1 (100%)	0 (0%)	0 (0%)
**Moderate problems**	0 (.%)	0 (.%)	0 (.%)	0 (.%)	0 (.%)	0 (0%)	1 (100%)	0 (0%)	1 (100%)	1 (100%)	0 (0%)	1 (100%)	0 (0%)	1 (100%)	1 (100%)
**Extreme problems**	0 (.%)	0 (.%)	0 (.%)	0 (.%)	0 (.%)	0 (0%)	0 (0%)	0 (0%)	0 (0%)	0 (0%)	0 (0%)	0 (0%)	0 (0%)	0 (0%)	0 (0%)
**Day 7**	Co-amoxiclav (N = 0)					Placebo (N = 1)					Total (N = 1)				
**No Problems**	0 (.%)	0 (.%)	0 (.%)	0 (.%)	0 (.%)	1 (100%)	0 (0%)	0 (0%)	0 (0%)	0 (0%)	1 (100%)	0 (0%)	0 (0%)	0 (0%)	0 (0%)
**Moderate problems**	0 (.%)	0 (.%)	0 (.%)	0 (.%)	0 (.%)	0 (0%)	1 (100%)	1 (100%)	1 (100%)	1 (100%)	0 (0%)	1 (100%)	1 (100%)	1 (100%)	1 (100%)
**Extreme problems**	0 (.%)	0 (.%)	0 (.%)	0 (.%)	0 (.%)	0 (0%)	0 (0%)	0 (0%)	0 (0%)	0 (0%)	0 (0%)	0 (0%)	0 (0%)	0 (0%)	0 (0%)
**Day 14**	Co-amoxiclav (N = 0)					Placebo (N = 1)					Total (N = 1)				
**No Problems**	0 (.%)	0 (.%)	0 (.%)	0 (.%)	0 (.%)	1 (100%)	0 (0%)	0 (0%)	0 (0%)	0 (0%)	1 (100%)	0 (0%)	0 (0%)	0 (0%)	0 (0%)
**Moderate problems**	0 (.%)	0 (.%)	0 (.%)	0 (.%)	0 (.%)	0 (0%)	1 (100%)	1 (100%)	1 (100%)	1 (100%)	0 (0%)	1 (100%)	1 (100%)	1 (100%)	1 (100%)
**Extreme problems**	0 (.%)	0 (.%)	0 (.%)	0 (.%)	0 (.%)	0 (0%)	0 (0%)	0 (0%)	0 (0%)	0 (0%)	0 (0%)	0 (0%)	0 (0%)	0 (0%)	0 (0%)
**Day 28**	Co-amoxiclav (N = 0)					Placebo (N = 1)					Total (N = 1)				
**No Problems**	0 (.%)	0 (.%)	0 (.%)	0 (.%)	0 (.%)	1 (100%)	1 (100%)	1 (100%)	0 (0%)	0 (0%)	1 (100%)	1 (100%)	1 (100%)	0 (0%)	0 (0%)
**Moderate problems**	0 (.%)	0 (.%)	0 (.%)	0 (.%)	0 (.%)	0 (0%)	0 (0%)	0 (0%)	1 (100%)	1 (100%)	0 (0%)	0 (0%)	0 (0%)	1 (100%)	1 (100%)
**Extreme problems**	0 (.%)	0 (.%)	0 (.%)	0 (.%)	0 (.%)	0 (0%)	0 (0%)	0 (0%)	0 (0%)	0 (0%)	0 (0%)	0 (0%)	0 (0%)	0 (0%)	0 (0%)

M – mobility, SC – Self-care, UA – Usual activities, PD – Pain/ discomfort, AD – Anxiety/ depression

For peer review only

For peer review only - http://bmjopen.bmj.com/site/about/guidelines.xhtml

Supplemental Table S3f: Responses to EQ-5D-Y domains (completed by child for children aged 2+ years)

	M	SC	UA	PD	AD	M	SC	UA	PD	AD	M	SC	UA	PD	AD
**Baseline**	Co-amoxiclav (N = 32)					Placebo (N = 29)					Total (N = 61)				
**No Problems**	20 (63%)	24 (75%)	4 (13%)	6 (19%)	10 (31%)	24 (83%)	23 (79%)	9 (31%)	5 (17%)	11 (38%)	44 (72%)	47 (77%)	13 (21%)	11 (18%)	21 (34%)
**Moderate problems**	10 (31%)	6 (19%)	22 (69%)	16 (50%)	20 (63%)	5 (17%)	5 (17%)	15 (52%)	16 (55%)	15 (52%)	15 (25%)	11 (18%)	37 (61%)	32 (52%)	35 (57%)
**Extreme problems**	2 (6%)	2 (6%)	6 (19%)	10 (31%)	2 (6%)	0 (0%)	1 (3%)	5 (17%)	8 (28%)	3 (10%)	2 (3%)	3 (5%)	11 (18%)	18 (30%)	5 (8%)
**Day 4**	Co-amoxiclav (N = 16)					Placebo (N = 12)					Total (N = 28)				
**No Problems**	13 (81%)	13 (81%)	7 (44%)	5 (31%)	7 (47%)	9 (75%)	10 (83%)	4 (33%)	4 (33%)	7 (64%)	22 (79%)	23 (82%)	11 (39%)	9 (32%)	14 (54%)
**Moderate problems**	2 (13%)	2 (13%)	5 (31%)	10 (63%)	7 (47%)	3 (25%)	2 (17%)	8 (67%)	7 (58%)	4 (36%)	5 (18%)	4 (14%)	13 (46%)	17 (61%)	11 (42%)
**Extreme problems**	1 (6%)	1 (6%)	4 (25%)	1 (6%)	1 (7%)	0 (0%)	0 (0%)	0 (0%)	1 (8%)	0 (0%)	1 (4%)	1 (4%)	4 (14%)	2 (7%)	1 (4%)
**Day 7**	Co-amoxiclav (N = 17)					Placebo (N = 10)					Total (N = 27)				
**No Problems**	14 (82%)	16 (94%)	10 (59%)	12 (71%)	13 (81%)	9 (90%)	8 (80%)	6 (60%)	5 (50%)	7 (78%)	23 (85%)	24 (89%)	16 (59%)	17 (63%)	20 (80%)
**Moderate problems**	2 (12%)	0 (0%)	6 (35%)	4 (24%)	3 (19%)	1 (10%)	2 (20%)	4 (40%)	5 (50%)	2 (22%)	3 (11%)	2 (7%)	10 (37%)	9 (33%)	5 (20%)
**Extreme problems**	1 (6%)	1 (6%)	1 (6%)	1 (6%)	0 (0%)	0 (0%)	0 (0%)	0 (0%)	0 (0%)	0 (0%)	1 (4%)	1 (4%)	1 (4%)	1 (4%)	0 (0%)
**Day 14**	Co-amoxiclav (N = 17)					Placebo (N = 9)					Total (N = 26)				
**No Problems**	16 (94%)	16 (94%)	14 (82%)	16 (94%)	14 (93%)	9 (100%)	9 (100%)	9 (100%)	7 (78%)	8 (100%)	25 (96%)	25 (96%)	23 (88%)	23 (88%)	22 (96%)
**Moderate problems**	1 (6%)	1 (6%)	3 (18%)	1 (6%)	1 (7%)	0 (0%)	0 (0%)	0 (0%)	2 (22%)	0 (0%)	1 (4%)	1 (4%)	3 (12%)	3 (12%)	1 (4%)
**Extreme problems**	0 (0%)	0 (0%)	0 (0%)	0 (0%)	0 (0%)	0 (0%)	0 (0%)	0 (0%)	0 (0%)	0 (0%)	0 (0%)	0 (0%)	0 (0%)	0 (0%)	0 (0%)
**Day 28**	Co-amoxiclav (N = 17)					Placebo (N = 9)					Total (N = 26)				
**No Problems**	17 (100%)	17 (100%)	15 (88%)	17 (100%)	15 (94%)	9 (100%)	9 (100%)	8 (89%)	8 (89%)	7 (88%)	26 (100%)	26 (100%)	23 (88%)	25 (96%)	22 (92%)
**Moderate problems**	0 (0%)	0 (0%)	2 (12%)	0 (0%)	1 (6%)	0 (0%)	0 (0%)	1 (11%)	1 (11%)	1 (13%)	0 (0%)	0 (0%)	3 (12%)	1 (4%)	2 (8%)
**Extreme problems**	0 (0%)	0 (0%)	0 (0%)	0 (0%)	0 (0%)	0 (0%)	0 (0%)	0 (0%)	0 (0%)	0 (0%)	0 (0%)	0 (0%)	0 (0%)	0 (0%)	0 (0%)

M – mobility, SC – Self-care, UA – Usual activities, PD – Pain/ discomfort, AD – Anxiety/ depression

For peer review only

For peer review only - http://bmjopen.bmj.com/site/about/guidelines.xhtml

Supplemental Table 3g: Responses to CARIFS domains (only including questionnaires with sufficient data to calculate the overall CARIFS score)

		Co-amoxiclav Baseline (N = 99) Day 7 (N = 66)					Placebo Baseline (N = 97) Day 7 (N = 57)				
		No Problem	Minor Problem	Moderate Problem	Major Problem	Missing	No Problem	Minor Problem	Moderate Problem	Major Problem	Missing
**Poor appetite**	**Baseline**	21 (21%)	36 (36%)	32 (32%)	10 (10%)	0 (0%)	16 (16%)	33 (34%)	39 (40%)	9 (9%)	0 (0%)
	**Day 7**	38 (58%)	21 (32%)	6 (9%)	1 (2%)	0 (0%)	23 (40%)	23 (40%)	7 (12%)	4 (7%)	0 (0%)
**Not sleeping well**	**Baseline**	13 (13%)	30 (30%)	40 (40%)	16 (16%)	0 (0%)	16 (16%)	24 (25%)	38 (39%)	19 (20%)	0 (0%)
	**Day 7**	44 (67%)	12 (18%)	8 (12%)	2 (3%)	0 (0%)	23 (40%)	19 (33%)	8 (14%)	7 (12%)	0 (0%)
**Irritable, cranky, fussy**	**Baseline**	18 (18%)	32 (32%)	40 (40%)	9 (9%)	0 (0%)	15 (15%)	24 (25%)	45 (46%)	13 (13%)	0 (0%)
	**Day 7**	30 (45%)	26 (39%)	7 (11%)	3 (5%)	0 (0%)	23 (40%)	18 (32%)	11 (19%)	5 (9%)	0 (0%)
**Feels unwell**	**Baseline**	4 (4%)	29 (29%)	46 (46%)	20 (20%)	0 (0%)	9 (9%)	22 (23%)	48 (49%)	18 (19%)	0 (0%)
	**Day 7**	39 (59%)	18 (27%)	7 (11%)	2 (3%)	0 (0%)	28 (49%)	16 (28%)	10 (18%)	3 (5%)	0 (0%)
**Low energy tired**	**Baseline**	14 (14%)	30 (30%)	41 (41%)	14 (14%)	0 (0%)	13 (13%)	31 (32%)	41 (42%)	12 (12%)	0 (0%)
	**Day 7**	38 (58%)	20 (30%)	7 (11%)	1 (2%)	0 (0%)	28 (49%)	16 (28%)	10 (18%)	3 (5%)	0 (0%)
**Not playing well**	**Baseline**	27 (27%)	36 (36%)	29 (29%)	7 (7%)	0 (0%)	34 (35%)	28 (29%)	25 (26%)	10 (10%)	0 (0%)
	**Day 7**	50 (76%)	13 (20%)	2 (3%)	1 (2%)	0 (0%)	38 (67%)	9 (16%)	9 (16%)	1 (2%)	0 (0%)
**Crying more than usual**	**Baseline**	37 (37%)	28 (28%)	25 (25%)	9 (9%)	0 (0%)	32 (33%)	26 (27%)	27 (28%)	12 (12%)	0 (0%)
	**Day 7**	40 (61%)	17 (26%)	6 (9%)	3 (5%)	0 (0%)	33 (58%)	16 (28%)	6 (11%)	2 (4%)	0 (0%)
**Needing extra care**	**Baseline**	23 (23%)	34 (34%)	36 (36%)	6 (6%)	0 (0%)	20 (21%)	33 (34%)	36 (37%)	8 (8%)	0 (0%)
	**Day 7**	43 (65%)	19 (29%)	2 (3%)	2 (3%)	0 (0%)	32 (56%)	17 (30%)	6 (11%)	2 (4%)	0 (0%)

For peer review only

For peer review only - http://bmjopen.bmj.com/site/about/guidelines.xhtml

		Co-amoxiclav Baseline (N = 99) Day 7 (N = 66) No Problem	Minor Problem	Moderate Problem	Major Problem	Missing	Placebo Baseline (N = 97) Day 7 (N = 57) No Problem	Minor Problem	Moderate Problem	Major Problem	Missing
**Clinginess**	**Baseline**	16 (16%)	32 (32%)	38 (38%)	13 (13%)	0 (0%)	23 (24%)	25 (26%)	34 (35%)	15 (15%)	0 (0%)
	**Day 7**	40 (61%)	17 (26%)	7 (11%)	2 (3%)	0 (0%)	29 (51%)	16 (28%)	9 (16%)	3 (5%)	0 (0%)
**Headache**	**Baseline**	38 (38%)	20 (20%)	24 (24%)	1 (1%)	16 (16%)	44 (45%)	17 (18%)	10 (10%)	8 (8%)	18 (19%)
	**Day 7**	47 (71%)	7 (11%)	2 (3%)	1 (2%)	9 (14%)	43 (75%)	4 (7%)	3 (5%)	2 (4%)	5 (9%)
**Sore throat**	**Baseline**	29 (29%)	21 (21%)	31 (31%)	8 (8%)	10 (10%)	32 (33%)	21 (22%)	16 (16%)	15 (15%)	13 (13%)
	**Day 7**	47 (71%)	8 (12%)	3 (5%)	1 (2%)	7 (11%)	33 (58%)	12 (21%)	4 (7%)	1 (2%)	7 (12%)
**Muscle aches and pains**	**Baseline**	41 (41%)	18 (18%)	21 (21%)	5 (5%)	14 (14%)	38 (39%)	19 (20%)	11 (11%)	11 (11%)	18 (19%)
	**Day 7**	44 (67%)	10 (15%)	0 (0%)	2 (3%)	10 (15%)	38 (67%)	8 (14%)	4 (7%)	1 (2%)	6 (11%)
**Fever**	**Baseline**	15 (15%)	35 (35%)	35 (35%)	14 (14%)	0 (0%)	16 (16%)	30 (31%)	34 (35%)	17 (18%)	0 (0%)
	**Day 7**	61 (92%)	4 (6%)	1 (2%)	0 (0%)	0 (0%)	48 (84%)	4 (7%)	3 (5%)	2 (4%)	0 (0%)
**Cough**	**Baseline**	4 (4%)	18 (18%)	52 (53%)	25 (25%)	0 (0%)	3 (3%)	19 (20%)	38 (39%)	37 (38%)	0 (0%)
	**Day 7**	21 (32%)	29 (44%)	11 (17%)	5 (8%)	0 (0%)	13 (23%)	18 (32%)	15 (26%)	11 (19%)	0 (0%)
**Nasal congestion, runny nose**	**Baseline**	5 (5%)	22 (22%)	50 (51%)	22 (22%)	0 (0%)	7 (7%)	24 (25%)	41 (42%)	25 (26%)	0 (0%)
	**Day 7**	21 (32%)	33 (50%)	11 (17%)	1 (2%)	0 (0%)	16 (28%)	19 (33%)	16 (28%)	6 (11%)	0 (0%)
**Vomiting**	**Baseline**	73 (74%)	21 (21%)	3 (3%)	2 (2%)	0 (0%)	80 (82%)	9 (9%)	5 (5%)	3 (3%)	0 (0%)
	**Day 7**	62 (94%)	3 (5%)	0 (0%)	1 (2%)	0 (0%)	51 (89%)	6 (11%)	0 (0%)	0 (0%)	0 (0%)
**Not interested in what's going on**	**Baseline**	60 (61%)	26 (26%)	12 (12%)	1 (1%)	0 (0%)	55 (57%)	26 (27%)	11 (11%)	5 (5%)	0 (0%)
	**Day 7**	58 (88%)	7 (11%)	0 (0%)	1 (2%)	0 (0%)	47 (82%)	6 (11%)	3 (5%)	1 (2%)	0 (0%)

For peer review only - http://bmjopen.bmj.com/site/about/guidelines.xhtml

		Co-amoxiclav Baseline (N = 99) Day 7 (N = 66)					Placebo Baseline (N = 97) Day 7 (N = 57)				
		No Problem	Minor Problem	Moderate Problem	Major Problem	Missing	No Problem	Minor Problem	Moderate Problem	Major Problem	Missing
**Unable to get out of bed**	**Baseline**	79 (80%)	16 (16%)	4 (4%)	0 (0%)	0 (0%)	74 (76%)	14 (14%)	8 (8%)	1 (1%)	0 (0%)
	**Day 7**	62 (94%)	3 (5%)	1 (2%)	0 (0%)	0 (0%)	52 (91%)	3 (5%)	2 (4%)	0 (0%)	0 (0%)

Note: The scoring algorithm used allowed for the total score to be calculated if there was missing data in items 10-12, but not in any other items.

For peer review only

For peer review only - http://bmjopen.bmj.com/site/about/guidelines.xhtml

Supplemental Table 3h: Responses to CARIFS domains for children less than 2 years old (only including questionnaires with sufficient data to calculate the overall CARIFS score)

		**Co-amoxiclav** Baseline (N = 27) Day 7 (N = 21)					**Placebo** Baseline (N = 29) Day 7 (N = 23)				
		**No Problem**	**Minor Problem**	**Moderate Problem**	**Major Problem**	**Missing**	**No Problem**	**Minor Problem**	**Moderate Problem**	**Major Problem**	**Missing**
**Poor appetite**	**Baseline**	7 (26%)	10 (37%)	7 (26%)	3 (11%)	0 (0%)	4 (14%)	13 (45%)	9 (31%)	3 (10%)	0 (0%)
	**Day 7**	14 (67%)	4 (19%)	3 (14%)	0 (0%)	0 (0%)	11 (48%)	7 (30%)	3 (13%)	2 (9%)	0 (0%)
**Not sleeping well**	**Baseline**	3 (11%)	12 (44%)	7 (26%)	5 (19%)	0 (0%)	4 (14%)	7 (24%)	12 (41%)	6 (21%)	0 (0%)
	**Day 7**	13 (62%)	5 (24%)	3 (14%)	0 (0%)	0 (0%)	10 (43%)	7 (30%)	3 (13%)	3 (13%)	0 (0%)
**Irritable, cranky, fussy**	**Baseline**	5 (19%)	10 (37%)	10 (37%)	2 (7%)	0 (0%)	2 (7%)	5 (17%)	17 (59%)	5 (17%)	0 (0%)
	**Day 7**	12 (57%)	6 (29%)	3 (14%)	0 (0%)	0 (0%)	8 (35%)	7 (30%)	7 (30%)	1 (4%)	0 (0%)
**Feels unwell**	**Baseline**	3 (11%)	11 (41%)	7 (26%)	6 (22%)	0 (0%)	2 (7%)	6 (21%)	18 (62%)	3 (10%)	0 (0%)
	**Day 7**	12 (57%)	6 (29%)	3 (14%)	0 (0%)	0 (0%)	9 (39%)	9 (39%)	5 (22%)	0 (0%)	0 (0%)
**Low energy tired**	**Baseline**	7 (26%)	12 (44%)	5 (19%)	3 (11%)	0 (0%)	2 (7%)	12 (41%)	14 (48%)	1 (3%)	0 (0%)
	**Day 7**	15 (71%)	6 (29%)	0 (0%)	0 (0%)	0 (0%)	11 (48%)	6 (26%)	6 (26%)	0 (0%)	0 (0%)
**Not playing well**	**Baseline**	12 (44%)	9 (33%)	3 (11%)	3 (11%)	0 (0%)	10 (34%)	11 (38%)	7 (24%)	1 (3%)	0 (0%)
	**Day 7**	16 (76%)	4 (19%)	1 (5%)	0 (0%)	0 (0%)	16 (70%)	3 (13%)	4 (17%)	0 (0%)	0 (0%)
**Crying more than usual**	**Baseline**	7 (26%)	10 (37%)	5 (19%)	5 (19%)	0 (0%)	4 (14%)	8 (28%)	13 (45%)	4 (14%)	0 (0%)
	**Day 7**	12 (57%)	7 (33%)	1 (5%)	1 (5%)	0 (0%)	12 (52%)	7 (30%)	4 (17%)	0 (0%)	0 (0%)
**Needing extra care**	**Baseline**	7 (26%)	10 (37%)	8 (30%)	2 (7%)	0 (0%)	4 (14%)	11 (38%)	13 (45%)	1 (3%)	0 (0%)
	**Day 7**	14 (67%)	7 (33%)	0 (0%)	0 (0%)	0 (0%)	11 (48%)	6 (26%)	5 (22%)	1 (4%)	0 (0%)
**Clinginess**	**Baseline**	2 (7%)	12 (44%)	9 (33%)	4 (15%)	0 (0%)	2 (7%)	6 (21%)	14 (48%)	7 (24%)	0 (0%)
	**Day 7**	10 (48%)	7 (33%)	4 (19%)	0 (0%)	0 (0%)	6 (26%)	8 (35%)	7 (30%)	2 (9%)	0 (0%)
**Headache**	**Baseline**	14 (52%)	2 (7%)	1 (4%)	0 (0%)	10 (37%)	14 (48%)	2 (7%)	1 (3%)	1 (3%)	11 (38%)
	**Day 7**	15 (71%)	2 (10%)	0 (0%)	0 (0%)	4 (19%)	15 (65%)	1 (4%)	2 (9%)	0 (0%)	5 (22%)
**Sore throat**	**Baseline**	11 (41%)	3 (11%)	5 (19%)	0 (0%)	8 (30%)	8 (28%)	4 (14%)	4 (14%)	4 (14%)	9 (31%)
	**Day 7**	15 (71%)	1 (5%)	1 (5%)	0 (0%)	4 (19%)	12 (52%)	5 (22%)	1 (4%)	0 (0%)	5 (22%)

For peer review only - http://bmjopen.bmj.com/site/about/guidelines.xhtml

		Co-amoxiclav<br>Baseline (N = 27)<br>Day 7 (N = 21)					Placebo<br>Baseline (N = 29)<br>Day 7 (N = 23)				
		**No Problem**	**Minor Problem**	**Moderate Problem**	**Major Problem**	**Missing**	**No Problem**	**Minor Problem**	**Moderate Problem**	**Major Problem**	**Missing**
**Muscle aches and pains**	**Baseline**	14 (52%)	3 (11%)	1 (4%)	0 (0%)	9 (33%)	10 (34%)	2 (7%)	3 (10%)	1 (3%)	13 (45%)
	**Day 7**	16 (76%)	1 (5%)	0 (0%)	0 (0%)	4 (19%)	15 (65%)	1 (4%)	1 (4%)	0 (0%)	6 (26%)
**Fever**	**Baseline**	9 (33%)	6 (22%)	7 (26%)	5 (19%)	0 (0%)	5 (17%)	10 (34%)	11 (38%)	3 (10%)	0 (0%)
	**Day 7**	19 (90%)	2 (10%)	0 (0%)	0 (0%)	0 (0%)	20 (87%)	3 (13%)	0 (0%)	0 (0%)	0 (0%)
**Cough**	**Baseline**	3 (11%)	6 (22%)	9 (33%)	9 (33%)	0 (0%)	1 (3%)	6 (21%)	14 (48%)	8 (28%)	0 (0%)
	**Day 7**	7 (33%)	11 (52%)	2 (10%)	1 (5%)	0 (0%)	5 (22%)	4 (17%)	9 (39%)	5 (22%)	0 (0%)
**Nasal congestion, runny nose**	**Baseline**	1 (4%)	8 (30%)	13 (48%)	5 (19%)	0 (0%)	1 (3%)	6 (21%)	15 (52%)	7 (24%)	0 (0%)
	**Day 7**	9 (43%)	8 (38%)	3 (14%)	1 (5%)	0 (0%)	3 (13%)	4 (17%)	12 (52%)	4 (17%)	0 (0%)
**Vomiting**	**Baseline**	21 (78%)	4 (15%)	0 (0%)	2 (7%)	0 (0%)	22 (76%)	3 (10%)	3 (10%)	1 (3%)	0 (0%)
	**Day 7**	20 (95%)	1 (5%)	0 (0%)	0 (0%)	0 (0%)	20 (87%)	3 (13%)	0 (0%)	0 (0%)	0 (0%)
**Not interested in what's going on**	**Baseline**	19 (70%)	4 (15%)	4 (15%)	0 (0%)	0 (0%)	17 (59%)	10 (34%)	2 (7%)	0 (0%)	0 (0%)
	**Day 7**	18 (86%)	3 (14%)	0 (0%)	0 (0%)	0 (0%)	18 (78%)	3 (13%)	2 (9%)	0 (0%)	0 (0%)
**Unable to get out of bed**	**Baseline**	23 (85%)	3 (11%)	1 (4%)	0 (0%)	0 (0%)	26 (90%)	2 (7%)	1 (3%)	0 (0%)	0 (0%)
	**Day 7**	21 (100%)	0 (0%)	0 (0%)	0 (0%)	0 (0%)	22 (96%)	1 (4%)	0 (0%)	0 (0%)	0 (0%)

Note: The scoring algorithm used allowed for the total score to be calculated if there was missing data in items 10-12, but not in any other items.

For peer review only

For peer review only - http://bmjopen.bmj.com/site/about/guidelines.xhtml

Supplemental Table 3i: Responses to CARIFS domains for children aged 2+ years (only including questionnaires with sufficient data to calculate the overall CARIFS score)

		**Co-amoxiclav** Baseline (N = 72) Day 7 (N = 45)					**Placebo** Baseline (N = 68) Day 7 (N = 34)				
		**No Problem**	**Minor Problem**	**Moderate Problem**	**Major Problem**	**Missing**	**No Problem**	**Minor Problem**	**Moderate Problem**	**Major Problem**	**Missing**
**Poor appetite**	**Baseline**	14 (19%)	26 (36%)	25 (35%)	7 (10%)	0 (0%)	12 (18%)	20 (29%)	30 (44%)	6 (9%)	0 (0%)
	**Day 7**	24 (53%)	17 (38%)	3 (7%)	1 (2%)	0 (0%)	12 (35%)	16 (47%)	4 (12%)	2 (6%)	0 (0%)
**Not sleeping well**	**Baseline**	10 (14%)	18 (25%)	33 (46%)	11 (15%)	0 (0%)	12 (18%)	17 (25%)	26 (38%)	13 (19%)	0 (0%)
	**Day 7**	31 (69%)	7 (16%)	5 (11%)	2 (4%)	0 (0%)	13 (38%)	12 (35%)	5 (15%)	4 (12%)	0 (0%)
**Irritable, cranky, fussy**	**Baseline**	13 (18%)	22 (31%)	30 (42%)	7 (10%)	0 (0%)	13 (19%)	19 (28%)	28 (41%)	8 (12%)	0 (0%)
	**Day 7**	18 (40%)	20 (44%)	4 (9%)	3 (7%)	0 (0%)	15 (44%)	11 (32%)	4 (12%)	4 (12%)	0 (0%)
**Feels unwell**	**Baseline**	1 (1%)	18 (25%)	39 (54%)	14 (19%)	0 (0%)	7 (10%)	16 (24%)	30 (44%)	15 (22%)	0 (0%)
	**Day 7**	27 (60%)	12 (27%)	4 (9%)	2 (4%)	0 (0%)	19 (56%)	7 (21%)	5 (15%)	3 (9%)	0 (0%)
**Low energy tired**	**Baseline**	7 (10%)	18 (25%)	36 (50%)	11 (15%)	0 (0%)	11 (16%)	19 (28%)	27 (40%)	11 (16%)	0 (0%)
	**Day 7**	23 (51%)	14 (31%)	7 (16%)	1 (2%)	0 (0%)	17 (50%)	10 (29%)	4 (12%)	3 (9%)	0 (0%)
**Not playing well**	**Baseline**	15 (21%)	27 (38%)	26 (36%)	4 (6%)	0 (0%)	24 (35%)	17 (25%)	18 (26%)	9 (13%)	0 (0%)
	**Day 7**	34 (76%)	9 (20%)	1 (2%)	1 (2%)	0 (0%)	22 (65%)	6 (18%)	5 (15%)	1 (3%)	0 (0%)
**Crying more than usual**	**Baseline**	30 (42%)	18 (25%)	20 (28%)	4 (6%)	0 (0%)	28 (41%)	18 (26%)	14 (21%)	8 (12%)	0 (0%)
	**Day 7**	28 (62%)	10 (22%)	5 (11%)	2 (4%)	0 (0%)	21 (62%)	9 (26%)	2 (6%)	2 (6%)	0 (0%)
**Needing extra care**	**Baseline**	16 (22%)	24 (33%)	28 (39%)	4 (6%)	0 (0%)	16 (24%)	22 (32%)	23 (34%)	7 (10%)	0 (0%)
	**Day 7**	29 (64%)	12 (27%)	2 (4%)	2 (4%)	0 (0%)	21 (62%)	11 (32%)	1 (3%)	1 (3%)	0 (0%)

For peer review only

For peer review only - http://bmjopen.bmj.com/site/about/guidelines.xhtml

		Co-amoxiclav<br>Baseline (N = 72)<br>Day 7 (N = 45)					Placebo<br>Baseline (N = 68)<br>Day 7 (N = 34)				
		No Problem	Minor Problem	Moderate Problem	Major Problem	Missing	No Problem	Minor Problem	Moderate Problem	Major Problem	Missing
**Clinginess**	**Baseline**	14 (19%)	20 (28%)	29 (40%)	9 (13%)	0 (0%)	21 (31%)	19 (28%)	20 (29%)	8 (12%)	0 (0%)
	**Day 7**	30 (67%)	10 (22%)	3 (7%)	2 (4%)	0 (0%)	23 (68%)	8 (24%)	2 (6%)	1 (3%)	0 (0%)
**Headache**	**Baseline**	24 (33%)	18 (25%)	23 (32%)	1 (1%)	6 (8%)	30 (44%)	15 (22%)	9 (13%)	7 (10%)	7 (10%)
	**Day 7**	32 (71%)	5 (11%)	2 (4%)	1 (2%)	5 (11%)	28 (82%)	3 (9%)	1 (3%)	2 (6%)	0 (0%)
**Sore throat**	**Baseline**	18 (25%)	18 (25%)	26 (36%)	8 (11%)	2 (3%)	24 (35%)	17 (25%)	12 (18%)	11 (16%)	4 (6%)
	**Day 7**	32 (71%)	7 (16%)	2 (4%)	1 (2%)	3 (7%)	21 (62%)	7 (21%)	3 (9%)	1 (3%)	2 (6%)
**Muscle aches and pains**	**Baseline**	27 (38%)	15 (21%)	20 (28%)	5 (7%)	5 (7%)	28 (41%)	17 (25%)	8 (12%)	10 (15%)	5 (7%)
	**Day 7**	28 (62%)	9 (20%)	0 (0%)	2 (4%)	6 (13%)	23 (68%)	7 (21%)	3 (9%)	1 (3%)	0 (0%)
**Fever**	**Baseline**	6 (8%)	29 (40%)	28 (39%)	9 (13%)	0 (0%)	11 (16%)	20 (29%)	23 (34%)	14 (21%)	0 (0%)
	**Day 7**	42 (93%)	2 (4%)	1 (2%)	0 (0%)	0 (0%)	28 (82%)	1 (3%)	3 (9%)	2 (6%)	0 (0%)
**Cough**	**Baseline**	1 (1%)	12 (17%)	43 (60%)	16 (22%)	0 (0%)	2 (3%)	13 (19%)	24 (35%)	29 (43%)	0 (0%)
	**Day 7**	14 (31%)	18 (40%)	9 (20%)	4 (9%)	0 (0%)	8 (24%)	14 (41%)	6 (18%)	6 (18%)	0 (0%)
**Nasal congestion, runny nose**	**Baseline**	4 (6%)	14 (19%)	37 (51%)	17 (24%)	0 (0%)	6 (9%)	18 (26%)	26 (38%)	18 (26%)	0 (0%)
	**Day 7**	12 (27%)	25 (56%)	8 (18%)	0 (0%)	0 (0%)	13 (38%)	15 (44%)	4 (12%)	2 (6%)	0 (0%)
**Vomiting**	**Baseline**	52 (72%)	17 (24%)	3 (4%)	0 (0%)	0 (0%)	58 (85%)	6 (9%)	2 (3%)	2 (3%)	0 (0%)
	**Day 7**	42 (93%)	2 (4%)	0 (0%)	1 (2%)	0 (0%)	31 (91%)	3 (9%)	0 (0%)	0 (0%)	0 (0%)
**Not interested in what's going on**	**Baseline**	41 (57%)	22 (31%)	8 (11%)	1 (1%)	0 (0%)	38 (56%)	16 (24%)	9 (13%)	5 (7%)	0 (0%)
	**Day 7**	40 (89%)	4 (9%)	0 (0%)	1 (2%)	0 (0%)	29 (85%)	3 (9%)	1 (3%)	1 (3%)	0 (0%)

For peer review only - http://bmjopen.bmj.com/site/about/guidelines.xhtml

		Co-amoxiclav Baseline (N = 72) Day 7 (N = 45)					Placebo Baseline (N = 68) Day 7 (N = 34)				
		**No Problem**	**Minor Problem**	**Moderate Problem**	**Major Problem**	**Missing**	**No Problem**	**Minor Problem**	**Moderate Problem**	**Major Problem**	**Missing**
**Unable to get out of bed**	**Baseline**	56 (78%)	13 (18%)	3 (4%)	0 (0%)	0 (0%)	48 (71%)	12 (18%)	7 (10%)	1 (1%)	0 (0%)
	**Day 7**	41 (91%)	3 (7%)	1 (2%)	0 (0%)	0 (0%)	30 (88%)	2 (6%)	2 (6%)	0 (0%)	0 (0%)

Note: The scoring algorithm used allowed for the total score to be calculated if there was missing data in items 10-12, but not in any other items.

For peer review only

For peer review only - http://bmjopen.bmj.com/site/about/guidelines.xhtml

Supplemental File 4: Additional summaries of research use

Supplemental Table 4a: Details of all reported hospital admissions

	Co-amoxiclav (N=133)	Placebo (N=132)	Total (N=265)
**Hospital admissions**			
**0**	126 (95%)	125 (95%)	251 (95%)
**1**	6 (5%)	6 (5%)	12 (5%)
**2**	1 (1%)	1 (1%)	2 (1%)
**Total nights in hospital***			
**0**	126 (95%)	125 (95%)	251 (95%)
**1**	4 (3%)	1 (1%)	5 (2%)
**2**	1 (1%)	3 (2%)	4 (2%)
**3**	0 (0%)	1 (1%)	1 (0%)
**7**	1 (1%)	1 (1%)	2 (1%)
**9**	1 (1%)	0 (0%)	1 (0%)
**10**	0 (0%)	1 (1%)	1 (0%)
**Antibiotics received during hospital admission***			
**0**	128 (96%)	128 (97%)	256 (97%)
**1**	4 (3%)	4 (3%)	8 (3%)
**2**	1 (1%)	0 (0%)	1 (0%)
**Other drugs received during hospital admission***			
**0**	128 (96%)	126 (95%)	254 (96%)
**1**	2 (2%)	1 (1%)	3 (1%)
**2**	1 (1%)	3 (2%)	4 (2%)
**3**	1 (1%)	2 (2%)	3 (1%)
**6**	1 (1%)	0 (0%)	1 (0%)
**X-rays during hospital admission***			
**0**	131 (98%)	129 (98%)	260 (98%)
**1**	1 (1%)	3 (2%)	4 (2%)
**2**	1 (1%)	0 (0%)	1 (0%)
**Other investigations during hospital admission****			
**0**	131 (98%)	130 (98%)	261 (98%)
**1**	2 (2%)	2 (2%)	4 (2%)
**Emergency department visits**			
**0**	128 (96%)	127 (96%)	255 (96%)
**1**	5 (4%)	5 (4%)	10 (4%)

**The summaries encompass the entire 28-day follow-up period, i.e. participants may have had a total of two x-rays made up of x-rays during two separate hospital admissions. Similarly, the total nights in hospital may have accumulated from more than one hospital admission.*

[a] Participants who were reported to have been referred to the hospital team/ emergency department for acute admission during a re-consultation for the same illness episode for which the child was randomised, but for whom no hospital admission is recorded at this date, are classed as having visited emergency department.

Hospital admission episodes for at least one night were collected during the notes review.

The trial did not report any admissions to intensive care units (ICU).

Two participants were reported to have a hospital admission on the same day as their discharge day; these were counted as emergency department visits.

***Other investigations include blood samples (2), throat swap and nasal aspirate.*

For peer review only - http://bmjopen.bmj.com/site/about/guidelines.xhtml

Supplemental Table 4b: Overview of healthcare use for re-consultations due to clinical deterioration and hospitalisations related to the illness episode for which the child was randomised to ARCHIE

Number of	N (Co-amoxiclav)	Co-amoxiclav: Mean (S.D.)	N (Placebo)	Placebo: Mean (S.D.)	N (Difference)	Difference (95% CI)
Re-consultations	133	0.27 (0.49)	132	0.23 (0.48)	265	0.04 (95% CI -0.07, 0.15), p-value = 0.495
Antibiotics received at re-consultation	133	0.10 (0.30)	132	0.10 (0.30)	265	0.00 (95% CI -0.07, 0.07), p-value = 0.993
Other drugs received at re-consultation	133	0.06 (0.27)	132	0.09 (0.49)	265	-0.03 (95% CI -0.12, 0.07), p-value = 0.535
Chest x-rays at re-consultation	133	0.03 (0.17)	132	0.01 (0.09)	265	0.02 (95% CI -0.01, 0.06), p-value = 0.178
Other interventions at re-consultation	133	0.02 (0.12)	132	0.00 (0.00)	265	0.01 (95% CI -0.01, 0.04), p-value = 0.163
Hospital admissions	133	0.04 (0.19)	132	0.05 (0.22)	265	-0.01 (95% CI -0.06, 0.03), p-value = 0.511
Total nights in hospital	133	0.09 (0.65)	132	0.14 (0.74)	265	-0.05 (95% CI -0.18, 0.08), p-value = 0.432
Antibiotics received during hospital admission	133	0.02 (0.15)	132	0.02 (0.15)	265	0.00 (95% CI -0.03, 0.03), p-value = 0.976
Other drugs received during hospital admission	133	0.05 (0.33)	132	0.09 (0.45)	265	-0.04 (95% CI -0.13, 0.05), p-value = 0.398
X-rays during hospital admission	133	0.01 (0.09)	132	0.02 (0.12)	265	-0.01 (95% CI -0.03, 0.02), p-value = 0.596
Other investigations during hospital admission	133	0.00 (0.00)	132	0.02 (0.12)	265	-0.01 (95% CI -0.04, 0.01), p-value = 0.168
Emergency department visits	133	0.03 (0.17)	132	0.03 (0.17)	265	0.00 (95% CI -0.04, 0.04), p-value = 0.983

**Differences have been adjusted for the stratification factors age (used as continuous variable) and seasonal influenza vaccination status. Clustering by centre has been accounted for using the 'cluster' option in Stata's 'regress' command and robust standard errors were generated.*

For peer review only

For peer review only - http://bmjopen.bmj.com/site/about/guidelines.xhtml

Supplemental File 5: Number of days children were unable to attend school or nursery, and subsequent changes to childcare requirements

Supplemental Table 5: Daily activity and child care

	Co-amoxiclav					Placebo					Difference		
	N	Mean (S.D.)	Median	IQR	Range	N	Mean (S.D.)	Median	IQR	Range	N	Difference (95% CI)*	p-value
**Days of school/ nursery missed**													
**week 1**	53	2.6 (2.2)	3	1, 4	0, 7	49	2.4 (2.2)	2	1, 3	0, 12	102	0.1 (95% CI -0.8, 1.0)	0.832
**week 2**	44	0.5 (1.5)	0	0, 0	0, 7	39	0.6 (1.5)	0	0, 0	0, 7	83	-0.2 (95% CI -0.8, 0.5)	0.630
**week 3**	36	0.3 (1.0)	0	0, 0	0, 5	33	0.1 (0.5)	0	0, 0	0, 3	69	0.1 (95% CI -0.2, 0.5)	0.492
**week 4**	38	0.0 (0.2)	0	0, 0	0, 1	26	0.2 (1.0)	0	0, 0	0, 5	64	-0.2 (95% CI -0.6, 0.2)	0.404
**Days of work missed****													
**week 1**	45	1.1 (1.3)	0	0, 2	0, 4	35	1.3 (1.4)	1	0, 2	0, 5	80	-0.2 (95% CI -0.8, 0.4)	0.463
**week 2**	41	0.1 (0.3)	0	0, 0	0, 1	33	0.2 (0.7)	0	0, 0	0, 3	74	-0.1 (95% CI -0.3, 0.1)	0.345
**week 3**	34	0.1 (0.5)	0	0, 0	0, 2	27	0.0 (0.0)	0	0, 0	0, 0	61	0.1 (95% CI -0.1, 0.3)	0.205
**week 4**	33	0.0 (0.0)	0	0, 0	0, 0	22	0.3 (1.1)	0	0, 0	0, 5	55	-0.3 (95% CI -0.7, 0.2)	0.246
**Days on which usual activities were changed*****													
**week 1**	37	2.0 (2.4)	2	0, 4	0, 7	34	2.8 (2.0)	3	1, 4	0, 7	71	-0.8 (95% CI -1.8, 0.2)	0.132
**week 2**	33	0.8 (2.1)	0	0, 0	0, 7	37	0.8 (1.6)	0	0, 0	0, 5	70	0.1 (95% CI -0.8, 1.0)	0.833
**week 3**	26	0.2 (0.7)	0	0, 0	0, 3	32	0.5 (1.7)	0	0, 0	0, 7	58	0.0 (95% CI -0.6, 0.5)	0.877
**week 4**	26	0.0 (0.2)	0	0, 0	0, 1	23	0.3 (0.8)	0	0, 0	0, 3	49	-0.2 (95% CI -0.6, 0.1)	0.150
**Days outside carer required**													
**week 1**	40	0.6 (1.2)	0	0, 1	0, 4	31	0.8 (2.1)	0	0, 1	0, 11	71	-0.3 (95% CI -1.2, 0.6)	0.520
**week 2**	40	0.3 (1.0)	0	0, 0	0, 5	34	0.1 (0.3)	0	0, 0	0, 2	74	0.2 (95% CI -0.2, 0.5)	0.344
**week 3**	34	0.1 (0.5)	0	0, 0	0, 2	34	0.1 (0.5)	0	0, 0	0, 3	68	0.0 (95% CI -0.2, 0.2)	0.933
**week 4**	34	0.2 (1.0)	0	0, 0	0, 6	30	0.1 (0.5)	0	0, 0	0, 3	64	0.1 (95% CI -0.3, 0.5)	0.637

**Differences have been adjusted for the stratification factors age (used as continuous variable) and seasonal influence vaccination status. Clustering by centre has been accounted for using the 'cluster' option in Stata's 'regress' command and robust standard errors were generated.*

***Collected for parents/ carers in paid employment; *** Collected for parents/ carers not in paid employment*

SD: Standard deviation; IQR: Interquartile range

For peer review only - http://bmjopen.bmj.com/site/about/guidelines.xhtml

CHEERS Checklist

Items to include when reporting economic evaluations of health interventions

The **ISPOR CHEERS Task Force Report**, *Consolidated Health Economic Evaluation Reporting Standards (CHEERS)—Explanation and Elaboration: A Report of the ISPOR Health Economic Evaluations Publication Guidelines Good Reporting Practices Task Force*, provides examples and further discussion of the 24-item CHEERS Checklist and the CHEERS Statement. It may be accessed via the *Value in Health* or via the ISPOR Health Economic Evaluation Publication Guidelines – CHEERS: Good Reporting Practices webpage: http://www.ispor.org/TaskForces/EconomicPubGuidelines.asp

Section/item	Item No	Recommendation	Reported on page No/ line No
**Title and abstract**			
Title	1	Identify the study as an economic evaluation or use more specific terms such as “cost-effectiveness analysis”, and describe the interventions compared.	Page 1, lines1-3
Abstract	2	Provide a structured summary of objectives, perspective, setting, methods (including study design and inputs), results (including base case and uncertainty analyses), and conclusions.	Page 3 line 1 to page 4 line 3
**Introduction**			
Background and objectives	3	Provide an explicit statement of the broader context for the study.<br>Present the study question and its relevance for health policy or practice decisions.	Page 6 lines 2-6<br>Page 6 lines 19-24
**Methods**			
Target population and subgroups	4	Describe characteristics of the base case population and subgroups analysed, including why they were chosen.	Page 7 line 11-22
Setting and location	5	State relevant aspects of the system(s) in which the decision(s) need(s) to be made.	P7 L6-8
Study perspective	6	Describe the perspective of the study and relate this to the costs being evaluated.	P7 L23/24
Comparators	7	Describe the interventions or strategies being compared and state why they were chosen.	P8 L5-10
Time horizon	8	State the time horizon(s) over which costs and consequences are being evaluated and say why appropriate.	P7 L7-8
Discount rate	9	Report the choice of discount rate(s) used for costs and outcomes and say why appropriate.	P11 L19
Choice of health outcomes	10	Describe what outcomes were used as the measure(s) of benefit in the evaluation and their relevance for the type of analysis performed.	P8 L 14 to P10 L 25
Measurement of effectiveness	11a	*Single study-based estimates:* Describe fully the design features of the single effectiveness study and why the single study was a sufficient source of clinical effectiveness data.	P7&8 P6 L20-26

For peer review only

	11b	*Synthesis-based estimates:* Describe fully the methods used for identification of included studies and synthesis of clinical effectiveness data.	N/A
Measurement and valuation of preference based outcomes	12	If applicable, describe the population and methods used to elicit preferences for outcomes.	N/A
Estimating resources and costs	13a	*Single study-based economic evaluation:* Describe approaches used to estimate resource use associated with the alternative interventions. Describe primary or secondary research methods for valuing each resource item in terms of its unit cost. Describe any adjustments made to approximate to opportunity costs.	Page 8 line 8 to page 9 line 18
	13b	*Model-based economic evaluation:* Describe approaches and data sources used to estimate resource use associated with model health states. Describe primary or secondary research methods for valuing each resource item in terms of its unit cost. Describe any adjustments made to approximate to opportunity costs.	N/A
Currency, price date, and conversion	14	Report the dates of the estimated resource quantities and unit costs. Describe methods for adjusting estimated unit costs to the year of reported costs if necessary. Describe methods for converting costs into a common currency base and the exchange rate.	P 11 L4 -19
Choice of model	15	Describe and give reasons for the specific type of decision-analytical model used. Providing a figure to show model structure is strongly recommended.	N/A
Assumptions	16	Describe all structural or other assumptions underpinning the decision-analytical model.	N/A
Analytical methods	17	Describe all analytical methods supporting the evaluation. This could include methods for dealing with skewed, missing, or censored data; extrapolation methods; methods for pooling data; approaches to validate or make adjustments (such as half cycle corrections) to a model; and methods for handling population heterogeneity and uncertainty.	P11 L23 to P13 L 23
**Results**			
Study parameters	18	Report the values, ranges, references, and, if used, probability distributions for all parameters. Report reasons or sources for distributions used to represent uncertainty where appropriate. Providing a table to show the input values is strongly recommended.	N/A
Incremental costs and outcomes	19	For each intervention, report mean values for the main categories of estimated costs and outcomes of interest, as well as mean differences between the comparator groups. If applicable, report incremental cost-effectiveness ratios.	Tables 3 & 5
Characterising uncertainty	20a	*Single study-based economic evaluation:* Describe the effects of sampling uncertainty for the estimated incremental cost and incremental effectiveness parameters, together with the impact	Tables 3 & 5

For peer review only

		of methodological assumptions (such as discount rate, study perspective).	
	20b	*Model-based economic evaluation:* Describe the effects on the results of uncertainty for all input parameters, and uncertainty related to the structure of the model and assumptions.	N/A
Characterising heterogeneity	21	If applicable, report differences in costs, outcomes, or cost-effectiveness that can be explained by variations between subgroups of patients with different baseline characteristics or other observed variability in effects that are not reducible by more information.	Figure 1
**Discussion**			
Study findings, limitations, generalisability, and current knowledge	22	Summarise key study findings and describe how they support the conclusions reached. Discuss limitations and the generalisability of the findings and how the findings fit with current knowledge.	P27 L5to P32 L6
**Other**			
Source of funding	23	Describe how the study was funded and the role of the funder in the identification, design, conduct, and reporting of the analysis. Describe other non-monetary sources of support.	page 34 line 1-6
Conflicts of interest	24	Describe any potential for conflict of interest of study contributors in accordance with journal policy. In the absence of a journal policy, we recommend authors comply with International Committee of Medical Journal Editors recommendations.	page 34 line 8-11

For consistency, the CHEERS Statement checklist format is based on the format of the CONSORT statement checklist

The **ISPOR CHEERS Task Force Report** provides examples and further discussion of the 24-item CHEERS Checklist and the CHEERS Statement. It may be accessed via the *Value in Health* link or via the ISPOR Health Economic Evaluation Publication Guidelines – CHEERS: Good Reporting Practices webpage: http://www.ispor.org/TaskForces/EconomicPubGuidelines.asp

The citation for the CHEERS Task Force Report is:
Husereau D, Drummond M, Petrou S, et al. Consolidated health economic evaluation reporting standards (CHEERS)—Explanation and elaboration: A report of the ISPOR health economic evaluations publication guidelines good reporting practices task force. Value Health 2013;16:231-50.

For peer review only
